# Mechanism of the Bifunctional Multiple Product Sesterterpene Synthase AcAS from *Aspergillus calidoustus*

**DOI:** 10.1002/anie.202117273

**Published:** 2022-02-04

**Authors:** Zhiyang Quan, Anwei Hou, Bernd Goldfuss, Jeroen S. Dickschat

**Affiliations:** ^1^ Kekulé-Institute for Organic Chemistry and Biochemistry University of Bonn Gerhard-Domagk-Straße 1 53121 Bonn Germany; ^2^ Department of Chemistry University of Cologne Greinstraße 4 50939 Cologne Germany

**Keywords:** DFT Calculations, Enzyme Mechanisms, Isotopes, Substrate Analogues, Terpenoids

## Abstract

The multiproduct chimeric sesterterpene synthase AcAS from *Aspergillus calidoustus* yielded spirocyclic calidoustene, which exhibits a novel skeleton, besides five known sesterterpenes. The complex cyclisation mechanism to all six compounds was investigated by isotopic labelling experiments in combination with DFT calculations. Chemically synthesised 8‐hydroxyfarnesyl diphosphate was converted with isopentenyl diphosphate and AcAS into four oxygenated sesterterpenoids that structurally resemble cytochrome P450 oxidation products of the sesterterpene hydrocarbons. Protein engineering of AcAS broadened the substrate scope and gave significantly improved enzyme yields.

## Graphical abstract

A sesterterpene synthase from *Aspergillus calidoustus* was characterised that produces calidoustene, a compound with a new skeleton, besides five known compounds. A deep mechanistic investigation through isotopic labelling experiments, site‐directed mutagenesis and DFT calculations is presented. The substrate analog 8‐hydroxy‐FPP was converted into several oxygenated terpenoids.

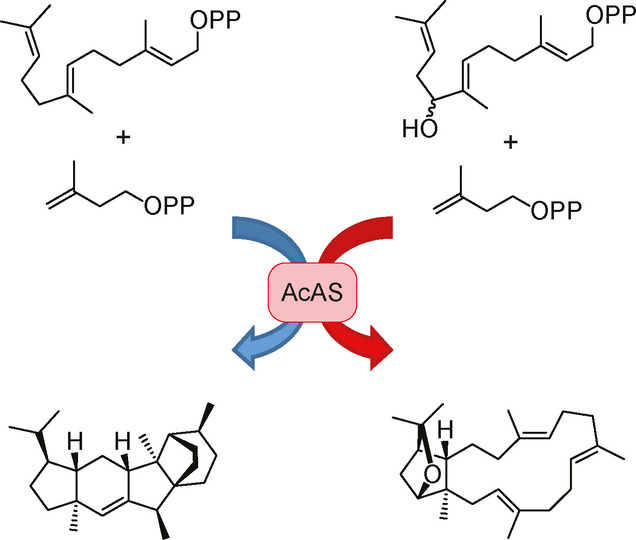

## Introduction

**Figure 1:**
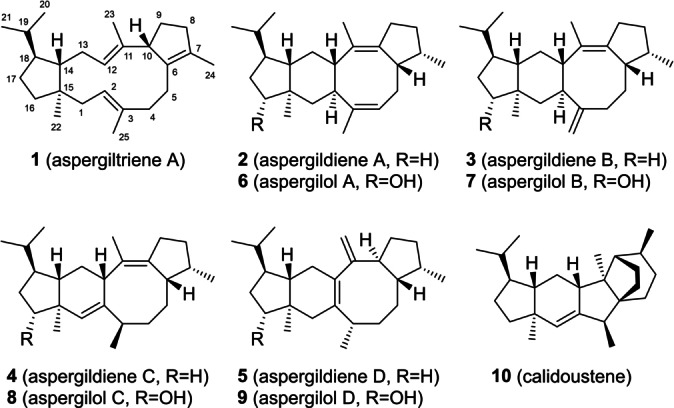
Compounds derived from AuAS and AcAS.

Class I fungal di‐ and sesterterpene synthases are composed of an N‐terminal prenyltransferase (PT) domain for the generation of geranylgeranyl (GGPP) or geranylfarnesyl diphosphate (GFPP) and a C‐terminal terpene cyclase (TC) domain for further conversion into di‐ or sesterterpenes. Their phylogenetic analysis has revealed that these chimeric enzymes can be divided into two clades that reflect their mechanisms of cyclisation.[^1^, ^2^] In clade I containing only sesterterpene synthases, the cation generated by the departure of pyrophosphate reacts in a 1,15‐14,18 cyclisation with participation of the olefin functions of the 4^th^ and the 5^th^ isoprenyl units to form a 5‐15 bicyclic ring system, as observed in the biosynthesis of sesterfisherol by NfSS,^[1]^ betaestacin I by PbTS1,^[3]^ quiannulatene by EvQS,^[4]^ sesterbrasiliatriene by PbSS,^[5]^ fusoxypene A by FoFS,^[6]^ and preaspterpenacid I by AtAS.^[6]^ In clade II, 1,11‐10,14 cyclisations through attacks of the 3^rd^ and 4^th^ isoprenyl units result in a 5‐11 bicyclic ring system, as found for the biosynthesis of asperterpenol A by AcldAS,^[7]^ ophiobolin F by AcOS^[8]^ and AcldOS,^[9]^ stellatatriene by EvSS,^[10]^ astellifadiene by EvAS,^[11]^ and preasperterpenoid A by TwAS^[12]^ and PvPS^[5]^ (Figure S1). Recently, the novel clade I sesterterpene synthase AuAS from *Aspergillus ustus* was reported that produces the five sesterterpenes aspergiltriene (**1**) and aspergildienes A–D (**2**–**5**). A genetically clustered cytochrome P450 monooxygenase (CYP450) then converts the four aspergildienes into the alcohol derivatives aspergilols A–D (**6**–**9**), following the natural cyclisation‐oxidation sequence of terpene biosynthesis (Figure 1).^[13]^ Here we report on a terpene synthase from *Aspergillus calidoustus* that produces the sesterterpene hydrocarbon calidoustene (**10**) with a novel skeleton, besides the five known compounds **1**–**5**. Isotopic labelling experiments in conjunction with DFT calculations revealed the cyclisation mechanism for all six compounds showing complex rearrangements for **10** and a mechanism for **5** that is different to a previous proposal.^[13]^ In a reversal of the biosynthetic process employing an oxidation‐cyclisation sequence, synthetic 8‐hydroxyfarnesyl diphosphate, a formal oxidation product of farnesyl diphosphate (FPP), was cyclised with AcAS to yield two new aspergilol derivatives, calidoustatriene ether and calidoustol. The yields of the enzymatic reaction catalysed by AcAS were optimised through site‐directed mutagenesis and improved incubation conditions by addition of cyclodextrin.

## Results and Discussion

**Figure 2:**
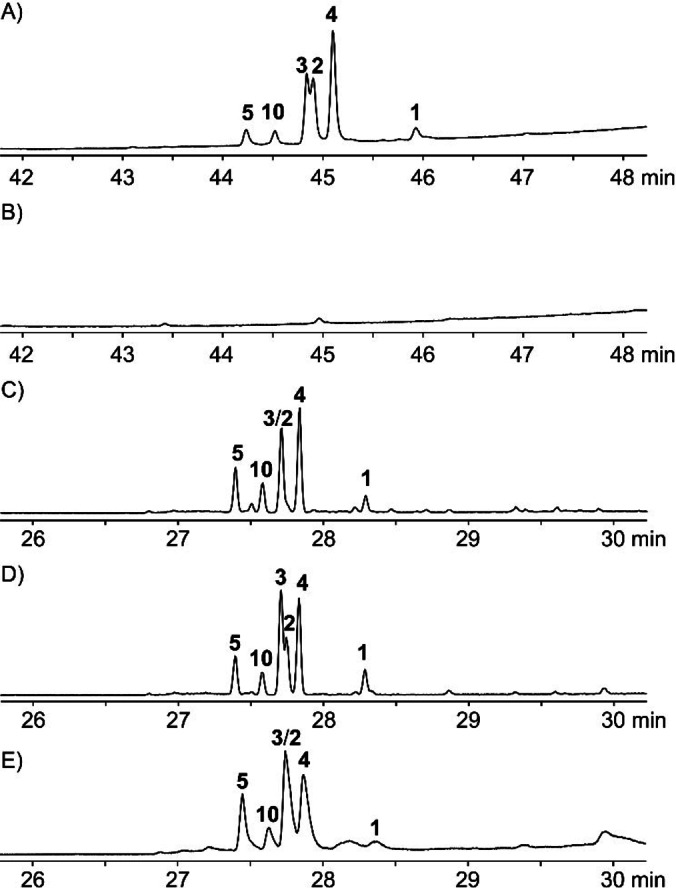
Total ion chromatograms of extracts from A) *A. oryzae* NSAR1 harboring *acAS*, B) negative control *A. oryzae* NSAR1 transformed with empty vector, C) enzyme reaction of GFPP incubated with AcAS‐TC, D) DMAPP+IPP incubated with AcAS‐TC+AcldOS‐PT, and E) DMAPP+IPP incubated with AcAS‐TC‐AcldOS‐PT. The retention times in parts (A) and (B) deviate from those in parts (C)–(E), because different temperature programs were used. The elution order of compounds and EI mass spectra were the same.

The gene encoding the bifunctional terpene synthase AcAS from *A. calidoustus*, which is a homologue of AuAS from *A. ustus* (Figure S2), was cloned into the fungal expression vector pArgB‐TAA^[14]^ to yield pArgB‐AcAS (Table S1) and transformed to *Aspergillus oryzae* NSAR1 using the protoplast‐polyethylene glycol method,^[15]^ resulting in the production of six sesterterpenes, five of which were tentatively identified as the known compounds **1**–**5** by a comparison of mass spectra to published data,^[13]^ besides one new compound **10** (Figures 2A and S3). However, a large‐scale fermentation (4 L) of *A. oryzae* NSAR1‐AcAS gave less than 1 mg L^−1^ of sesterterpenes in total, which was insufficient for compound isolation. Cloning of the coding sequence of AcAS (Figure S4) into the expression vector pYE‐Express, expression in *Escherichia coli* BL21 (DE3), and purification by Ni^2+^‐NTA affinity chromatography (Figure S5) yielded a protein that efficiently converted GFPP into the same six sesterterpenes (Figure 2C). The combinations of GGPP+isopentenyl diphosphate (IPP), farnesyl diphosphate (FPP)+IPP, and geranyl diphosphate (GPP)+IPP resulted in the same products with decreasing efficiency, while dimethylallyl diphosphate (DMAPP)+IPP gave no sesterterpenes (Figure S6), suggesting that AcAS cooperates with FPP synthase (FPPS) in vivo. Based on the crystal structure of *Phomopsis amygdali* fusicoccadiene synthase (PaFS)[^16^, ^17^] and a sequence alignment with AcAS and AcldOS that is known to accept DMAPP^[9]^ truncation sites for the TC and PT domains for both enzymes were identified (Figures S7 and S8). Expression constructs for the PT domain of AcldOS (AcldOS‐PT, C‐terminal 388 amino acids of AcldOS), the TC domain of AcAS (AcAS‐TC, N‐terminal 401 amino acids of AcAS), and a fusion protein AcAS‐TC‐AcldOS‐PT were made available (Figures S9 and S10). The combination of AcAS‐TC and AcldOS‐PT and the fusion protein AcAS‐TC‐AcldOS‐PT also yielded all six sesterterpenes from DMAPP and IPP (Figures 2D and E).

A large‐scale incubation (800×1 mL) of FPP (400 mg) and IPP (400 mg) with AcAS yielded the mixture of all six compounds that were isolated by preparative HPLC. One‐ and two‐dimensional NMR spectroscopy confirmed the structures of **1** to **5** (Tables S2–S6, Figures S11–S50) and allowed to elucidate the structure of **10** (Table S7, Figures S51–S58) that exhibits a new skeleton and for which we suggest the name calidoustene. The enzyme characterised here is designated *Aspergillus calidoustus* Aspergildiene Synthase (AcAS). The absolute configurations of the AcAS products were investigated through stereoselective deuteration (a summary of labelling experiments is given in Table S8). For this purpose, (*E*)‐ and (*Z*)‐(4‐^13^C,4‐^2^H)IPP^[18]^ were incubated with DMAPP and AcldOS‐PT to obtain GFPP with a stereoselective deuteration at C4, C8, C12 and C16 of known configuration.^[19]^ Further conversion with AcAS‐TC introduced corresponding stereogenic centres into the sesterterpenes, and the additional ^13^C‐labels at the deuterated carbons allow for a highly sensitive analysis through HSQC spectroscopy, showing for each experiment one strongly enhanced and one vanished crosspeak for the diastereotopic hydrogens (Figures S59–S64). Together with the NOESY based assignment of their relative orientation with respect to the naturally present stereogenic centres, these data allowed to conclude on the absolute configurations of all six compounds as shown in Figure 1. Additional experiments with AcAS and FPP plus (*R*)‐ or (*S*)‐(1‐^13^C,1‐^2^H)IPP,^[20]^ and (*R*)‐ or (*S*)‐(1‐^13^C,1‐^2^H)FPP^[21]^ plus IPP confirmed these assignments (Figures S65–S70). These results are in line with the reported absolute configuration of **1** that was previously determined through comparison of experimental and calculated ECD spectra,^[13]^ while the absolute configurations of **2**–**5** and **10** are established here.

**Scheme 1:**
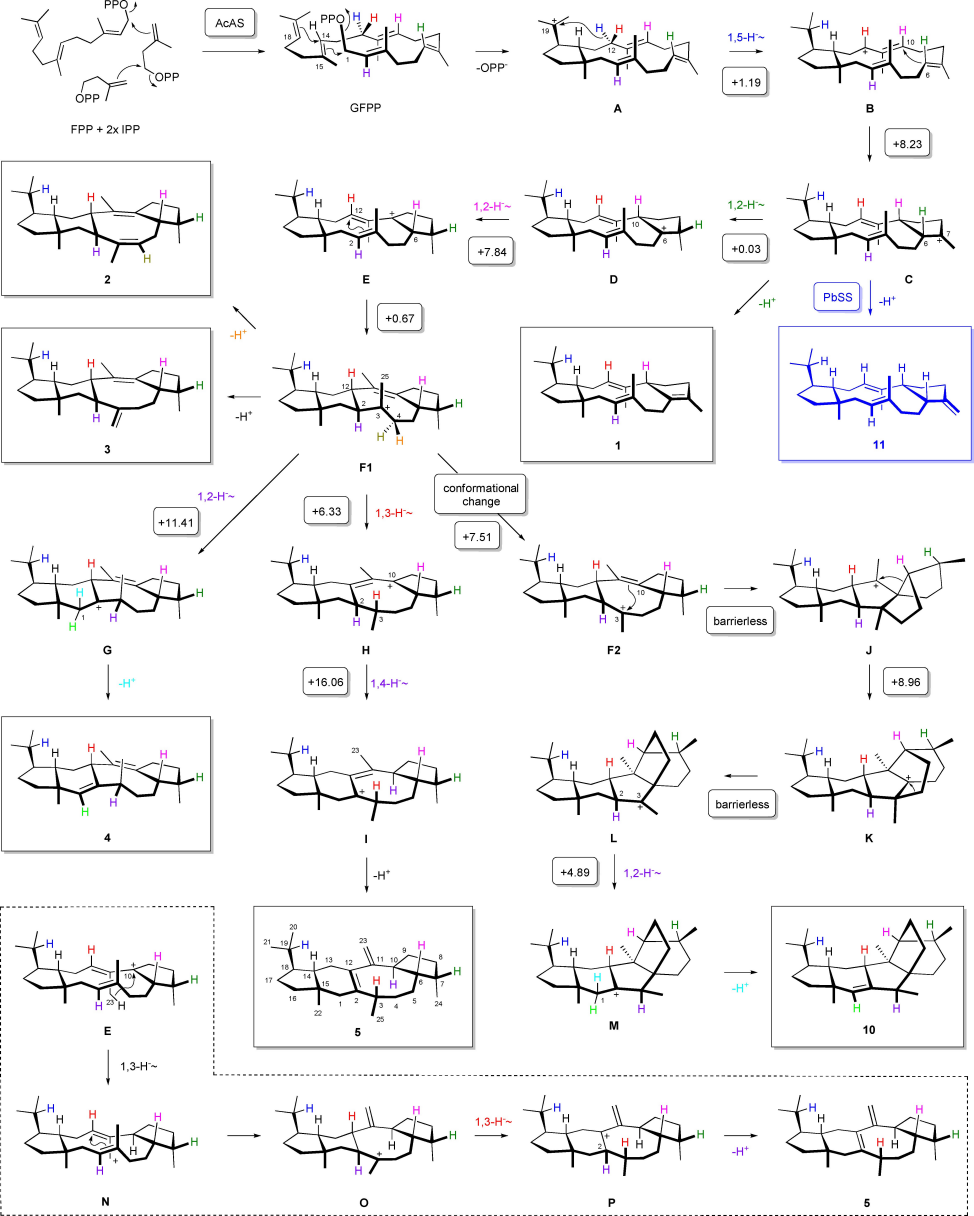
The cyclisation mechanism of AcAS from GFPP to **1**–**5** and **10**. Numbers in round boxes are computed transition state energies in kcal mol^−1^ (mPW1PW91/6‐311+G(d,p)//B97D3/6‐31G**, 298.15 K). The blue box shows the structure of sesterbrasiliatriene (**11**) made by PbSS from *Penicillium brasilianum*.^[5]^ The steps in the dashed box for the formation of **5** were proposed earlier.^[13]^

A mechanistic hypothesis for the conversion of GFPP to the AcAS products starts with a 1,15‐14,18‐cyclisation to **A**, followed by a 1,5‐hydride shift to **B** and 6,10‐cyclisation to **C**, the precursor of **1** by deprotonation (Scheme 1). Two sequential 1,2‐hydride migrations then lead via **D** to **E** that can undergo 2,12‐cyclisation to the central intermediate **F1**. Alternative deprotonations from C4 or C25 result in **2** or **3**, respectively, while another 1,2‐hydride shift can proceed to **G** and **4** by deprotonation. Starting from **F1**, a sequence of 1,3‐ and 1,4‐hydride migrations through **H** to **I** and deprotonation yield **5**. A conformational rearrangement of **F1** to **F2** and 3,10‐ring closure lead to **J**, from which **10** can be explained by Wagner–Meerwein rearrangements to **K** and **L**, 1,2‐hydride transfer to **M**, and deprotonation. The conformational rearrangement in **F** must be assumed, because the hydrogen at C2 in **E** must point down (and consequently Me25 must point up) to achieve the *trans* substitution at the newly closed 6‐membered ring in **F1**, while for the ring closure from **F2** to **J** Me25 must point down to install the correct stereochemistry at C3 in **J**. Related enzyme mechanisms were previously reported for other cyclopentane forming di‐ and sesterterpene synthases and are summarised in a recent review.^[22]^

To obtain evidence for this mechanistic proposal, a series of isotopic labelling experiments were carried out (Table S8). Conversion of all 25 isotopomers of (^13^C)GFPP, enzymatically generated by the PT domain of AcAS from their correspondingly labelled biosynthetic precursors that were synthesised as reported previously,[^5^, ^9^, ^21^, ^23^] resulted in the incorporation of labelling into the correct positions for all compounds **1**–**5** and **10** (Figures S71–S95). In particular, these data confirmed the skeletal rearrangements from **J** to **K** and from **K** to **L** along the pathway to **10**.

The hydride shifts in the biosynthesis of **1**–**5** and **10** were studied through a ^13^C and ^2^H double labelling strategy, in which labelled substrates were designed so that the migrating ^2^H ends up at a ^13^C‐substituted carbon, resulting in slightly upfield shifted triplets in the ^13^C‐NMR spectra as a result of a deuterium isotope effect and ^13^C‐^2^H spin coupling. While the HSQC spectra for H12 (Figures S59–S64) indicate the specific migration of 4H_*E*_ incorporated from IPP (blue H in Scheme 1) into another position, the enzyme conversions of (7‐^13^C)GPP^[5]^ and (*E*)‐ or (*Z*)‐(4‐^13^C,4‐^2^H)IPP with AcldOS‐PT and AcAS‐TC revealed its 1,5‐migration from **A** to **B** for the compounds **2**–**5** (Figure S96). For the minor compound **10** very weak signals could be observed in the ^13^C‐NMR spectra, and no corresponding triplet for **1** was detected, but GC/MS analysis showed migration of the same hydrogen by loss of deuterium for the fragment ion arising by cleavage of the *i*Pr group, if H_*E*_ is substituted by ^2^H (Figures S97–S99).

For compounds **2**–**4** and **10** the 1,2‐hydride shift from **C** to **D** (dark green H) was evident from an incubation of FPP and (3‐^13^C,2‐^2^H)DMAPP^[7]^ with isopentenyl diphosphate isomerase (IDI) from *Serratia plymuthica* ^[24]^ and AcAS (Figures S100 and S101). Only for **5** the broad peak for C7 in the ^13^C‐NMR prevented the observation of a triplet, while for **1** the hydrogen from C6 was lost in the deprotonation to **1** (Figure S102). The same experiment indicated the 1,2‐hydride transfers from **F1** to **G** and from **L** to **M** in the biosynthesis of **4** and **10**, respectively (purple H, Figure S103). Since the experiment with FPP and (3‐^13^C,2‐^2^H)DMAPP does not rule out another mechanism involving hydride migrations from C2 to C7 and from C6 to C2 or C3 (so that the purple H and the dark green H would exchange positions in all molecules), another labelling experiment with conversion of GGPP and (3‐^13^C,2‐^2^H)DMAPP by IDI and AcAS was performed. The results confirmed the 1,2‐hydride shifts towards **4** and **10** (Figure S104), while the slightly upfield shifted signals for C3 of **1**–**3** (between Δ*δ*=−0.03 and −0.10 ppm) indicated a deuterium bound to a neighbouring carbon (C2, Figure S105).

For compound **5** the migration of H2 to C10 in the reaction of **H** to **I** was more difficult to follow, because the ^13^C‐NMR peak for the target carbon was again broad. However, the signal for C11 of **5** is sharp, and therefore the neighbouring effect was used to follow this step. The incubation of (3‐^13^C)FPP^[23b]^ and (2‐^2^H)DMAPP^[25]^ with IDI and AcAS resulted in a slight upfield shift for C11 of **5** (Δ*δ*=−0.03 ppm), in agreement with deuterium bound to C10 (Figure S106). This deuterium must originate from C2, as H6 was shown to migrate to C7 (Figure S96). Furthermore, this finding ruled out a mechanistic alternative for **5** that was proposed earlier (dashed box in Scheme 1),^[13]^ involving a 1,3‐hydride shift from Me23 to C10 (**E** to **N**), followed by cyclisation to **O**, 1,3‐hydride migration to **P** and deprotonation from C2.

The 1,2‐hydride shift from **D** to **E** (pink H) is relevant for **2**–**5** and **10**, and was investigated through conversion of (2‐^2^H)FPP^[21]^ and (2‐^13^C)IPP^[23a]^ with AcAS, yielding triplets for C6 in all cases (Figure S107). Only for **5** peak broadening was observed, like reported for other terpenes before,[^1^, ^20^, ^26^, ^27^] that is a result of special molecular mechanics associated with slowly interconverting conformers. For **5** this phenomenon prevented the detection of the expected triplet signal. The 1,3‐hydride shift from **F1** to **H** in the biosynthesis of **5** (red H) turned out to be the most intricate problem in the mechanistic investigation of AcAS, because not only the target carbon C3, but also all three neigbouring carbons showed peak broadening. In consequence, neither the strategy through detection of a triplet signal (migration of deuterium from C12 to ^13^C‐labelled C3) nor through a neighbouring effect (^13^C‐labelling of a neighbouring carbon, with the deuterium migration to C3 causing a slight upfield shift) could be applied successfully. However, the incubation of (4,4,15,15,15‐^2^H_5_)FPP, synthesised from (^2^H_6_)acetone (Scheme S1, Figures S108–S110), and (5‐^13^C)IPP^[23d]^ with AcAS and GC/MS analysis of the product **5** demonstrated retainment of four deuterium atoms, with loss of one deuterium from Me23 in the final deprotonation step (Figure S111, for the alternative mechanism in the dashed box all five deuterium atoms should be retained). As C12 of **5** is quarternary, both hydrogens must migrate away from this carbon. In the above‐described labelling experiments, the 12‐*pro*‐*S* hydrogen (blue) shifts to C19 (Figures S96–S99), H6 (dark green) moves to C7 (Figures S100 and S101), H2 (purple) to C10 (Figure S106), and H10 (pink) to C6 (Figure S107). The stereoselective deuteration experiments revealed that all hydrogens at C1, C4, C5, C8, C9 and C16 stay at their places (Figures S63 and S69), and assuming that the carbons C13, C14, C17, C18 and C20–C22 in the left portion of **5** do not participate in hydrogen migrations, the only remaining target position for the 12‐*pro*‐*R* hydrogen (red) is C3. Finally, the stereoselective labelling experiments also revealed the specific loss of the 4‐*pro*‐*R* hydrogen (orange) in the deprotonation to **2** (Figure S60) and of the 1‐*pro*‐*R* hydrogen (cyan) in the deprotonations to **4** and **10** (Figures S68 and S70). These hydrogens are located in the top hemispheres of **F**, **G** and **M**, similar to the situation for the abstracted H6 in **C** to **1**, which is the side to which also the diphosphate in the initial cyclisation step to **A** must leave, suggesting that diphosphate may act as the base in these deprotonations.

The cyclisation mechanism of AcAS was also investigated through DFT calculations (Table S9, a complete energy profile is shown in Figure S112). For all reactions from **A** to the central intermediate **F1** low transition state (TS) barriers were found (Scheme 1), similar to the results from DFT calculations reported for sesterfisherol,^[28]^ the product of *Neosartorya fischeri* Sesterfisherol Synthase (NfSS),^[1]^ that has the same 5‐6‐8‐5 ring system, but a different relative configuration. In particular, the conversion from **A** to **B** could be realised with specific migration of the 12‐*pro*‐*S* hydrogen. The transition state barrier to **G** is with +11.41 kcal mol^−1^ slightly higher. The reaction from **F1** to **H** proceeds smoothly, while the subsequent 1,4‐hydride shift to **I** is associated with the highest barrier in the profile (+16.06 kcal mol^−1^), but still achievable at room temperature. The conformational change from **F1** to **F2** results in the direct barrierless cyclisation to **J**. Also the subsequent rearrangement to **K** is directly followed by a barrierless transition to **L** and a smooth 1,2‐hydride shift to **M**. Overall, these computational data further support the experimental findings for the AcAS enzyme mechanism.

**Figure 3:**
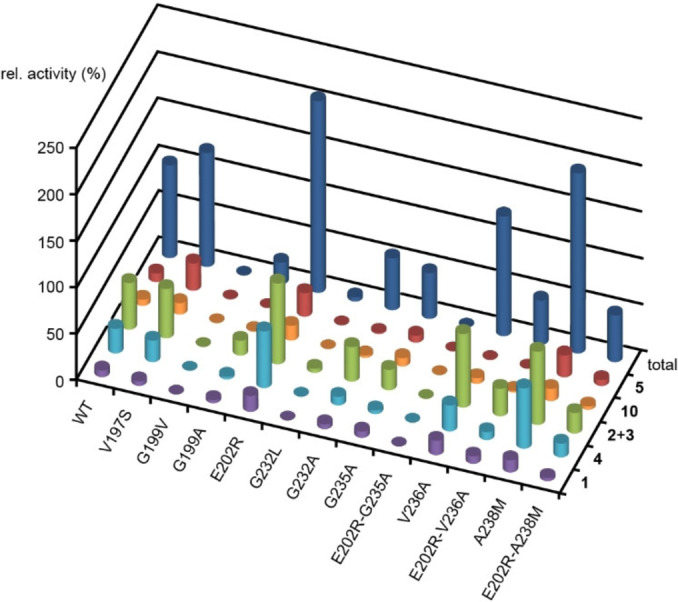
Site‐directed mutagenesis of AcAS. The dark blue cylinders in the top row show the relative total production (wildtype=100 %). The other cylinders show the relative production of compounds **1**–**5** and **10** (green cylinders for the sum of **2** and **3** that coelute in GC). All data are based on peak integrals in the GC from triplicates. Mean and standard deviations are listed in Table S10.

The closest characterised homolog of AcAS and AuAS is the sesterbrasiliatriene (**11**) synthase (PbSS) from *Penicillium brasilianum* (Figure S1). This enzyme is with ca. 20 mg L^−1^ under heterologous expression in *A. oryzae* high yielding. In order to investigate the mechanistic divergence between multiproduct AcAS and the single product PbSS and to increase the activity of AcAS, site‐directed mutagenesis experiments were carried out targeting active site residues of AcAS. A sequence comparison between AcAS and PbSS resulted in the identification of several key residues near the pyrophosphate sensor and the NSE triad (Figure S113). Exchange of the AcAS residues against those found in the analogous positions of PbSS yielded the enzyme variants V197S, G199V, E202R, G232L, G235A, V236A, and A238M. In addition, the Gly residues were exchanged against Ala, giving the variants G199A and G232A. All enzyme variants were expressed, purified and tested for their product profile and relative activity in comparison to the wildtype by incubation with FPP and IPP (Figure 3, Table S10). The V197S variant showed almost the same product composition and activity as the wildtype (123±9 %), while the V236A variant lost the ability to produce **5**, but retained wildtype activity (129±1 %). Several enzyme variants showed a reduced (G199A: 23±4 %, G232L: 4.5±0.7 %, G232A: 56±4 %, G235A: 49±2 %) or completely lost activity (G199V). Two enzyme variants resulted in an increased production of all six compounds (E202R: 207±10 %, A238M: 194±12 %). While the single mutations of E202R and G235A showed increased and moderately reduced activity, respectively, the double exchange E202R‐G235A was inactive. A combination of other successful mutations in the E202R‐V236A and E202R‐A238M variants was also less productive than the single exchanges. Taken together, the mutational studies resulted in the identification of the more productive enzyme variants E202R and A238M, but in no case a new product or simplified product profile was observed, although according to a protein homology model based on the structure of the TC domain of *Fusarium graminearum* GJ1012 synthase (FgGS)[^29^, ^30^] residues G199, G232, G235 and V236 were observed in active site positions (Figure S114). Notably, the effects in terms of reducing the productivity of AcAS were strongest for the exchanges of the putative active site Gly residues against larger residues. It is well known that cyclodextrins can form supramolecular host guest complexes with terpenes.[^31^, ^32^] Accordingly, a further increase of the enzyme yield could be achieved for incubations in the presence of β‐cyclodextrin (10 mm) which was most pronounced for the substrate combination of FPP and IPP with the A238M variant (1700 % of wildtype production in the absence of β‐cyclodextrin, Figure S115).

**Scheme 2:**
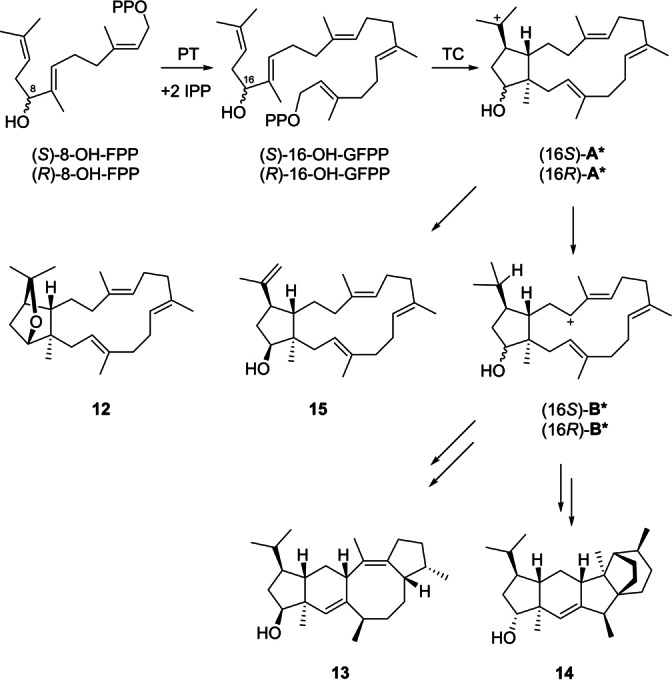
Proposed cyclisation mechanism towards compounds **12**–**15**.

Naturally, the sesterterpene hydrocarbons **2**–**5** are oxidised by a CYP450 that is genetically clustered with AuAS.^[13]^ It has been shown recently by Allemann and co‐workers that 12‐hydroxy‐FPP can be converted by amorphadiene synthase into dihydroartemisinic aldehyde,^[33]^ which is an important intermediate in artemisinin biosynthesis. Also many other studies have shown that terpene synthases can convert non‐natural substrate analogues,^[34]^ but bifunctional fungal terpene synthases with PT and TC domains have not been studied for this aspect. A direct access to the CYP450 oxidation products could be possible from 8‐hydroxy‐FPP (8‐OH‐FPP) that was synthesised (Scheme S2, Figures S116–S118) and incubated with IPP and AcAS, resulting in the formation of four compounds **12**–**15** (Scheme 2, Figure S119) that were isolated and structurally characterised by NMR spectroscopy (Tables S11–S14, Figures S120–S151). The stereoselective deuteration approach using 8‐hydroxy‐FPP in combination with (*E*)‐ and (*Z*)‐(4‐^13^C,4‐^2^H)IPP as well as (*R*)‐ and (*S*)‐(1‐^13^C,1‐^2^H)IPP (Figures S152–S159) revealed the absolute configurations as shown in Scheme 2. Interestingly, the compounds **12**, **13** and **15** are derived from (*S*)‐8‐hydroxy‐FPP, while **14** arises from (*R*)‐8‐hydroxy‐FPP, but the configurations of the stereogenic centres within the carbon skeleton are the same as for the sesterterpene hydrocarbons produced by AcAS. Their formation can be understood by elongation of both enantiomers of 8‐OH‐FPP with IPP to 16‐OH‐GFPP by the AcAS‐PT domain. Subsequent cyclisation of (*S*)‐16‐OH‐GFPP by the AcAS‐TC domain leads to (16*S*)‐A* that yields calidoustatriene ether (**12**) by intramolecular attack of the hydroxy function and 16‐hydroxycalidoustatetraene (**15**) by deprotonation. Alternatively, (16*S*)‐A* can react in a 1,5‐hydride shift to (16*S*)‐B*, and subsequent analogous steps as for **4** in Scheme 1 result in 16‐*epi*‐aspergilol C (**13**), the 16‐epimer of natural product **8**. Starting from (*R*)‐16‐OH‐GFPP, analogous reactions lead to (16*R*)‐A* and (16*R*)‐B* that can be further converted into 16‐hydroxycalidoustene (**14**) by similar reactions as for **10** in Scheme 1. Compound **14** represents the putative natural CYP450 oxidation product of **10**.

## Conclusion

In summary, the bifunctional terpene synthase AcAS producing five known and the unusual spirocyclic compound calidoustene (**10**) was deeply studied. The absolute configurations of all six sesterterpenes were determined through a stereoselective deuteration approach, and the cyclisation mechanism towards each of products was elucidated by isotopic labelling experiments and computational chemistry in great detail, revealing an interesting sequence of hydride transfers for **5** and a remarkable skeletal rearrangement for **10**. Protein engineering through domain swapping yielded an enzyme with broadened substrate scope which was critical for the acceptance of DMAPP and GPP in conjunction with IPP for labelling experiments targeting the first two terpene units, while site‐directed mutagenesis and improvement of incubation conditions by the addition of β‐cyclodextrin resulted in strongly enhanced enzyme activity. Naturally, the sesterterpene hydrocarbons become oxidised at C16 by a tailoring CYP450, but neither the sesterterpene hydrocarbons nor their CYP450 oxidation products could be observed in culture extracts of *A. calidoustus*, suggesting that the corresponding genes are not expressed under laboratory culture conditions. In a reversal of biosynthetic steps, the oxygen functionality was introduced first through chemical synthesis of 8‐hydroxy‐FPP, followed by late‐stage enzymatic elongation with IPP and cyclisation into four new oxygenated sesterterpenes by AcAS whose absolute configurations were likewise addressed through use of stereoselectively deuterated precursors. This work demonstrated for the first time that chimeric bifunctional terpene synthases with their two domains can efficiently convert non‐natural substrate analogues into functionalised sesterterpenes. This approach is particularly interesting, because cytochromes P450 are difficult to handle in heterologous expressions and especially in in vitro reactions. As a chiral catalyst AcAS provides asymmetric active site pockets in its PT and TC domains, and consequently showed the potential to discriminate between the enantiomers of 8‐OH‐FPP, with more efficient conversion of the *S* enantiomer.

## Conflict of interest

The authors declare no conflict of interest.

1

## Supporting information

As a service to our authors and readers, this journal provides supporting information supplied by the authors. Such materials are peer reviewed and may be re‐organized for online delivery, but are not copy‐edited or typeset. Technical support issues arising from supporting information (other than missing files) should be addressed to the authors.

Supporting Information

## Data Availability

The data that support the findings of this study are available from the corresponding author upon reasonable request.
