# Supplementary material for: Mechanism of the Bifunctional Multiple Product Sesterterpene Synthase AcAS from Aspergillus calidoustus
Source: Angew Chem Int Ed Engl. 2022 Feb 4;61(13):e202117273. doi: 10.1002/anie.202117273 (PMC9303889; doi:10.1002/anie.202117273)
Supplement: Supplementary file 1 — Supporting Information [file ANIE-61-0-s001.pdf]

Angewandte Chemie
Eine Zeitschrift der Gesellschaft Deutscher Chemiker

Supporting Information

Mechanism of the Bifunctional Multiple Product Sesterterpene Synthase AcAS from *Aspergillus calidoustus*

*Z. Quan, A. Hou, B. Goldfuss, J. S. Dickschat**

Mechanism of the Bifunctional Multiple Product Sesterterpene Synthase AcAS from *Aspergillus calidoustus*

Zhiyang Quan,[a] Anwei Hou,[a] Bernd Goldfuss,[b] and Jeroen S. Dickschat*[a]

[a] Dr. Z. Quan, Dr. A. Hou, Prof. Dr. J. S. Dickschat
Kekulé-Institute for Organic Chemistry and Biochemistry
University of Bonn
Gerhard-Domagk-Straße 1, 53121 Bonn (Germany)
E-mail: dickschat@uni-bonn.de

[b] Prof. Dr. B. Goldfuss
Department of Chemistry
University of Cologne
Greinstraße 4, 50939 Cologne (Germany)

Table of Contents

General 2
Investigation *in vivo* 5
Primers 6
Investigation *in vitro* 10
Produciton, isolation, and structure elucidation of compound **1-10** 16
NMR data of compound **1-10** 18
Isotopic labeling experiments results 72
Synthesis of (4,4,15,15,15-$^{2}H_{5}$)FPP 123
DFT calculation methods and results 130
Mutation research of AcAS 133
Synthesis of 8-hydroxyl FPP 136
Production, isolation, and structure elucidation of compound **12-15** 144
NMR data of compound **12-15** 148
Isotopic labeling experiments results 184
References 192

Enzyme name

The enzyme investigated in this study was initially named AcldAS (also in the first published version as an Accepted Article. Unfortunately, we missed the point that this enzyme name was already in use for *Aspergillus calidoustus* Asperterpenol A Synthase.[1] During corrections of the page proofs the name of the enzyme investigated in this study was changed to AcAS. The previous name AcldAS should be considered obsolete.

General

Chemicals were purchased from Sigma Aldrich Chemie GmbH (Steinheim, Germany), Carbolution Chemicals GmbH (St. Ingbert, Germany), or Carl Roth (Karlsruhe, Germany) and used without purification. Solvents for column chromatography were purchased in p.a. grade and purified by distillation. Thin-layer chromatography was performed with 0.2 mm precoated plastic sheets Polygram Sil G/UV254 purchased from Machery-Nagel (Düren, Germany). Column chromatography was performed using silica gel 60 (0.040-0.060 nm) purchased from Merck (Darmstadt, Germany).

GC/MS

GC/MS analyses were carried out with an Agilent (Santa Clara, USA) HP 7890B gas chromatograph fitted with a HP5-MS silica capillary column (30 m, 0.25 mm, i. d., 0.50 μm film) connected to a HP 5977A inert mass detector. The GC parameters were 1) inlet pressure: 77.1 kPa, He 23.3 mL min^{-1}, 2) temperature program: 5 min at 50 °C, increasing at 5 or 10 °C min^{-1} to 320 °C, 3) injection volume: 1 or 2 μL, 4) splitless or split ratio 10:1, 60 s valve time and 5) carrier gas: He at 1 mL min^{-1}. The MS parameters were 1) transfer line: 250 °C, and 2) electron energy: 70 eV. Retention indices (*I*) were determined from a homologous series of *n*-alkanes (C8-C40).

NMR spectoscopy

^{1}H NMR, ^{13}C NMR, and ^{31}P NMR spectra were recorded on a Bruker Avance I 500 MHz spectrometer and a Bruker Avance III HD 700 MHz Cryo spectrometer. Chemical shifts were referenced to the residual proton signal of C_6D_6 (δ = 7.16 ppm) for ^{1}H NMR and the ^{13}C signal of C_6D_6 (δ = 128.06) for ^{13}C NMR.[2]

IR spectroscopy

IR spectra were recorded on a Bruker α infrared spectrometer with a diamond ATR probehead. Peak intensities are given as s (strong), m (medium), w (weak) and br (broad).

Optical rotations

Optical rotations were recorded on a Modular Compact Polarimeter MCP 100 (Anton Paar, Graz, Austria). The optical rotation parameters are 1) temperature: 25 °C, 2) wavelength: 589 nm (the sodium D line), and 3) the path-length: 10 cm. The compound concentrations *c* are given in g 100 mL^{-1}.

High resolution MS

High resolution mass spectra were measured with LTQ Orbitrap XL (Thermo Scientific, Waltham, Massachusetts, USA).

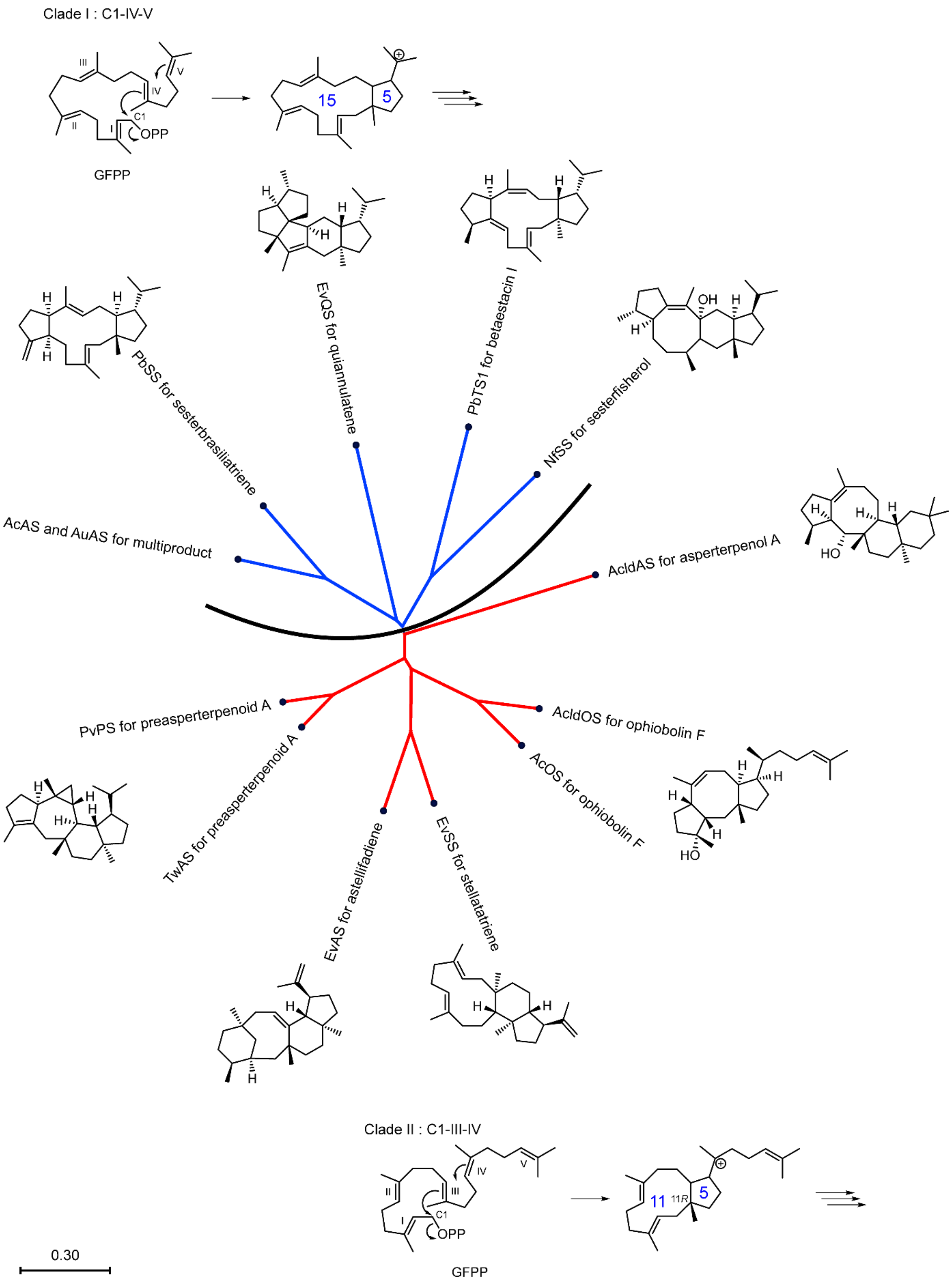

Figure S1. Aspergildiene synthase from *Aspergillus calidoustus* (AcAS) and from *A. ustus* (AuAS) belong to clade I sesterterpene synthases.[1,3-12]

MDAALRDICQLSDPCDPRSFEPPIKDFFCIYPMYRSRYEAKAIQGSNEFLDGWNKAIEKDGLRNDGRPFLGCNTIYGNYVAWAYPECLPERA

MDAALRDICQLSDPCDPRSFEPPIKDFFCIYPMYRSRYEAKAIQGSNEFLDGWNKAIEKDGLRNDGRPFLGCNTIYGNYVAWAYPECLPERA

AHVAAYCDWGFFWDDATDAMSMEKNHEATKDLILTIMSTVGIGQKHEPLLAVNKLVVPFVLNKLAGTDGDLGLNHMKAWKAHLDGQARSSHA

AHVAAYCDWGFFWDDATDAMSMEKNHEATKDLILTIMSTVGIGQKHEPLLAVNKLVVPFVLNKLAGTDGDLGLNHMKAWKAHLDGQARSSHA

NMSWEELKQHRLVEGGPEWAIRLGAWGAGIRCTAEEIESVREIIDIGGIAGVLANDYYSFNKEFDEHSRAGTIERMQNGVALLMREYGYSEE

NMSWEELKQHRLVEGGPEWAIRLGAWGAGIRCTAEEIESVREIIDIGGIAGVLANDYYSFNKEFDEHSRAGTIERMQNGVALLMREYGYSEE

EAREILKKEINKMEQQFMDMYLTWLNGPVQKSRGLIQYLTMVLCLYSGTMFWMAHGARYHRTDLITTAEDRATIIGKCQGDAFRVMEGYPPP

EAREILKKEINKMEQQFMDMYLTWLNGPVQKSRGLIQYLTMVLCLYSGTMFWMAHGARYHRTDLITTAEDRATIIGKCQGDAFRVMEGYPPP

KGLKRTASSPESAPKRRASKANNINQSNGRGGDPMVAFSGPFVKAPSHICDAPYEYIDSLQSKNMRDKFINILNSWLNVPSDSLQIIKNIVQ

KGLKRTASSPESAPKRRASKANNINQSNGRGGDPMVAFSGPFVKAPSHICDAPYEYIDSLQSKNMRDKFINILNSWLNVPSDSLQIIKNIVQ

MLHNSSLMLDDIEDASPLRRGQPATHIFYGASQTINSANFSYVKTVIEATHLKNPQCLQIFLEEVSDLHRGQSLDLHWRHHGRCPTTDEYIM

MLHNSSLMLDDIEDASPLRRGQPATHIFYGASQTINSANFSYVKTVIEATHLKNPQCLQIFLEEVSDLHRGQSLDLHWRHHGRCPTTDEYIM

MVDNKTGGLFRLMARLMEAESPSPITTPHLSRLLTLIGRYYQIRDDYMNLTSADYTTKKGYCEDLDEGKFSLPLIHLLLHTSCPDRITSALY

MVDNKTGGLFRLMARLMEAESPSPITIPHLSRLLTLIGRYYQIRDDYMNLTSADYTTKKGYCEDLDEGKFSLPLIHLLLHTSCPDRITSALY

NRVPSTGLQDEVKTYILDAMQSARTFEYVREVLSHLHGEIMKTLDEAEKTLGINNGVRMLLVGLGL

NRVPSTGLQDEVKTYILDAMQSARTFEYVREVLSHLHGEIMKTLDEAEKTLGINNGVRMLLVGLGL

Figure S2. Amino acid sequence alignment of up: AuAS and down: AcAS. The difference between AuAS and AcAS is highlighted in red. Highly conserved motifs are highlighted in yellow.[13-16]

Strains and culture conditions

Aspergillus calidoustus CBS121601 was obtained from the CBS Culture Collection, Westerdijk Fungal Biodiversity Institute (Utrecht, Netherlands) and grown in DPY medium (2 % dextrin, 1 % polypeptone, 0.5 % yeast extract, 0.05 % $MgSO_4$, 0.5 % KH_2PO_4) at 30 °C. *Aspergillus oryzae* NSAR1, the fungus used for fungal gene heteroexpression, was kindly provided by Prof. K. Gomi (Graduate School of Agricultural Sciences, Tohoku University) and Prof. K. Kitamoto (Graduate School of Agricultural Sciences, The University of Tokyo), and grown in DPY medium at 30 °C. *Escherichia coli* Stellar was purchased from Clontech (Saint-Germain-en-Laye, France), and grown in LB medium at 37 °C. *E. coli* BL21 was purchased from Thermo Scientific (Waltham, Massachusetts, USA), and grown in terrific broth (TB) medium (2.4 % yeast extract, 2.0 % tryptone, 0.4 % glycerol, 0.017 M KH_2PO_4, 0.072 M K_2HPO_4) at 37 °C. *Saccharomyces cerevisiae* FY834 was purchased from ATCC (Manassas, Virginia, USA), and grown in YPD medium (1 % yeast extract, 2 % peptone, 2 % glucose) at 30 °C.

Gene cloning

A. calidoustus CBS121601 was inoculated in 10 mL DPY medium and incubated with shaking at 120 rpm at 30 °C for 3 days. Then, the pre-culture was shaken thoroughly and added into a 500 mL Erlenmeyer flask with 100 mL DPY medium. The culture was incubated at 30 °C with shaking at 120 rpm for 2 days. The mycelium was sapareated and washed with EtOH. The genomic DNA of *A. calidoustus* was isolated and purified by phenol:chloroform method.[17]

The *acAS* gene (accession number: CEN61919.1) was amplified from genomic DNA of *A. calidoustus* CBS121601 by PCR using Q5 polymerase (New England Biolabs, Ipswich, MA, USA) and the primers AcAS-Fw and AcAS-Rv (Table S1). The amplificate was integrated into the vector pArgB-TAA[18] digested with SmaI by ligation using the In-Fusion HD cloning kit (Clontech, Saint-Germain-en-Laye, France) according to the manual provided by the manufacturer to yield pArgB-AcAS. The resulting plasmid was used to transform *E. coli* Stellar that was grown on LB medium with ampicillin (Carl Roth, Karlsruhe, Germany) at 37 °C overnight.

Fungal transformation and products detection

Transformations of *A. oryzae* NSAR1 were performed with the protoplast–polyethylene glycol method, as reported previously.[19] The fungal expression plasmid pArgB-AcAS was transformed into *A. oryzae* NSAR1 to give *A. oryzae* NSAR1-AcAS. The empty vector pArgB-TAA was also transformed into *A. oryzae* NSAR1 to generate *A. oryzae* NSAR1-NC as the negative control strain. Candidate transformants were stabilized in selective agar medium (M agar with methionine and adenine: 0.2 % NH_4Cl, 0.1 % $(NH_4)_2SO_4$, 0.05 % KCl, 0.05 % NaCl, 0.1 % KH_2PO_4, 0.05 % $MgSO_4$, 0.002 % $FeSO_4{\cdot}7H_2O$, 2 % glucose, 0.15 % methionine, 0.01 % adenine, 1.5 % agar, pH=5.5) at 30 °C for 3 days, then inoculated in DPY medium at 30 °C for 3 days. The mycelia were separated and dried, then extracted with acetone under sonication. The extracts were subjected to GC/MS analysis (Figure 2, Figure S3).

Transformant A. oryzae NSAR1-AcAS was inoculated in 10 mL DPY medium and incubated at 30 °C with shaking at 120 rpm for 3 days. The preculture was shaken thoroughtly and then added into a 2 L Erlenmeyer flask for 1 L of CD-starch medium (0.3 % $NaNO_3$, 0.2 % KCl, 1.0 % polypepton, 0.05 % $MgSO_4{\cdot}7H_2O$, 0.1 % KH_2PO_4, 0.002 % $FeSO_4{\cdot}7H_2O$, 0.01 % adenine, 2.0 % starch, pH = 5.5) and incubated at 30 °C with shaking at 120 rpm for 5 days.

The mycelium of *A. oryzae* NSAR1-AcAS was separated and extracted with acetone under sonication. The extract was suspended in deionized water and re-extracted with cyclohexane. After removal of the solvent under reduced pressure, the extract was subjected to column chromatography in silica gel and eluted with cyclohexane. The fractions containing the sesterterpene products were collected and evaporated to give less than 1 mg (<1 mg L^{-1}) in total.

Table S1. Primers used in this study.

Primer name	Nucleotide sequence (5‘ → 3‘)[a]
AcAS-Fw	TCGAGCTCGGTACCCATGGACGCGGCACTGCGT
AcAS-Rv	ACTCTCCACCCTCCCCTACAGCCCCAACCCAACCAACAAC
AcAS-pYE-Fw	AGCATGACTGGTGGAATGGACGCGGCACTGCGTG
AcAS-pYE-Rv	GGTGGTGCTCGAGTGCTACAGCCCCAACCCAACCAACAACATCC
pYE-Fw	CACTCGAGCACCACCACCAC
pYE-Rv	TCCACCAGTCATGCTAGCCATATGG
pYE-AcAS-TC-Fw	GGCAGCCATATGGCTAGCATGACTGGTGGAATGGACGCGGCACTGC
pYE-AcAS-TC-Rv	CTCAGTGGTGGTGGTGGTGGTGCTCGAGTGTTAATCACCGCCACGTCCATTGC
pYE-AcldOS-PT-Fw	GGCAGCCATATGGCTAGCATGACTGGTGGAATCAAGAAACCAGCACATGTTCTCGAG
pYE-AcldOS-PT-Rv	CTCAGTGGTGGTGGTGGTGGTGCTCGAGTGTTAAACCTTCAGCAGCTCCAGCATC
pYE-AcAS-TC-link-Fw	GCATGACTGGTGGAATGGACGCG
pYE-AcAS-TC-link-Rv	ACCGCCGCTACCGCCATCACCGCCACGTCCATTGCTTTG
pYE-link-AcldOS-PT-Fw	GGCGGTAGCGGCGGTGGCTCGGGCGGTGGCAGCGGTATCAAGAAACCAGCACATGTTCTC GAGTACAG
pYE-link-AcldOS-PT-Rv	GGTGGTGCTCGAGTGTTAAACCTTCAGCAG
AcAS-V197S-Fw	CATCGGCTGAGCGAGGGAGGCCCAGAATGGG
AcAS-V197S-Rv	CCTCCCTCGCTCAGCCGATGCTGCTTGAGC
AcAS-G199V-Fw	GGTTGAGGTGGGCCCAGAATGGGCCATTC
AcAS-G199V-Rv	CATTCTGGGCCCACCTCAACCAGCCGATGC
AcAS-G199A-Fw	GGTTGAGGCGGGCCCAGAATGGGCCATTC
AcAS-G199A-Rv	CATTCTGGGCCCGCCTCAACCAGCCGATGC
AcAS-E202R-Fw	GAGGCCCACGCTGGGCCATTCGACTCGGAG
AcAS-E202R-Rv	GTCGAATGGCCCAGCGTGGGCCTCCCTCAAC
AcAS-G232L-Fw	CGATATTGGGCTGATTGCTGGAGTCTTGGCCAACG
AcAS-G232L-Rv	CCAGCAATCAGCCCAATATCGATTATCTCCCGTACTGACTC
AcAS-G232A-Fw	CGATATTGGGGCGATTGCTGGAGTCTTGGCCAACG
AcAS-G232A-Rv	CCAGCAATCGCCCCAATATCGATTATCTCCCGTACTGACTC
AcAS-G235A-Fw	GCATTGCTGCGGTCTTGGCCAACGACTACTACAGC
AcAS-G235A-Rv	GCCAAGACCGCAGCAATGCCCCCAATATCGATTATC
AcAS-G236A-Fw	GCTGGAGCGTTGGCCAACGACTACTACAGCTTC
AcAS-G236A-Rv	CGTTGGCCAACGCTCCAGCAATGCCCCCAATATC
AcAS-A238M-Fw	GGAGTCTTGATGAACGACTACTACAGCTTCAACAAGGAGTTTG
AcAS-A238M-Rv	GTAGTCGTTCATCAAGACTCCAGCAATGCCCCC

[a] Nucleotide exchanges for mutational primers are underlined.

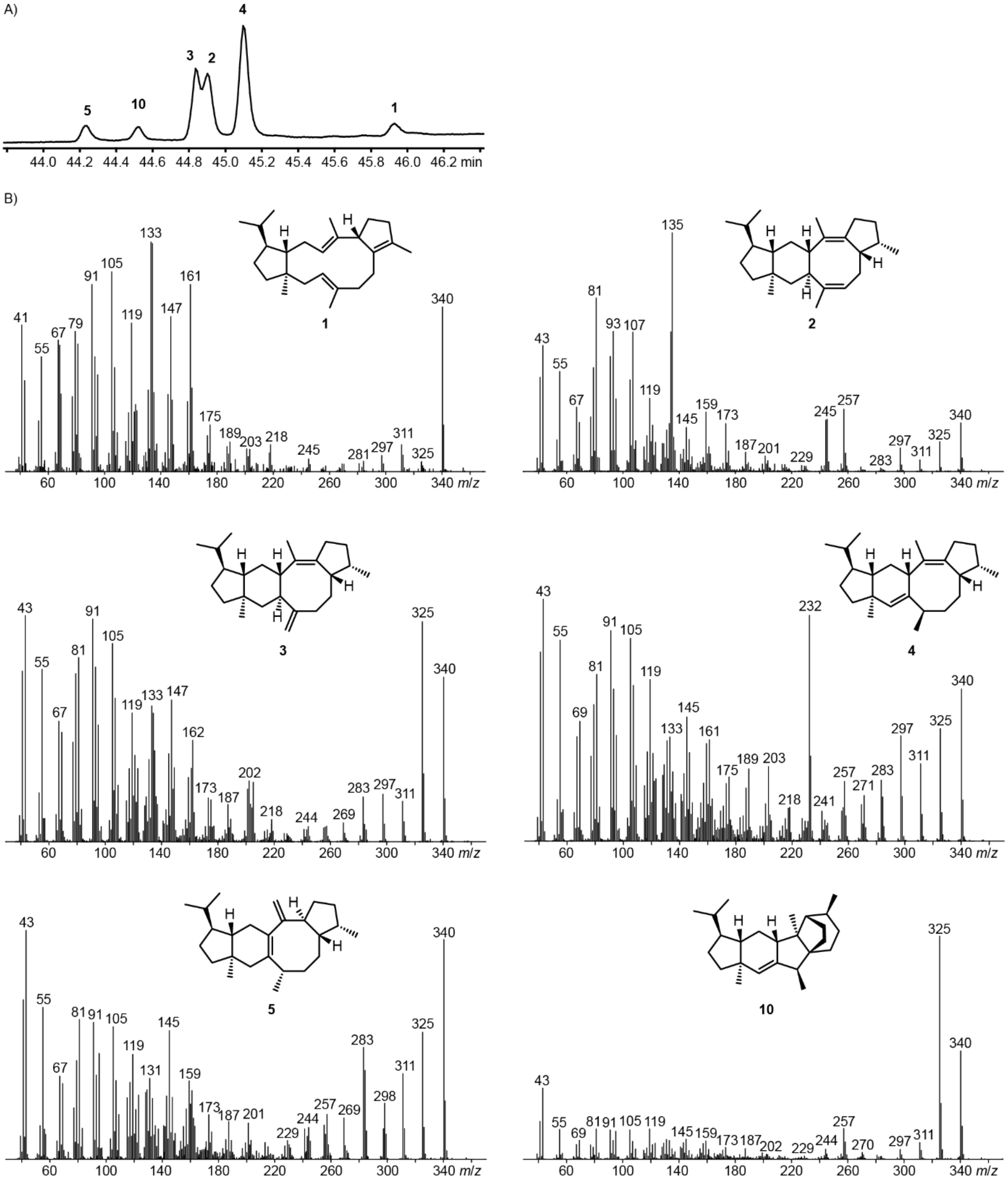

Figure S3. Identification of the AcAS products by GC/MS. A) Total ion chromatogram of an extract of an enzyme incubation of GFPP with AcAS, B) EI mass spectra of compounds **1** – **5** and **10**. Numbers at chromatographic peaks refer to compound numbers.

Extraction of RNA and reverse-transcription of cDNA

The mycelium of *A*. *oryzae* NSAR1-AcAS was obtained from a 100 mL DPY culture that was incubated for 2 days at 30 °C with shaking at 160 rpm. The mycelium was separated, washed with deionised water, 100% ethanol, and deionised water, and then frozen at –80 °C. The frozen mycelium was powdered using a mortar. During this procedure, the temperature was kept low by administration of liquid nitrogen. The RNA was extracted and purified from the powdered mycelium using TRIzol (Sigma Aldrich Chemie GmbH, Steinheim, Germany). cDNA reverse-transcription from purified RNA was carried out using SuperScript III Reverse Transcriptase (Invitrogen, Carlsbad, California). All the procedures during the extraction and reverse-transcription were performed in an environment without RNAse that was deactivated with RNAse AWAY ® (Sigma Aldrich Chemie GmbH, Steinheim, Germany). All the operations were conducted following the manuals provided by manufacturers.

Construction of an *E. coli* expression plasmid for AcAS

Primers used for the plasmid construction for expression of AcAS are listed in Table S1. The DNA fragment encoding AcAS was amplified by PCR using reverse-transcribed cDNA as the template and primers AcAS-pYE-Fw and AcAS-pYE-Rv. The linearised vector pYE-Express was prepared by PCR using empty vector as the template and primers pYE-Fw and pYE-Rv. The DNA fragment encoding AcAS was cloned into linearised pYE-Express to yield pYE-AcAS-cDNA using the In-Fusion HD cloning kit. The constructed plasmid was used to transform *E*. *coli* Stellar. Cells were grown on LB medium with kanamycin (50 μg mL^{-1}) at 37 °C overnight, followed by inoculation of a liquid culture from a single colony. Plasmid DNA was isolated and the cloned gene was verified by sequencing.

```
ATGGACGCGGCACTGCGTGATATCTGCCAGCTCAGCGATCCCTGTGATCCGCGCAGCTTTGAGCCTCCGATCAAGGACTTCTTCTGCATCTA
ATGGACGCGGCACTGCGTGATATCTGCCAGCTCAGCGATCCCTGTGATCCGCGCAGCTTTGAGCCTCCGATCAAGGACTTCTTCTGCATCTA

TCCCATGTACCGCTCTCGCTACGAGGCCAAGGCCATTCAAGGATCAAATGAGTTTCTCGATGGTTGGAACAAAGCGATTGAAAAGGACGGAT
TCCCATGTACCGCTCTCGCTACGAGGCCAAGGCCATTCAAGGATCAAATGAGTTTCTCGATGGTTGGAACAAAGCGATTGAAAAGGACGGAT

TGAGAAACGATGGGCGCCCGTTTCTGGGTTGCAACACAATCTACGGAAACTACGTTGCATGGGCGTATCCAGAGTGTCTTCCAGAGCGGGCA
TGAGAAACGATGGGCGCCCGTTTCTGGGTTGCAACACAATCTACGGAAACTACGTTGCATGGGCGTATCCAGAGTGTCTTCCAGAGCGGGCA

GCACATGTGGCGGCGTACTGCGACTGGGGATTCTTCTGGGACGGTTAGTAGGGTCTTTTCTTCTATAGACAGGCATTGACGAACCTTTGCTT
GCACATGTGGCGGCGTACTGCGACTGGGGATTCTTCTGGGACG-------------------------------------------------

TGCAGATGCTACCGACGCCATGTCGATGGAGAAAAACCACGAGGCCACCAAGGATCTTATCCTTACTATAATGTCAACGGTGGGGATTGGTC
-----ATGCTACCGACGCCATGTCGATGGAGAAAAACCACGAGGCCACCAAGGATCTTATCCTTACTATAATGTCAACGGTGGGGATTGGTC

AGAAGCACGAGCCGCTTCTGGCGGTCAATAAGCTTGTCGTGCCTTTTGTGCTGAACAAGCTCGCTGGAACGGACGGGGATCTTGGGCTAAAC
AGAAGCACGAGCCGCTTCTGGCGGTCAATAAGCTTGTCGTGCCTTTTGTGCTGAACAAGCTCGCTGGAACGGACGGGGATCTTGGGCTAAAC

CACATGAAGGCGTGGAAGGCCCATCTCGATGGCCAGGCCAGGAGCTCACATGCCAACATGTCTTGGGAGGAGCTCAAGCAGCATCGGCTGGT
CACATGAAGGCGTGGAAGGCCCATCTCGATGGCCAGGCCAGGAGCTCACATGCCAACATGTCTTGGGAGGAGCTCAAGCAGCATCGGCTGGT

TGAGGGAGGCCCAGAGTAAGCAAAACAGTCAGCTGCATCCGCTCGCACGCTAATTCTGCCAGATGGGCCATTCGACTCGGAGCGTGGGGCGC
TGAGGGAGGCCCAGA-----------------------------------------------ATGGGCCATTCGACTCGGAGCGTGGGGCGC

CGGGATTCGGTGCACCGCAGAGGAGATTGAGTCAGTACGGGAGATAATCGATATTGGGGGCATTGCTGGAGTCTTGGCCAACGACTACTACA
CGGGATTCGGTGCACCGCAGAGGAGATTGAGTCAGTACGGGAGATAATCGATATTGGGGGCATTGCTGGAGTCTTGGCCAACGACTACTACA

GCTTCAACAAGGAGTTTGATGAGCACTCCCGAGCAGGCACGATAGAGCGGATGCAGAACGGAGTGGCCCTGCTGATGCGGGAATATGGCTAC
GCTTCAACAAGGAGTTTGATGAGCACTCCCGAGCAGGCACGATAGAGCGGATGCAGAACGGAGTGGCCCTGCTGATGCGGGAATATGGCTAC

AGCGAAGAGGAGGCGCGCGAGATCCTGAAAAAGGAGATCAATAAGATGGAGCAGCAGTTCATGGACATGTACCTGACCTGGTTGAACGGCCC
AGCGAAGAGGAGGCGCGCGAGATCCTGAAAAAGGAGATCAATAAGATGGAGCAGCAGTTCATGGACATGTACCTGACCTGGTTGAACGGCCC

TGTTCAAAAGTCTCGCGGCCTGATCCAGTATTTGACCATGGTCCTTTGTCTCTACTCGGGCACAATGTTCTGGATGGCCCACGGCGCGAGGT
TGTTCAAAAGTCTCGCGGCCTGATCCAGTATTTGACCATGGTCCTTTGTCTCTACTCGGGCACAATGTTCTGGATGGCCCACGGCGCGAGGT

ACCACCGCACCGATCTCATTACCACAGCAGAGGATCGGGCTACGATTATTGGGAAGTGCCAGGGGGACGCTTTTCGCGTAATGGAGGGATAT
ACCACCGCACCGATCTCATTACCACAGCAGAGGATCGGGCTACGATTATTGGGAAGTGCCAGGGGGACGCTTTTCGCGTAATGGAGGGATAT

CCTCCGCCAAAGGGGCTGAAGCGGACGGCCAGCTCCCCAGAGTCAGCACCCAAACGGAGGGCTTCAAAAGCGAACAATATCAACCAAAGCAA
CCTCCGCCAAAGGGGCTGAAGCGGACGGCCAGCTCCCCAGAGTCAGCACCCAAACGGAGGGCTTCAAAAGCGAACAATATCAACCAAAGCAA

TGGACGTGGCGGTGATCCCATGGTCGCCTTTTCAGGTCCCTTCGTGAAGGCTCCAAGCCATGTACGTGTCGCTCCTGGGCTGTATTTGTGCA
TGGACGTGGCGGTGATCCCATGGTCGCCTTTTCAGGTCCCTTCGTGAAGGCTCCAAGCCAT-------------------------------

CTGACCTGACCGAGATAGATCTGCGATGCTCCGTACGAGTACATCGACTCTCTCCAATCCAAGAACATGCGTGACAAGTTCATCAACATCCT
------------------ATCTGCGATGCTCCGTACGAGTACATCGACTCTCTCCAATCCAAGAACATGCGTGACAAGTTCATCAACATCCT

CAACTCCTGGCTGAACGTGCCGTCCGACTCGCTGCAAATCATCAAAAACATTGTCCAGATGTTGCACAACTCATCATTAATGTACGCCCCTT
CAACTCCTGGCTGAACGTGCCGTCCGACTCGCTGCAAATCATCAAAAACATTGTCCAGATGTTGCACAACTCATCATTAAT-----------

GTATGCGCTGAGTTGTTCTAGCCCTAATTTTCCAGGCTTGACGACATTGAAGACGCCTCTCCTCTCCGTCGAGGCCAACCGGCAACCCACAT
-----------------------------------GCTTGACGACATTGAAGACGCCTCTCCTCTCCGTCGAGGCCAACCGGCAACCCACAT

TTTCTACGGCGCCAGCCAGACCATCAACAGCGCCAACTTTAGCTACGTCAAGACGGTCATCGAGGCCACTCACCTTAAGAACCCGCAATGTC
TTTCTACGGCGCCAGCCAGACCATCAACAGCGCCAACTTTAGCTACGTCAAGACGGTCATCGAGGCCACTCACCTTAAGAACCCGCAATGTC

TGCAAATCTTCCTCGAGGAAGTCAGCGACCTCCACCGCGGTCAGAGCCTCGACCTGCACTGGCGCCACCACGGCCGTTGCCCGACGACAGAC
TGCAAATCTTCCTCGAGGAAGTCAGCGACCTCCACCGCGGTCAGAGCCTCGACCTGCACTGGCGCCACCACGGCCGTTGCCCGACGACAGAC

GAGTACATTATGATGGTCGACAACAAGACTGGCGGGCTCTTCCGTCTGATGGCCCGTCTGATGGAAGCCGAATCGCCCTCTCCCATAACGAT
GAGTACATTATGATGGTCGACAACAAGACTGGCGGGCTCTTCCGTCTGATGGCCCGTCTGATGGAAGCCGAATCGCCCTCTCCCATAACGAT

TCCCCATCTCAGCCGCCTCCTCACCCTGATAGGTCGCTACTACCAAATCCGAGATGACTATATGAATCTTACTTCAGCTGATGTCAGTCCTC
TCCCCATCTCAGCCGCCTCCTCACCCTGATAGGTCGCTACTACCAAATCCGAGATGACTATATGAATCTTACTTCAGCTGAT----------

CTGTATTTTCTTTTCCCAATATTTACTAACAATAGCCAAGTATACCACAAAGAAGGGCTATTGCGAAGACCTCGACGAGGGCAAATTCTCGC
----------------------------------------TATACCACAAAGAAGGGCTATTGCGAAGACCTCGACGAGGGCAAATTCTCGC

TCCCCCTCATCCACCTCCTCCTCCACACCTCGTGCCCGGACCGGATCACTTCCGCTCTATACAACCGCGTCCCATCGACAGGTCTGCAGGAC
TCCCCCTCATCCACCTCCTCCTCCACACCTCGTGCCCGGACCGGATCACTTCCGCTCTATACAACCGCGTCCCATCGACAGGTCTGCAGGAC

GAGGTCAAGACGTATATCCTGGACGCTATGCAGTCTGCGCGTACATTTGAATACGTTCGTGAGGTGCTGTCGCATTTGCACGGGGAGATTAT
GAGGTCAAGACGTATATCCTGGACGCTATGCAGTCTGCGCGTACATTTGAATACGTTCGTGAGGTGCTGTCGCATTTGCACGGGGAGATTAT

GAAGACGCTGGATGAGGCTGAGAAGACGTTGGGGATTAACAATGGGGTTCGGATGTTGTTGGTTGGGTTGGGGCTGTAG
GAAGACGCTGGATGAGGCTGAGAAGACGTTGGGGATTAACAATGGGGTTCGGATGTTGTTGGTTGGGTTGGGGCTGTAG
```

Figure S4. Alignment of gDNA (top) and cDNA of *acAS* (bottom). Intron sequences are highlighted in yellow.

Protein expression and purification

E. *coli* BL21 (DE3) was transformed with plasmid pYE-AcAS-cDNA for protein expression. A preculture of the transformant was cultivated in TB medium (2.4 % yeast extract, 2.0 % tryptone, 0.4 % glycerol, 0.017 M KH_2PO_4, 0.072 M K_2HPO_4). For this medium, 900 mL nutrient solution containing all other components except phosphates and 100 mL phosphate buffer are prepared and sterilised separately. After cooling to room temperature, the phosphate buffer is added to the nutrient solution with kanamycin (50 μg mL^{-1}). The preculture (20 mL) was incubated at 37 °C overnight with shaking at 160 rpm and then used to inoculate an expression culture (1 L) in TB medium with kanamycin (50 μg mL^{-1}). The culture was grown at 37 °C for ca. 4 h until an OD_{600} of 0.4 – 0.6 was reached and then cooled to 18 °C. IPTG solution (400 mM, 1 mL) was added to induce protein expression. The expression was carried out at 18 °C with shaking at 160 rpm for 20 h.

The culture was centrifuged (3,600 x g, 40 min) to saparete the cells from the medium. The supernatant was discarded and the cell pellet was resuspended in binding buffer (20 mL; 20 mM Na_2HPO_4, 500 mM NaCl, 20 mM imidazole, 1 mM $MgCl_2$, pH = 7.4, 4 °C). Cell lysis was carried out through ultra sonication (6 x 1 min). The resulting suspension was centrifuged (14,610 x g, 2 x 10 min) to remove cell debris. The supernatant was filtered and transferred to a Ni^{2+}-NTA affinity column (5 mL resin volume; Super Ni-NTA, Generon, Slough, UK). Undesired proteins were eluted with binding buffer (2 x 10 mL) and washing buffer (2 x 10 mL; 20 mM Na_2HPO_4, 500 mM NaCl, 50 mM imidazole, 1 mM $MgCl_2$, pH = 7.4, 4 °C). The desired protein was eluted with elution buffer (2 x 10 mL; 20 mM Na_2HPO_4, 500 mM NaCl, 500 mM imidazole, 1 mM $MgCl_2$, pH = 7.4, 4 °C). The protein purity was checked by SDS-PAGE and the concentration was determined by Bradford assay (yield: 20 mL protein preparation, 0.2 mg mL^{-1}).[20]

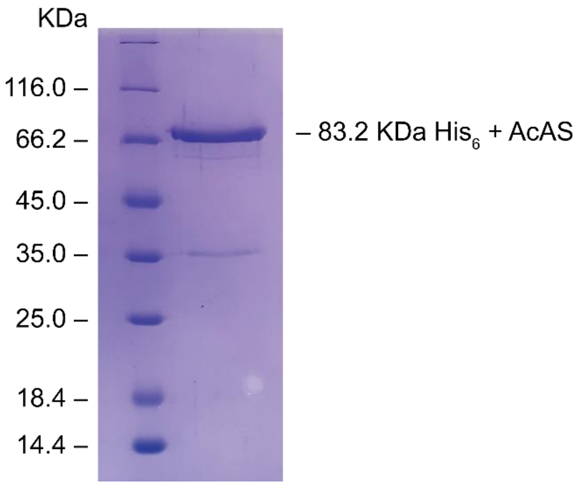

Figure S5. SDS-PAGE analysis of purified AcAS.

Small scale reactions with AcAS (substrate scope)

Enzymatic test reactions were performed using freshly prepared AcAS and different combinations of substrates dissolved in 25 mM NH_4HCO_3 (10 mg mL^{-1}). The enzyme solution obtained from a Ni^{2+}-NTA affinity chromatography was concentrated by ultrafiltration to >2 mg mL^{-1}, then diluted with incubation buffer (50 mM TRIS, 10 mM $MgCl_2$, 20% glycerol, pH 8.2, final enzyme concentration 0.2 mg mL^{-1}). To 1 mL of this enzyme solution 0.5 mg of each substrate (GFPP, GGPP + IPP, FPP + IPP, GPP + IPP, and DMAPP + IPP) was added. The reaction mixture was incubated at 30 °C overnight, extracted with benzene or hexane for GC/MS analysis or compound isolation. The results are shown in Figure S6.

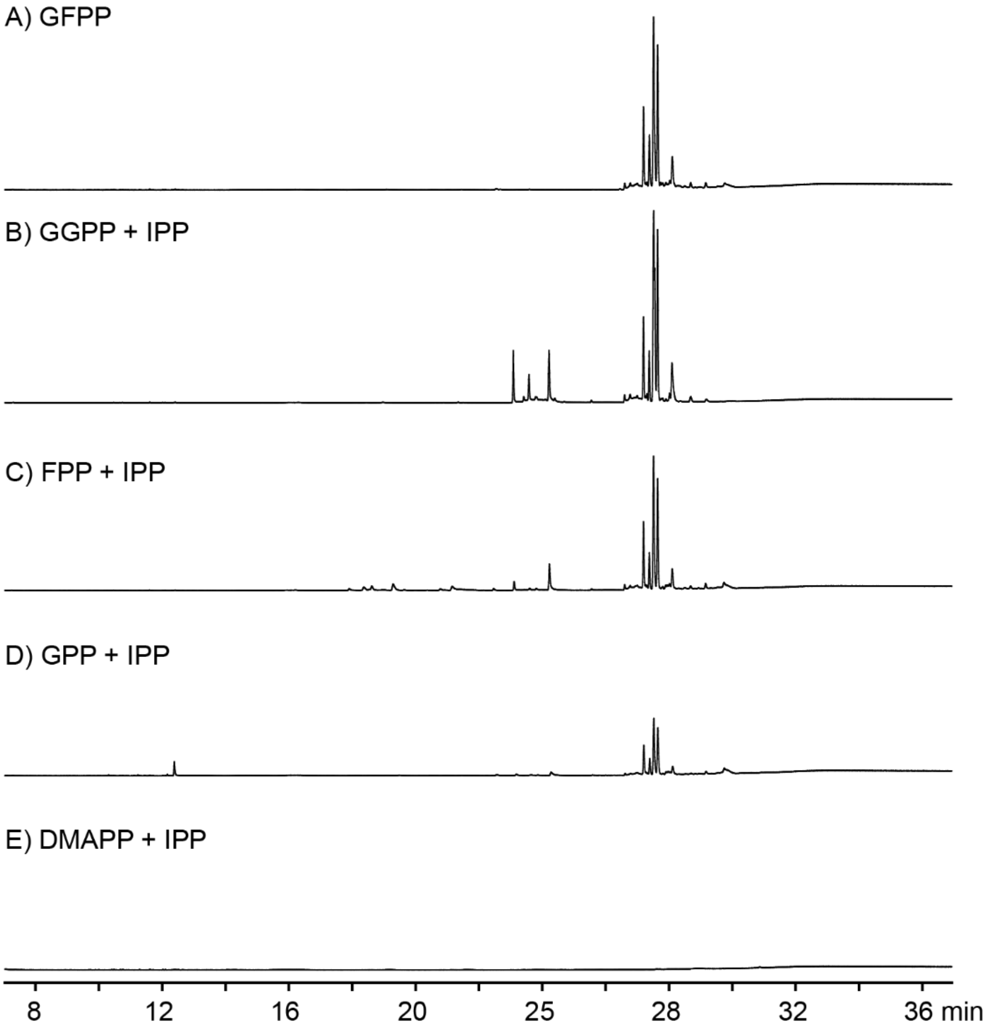

Figure S6. Total ion chromatograms of extracts from enzyme incubations of AcAS with A) GFPP, B) GGPP + IPP, C) FPP + IPP, D) GPP + IPP, and E) DMAPP + IPP. The peaks between 20 min and 25 min in B) are spontaneous hydrolysis products of GGPP.

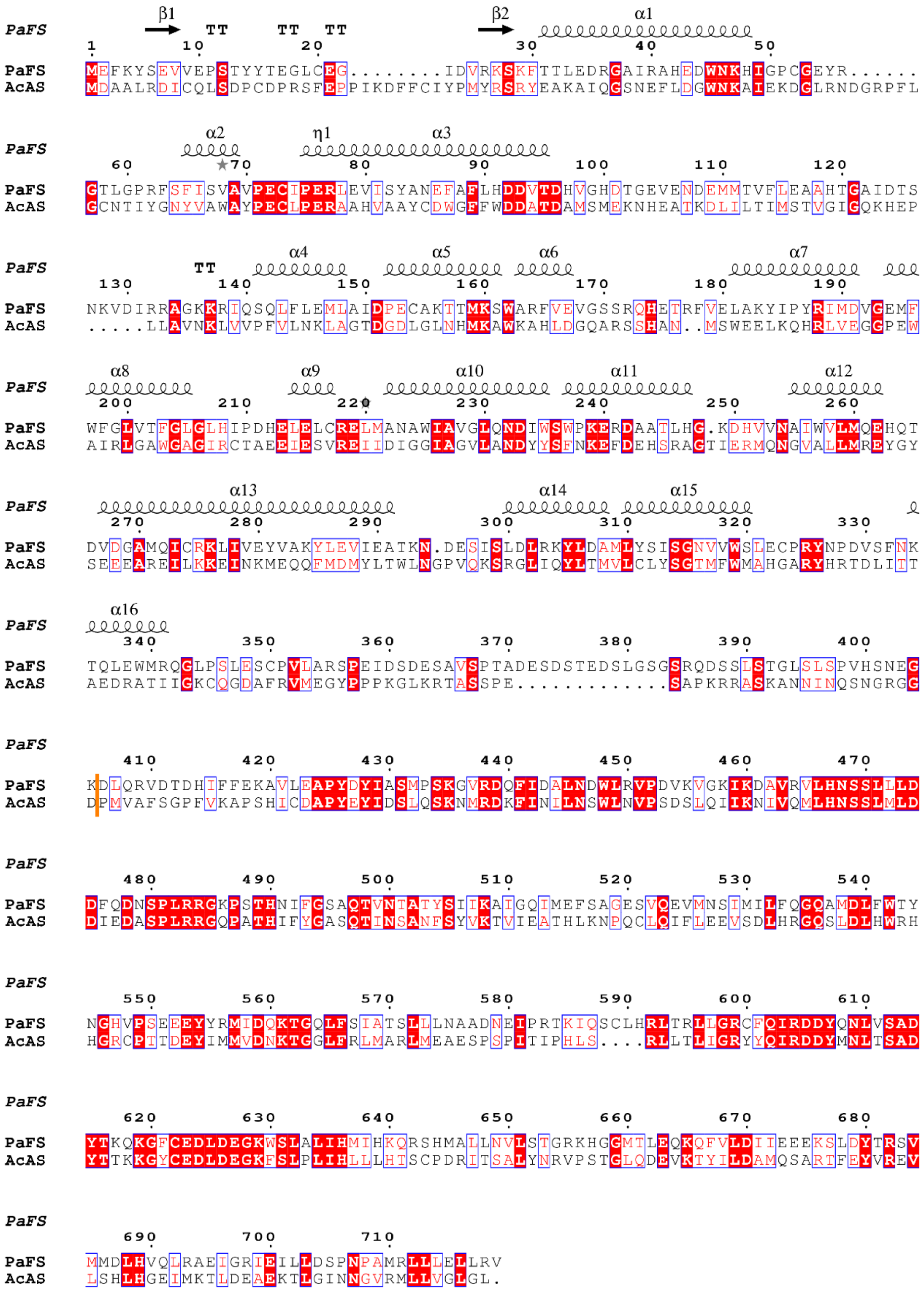

Figure S7. Comparison of secondary structures of PaFS (top) and AcAS (bottom) using ESPript 3.0.[21] The orange bar indicates the truncation site for cloning of the gene sequence of the N-terminal AcAS-TC domain.

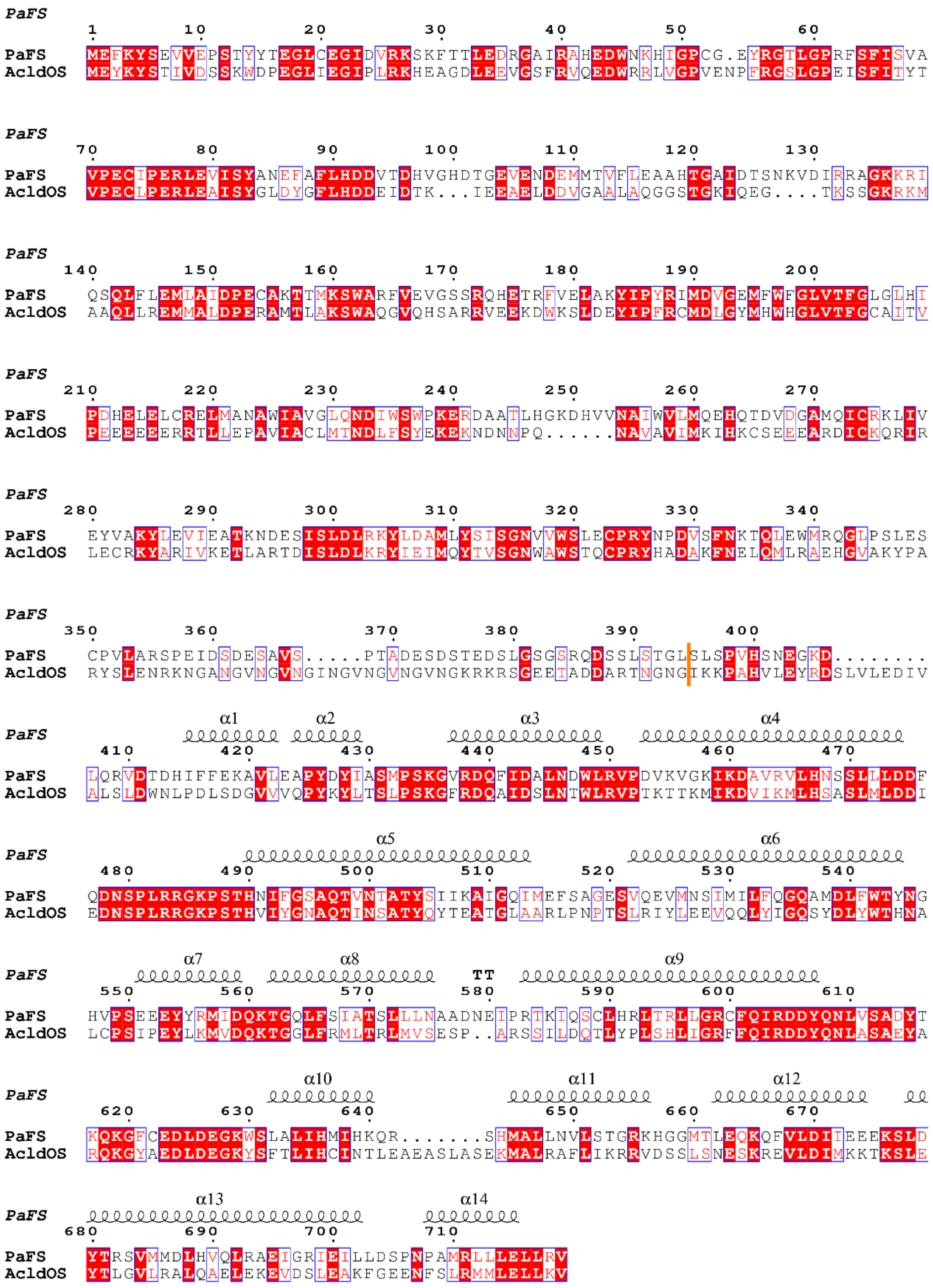

Figure S8. Comparison of secondary structures of PaFS (top) and AcldOS (bottom) using ESPript 3.0.[21] The orange bar indicates the truncation site for cloning of the gene sequence of the C-terminal AcldOS-PT domain.

Construction of *E. coli* expression plasmids for truncated and fusion proteins

Primers used for the plasmids construction for truncated enzymes (AcAS-TC and AcIdOS-PT) and the fusion protein (AcAS-TC-AcIdOS-PT) are listed in Table S1.

For the truncated proteins, the DNA fragment encoding AcAS-TC (N-terminal 401 aa) was amplified by PCR using pYE-AcAS-cDNA as the template and primers pYE-AcAS-TC-Fw and pYE-AcAS-TC-Rv. The DNA fragment encoding AcIdOS-GFPPS (C-terminal 388 aa) was amplified by PCR using pYE-AcIdOS[8] as the template and primers pYE-AcIdOS-PT-Fw and AcIdOS-PT-Rv. The linearised vector pYE-Express was prepared by restriction enzyme digestion using BamHI and HindIII. The DNA fragment encoding AcAS-TC was cloned into linearised pYE-Express to yield pYE-AcAS-TC using homologous recombination in *S. cerevisiae* FY834 through the PEG/LiOAc method.[22] With the same method, the DNA fragment encoding AcIdOS-PT was cloned into linearised pYE-express to yield pYE-AcIdOS-GFPPS. After culturing the yeast on selective agar medium (SM–ura: 0.17% yeast nitrogen base, 0.50% $(NH_4)_2SO_4$, 2.0% glucose, 0.077% nutritional supplement minus uracil (Sigma Aldrich Chemie GmbH, Steinheim, Germany), 2.4% agar) for three days, the plasmid integrated with DNA fragment was isolated from the yeast by using the NucleoSpin Plasmid (NoLid) kit (Macherey-Nagel GmbH & Co. KG, Düren, Germany), followed by transformation of *E. coli* BL21 (DE3). *E. coli* BL21 (DE3) was grown on selective LB medium with kanamycin (50 μg mL^{-1}) at 37 °C overnight. A single colony was selected to grow a liquid culture, from which plasmid DNA was isolated and verified by sequencing.

For the fusion protein, the DNA fragment encoding AcAS-TC was amplified by PCR using pYE-AcAS-TC as the template and primers pYE-AcAS-TC-link-Fw and pYE-AcAS-TC-link-Rv, and the DNA fragment encoding AcIdOS-GFPPS was amplified by PCR using pYE-AcIdOS-PT as template and primers pYE-link-AcIdOS-PT-Fw and pYE-link-AcIdOS-PT-Rv. The linearised vector pYE-Express was prepared by PCR using empty vector as the template and primers pYE-Fw and pYE-Rv. The DNA fragments encoding AcAS-TC and AcIdOS-GFPPS were cloned into linearised pYE-express to yield pYE-AcAS-TC-AcIdOS-PT using In-Fusion HD cloning kit. The constructed plasmid was used to transform *E. coli* Stellar. Cells were grown on selective LB medium with kanamycin (50 μg mL^{-1}) at 37 °C overnight. A single colony was selected to grow a liquid culture, from which plasmid DNA was isolated and verified by sequencing.

Protein expression and enzymatic reactions

E. coli BL21 (DE3) transformed with plasmid pYE-AcAS-TC, pYE-AcIdOS-PT or pYE-AcAS-TC-AcIdOS-PT for protein expression was pre-cultivated in TB medium with kanamycin (50 μg mL^{-1}) at 37 °C overnight with shaking at 160 rpm. The preculture was inoculated to a larger culture of TB medium (20 mL L^{-1}) with kanamycin (50 μg mL^{-1}). The culture was grown at 37 °C for ca. 4 h until an OD_{600} of 0.4 – 0.6 was reached. The culture was cooled to 18 °C, and IPTG solution (400 mM, 1 mL L^{-1}) was added to induce protein expression. The expression was carried out at 18 °C with shaking at 160 rpm overnight.

The culture was centrifuged (3,600 x g, 40 min) to separate the cells from the medium. The supernatant was discarded and the cell pellet was resuspended in binding buffer. Cell lysis was carried out using ultra sonication (6 x 1 min), and the resulting suspension was centrifuged (14,610 x g, 2 x 10 min) to remove the cell debris. The supernatant was filtrated and transferred to a Ni^{2+}-NTA affinity column. Undesired protein was washed off the column with binding buffer (2 x 10 mL L^{-1} culture) and washing buffer (2 x 10 mL L^{-1} culture), the desired His6-tagged protein, AcAS-TC, AcIdOS-PT or AcAS-TC-AcIdOS-PT, was eluted with elution buffer (2 x 10 mL L^{-1}). Protein purity was confirmed by SDS-PAGE (Figures S9 and S10) and protein concentrations were determined by Bradford assay (AcAS-TC: 4.0 mg mL^{-1}, AcIdOS-PT: 2.0 mg mL^{-1}, AcAS-TC-AcIdOS-PT: 2.5 mg mL^{-1}.

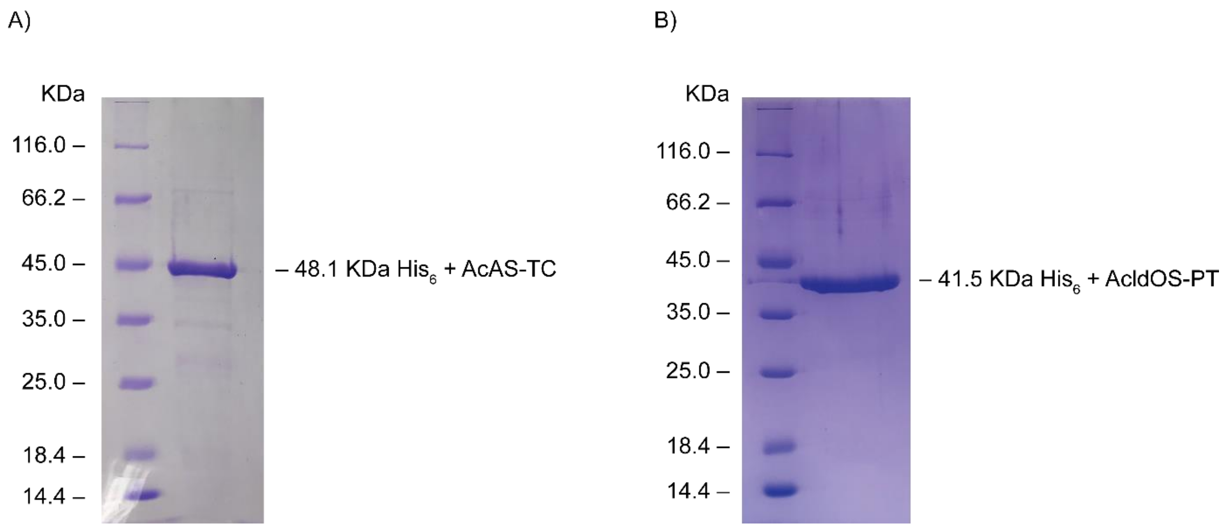

Figure S9. SDS-PAGE analysis of A) AcAS-TC and B) AcldOS-PT both with N-terminal His_6-tags.

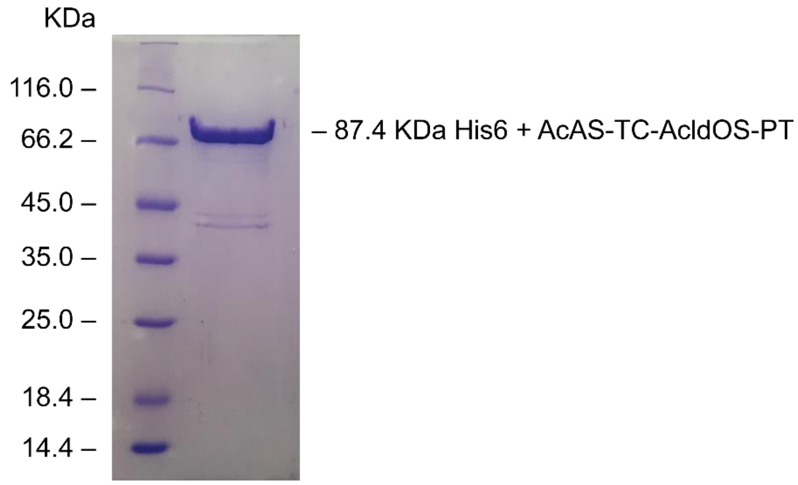

Figure S10. SDS-PAGE analysis of AcAS-TC-AcldOS-PT with N-terminal His_6-tag.

Small scale reactions with truncated enzymes (AcAS-TC and AcIdOS-PT) and fusion protein (AcAS-TC-AcIdOS-PT)

The reaction catalysed by AcAS-TC was performed using freshly prepared AcAS-TC enzyme and GFPP (0.5 mg) dissolved in 25 mM NH_4HCO_3 buffer (50 μL). An enzyme preparation of AcAS-TC (80 μL, 5 mg mL^{-1}) and incubation buffer (870 μL) were added. The reaction mixture was incubated at 30 °C overnight and then extracted with benzene for GC/MS analysis.

For the reactions with the truncated enzymes AcAS-TC and AcIdOS-PT or the fusion protein AcAS-TC-AcIdOS-PT, DMAPP (0.5 mg) and IPP (0.5 mg) were dissolved in 25 mM NH_4HCO_3 buffer (100 μL). Enzyme preparations of AcAS-TC (80 μL, 5 mg mL^{-1}) and AcIdOS-PT (100 μL, 2 mg mL^{-1}) or of AcAS-TC-AcIdOS-PT (100 μL, 2.5 mg mL^{-1}) were added. Incubation buffer was added to a final volume of 1 mL. The reaction mixture was incubated at 30 °C overnight and then extracted with benzene for GC/MS analysis.

The results are shown in Figure 2 of main text.

Preparative scale enzyme reactions with AcAS

FPP trisammonium salt (400 mg, 0.92 mmol) and IPP trisammonium salt (400 mg, 1.35 mmol) were each dissolved in 25 mM NH_4HCO_3 (40 mL). For the following enzyme reactions enzyme preparation from 40 L of expression culture was used. A total number of 800 small scale reactions, each containing incubation buffer (800 μL; 50 mM TRIS, 10 mM $MgCl_2$, 20% glycerol, pH = 8.2), AcAS enzyme preparation concentrated by ultrafiltration (100 μL, 2 mg mL^{-1} in elution buffer), FPP solution (50 μL) and IPP solution (50 μL), were incubated at 30 °C overnight. The reaction mixtures were combined and extracted with hexane for two times. The combined organic layers were washed with brine, dried with Na_2SO_4, and concentrated under reduced pressure. The residue was subjected to silica gel chromatography using hexane for compound elution. The mixture of six sesterterpenes was collected and separated by semi-preparative HPLC on a Nucleodur 110-5 Gravity C18 column (5 μm, 250 x 16 mm) using acetonitrile (100% isocratic, 20 mL min^{-1}, 106 bar) for elution of the pure compounds.

Aspergiltriene (1). TLC (cyclohexane [100 %]): R_f = 0.99. $[\alpha]_D^{25}$ = –25.0 (*c* 0.02, cyclohexnae), HRMS (EI): $[M]^+$ calcd. for $C_{25}H_{40}^+$ *m*/*z* 340.3125; found *m*/*z* 340.3127. GC (HP5-MS): *I* = 2485. MS (EI, 70 eV): *m*/*z* (%) = 340 (41), 325 (5), 311 (5), 297 (3), 283 (1), 269 (2), 255 (2), 245 (4), 232 (3), 217 (10), 210 (9), 189 (10), 175 (17), 161 (77), 147 (66), 134 (100), 133 (100), 119 (50), 105 (76), 91 (70), 81 (57), 67 (39), 55 (46), 41 (42). IR (diamond ATR): $\tilde{\nu}$ / cm^{-1} = 2954 (s), 2925 (s), 2854 (m), 1740 (m), 1453 (m), 1377 (m), 1260 (m), 1093 (m), 1020 (m), 799 (m). For NMR data cf. Table S2.

Aspergildiene A (2). TLC (cyclohexane [100 %]): R_f = 0.99. $[\alpha]_D^{25}$ = –63.1 (*c* 0.16, cyclohexane), HRMS (EI): $[M]^+$ calcd. for $C_{25}H_{40}^+$ *m*/*z* 340.3125; found *m*/*z* 340.3125. GC (HP5-MS): *I* = 2419. MS (EI, 70 eV): *m*/*z* (%) = 340 (17), 325 (6), 311 (3), 297 (9), 283 (1), 271 (1), 257 (20), 245 (21), 229 (4), 215 (2), 201 (6), 187 (5), 173 (17), 159 (22), 147 (14), 135 (100), 119 (24), 107 (51), 93 (54), 81 (50), 67 (21), 55 (25), 41 (21). IR (diamond ATR): $\tilde{\nu}$ / cm^{-1} =.2956 (s), 2927 (s), 2872 (m), 1738 (m), 1462 (m), 1375 (m), 1260 (m), 1203 (m), 1095 (m), 1031 (m), 803 (m), 542 (w), 817 (w). For NMR data cf. Table S3.

Aspergildiene B (3). TLC (cyclohexane [100 %]): R_f = 0.99. $[\alpha]_D^{25}$ = –11.4 (*c* 0.33, cyclohexane), HRMS (EI): $[M]^+$ calcd. for $C_{25}H_{40}^+$ *m*/*z* 340.3125; found *m*/*z* 340.3127. GC (HP5-MS): *I* = 2414. MS (EI, 70 eV): *m*/*z* (%) = 340(41), 325 (66), 311 (7), 297 (15), 283 (13), 269 (3), 256 (5), 241 (4), 229 (3), 218 (14), 205 (29), 187 (16), 173 (22), 162 (53), 147 (67), 134 (74), 119 (64), 105 (94), 91(97), 81 (100), 67 (47), 55 (79), 41 (66). IR (diamond ATR): $\tilde{\nu}$ / cm^{-1} = 3063 (w), 2951 (s), 2927 (s), 2870 (s), 1738 (m), 1678 (w), 1666 (w), 1636 (w), 1460 (m), 1377 (m), 1260 (s), 1092 (s), 1024 (s), 882 (m), 799 (s), 701 (w), 669 (w), 494 (w). For NMR data cf. Table S4.

Aspergildiene C (4). TLC (cyclohexane [100 %]): R_f = 0.99. $[\alpha]_D^{25}$ = –80.9 (*c* 0.15, cyclohexane), HRMS (EI): $[M]^+$ calcd. for $C_{25}H_{40}^+$ *m*/*z* 340.3125; found *m*/*z* 340.3124. GC (HP5-MS): *I* = 2429. MS (EI, 70 eV): *m*/*z* (%) = 340 (56), 325 (40), 311 (32), 297 (40), 283 (22), 271 (17), 257 (24) 241 (12), 232 (100) 218 (14), 203 (34), 189 (29), 175 (30), 161 (45), 145 (49),

133 (45), 119 (64), 105 (77), 91 (76), 81 (68), 69 (45), 55 (66), 43 (55). IR (diamond ATR): $\tilde{\nu}$ / cm^{-1} = 2958 (s), 2927 (s), 2856 (m), 2358 (m), 1728 (s), 1451 (m), 1366 (m), 1260 (s), 1229 (m), 1098 (s), 1027 (s), 799 (s). For NMR data cf. Table S5.

Aspergildiene D (5). TLC (cyclohexane [100 %]): R_f = 0.99. $[\alpha]_D^{25}$ = –33.6 (*c* 0.17, cyclohexane), HRMS (EI): $[M]^+$ calcd. for $C_{25}H_{40}^+$ *m*/*z* 340.3125; found *m*/*z* 340.3124. GC (HP5-MS): *I* = 2376. MS (EI, 70 eV): *m*/*z* (%) = 340 (74), 325 (42), 311 (25), 298 (21), 283 (41), 269 (13), 257 (15), 244 (12), 230 (7), 213 (5), 210 (14), 187 (14), 173 (18), 159 (30), 145 (49), 131 (28), 119 (41), 105 (51), 95 (55), 81 (74), 72 (74), 59 (100), 41 (78). IR (diamond ATR): $\tilde{\nu}$ / cm^{-1} = 3078 (w), 2953 (s), 2928 (s), 2871 (s), 1621 (w), 1455 (m), 1376 (m), 1260 (w), 1084 (w), 1017 (w), 890 (m), 802 (w), 543 (w). For NMR data cf. Table S6.

Calidoustene (10). TLC (cyclohexane [100 %]): R_f = 0.99. $[\alpha]_D^{25}$ = –66.4 (*c* 0.05, cyclohexane), HRMS (EI): $[M]^+$ calcd. for $C_{25}H_{40}^+$ *m*/*z* 340.3125; found *m*/*z* 340.3126. GC (HP5-MS): *I* = 2398. MS (EI, 70 eV): *m*/*z* (%) = 340 (47), 325 (100), 311 (11), 297 (9), 283 (2), 269 (3), 257 (8), 244 (4), 230 (4), 215 (5), 203 (7), 187 (9), 173 (7), 161 (10), 145 (14), 133 (17), 119 (23), 105 (28), 91 (27), 81 (25), 69 (17), 55 (24), 43 (24). IR (diamond ATR): $\tilde{\nu}$ / cm^{-1} = 2953 (s), 2925 (s), 2869 (s), 2365 (m), 2330 (m), 2050 (w), 1952 (w), 1453 (m), 1356 (m), 1261 (m), 1090 (m), 1005 (m), 798 (m). For NMR data cf. Table S7.

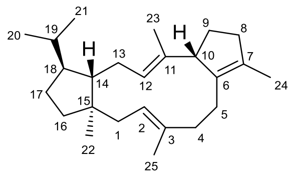

1

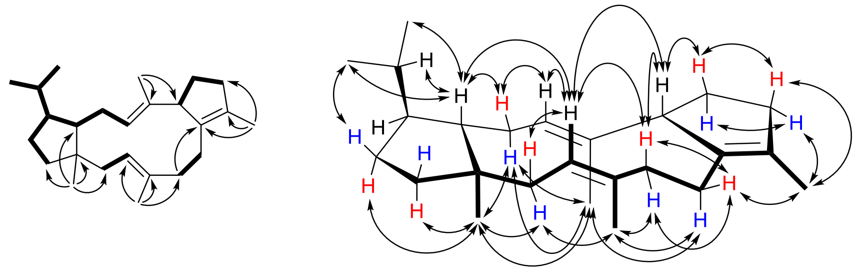

Figure S11. Structure elucidation of **1**. Bold: ^{1}H,^{1}H-COSY correlations, single-headed arrows: key HMBC correlations, and double headed arrows: NOESY correlations.

Table S2. NMR data of aspergiltriene A (**1**) in C_6D_6 recorded at 298 K.

C[a]		δ_C[b]	δ_H[b]	δ_C[c]	δ_H[c]
1	CH_2	42.9	2.03 (m, 1H, H_β)<br>2.11 (m, 1H, H_α)	42.5	1.93 (dd, J = 14.9, 7.3, 1H)<br>1.99 (dd, J = 14.9, 9.1, 1H)
2	CH	123.5	5.41 (t, $^3J_{H,H}$ = 7.8, 1H)	123.1	5.27 (dd, J = 9.1, 7.3, 1H)
3	C_q	135.5	–	135.6	–
4	CH_2	37.1	1.98 (m, 1H, H_β)<br>2.21 (m, 1H, H_α)	36.8	1.86 (ddd, J = 15.2, 9.3, 4.6, 1H)<br>2.11 (ddd, J = 15.2, 9.3, 4.0, 1H)
5	CH_2	26.1	1.83 (m, 1H, H_α)<br>2.52 (m, 1H, H_β)	25.8	1.70 (m, 1H)<br>2.45 (ddd, J = 14.0, 9.6, 4.0, 1H)
6	C_q	139.3	–	138.9	–
7	C_q	132.7	–	132.6	–
8	CH	38.1	2.26 (m, 1H, H_β)<br>2.30 (m, 1H, H_α)	37.9	2.24 (m, 2H)
9	CH_2	28.0	1.72 (m, 1H, H_α)<br>2.00 (m, 1H, H_β)	27.6	1.56 (m, 1H)<br>1.88 (m, 1H)
10	CH	58.3	3.46 (t, $^3J_{H,H}$ = 7.0, 1H)	57.9	3.33 (t, J = 6.4, 1H)
11	C_q	134.0	–	134.2	–
12	CH	129.8	5.18 (m, 1H)	129.6	5.08 (dd, J = 9.0, 4.9, 1H)
13	CH_2	29.7	2.01 (m, 2H)	29.3	1.95 (m, 2H)
14	CH	51.4	1.47 (m, 1H)	51.0	1.40 (m, 1H)
15	C_q	45.3	–	45.4	–
16	CH_2	44.3	1.39 (m, 1H, H_α)<br>1.53 (m, 1H, H_β)	44.0	1.37 (m, 1H)<br>1.48 (m, 1H)
17	CH_2	23.8	1.28 (m, 1H, H_β)<br>1.53 (m, 1H, H_α)	23.5	1.24 (m, 1H)<br>1.50 (m, 1H)
18	CH	53.8	1.42 (m, 1H)	53.7	1.44 (m, 1H)
19	CH	29.2	1.70 (m, 1H)	28.9	1.73 (m, 1H)
20	CH_3	22.8	0.96 (d, $^3J_{H,H}$ = 6.9, 3H)	22.8	0.93 (d, J = 6.8, 3H)
21	CH_3	17.2	0.87 (d, $^3J_{H,H}$ = 6.8, 3H)	17.0	0.82 (d, J = 6.8, 3H)
22	CH_3	22.4	0.91 (s, 3H)	22.2	0.85 (s, 3H)
23	CH_3	12.5	1.52 (s, 3H)	12.3	1.39 (s, 3H)
24	CH_3	14.3	1.64 (s, 3H)	14.3	1.64 (s, 3H)
25	CH_3	17.0	1.61 (s, 3H)	17.0	1.58 (s, 3H)

[a] Carbon numbering indicating the origin for each carbon from GFPP by same number and colour code for diastereotopic hydrogens as shown in Figure S11. [b] Chemical shifts δ in ppm, multiplicity: s = singlet, d = doublet, t = triplet, m = multiplet, coupling constants J are given in Hertz. [c] Chemical shifts δ in ppm reported previously (recorded in $CDCl_3$).[12]

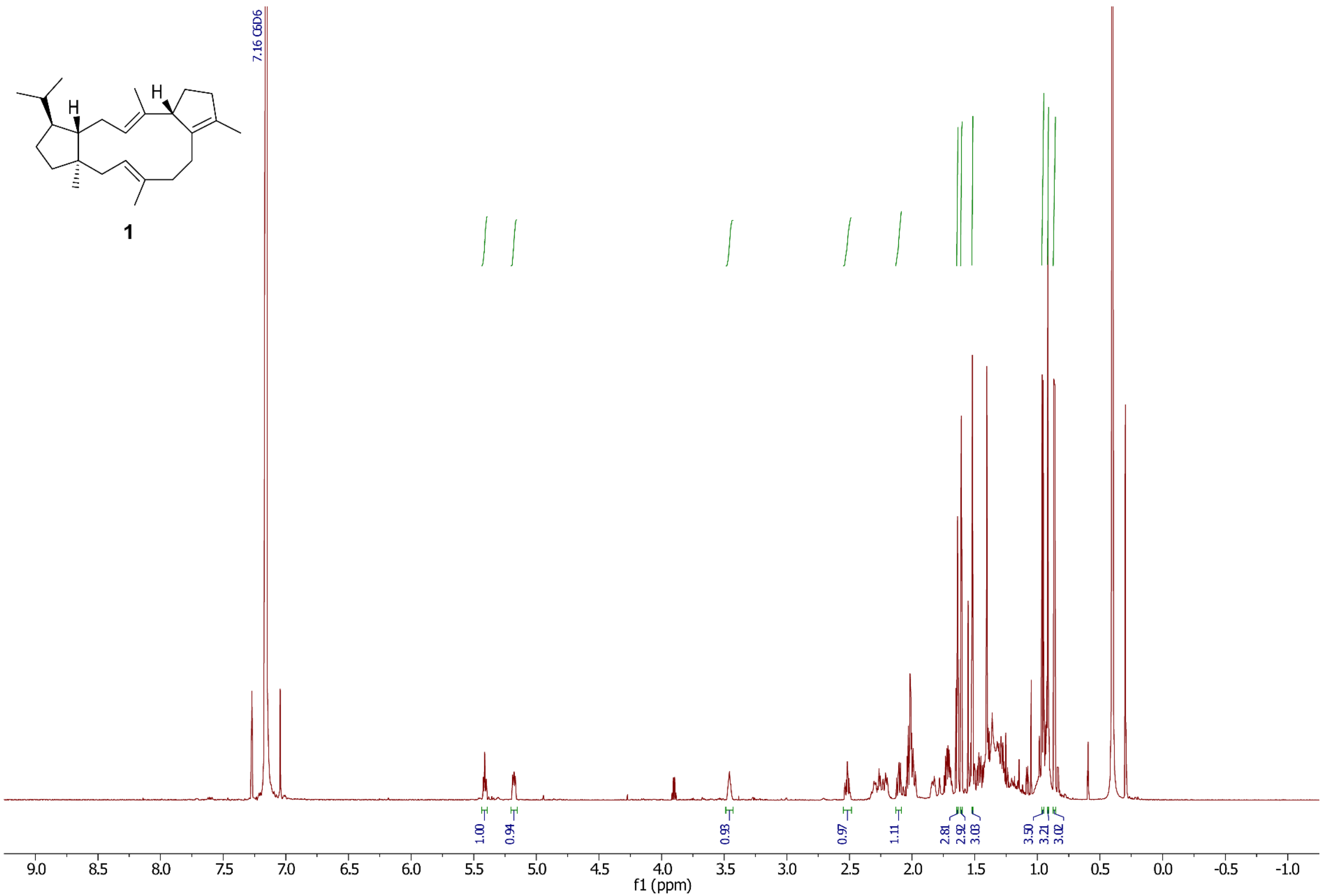

Figure S12. ^{1}H-NMR spectrum of **1** (700 MHz, C_6D_6).

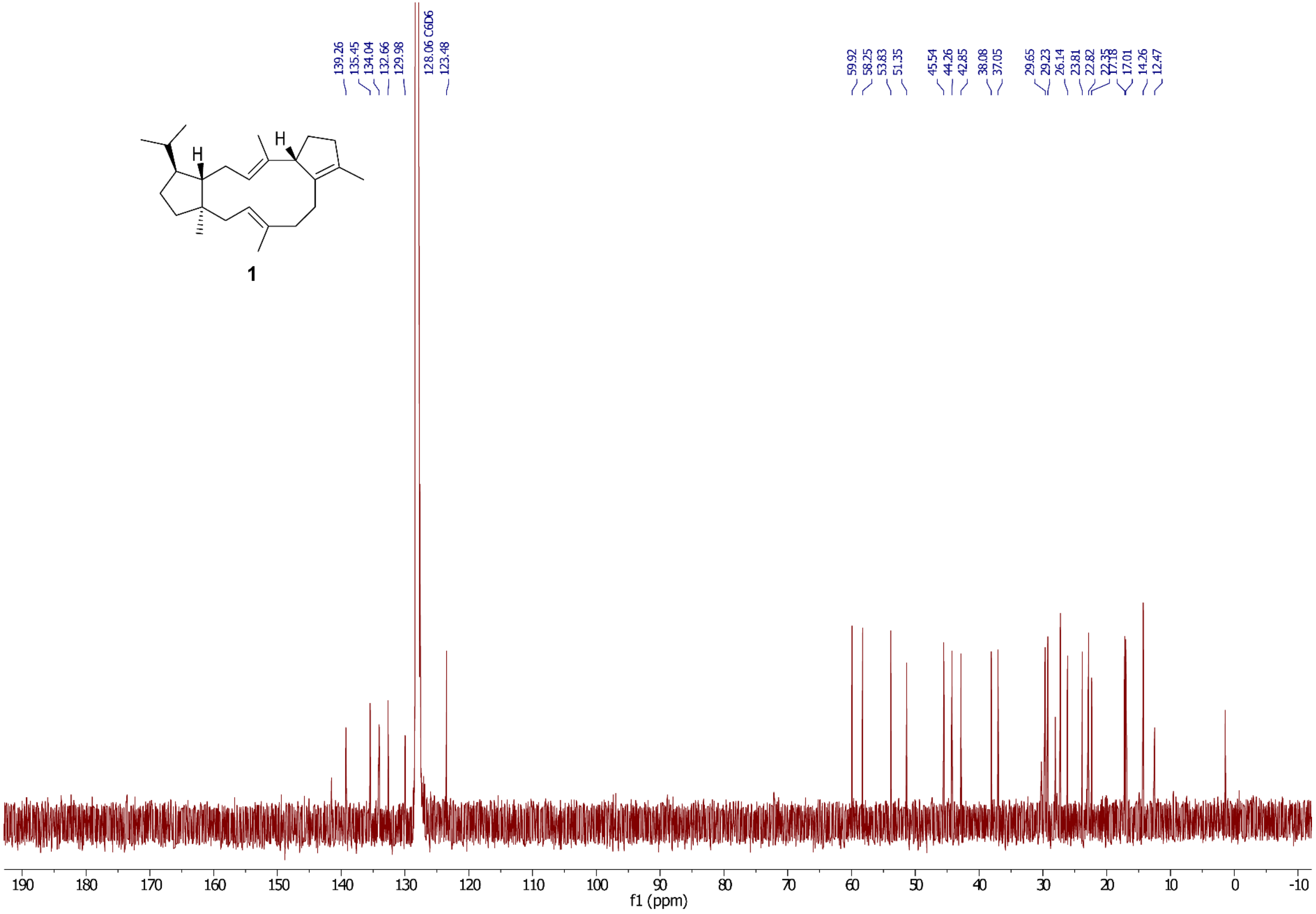

Figure S13. ^{13}C-NMR spectrum of **1** (176 MHz, C_6D_6).

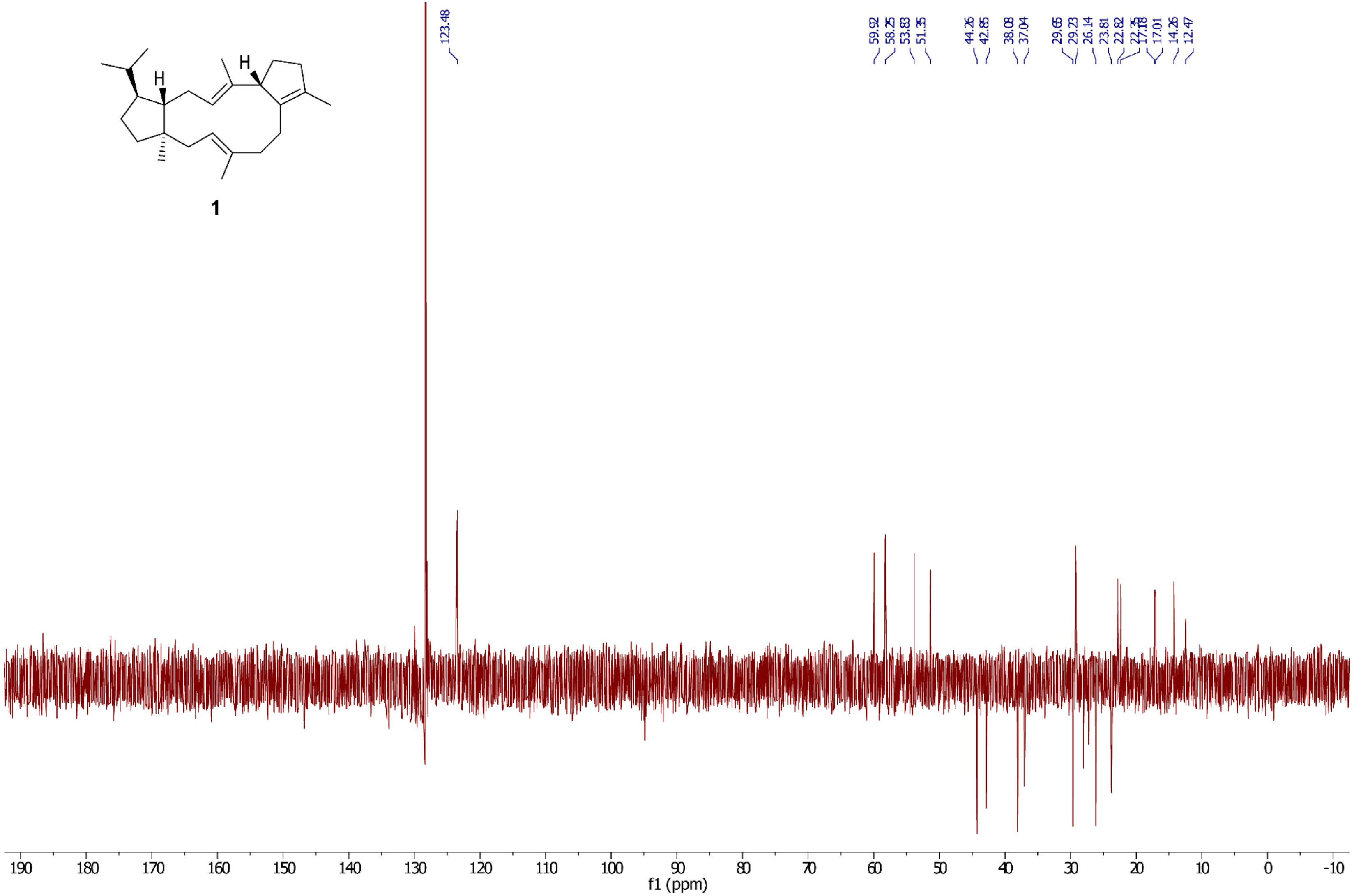

Figure S14. DEPT spectrum of **1** (176 MHz, C_6D_6).

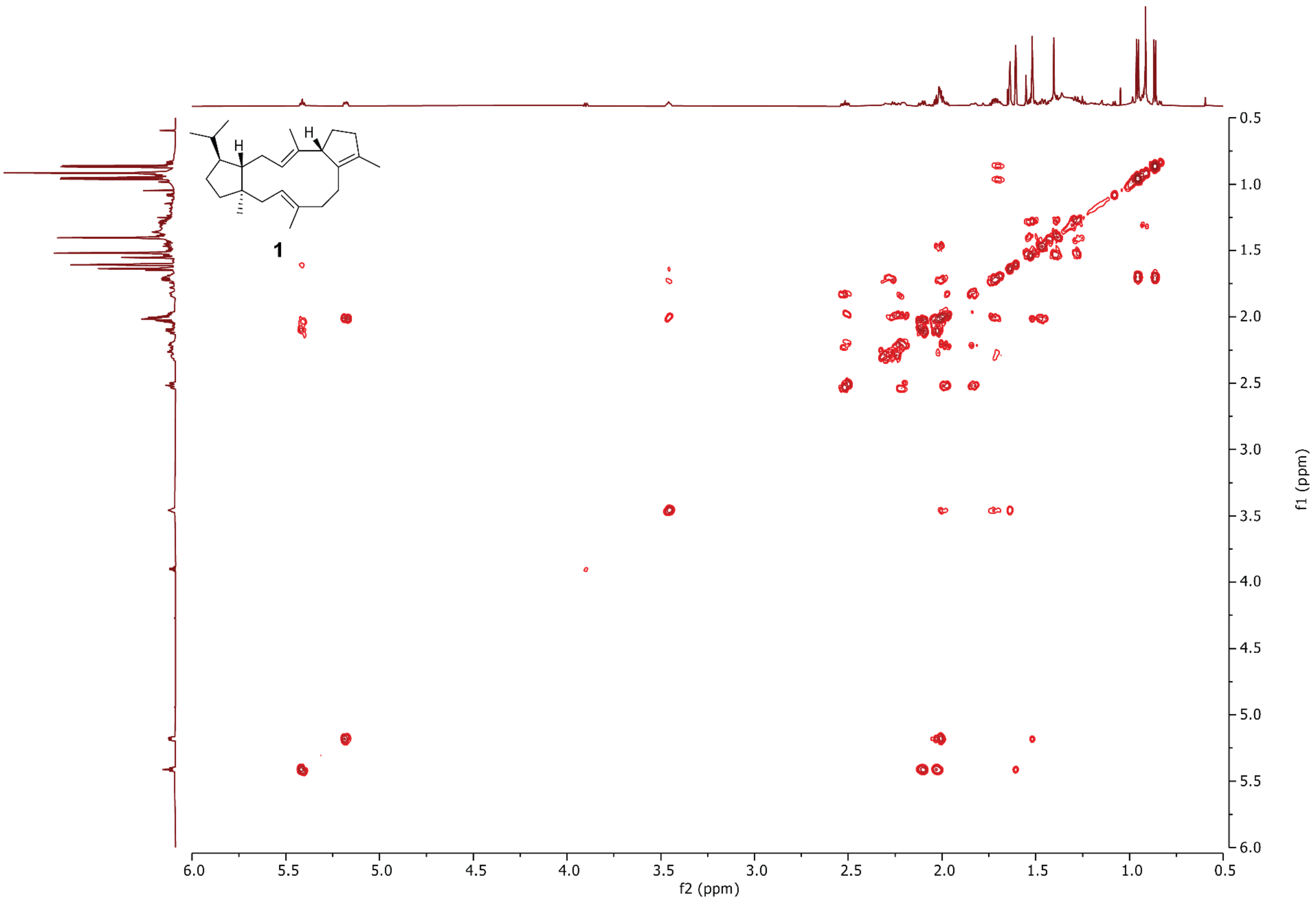

Figure S15. $^{1}H,^{1}H$-COSY spectrum of **1** (700 MHz, C_6D_6).

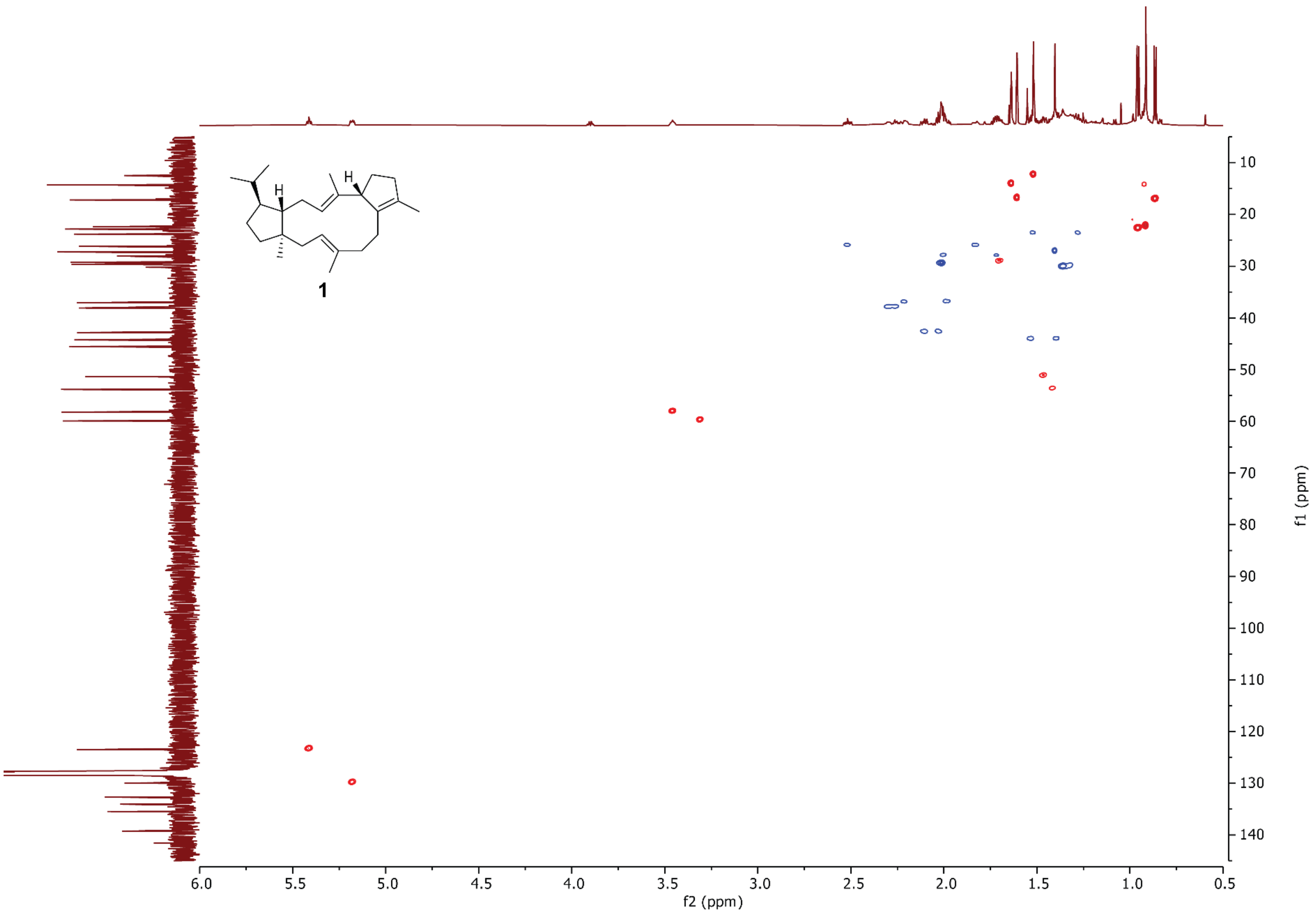

Figure S16. HMQC spectrum of **1** (C_6D_6).

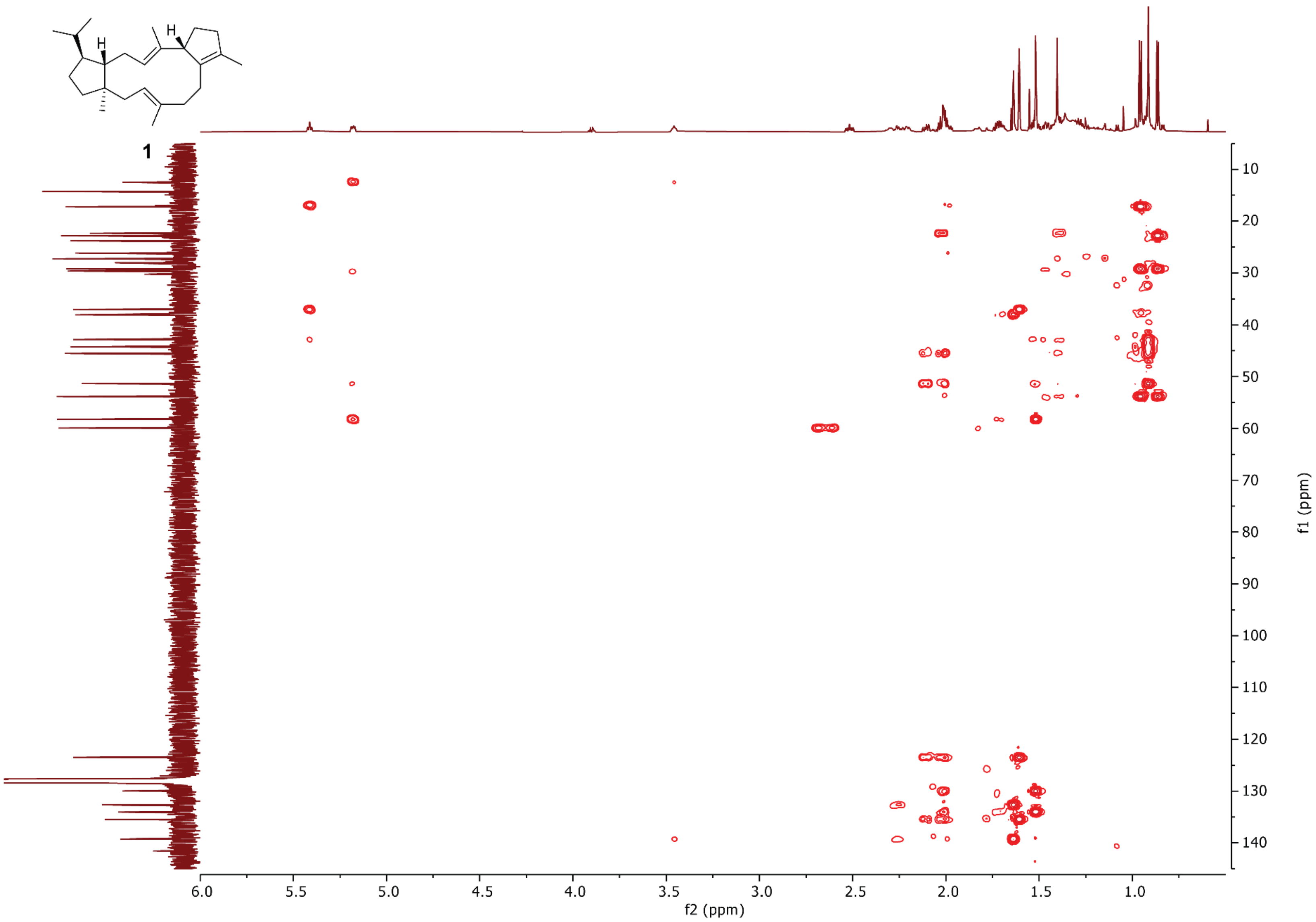

Figure S17. HMBC spectrum of **1** (C_6D_6).

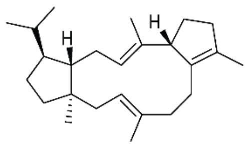

1

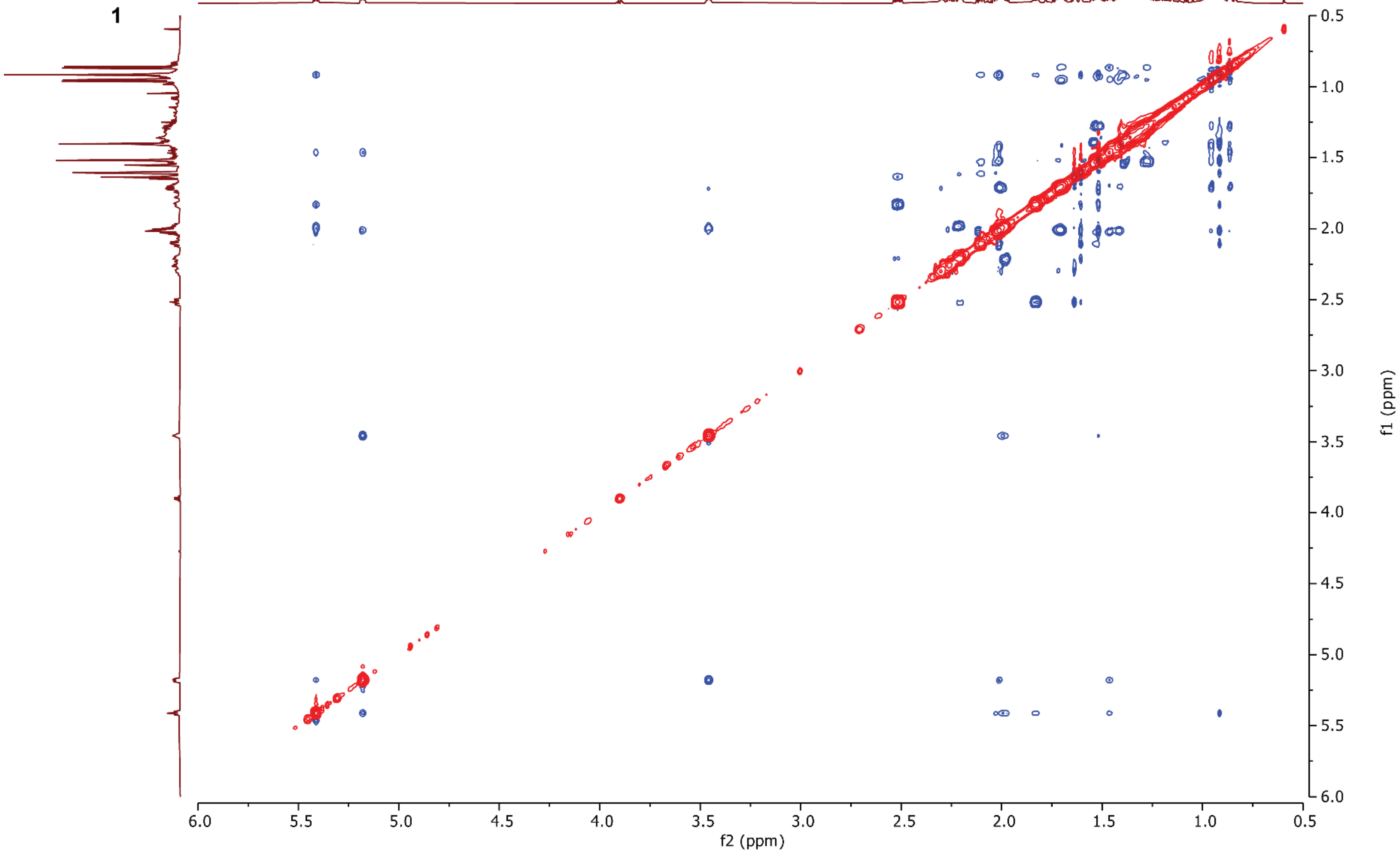

Figure S18. NOESY spectrum of **1** (700 MHz, C_6D_6).

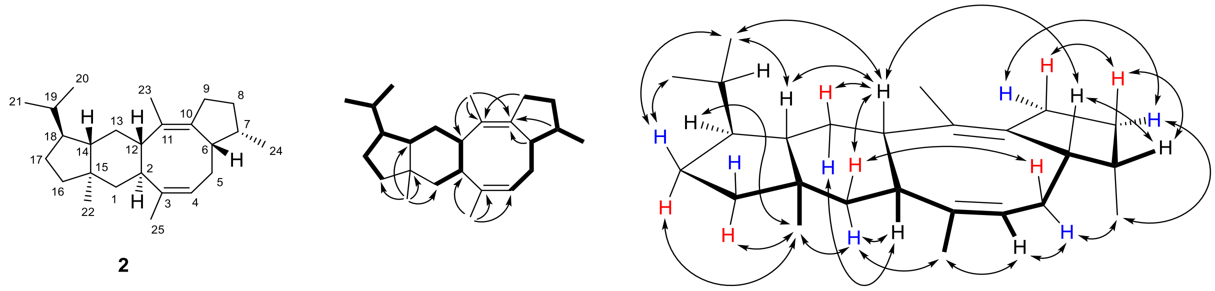

Figure S19. Structure elucidation of **2**. Bold: ^{1}H,^{1}H-COSY correlations, single-headed arrows: key HMBC correlations, and double headed arrows: NOESY correlations.

Table S3. NMR data of aspergildiene A (**2**) in C_6D_6 recorded at 298 K.

C[a]		δ_C[b]	δ_H[b]	δ_C[c]	δ_H[c]
1	CH_2	44.6	1.35 (m, 1H, H_β)<br>1.95 (dd, $^3J_{H,H}$ = 12.4, 3.2, 1H, H_α)	44.3	1.20 (m, 1H)<br>1.84 (dd, J = 10.6, 2.2, 1H)
2	CH	45.4	2.11 (td, $^3J_{H,H}$ = 12.2, 3.2, 1H)	45.0	1.98 (dt, J = 12.2, 2.2, 1H)
3	C_q	140.0	–	140.1	–
4	CH	121.6	5.28 (m, 1H)	121.0	5.08 (t, J = 8.7, 1H)
5	CH_2	29.4	1.90 (m, 1H, H_α)<br>2.90 (m, 1H, H_β)	29.1	1.51* (m, 1H)<br>2.80 (m, 1H)
6	CH	47.6	2.90 (m, 1H)	47.2	2.93 (m, 1H)
7	CH	40.0	1.90 (m, 1H)	39.7	2.14 (m, 1H)
8	CH_2	32.9	1.38 (m, 1H, H_β)<br>1.59 (m, 1H, H_α)	32.7	1.39 (m, 1H)<br>1.60 (m, 1H)
9	CH_2	30.0	2.28 (m, 1H, H_β)<br>2.34 (m, 1H, H_α)	29.7	2.21 (m, 2H)
10	C_q	137.7	–	137.5	–
11	C_q	128.8	–	128.9	–
12	CH	47.6	3.04 (m, 1H)	47.1	2.87 (m, 1H)
13	CH_2	29.4	1.26 (m, 1H, H_α)<br>1.64 (m, 1H, H_β)	29.0	1.17 (m, 1H)<br>1.88* (dt, J = 14.2, 8.6, 1H)
14	CH	51.7	1.26 (m, 1H)	51.3	1.18 (m, 1H)
15	C_q	42.7	–	42.6	–
16	CH_2	39.5	1.18 (m, 1H, H_β)<br>1.47 (m, 1H, H_α)	39.3	1.11 (m, 1H)<br>1.42 (m, 1H)
17	CH_2	23.7	1.39 (m, 1H, H_β)<br>1.70 (m, 1H, H_α)	23.3	1.37 (m, 1H)<br>1.67 (m, 1H)
18	CH	46.3	1.55 (m, 1H)	46.0	1.55 (m, 1H)
19	CH	29.5	1.58 (m, 1H)	29.0	1.69 (m, 1H)
20	CH_3	22.4	0.94 (d, $^3J_{H,H}$ = 6.9, 3H)	22.3	0.90 (d, J = 6.9, 3H)
21	CH_3	17.9	0.84 (d, $^3J_{H,H}$ = 6.8, 3H)	17.7	0.80 (d, J = 6.8, 3H)
22	CH_3	18.9	0.82 (s, 3H)	18.8	0.83 (s, 3H)
23	CH_3	15.4	1.67 (s, 3H)	15.1	1.54 (s, 3H)
24	CH_3	17.0	0.87 (d, $^3J_{H,H}$ = 7.1, 3H)	16.9	0.81 (s, 3H)
25	CH_3	26.2	1.73 (s, 3H)	26.1	1.61 (s, 3H)

[a] Carbon numbering indicating the origin for each carbon from GFPP by same number and colour code for diastereotopic hydrogens as shown in Figure S19. [b] Chemical shifts δ in ppm, multiplicity: s = singlet, d = doublet, t = triplet, m = multiplet, coupling constants J are given in Hertz. [c] Chemical shifts δ in ppm reported previously (recorded in $CDCl_3$).[12] Asterisks indicate assignments that must be interchanged.

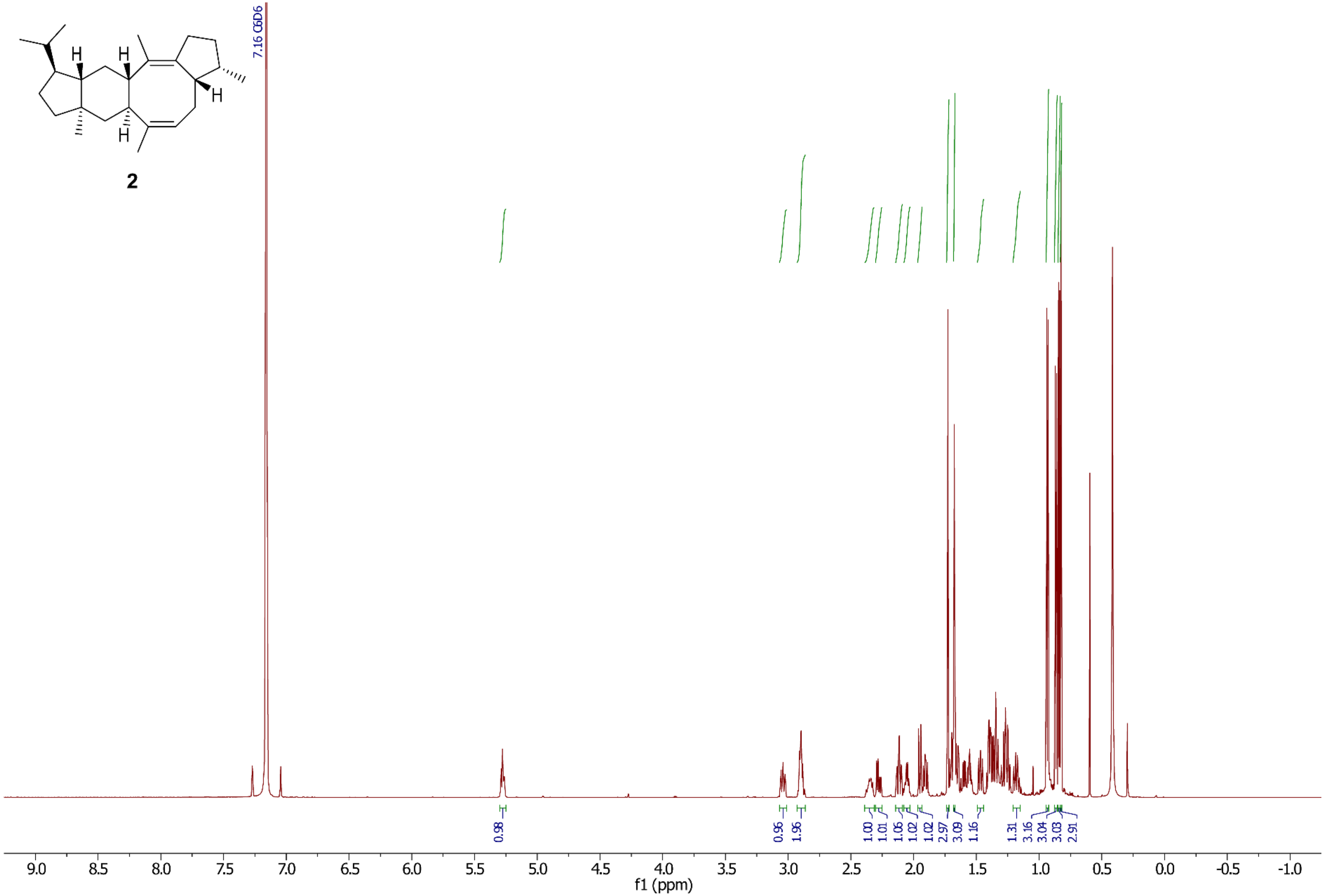

Figure S20. ^{1}H-NMR spectrum of **2** (700 MHz, C_6D_6).

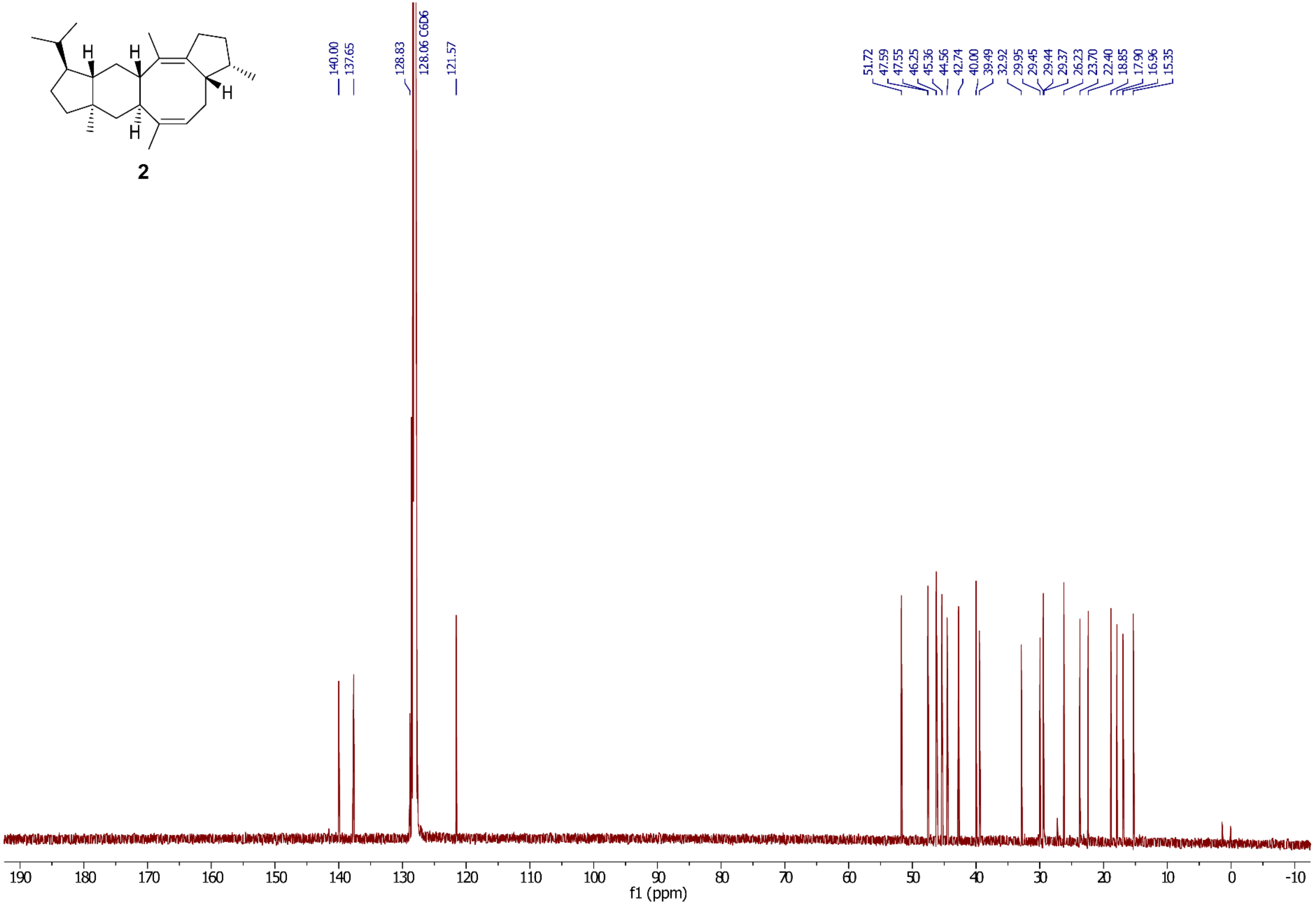

Figure S21. ^{13}C-NMR spectrum of **2** (176 MHz, C_6D_6).

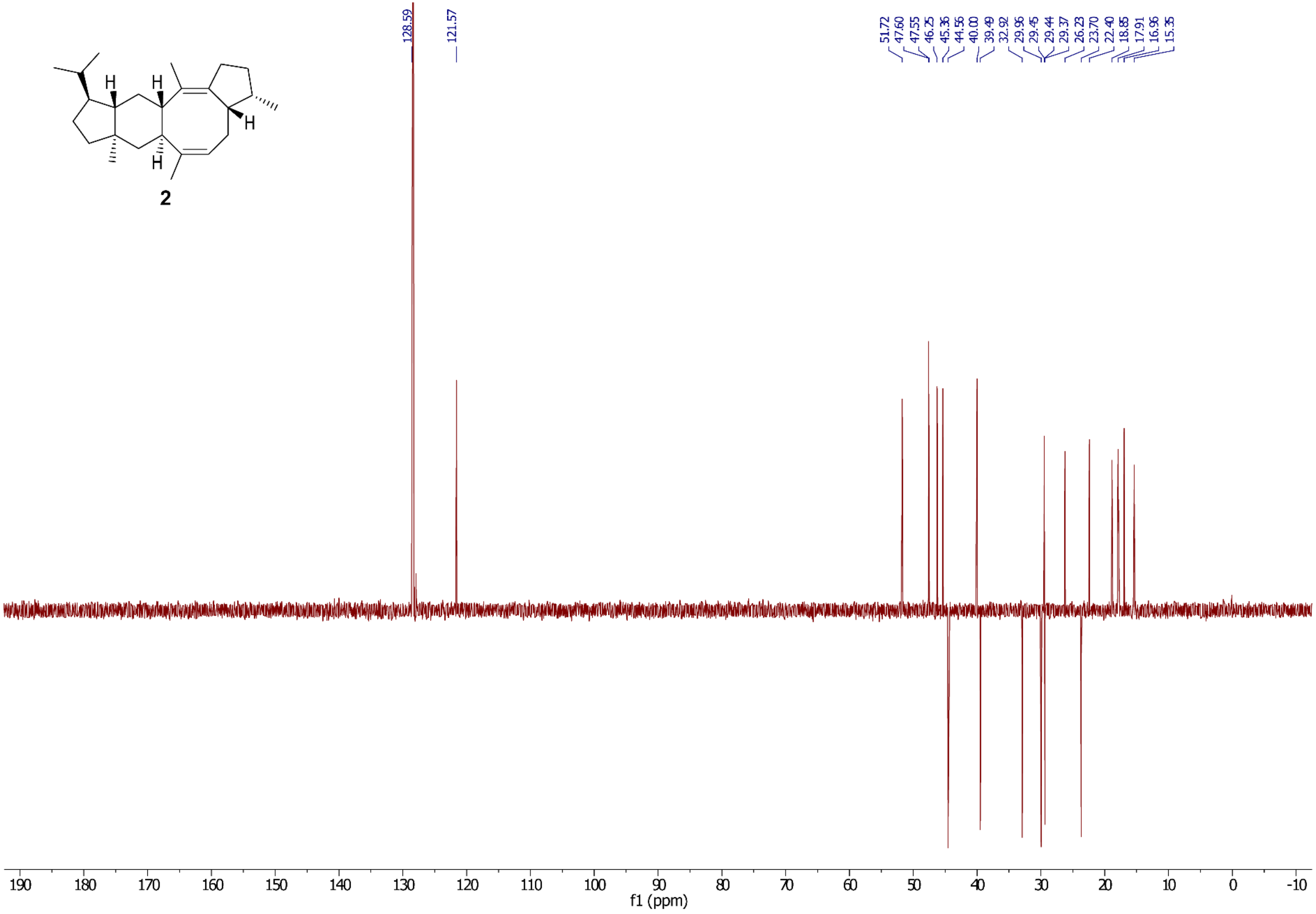

2

Figure S22. DEPT spectrum of **2** (176 MHz, C_6D_6).

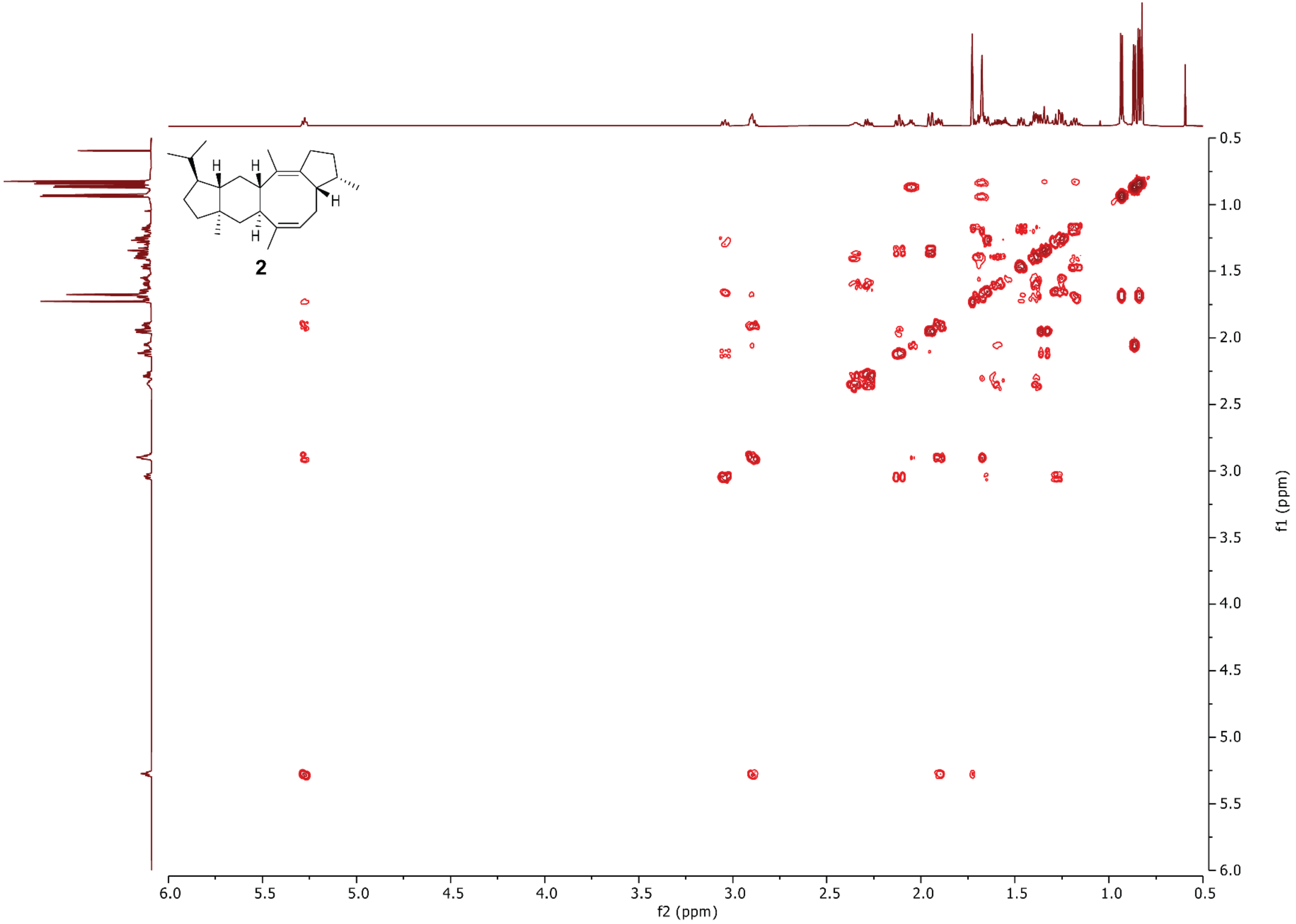

Figure S23. ^{1}H,^{1}H-COSY spectrum of **2** (700 MHz, C_6D_6).

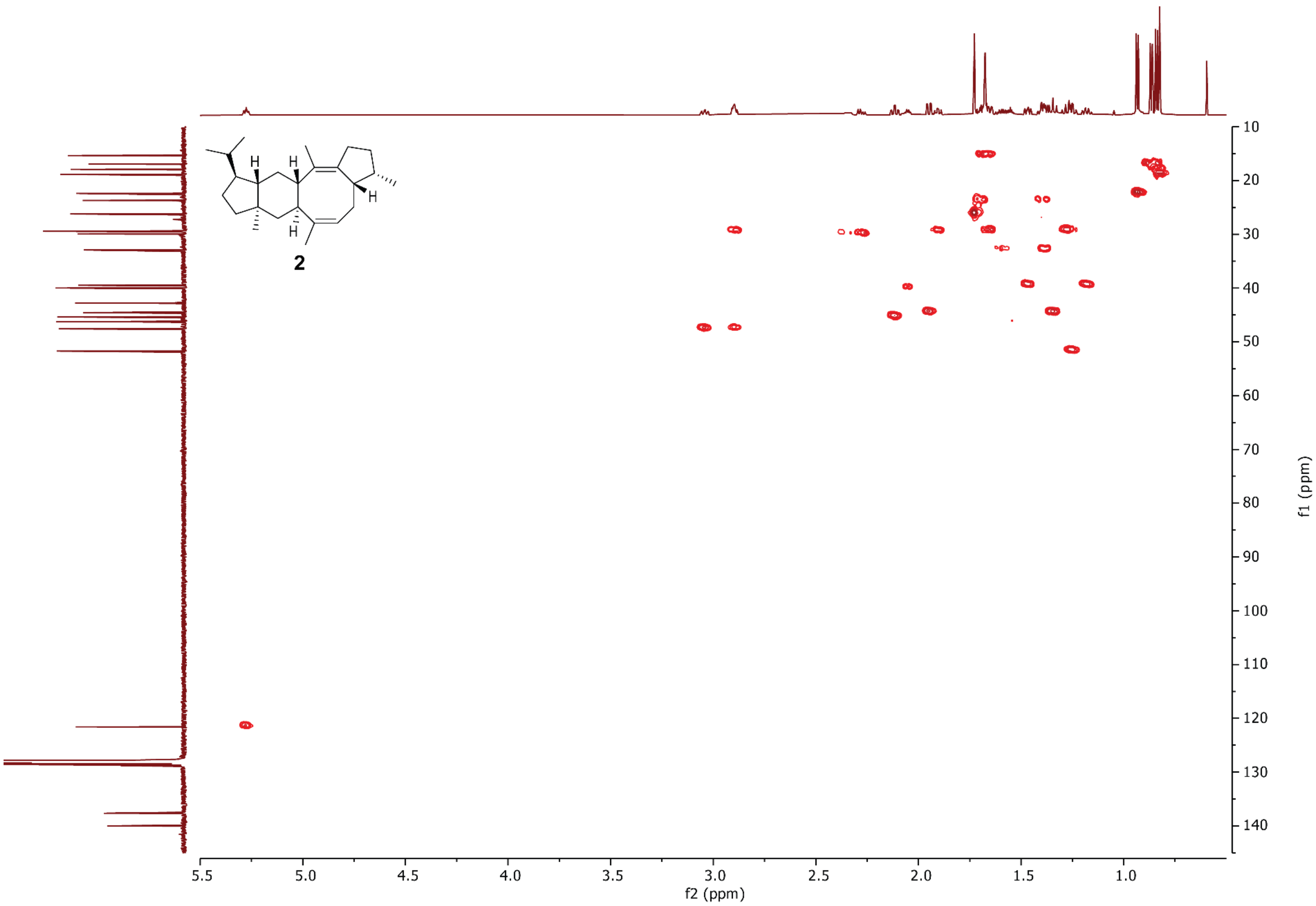

Figure S24. HMQC spectrum of **2** (C_6D_6).

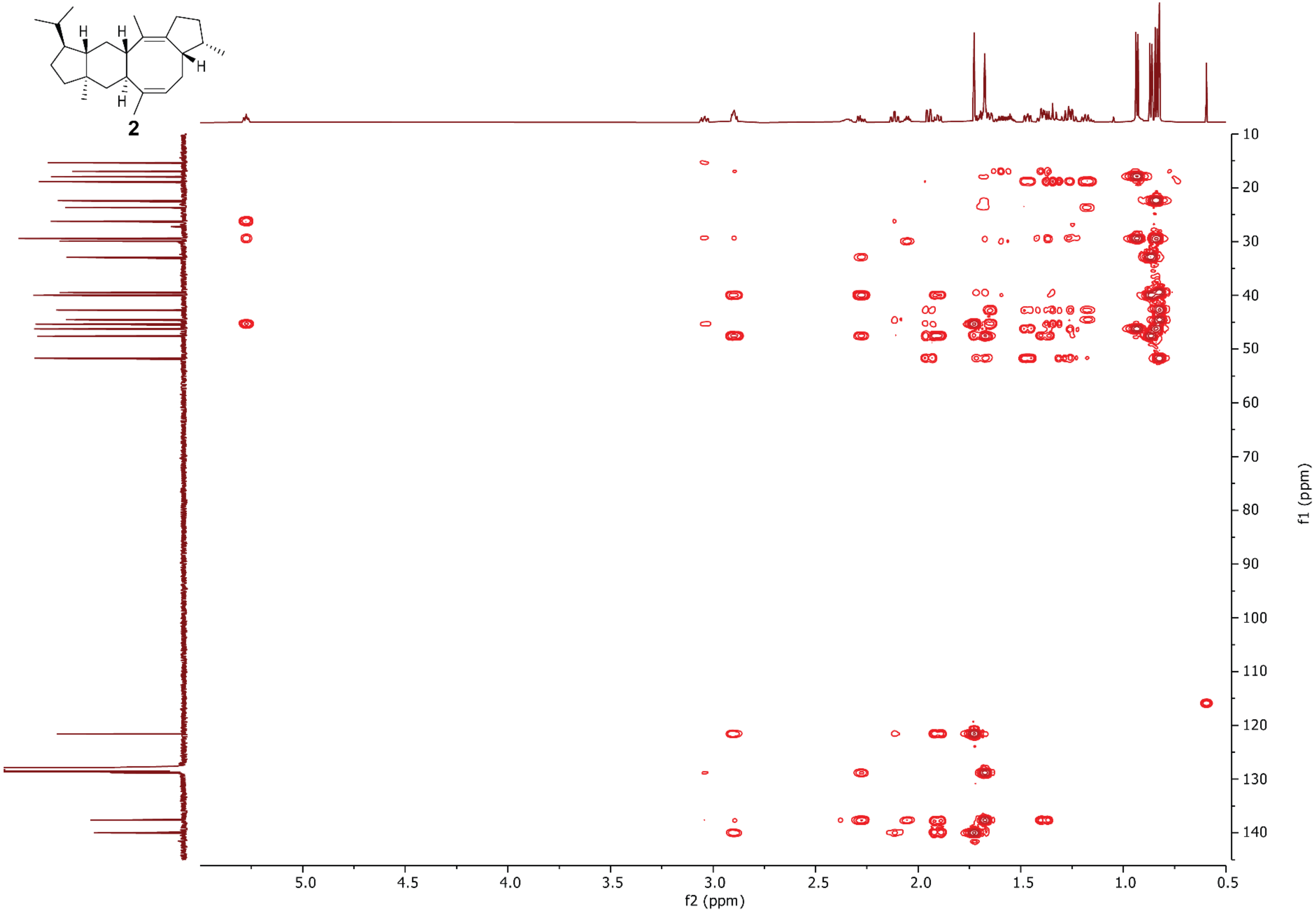

Figure S25. HMBC spectrum of **2** (C_6D_6).

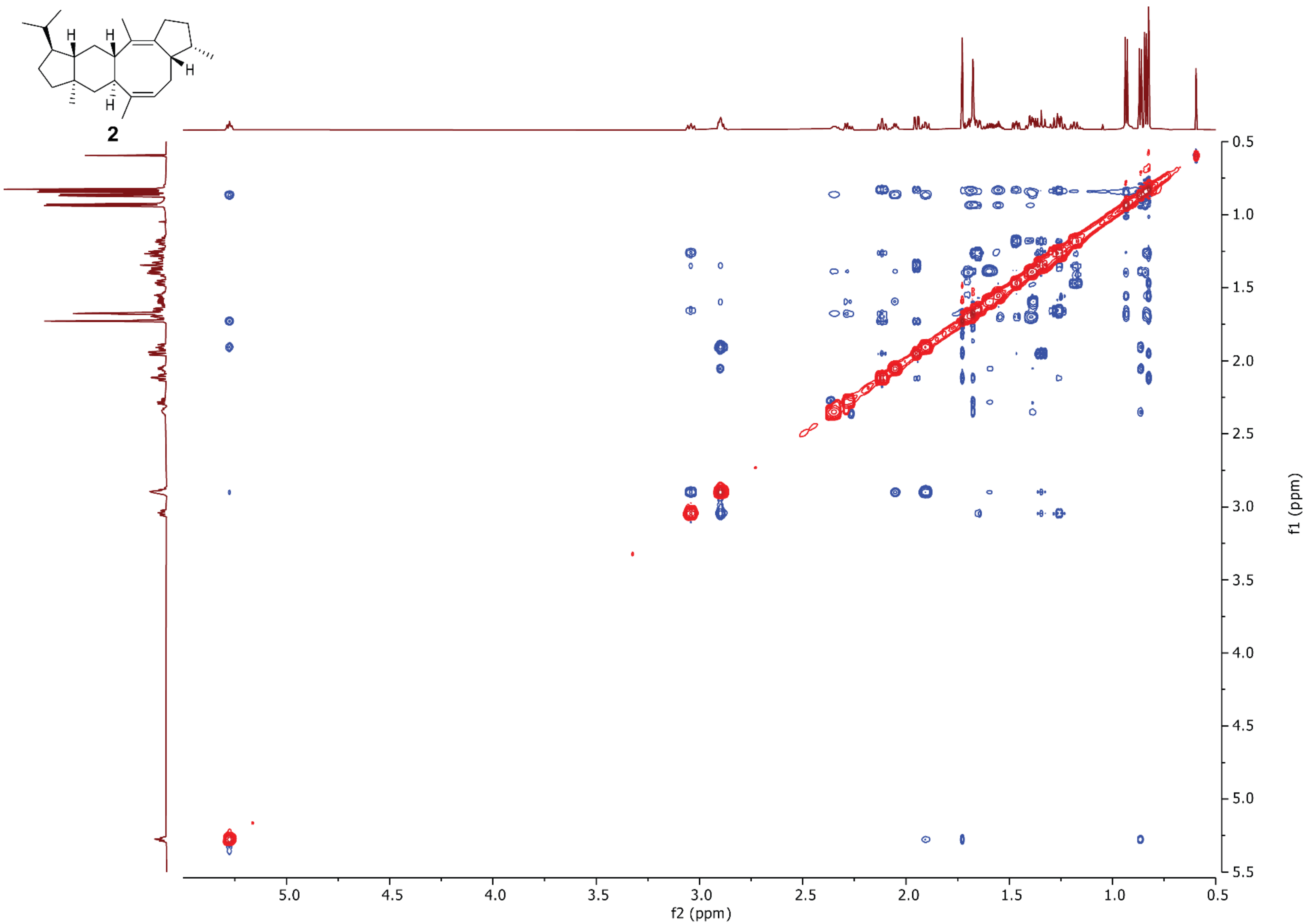

Figure S26. NOESY spectrum of **2** (700 MHz, C_6D_6).

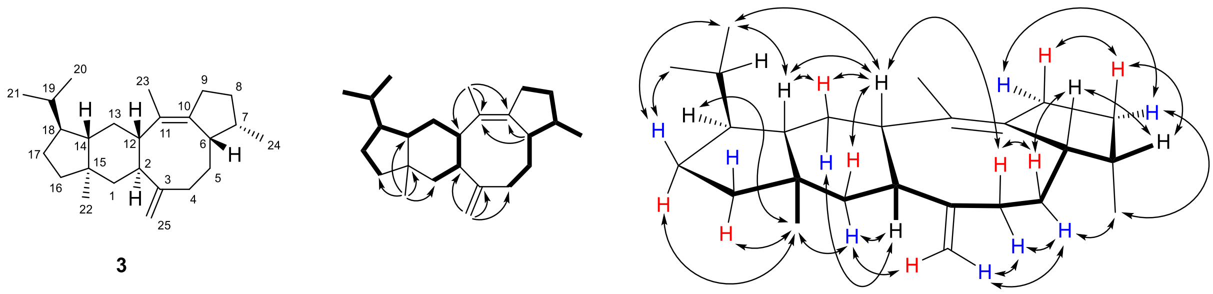

Figure S27. Structure elucidation of **3**. Bold: $^{1}H,^{1}H$-COSY correlations, single-headed arrows: key HMBC correlations, and double headed arrows: NOESY correlations.

Table S4. NMR data of aspergildiene B (**3**) in C_6D_6 recorded at 298 K.

C[a]		δ_C[b]	δ_H[b]	δ_C[c]	δ_H[c]
1	CH_2	46.3	1.34 (m, 1H, H_β)<br>1.71 (m, 1H, H_α)	46.1	1.24 (m, 1H)<br>1.53 (m, 1H)
2	CH	48.5	2.51 (m, 1H)	48.1	2.36 (m, 1H)
3	C_q	156.3	–	156.6	–
4	CH_2	30.8	1.88 (m, 1H, H_β)<br>2.26 (m, 1H, H_α)	30.6	1.98 (t, J = 12.9 Hz, 1H)<br>2.25 (m, 1H)
5	CH_2	31.6	1.07 (m, 1H, H_α)<br>1.69 (m, 1H, H_β)	31.4	1.31* (m, 1H)<br>1.78 (dt, J = 13.0, 5.0, 2.4, 1H)
6	CH	46.7	2.53 (m, 1H)	46.5	2.55 (dd, J = 12.1, 7.0, 1H)
7	CH	39.4	1.91 (m, 1H)	39.2	1.95 (m, 1H)
8	CH_2	31.6	1.33 (m, 1H, H_α)<br>1.64 (m, 1H, H_β)	31.4	1.03* (dt, J = 12.9, 2.2, 1H)<br>1.68 (m, 1H)
9	CH_2	29.8	2.21 (m, 1H, H_β)<br>2.33 (m, 1H, H_α)	29.5	2.15 (dd, J = 16.4, 7.8, 2H)<br>2.25 (d, J = 16.4, 1H)
10	C_q	141.2	–	141.0	–
11	C_q	129.1	–	129.0	–
12	CH	46.0	2.57 (ddd, $^3J_{H,H}$ = 11.6, 11.5, 3.9, 1H)	45.6	2.48 (t, J = 11.4, 1H)
13	CH_2	28.0	1.42 (m, 1H, H_α)<br>1.66 (m, 1H, H_β)	27.7	1.34 (m, 1H)<br>1.55 (m, 1H)
14	CH	50.9	1.18 (m, 1H)	50.5	1.10 (m, 1H)
15	C_q	42.3	–	42.1	–
16	CH_2	39.8	1.16 (m, 1H, H_β)<br>1.46 (m, 1H, H_α)	39.6	1.09 (m, 1H)<br>1.42 (m, 1H)
17	CH_2	23.7	1.39 (m, 1H, H_β)<br>1.69 (m, 1H, H_α)	23.4	1.36 (m, 1H)<br>1.69 (m, 1H)
18	CH	46.4	1.55 (m, 1H)	46.2	1.58 (m, 1H)
19	CH	29.6	1.67 (m, 1H)	29.3	1.69 (m, 1H)
20	CH_3	22.4	0.94 (d, $^3J_{H,H}$ = 6.9, 3H)	22.3	0.91 (d, J = 6.8, 3H)
21	CH_3	18.0	0.86 (d, $^3J_{H,H}$ = 6.9, 3H)	19.7	0.82 (d, J = 6.9, 3H)
22	CH_3	18.9	0.87 (s, 3H)	18.8	0.84 (s, 3H)
23	CH_3	14.2	1.60 (br s, 3H)	14.0	1.47 (s, 3H)
24	CH_3	15.9	0.95 (d, $^3J_{H,H}$ = 6.9, 3H)	15.8	0.96 (d, J = 6.8, 3H)
25	CH_2	111.2	4.75 (br s, 1H)<br>4.82 (br s, 1H)	110.7	4.57 (br s, 1H)<br>4.61 (br s, 1H)

[a] Carbon numbering indicating the origin for each carbon from GFPP by same number and colour code for diastereotopic hydrogens as shown in Figure S27. [b] Chemical shifts δ in ppm, multiplicity: s = singlet, d = doublet, m = multiplet, br = broad. Coupling constants J are given in Hertz. [c] Chemical shifts δ in ppm reported previously (recorded in $CDCl_3$).[12] Asterisks indicate assignments that must be interchanged.

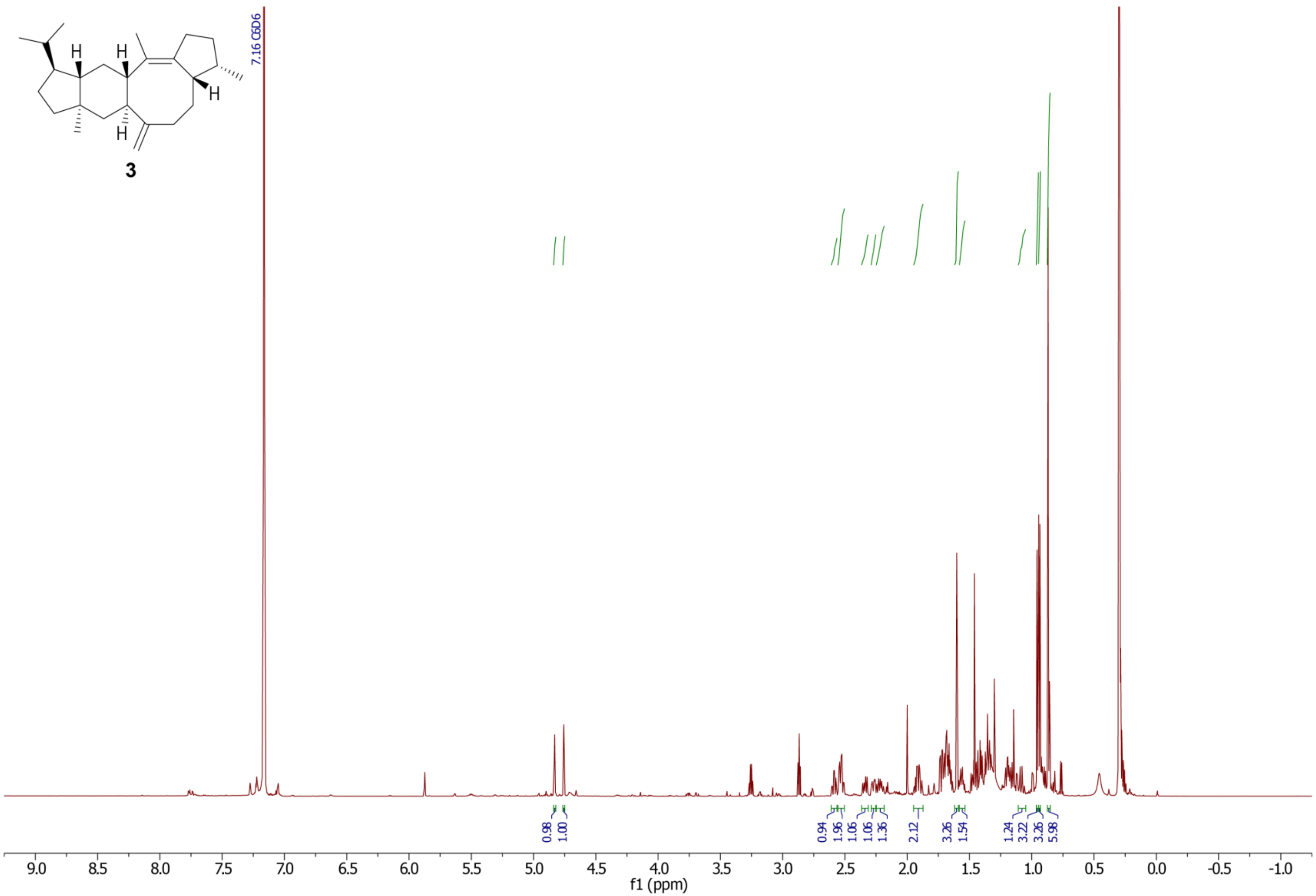

Figure S28. ^{1}H-NMR spectrum of **3** (700 MHz, C_6D_6).

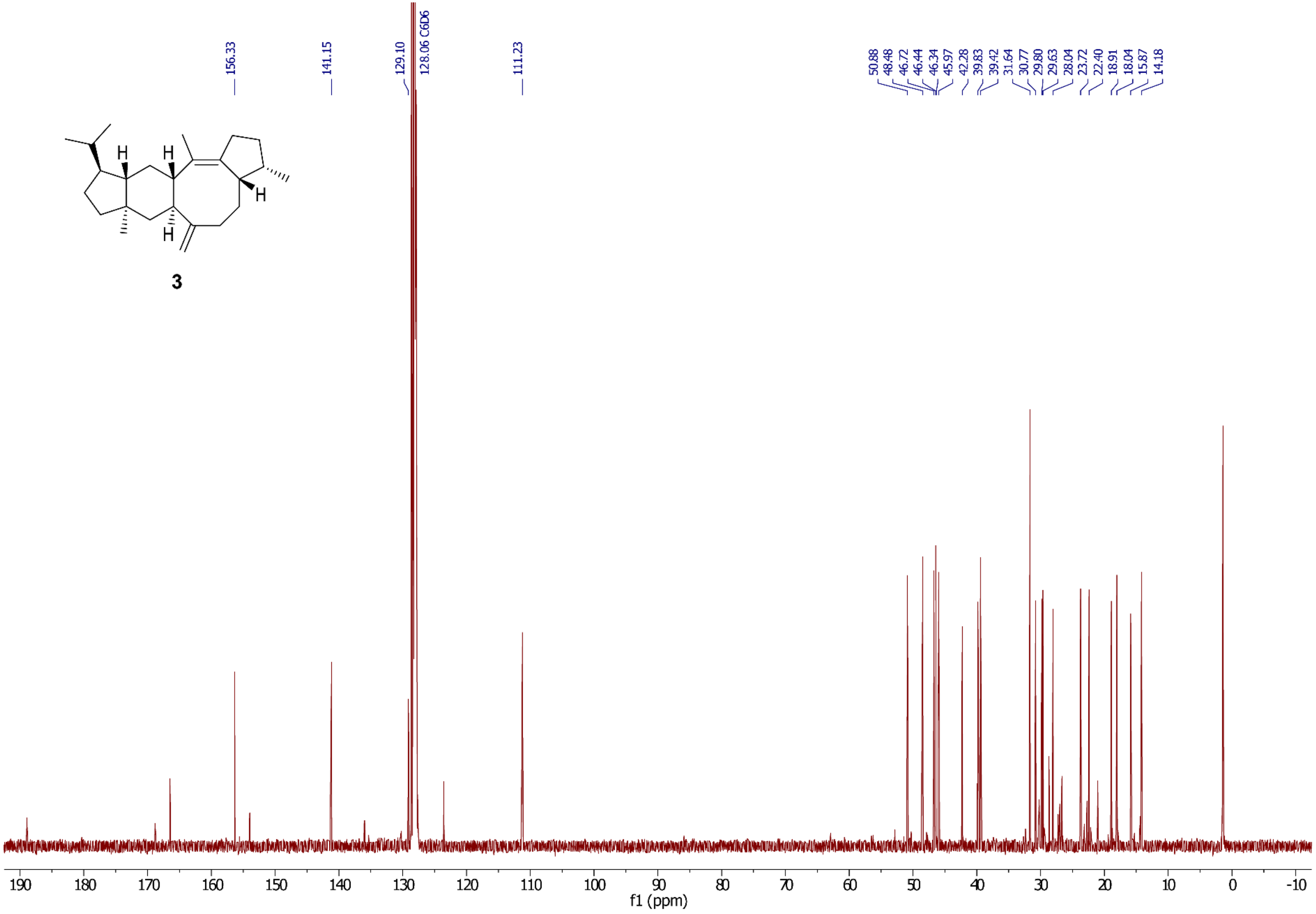

Figure S29. ^{13}C-NMR spectrum of **3** (176 MHz, C_6D_6).

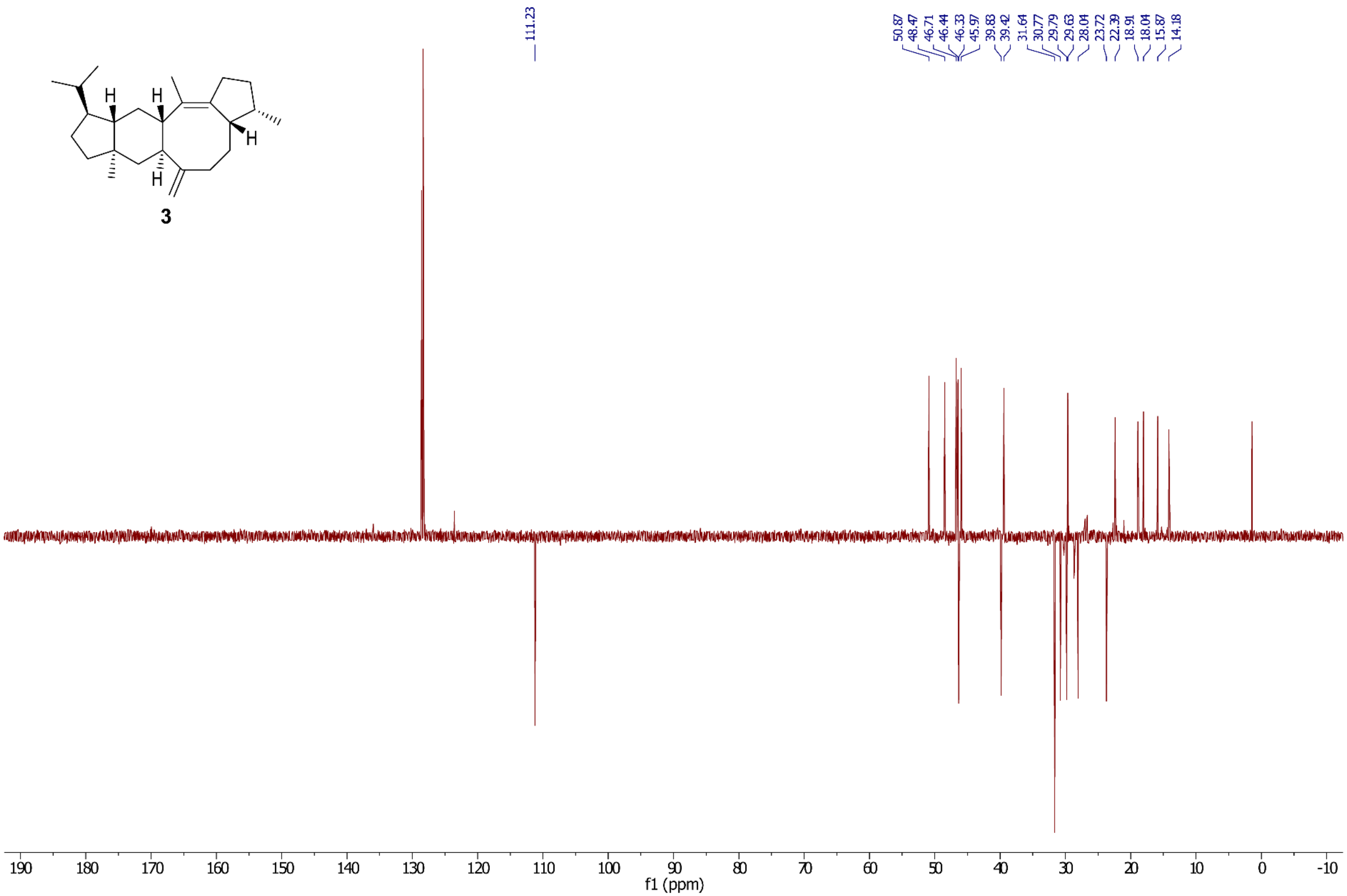

Figure S30. DEPT spectrum of **3** (176 MHz, C_6D_6).

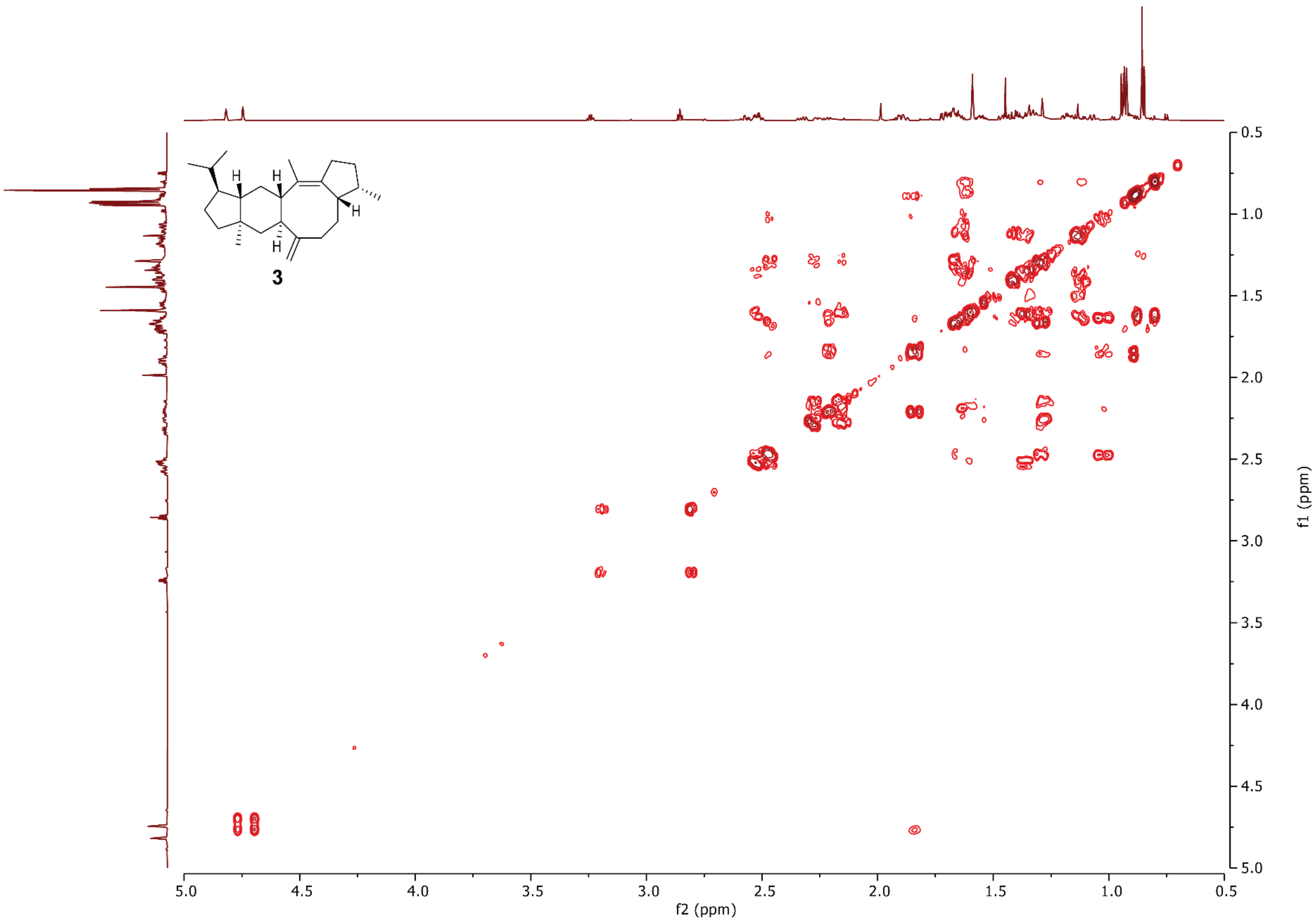

Figure S31. $^{1}H,^{1}H$-COSY spectrum of **3** (700 MHz, C_6D_6).

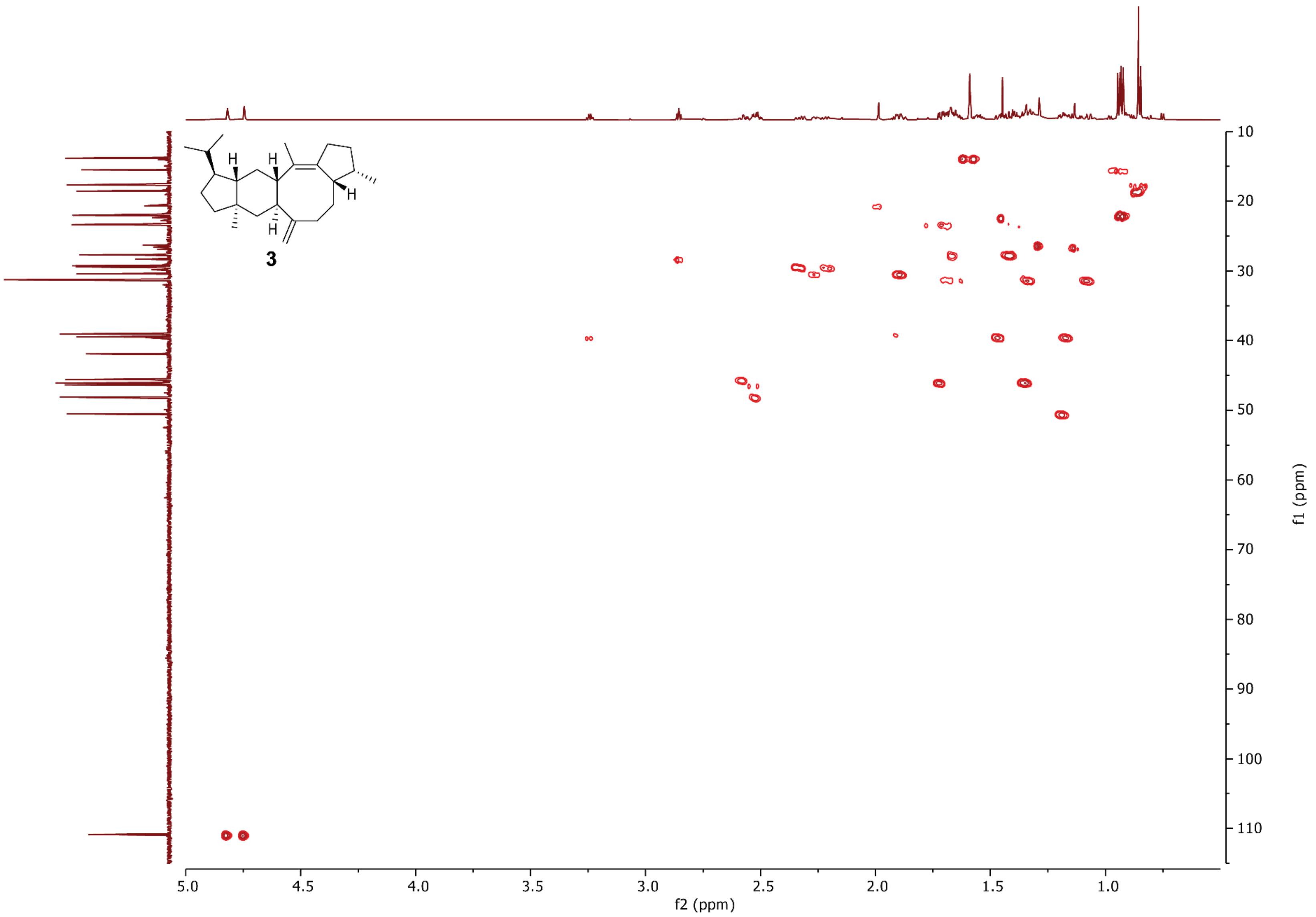

Figure S32. HMQC spectrum of **3** (C_6D_6).

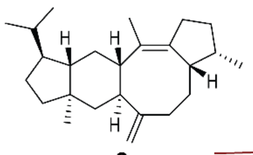

3

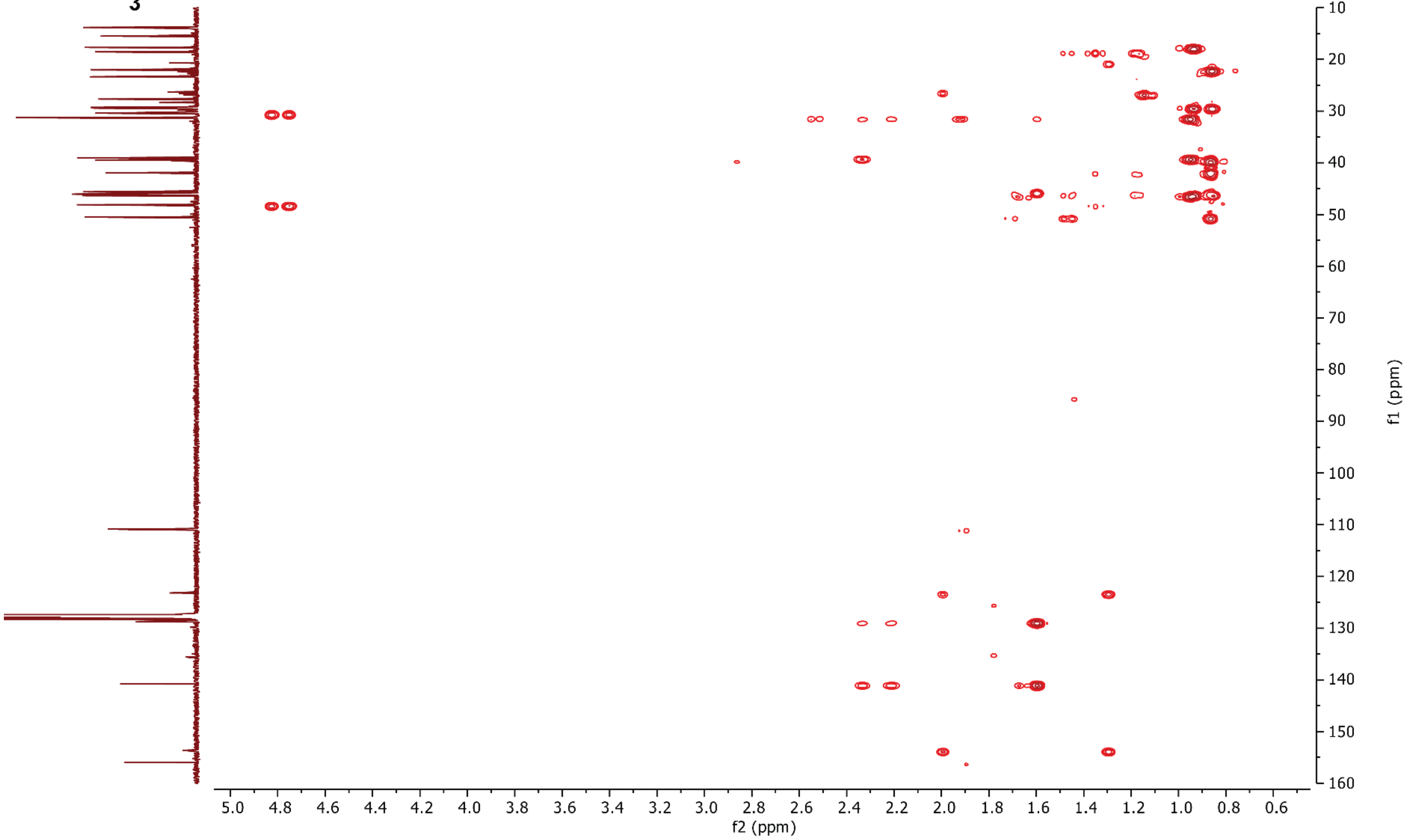

Figure S33. HMBC spectrum of **3** (C_6D_6).

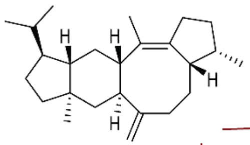

3

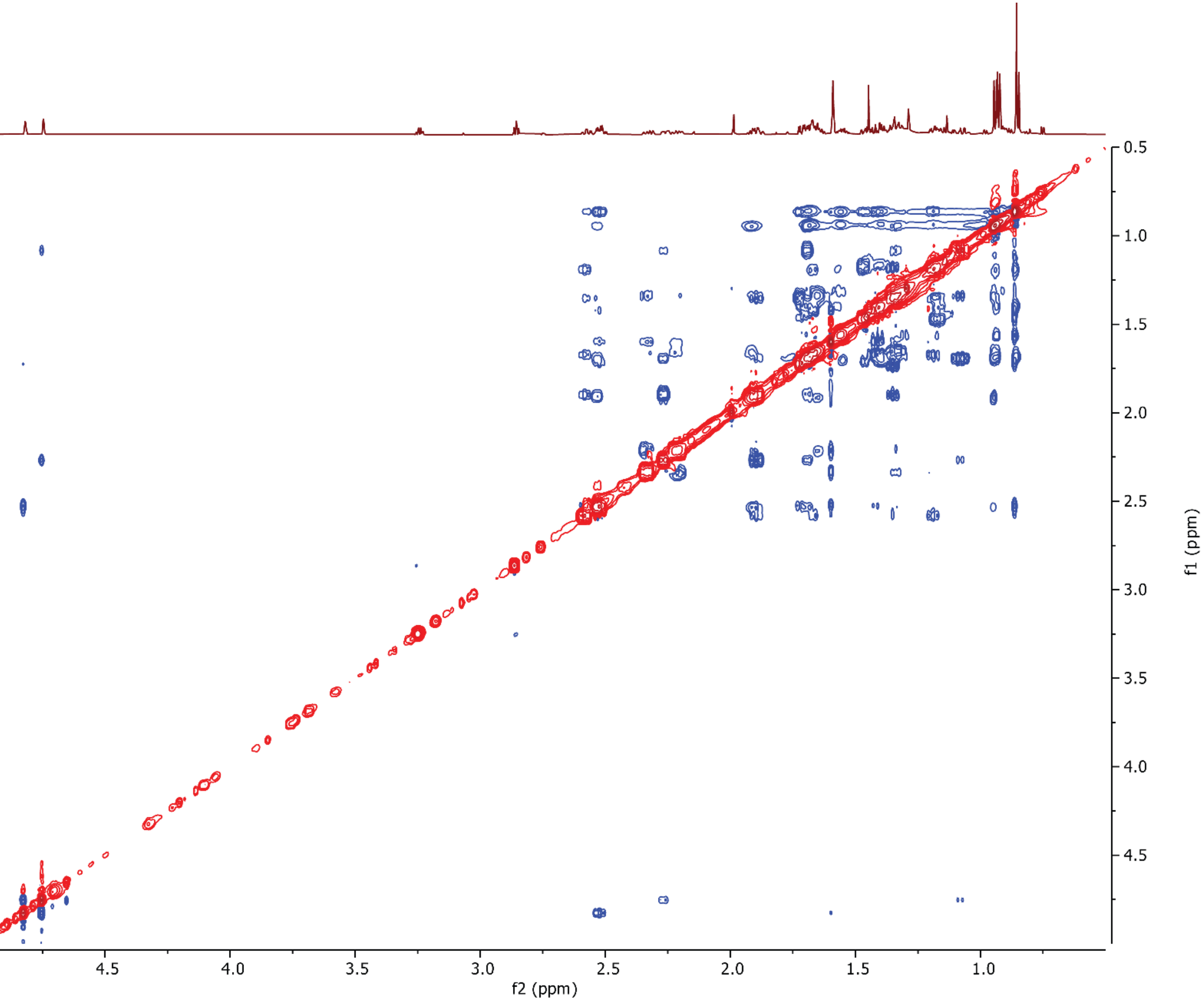

Figure S34. NOESY spectrum of **3** (700 MHz, C_6D_6).

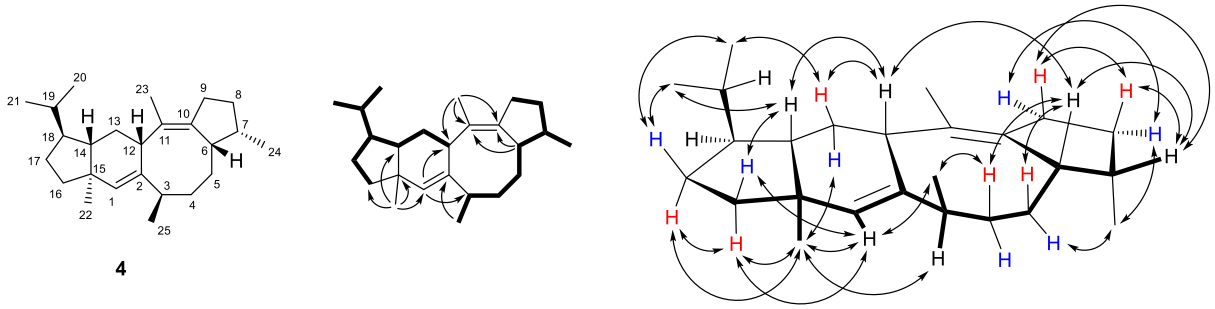

4

Figure S35. Structure elucidation of **4**. Bold: ^{1}H,^{1}H-COSY correlations, single-headed arrows: key HMBC correlations, and double headed arrows: NOESY correlations.

Table S5. NMR data of aspergildiene C (**4**) in C_6D_6 recorded at 298 K.

C[a]		δ_C[b]	δ_H[b]	δ_C[c]	δ_H[c]
1	CH	128.5*	5.84 (m, 1H)	128.1	5.57 (dd, J = 3.4, 2.0, 1H)
2	C_q	144.4	–	144.0	–
3	CH	35.1	2.24 (m, 1H)	34.8	2.06 (m, 1H)
4	CH_2	38.6	1.48 (m, 1H, H_α)<br>1.51 (m, 1H, H_β)	38.3	1.41 (m, 1H)<br>1.48 (m, 1H)
5	CH_2	28.7	0.98 (m, 1H, H_α)<br>1.56 (m, 1H, H_β)	28.4	1.05 (m, 1H)<br>1.65 (m, 1H)
6	CH	47.1	2.61 (m, 1H)	47.0	2.65 (m, 1H)
7	CH	39.4	1.94 (m, 1H)	39.2	2.03 (m, 1H)
8	CH_2	31.7	1.34 (m, 1H, H_α)<br>1.67 (m, 1H, H_β)	31.5	1.40 (m, 1H)<br>1.75 (m, 1H)
9	CH_2	29.9	2.23 (m, 1H, H_β)<br>2.32 (m, 1H, H_α)	29.7	2.24 (dd, J = 17.4, 9.4, 2H)<br>2.37 (dd, J = 17.4, 9.4, 1H)
10	C_q	142.4	–	142.2	–
11	C_q	128.2*	–	128.2	–
12	CH	46.0	3.68 (m, 1H)	45.5	3.48 (m, 1H)
13	CH_2	26.5	1.80 (m, 1H, H_α)<br>1.90 (m, 1H, H_β)	26.1	1.64 (m, 1H)<br>1.72 (m, 1H)
14	CH	48.7	1.55 (m, 1H)	48.1	1.33 (m, 1H)
15	C_q	42.9	–	42.5	–
16	CH_2	36.9	1.38 (m, 1H, H_β)<br>1.51 (m, 1H, H_α)	36.7	1.24 (m, 1H)<br>1.42 (m, 1H)
17	CH_2	24.6	1.42 (m, 1H, H_β)<br>1.74 (m, 1H, H_α)	24.3	1.40 (m, 1H)<br>1.72 (m, 1H)
18	CH	45.8	1.61 (m, 1H)	45.5	1.56 (m, 1H)
19	CH	30.4	1.72 (m, 1H)	30.0	1.69 (m, 1H)
20	CH_3	22.3	0.97 (d, $^3J_{H,H}$ = 6.8, 3H)	22.2	0.93 (d, J = 6.9, 3H)
21	CH_3	18.8	0.90 (d, $^3J_{H,H}$ = 6.8, 3H)	18.7	0.84 (d, J = 6.8, 3H)
22	CH_3	22.9	0.98 (s, 3H)	22.6	0.84 (s, 3H)
23	CH_3	15.3	1.59 (s, 3H)	15.1	1.46 (s, 3H)
24	CH_3	15.8	0.92 (d, $^3J_{H,H}$ = 6.9, 3H)	15.8	0.97 (d, J = 6.8, 3H)
25	CH_3	21.7	1.10 (d, $^3J_{H,H}$ = 7.0, 3H)	21.5	0.94 (d, J = 6.9, 3H)

[a] Carbon numbering indicating the origin for each carbon from GFPP by same number and colour code for diastereotopic hydrogens as shown in Figure S35. [b] Chemical shifts δ in ppm, multiplicity: s = singlet, d = doublet, m = multiplet. Coupling constants J are given in Hertz. Asterisks indicate signals that were covered by the solvent peak. Their assignment is based on the HMQC spectrum. [c] Chemical shifts δ in ppm reported previously (recorded in $CDCl_3$).[12]

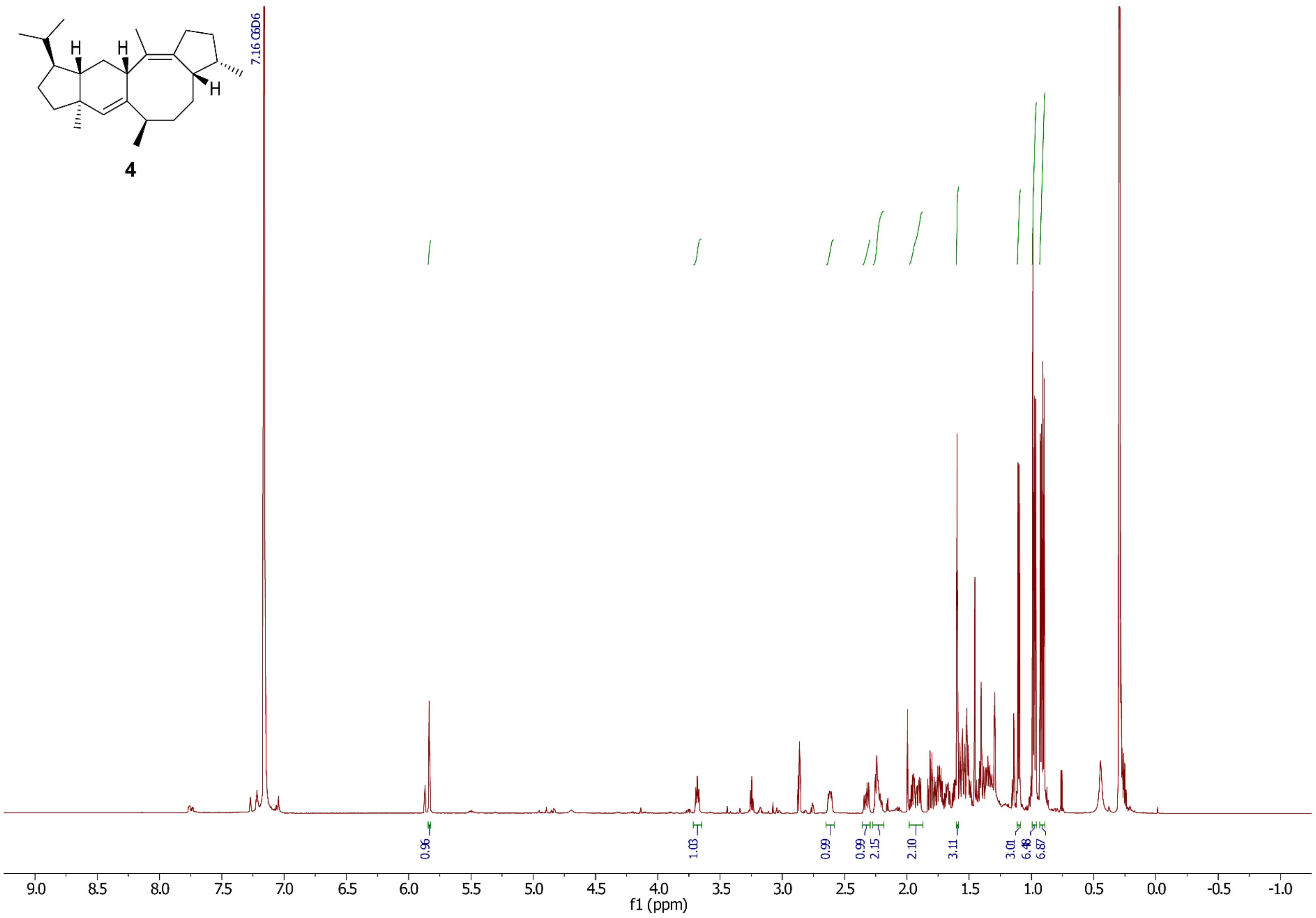

Figure S36. ^{1}H-NMR spectrum of **4** (700 MHz, C_6D_6).

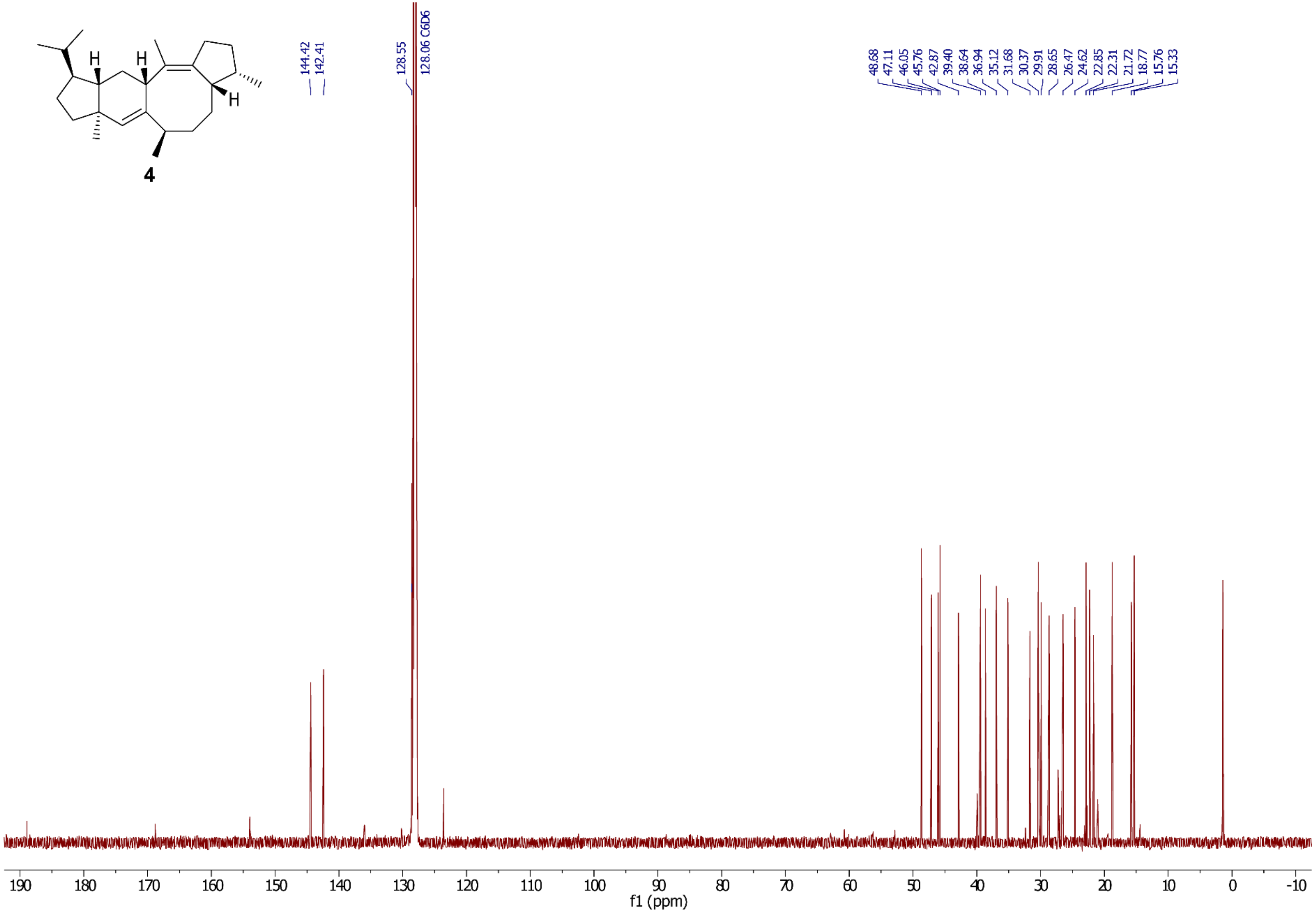

Figure S37. ^{13}C-NMR spectrum of **4** (176 MHz, C_6D_6).

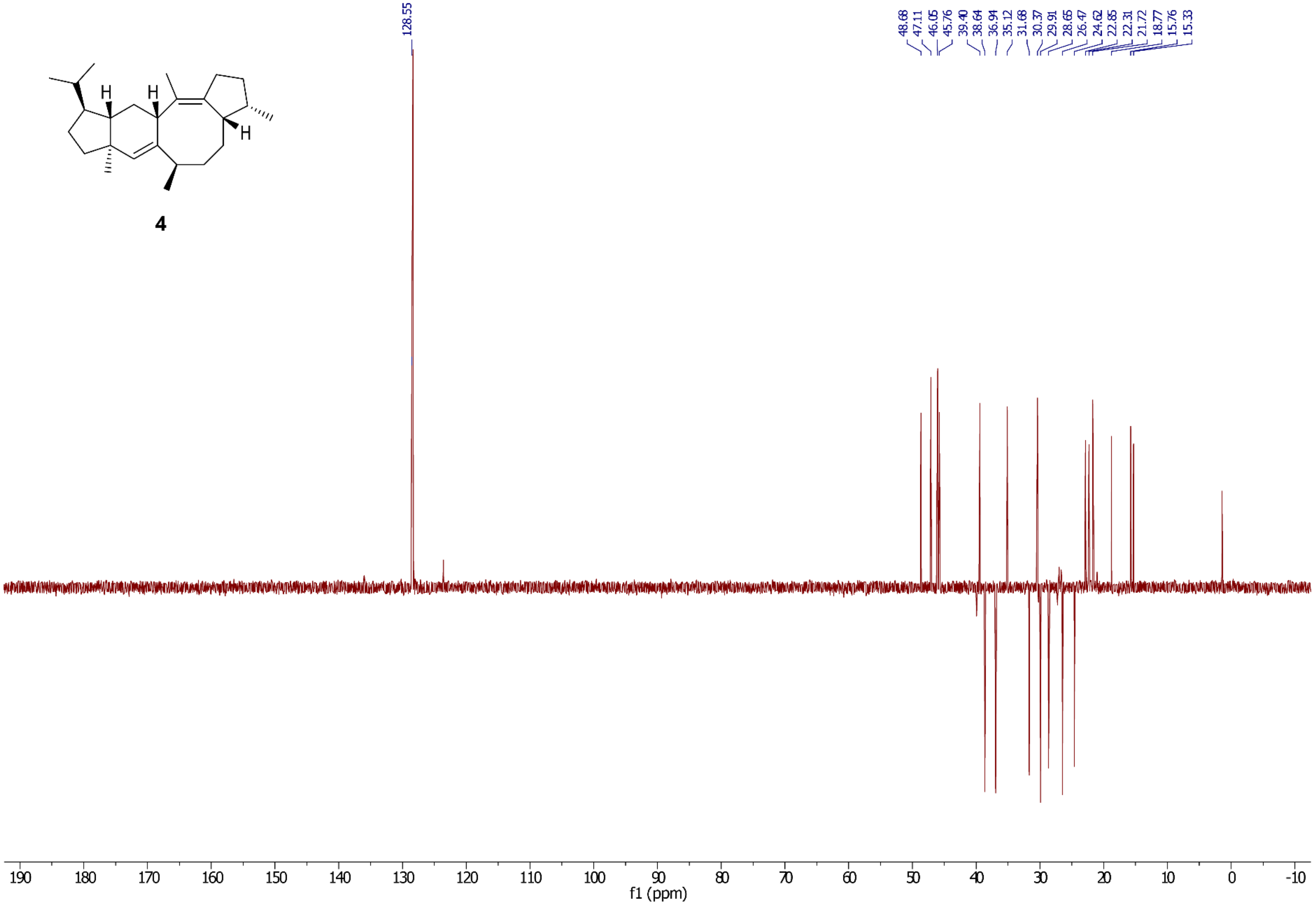

Figure S38. DEPT spectrum of **4** (176 MHz, C_6D_6).

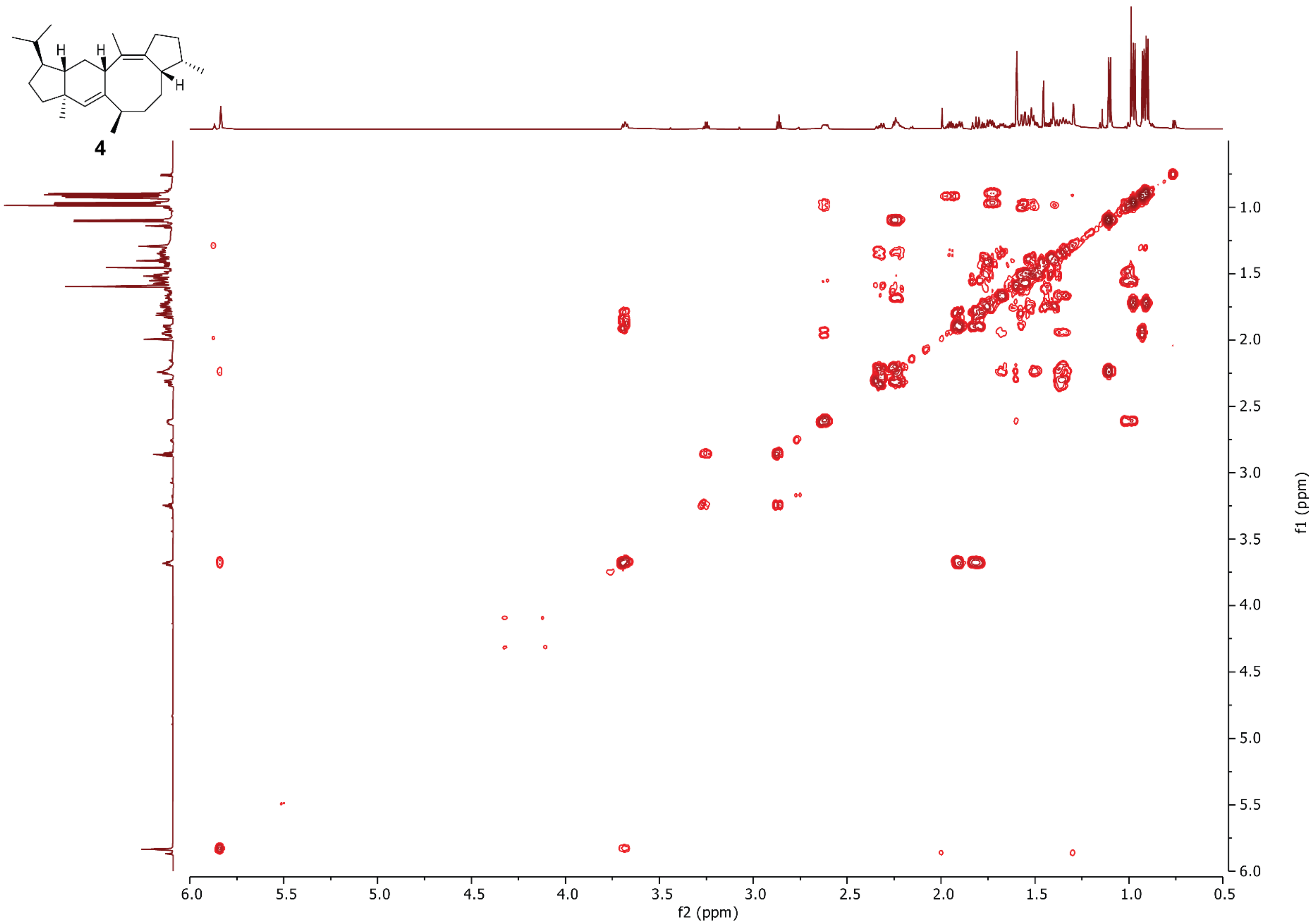

Figure S39. ^{1}H,^{1}H-COSY spectrum of **4** (700 MHz, C_6D_6).

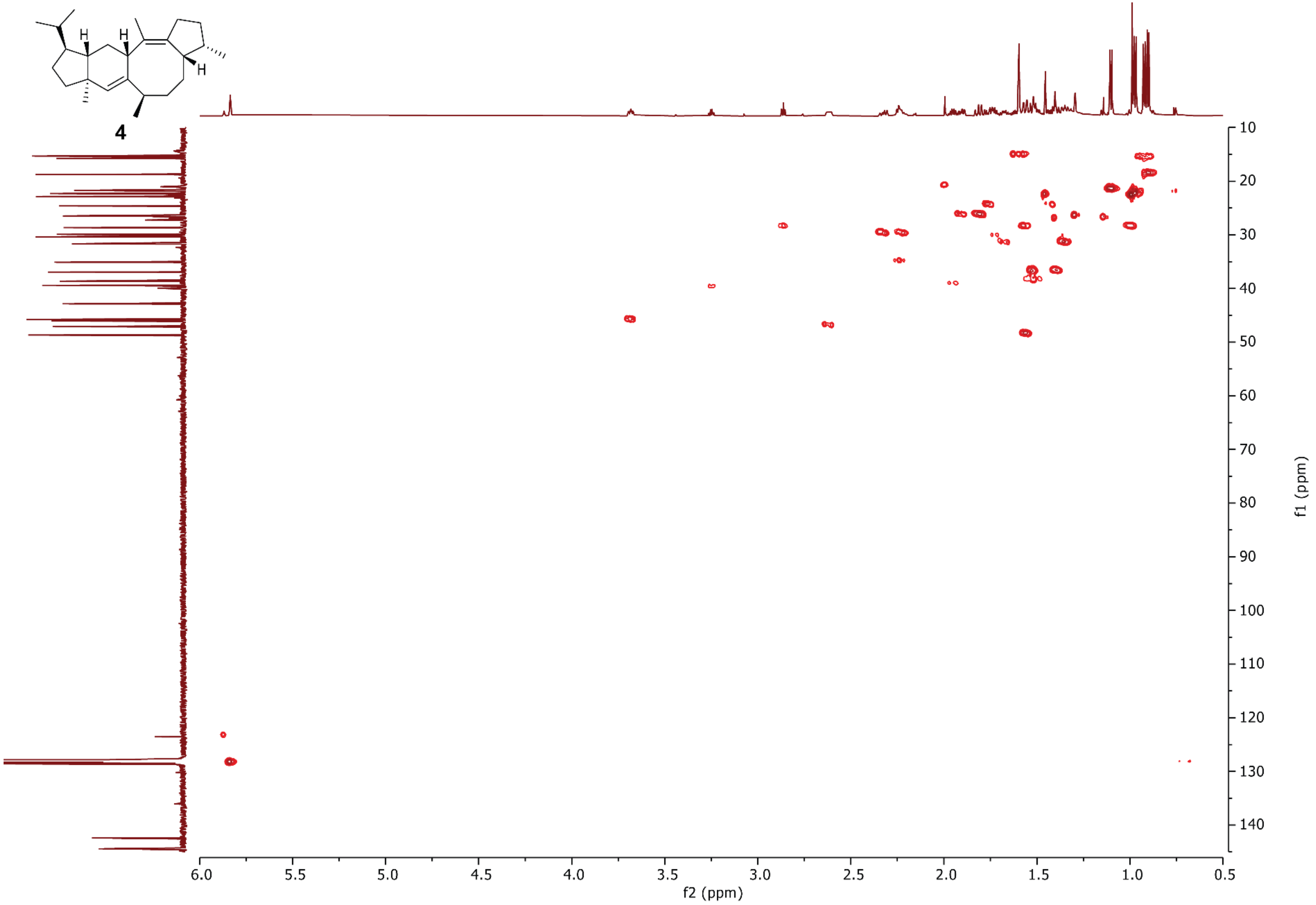

Figure S40. HMQC spectrum of **4** (C_6D_6).

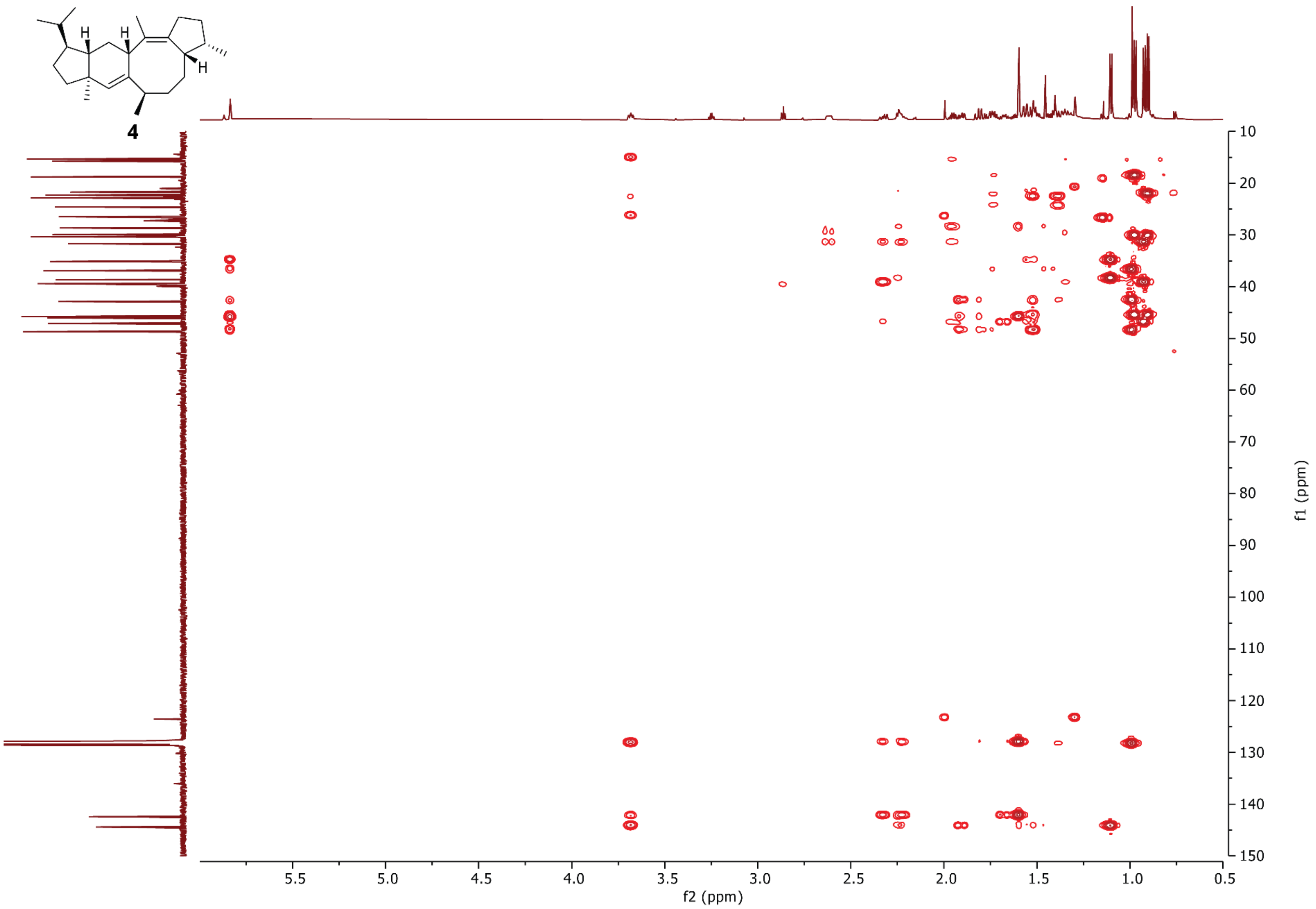

Figure S41. HMBC spectrum of **4** (C_6D_6).

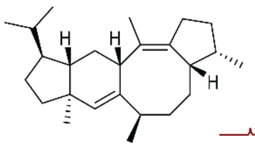

4

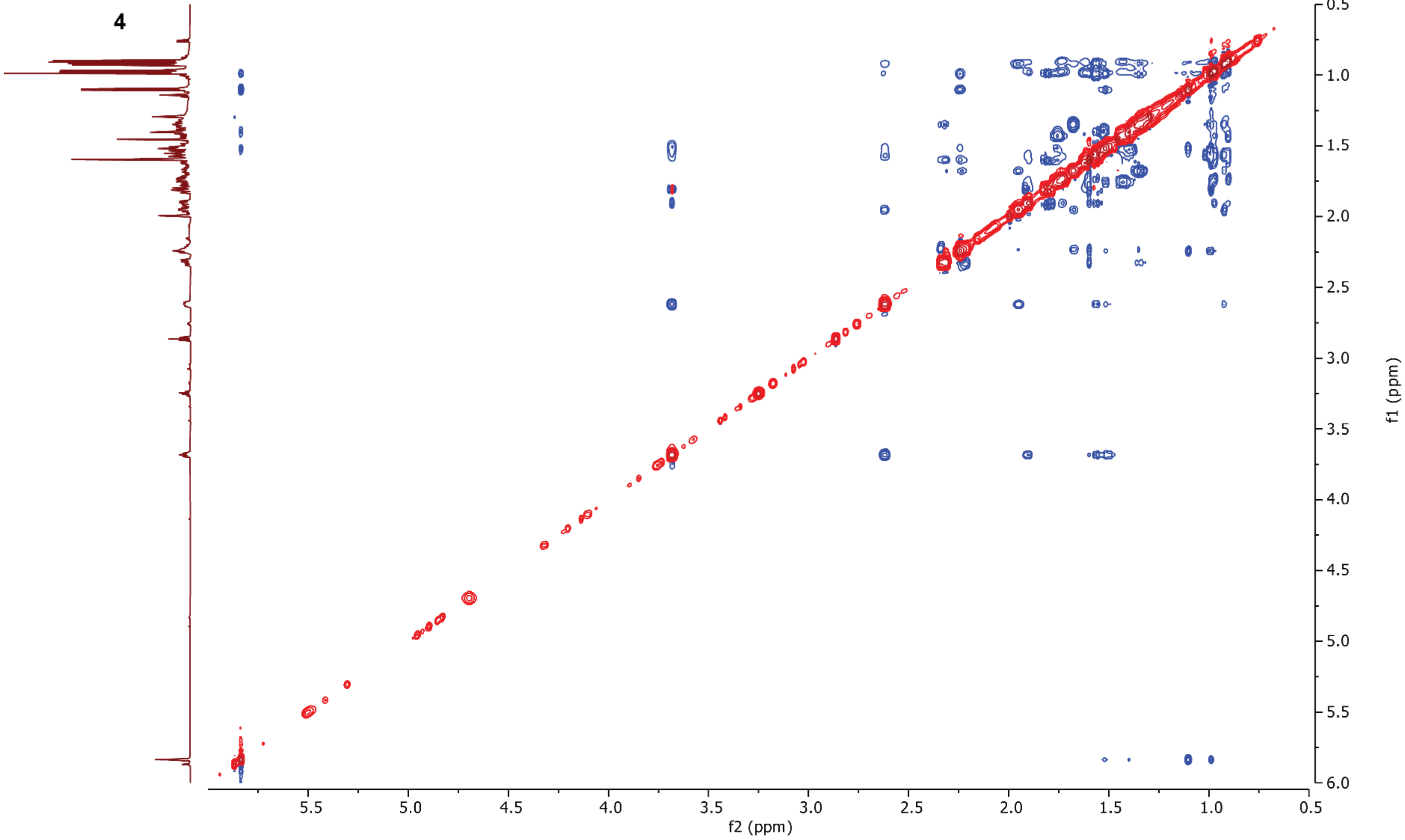

Figure S42. NOESY spectrum of **4** (700 MHz, C_6D_6).

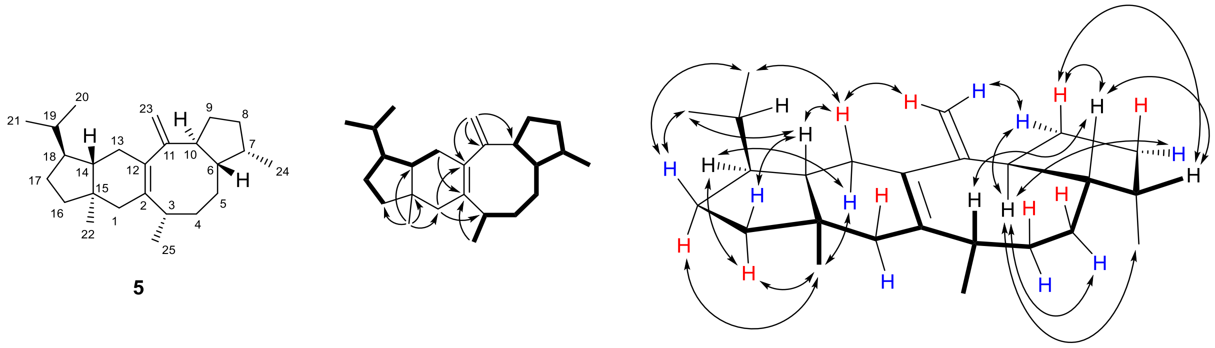

Figure S43. Structure elucidation of **5**. Bold: ^{1}H,^{1}H-COSY correlations, single-headed arrows: key HMBC correlations, and double headed arrows: NOESY correlations.

Table S6. NMR data of aspergildiene D (**5**) in C_6D_6 recorded at 298 K.

C[a]		δ_C[b]	δ_H[b]	δ_C'[d]	δ_H'[d]
1	CH_2	38.7 (br)	1.85 (m, 2H)	38.3	1.79 (m, 2H)
2	C_q	130.8 (br)	–	130.6	–
3	CH	33.5 (br)	2.97 (m, 1H)	33.3	2.77 (m, 1H)
4	CH_2	35.1 (br)	1.26 (m, 1H, H_β)[d]<br>1.43 (m, 1H, H_α)[d]	34.9	1.23 (m, 1H)<br>1.36 (m, 1H)
5	CH_2	27.9 (br)	1.36 (m, 1H, H_α)<br>1.61 (m, 1H, H_β)	27.5	1.23 (m, 1H)<br>1.57 (m, 1H)
6	CH	50.8 (br)	1.40 (m, 1H)	50.7	1.40 (m, 1H)
7	CH	39.6	2.02 (m, 1H)	39.4	2.08 (m, 1H)
8	CH_2	31.2 (br)	1.23 (m, 1H, H_α)<br>1.85 (m, 1H, H_β)	31.0	1.22 (m, 1H)<br>1.86 (m, 1H)
9	CH_2	28.4 (br)	1.79 (m, 2H)	28.1	1.71 (m, 2H)
10	CH	49.3	2.20 (m, 1H)	48.9	2.08 (m, 1H)
11	C_q	157.0	–	157.2	–
12	C_q	135.8 (br)	–	135.4	–
13	CH_2	35.0 (br)	2.01 (m, 1H, H_α)<br>2.28 (dd, $^3J_{H,H}$ = 17.1, 5.3, 1H, H_β)	34.7	1.90 (m, 1H)<br>2.06 (m, 1H)
14	CH	47.0	1.44 (m, 1H)	46.6	1.34 (m, 1H)
15	C_q	41.3	–	41.1	–
16	CH_2	39.6	1.23 (m, 1H, H_β)<br>1.56 (m, 1H, H_α)	39.4	1.20 (m, 1H)<br>1.53 (m, 1H)
17	CH_2	25.4	1.39 (m, 1H, H_β)<br>1.76 (m, 1H, H_α)	25.1	1.37 (m, 1H)<br>1.75 (m, 1H)
18	CH	48.2	1.60 (m, 1H)	47.9	1.51 (m, 1H)
19	CH	31.3	1.60 (m, 1H)	30.9	1.62 (m, 1H)
20	CH_3	22.1	0.94 (d, $^3J_{H,H}$ = 6.8, 3H)	22.0	0.90 (d, J = 6.8, 3H)
21	CH_3	19.5	0.86 (d, $^3J_{H,H}$ = 6.7, 3H)	19.3	0.84 (d, J = 6.7, 3H)
22	CH_3	18.8	0.80 (br s, 3H)	18.7	0.72 (s, 3H)
23	CH_2	108.6	4.90 (br, 1H)<br>5.16 (br, 1H)	108.1	4.67 (t, J = 1.4, 1H)<br>5.01 (t, J = 1.4, 1H)
24	CH_3	17.0	0.83 (d, $^3J_{H,H}$ = 7.2, 3H)	16.9	0.81 (d, J = 7.1, 3H)
25	CH_3	18.5 (br)	1.01 (d, $^3J_{H,H}$ = 6.9, 3H)	18.3	0.89 (d, J = 6.8, 3H)

[a] Carbon numbering indicating the origin for each carbon from GFPP by same number and colour code for diastereotopic hydrogens as shown in Figure S43. [b] Chemical shifts δ in ppm, multiplicity: s = singlet, d = doublet, m = multiplet, br = broad. Coupling constants J are given in Hertz. Their assignment is based on the HMQC spectrum. [c] Chemical shifts δ in ppm reported previously (recorded in $CDCl_3$).[12] [d] Assignment of diastereotopic hydrogens based on labelling experiments (Figure S63).

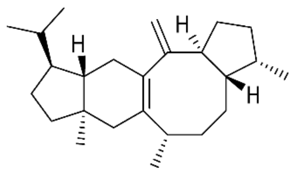

5

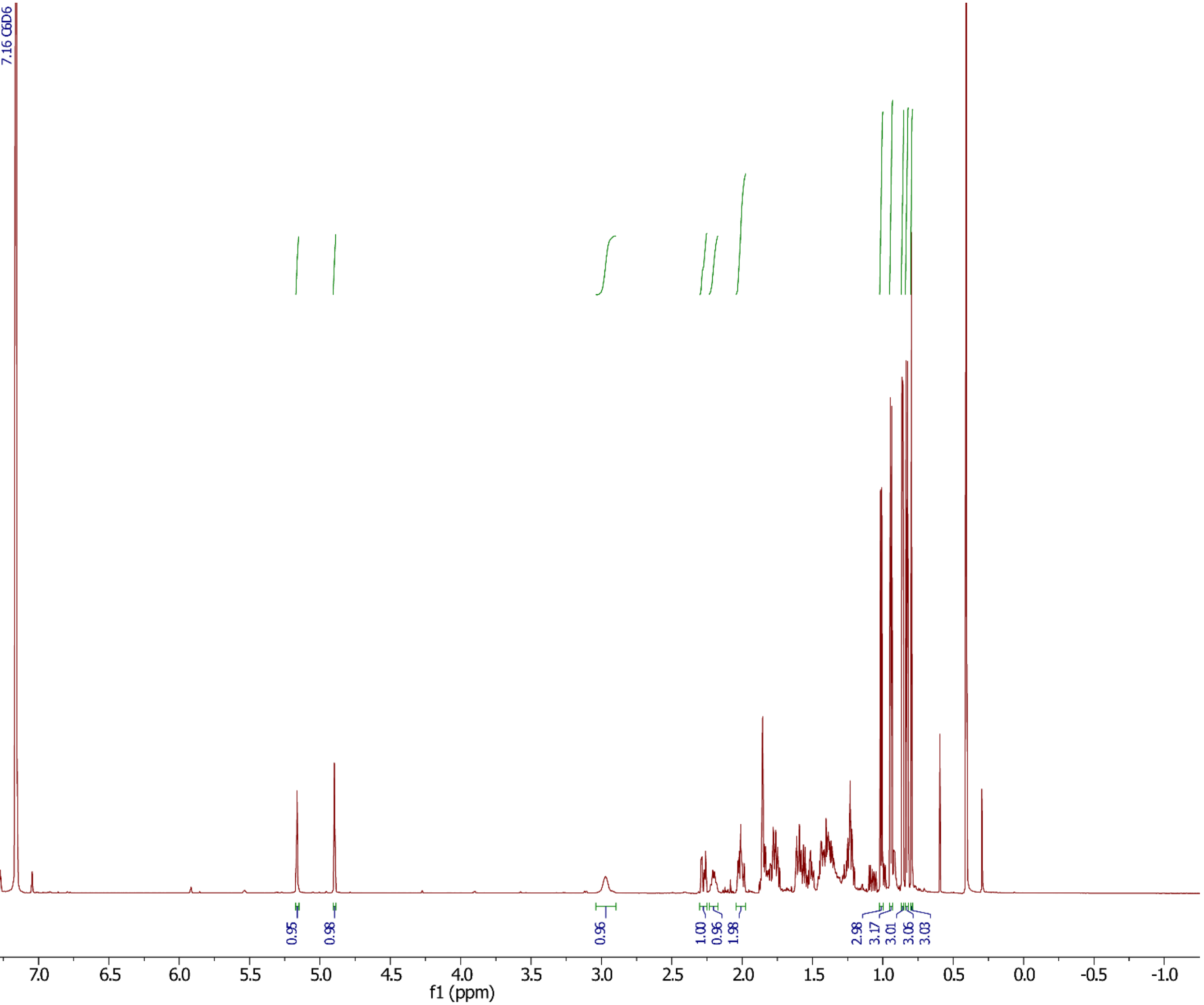

Figure S44. ^{1}H-NMR spectrum of **5** (700 MHz, C_6D_6).

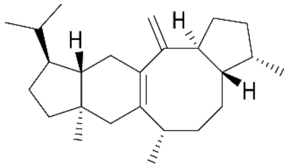

5

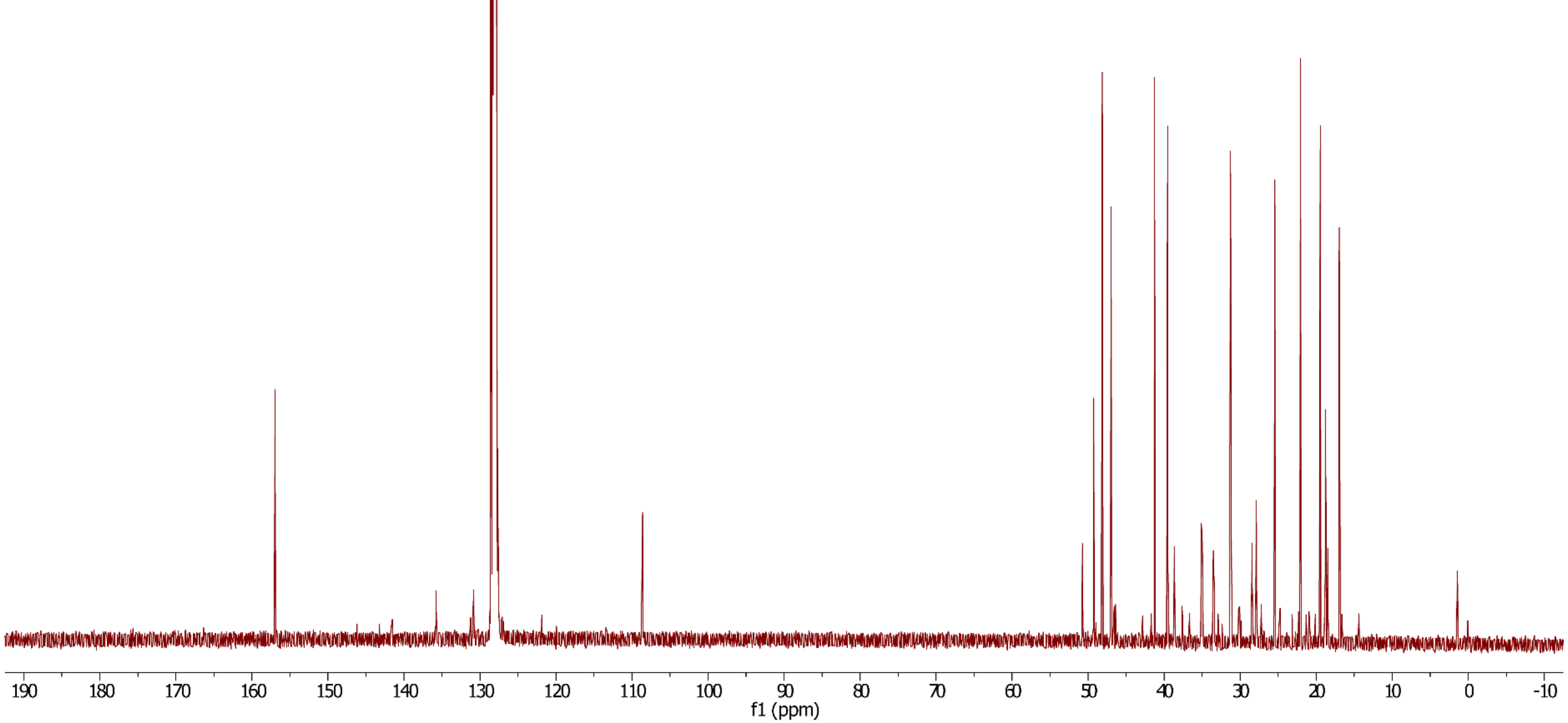

Figure S45. ^{13}C-NMR spectrum of **5** (176 MHz, C_6D_6).

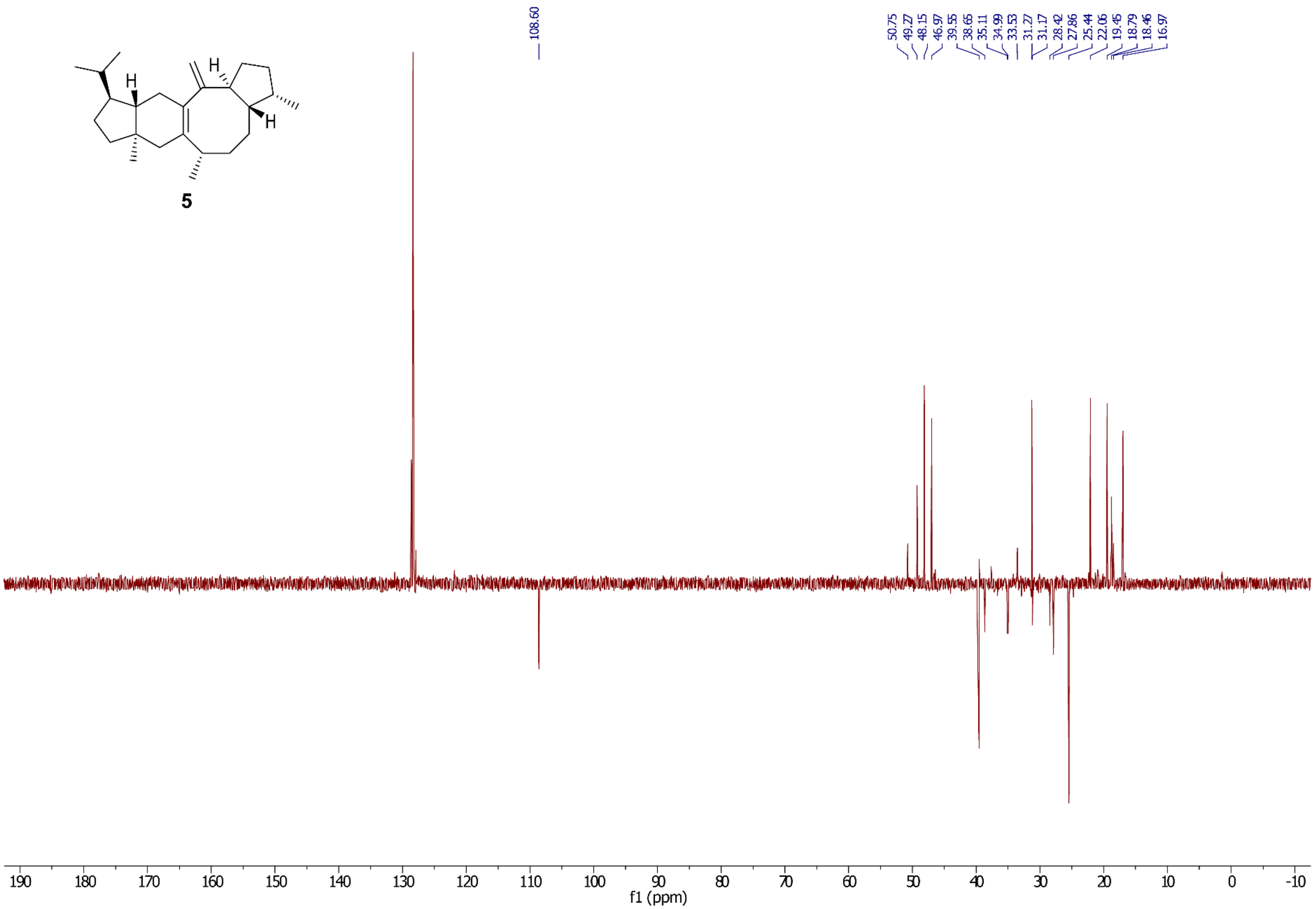

Figure S46. DEPT spectrum of **5** (176 MHz, C_6D_6).

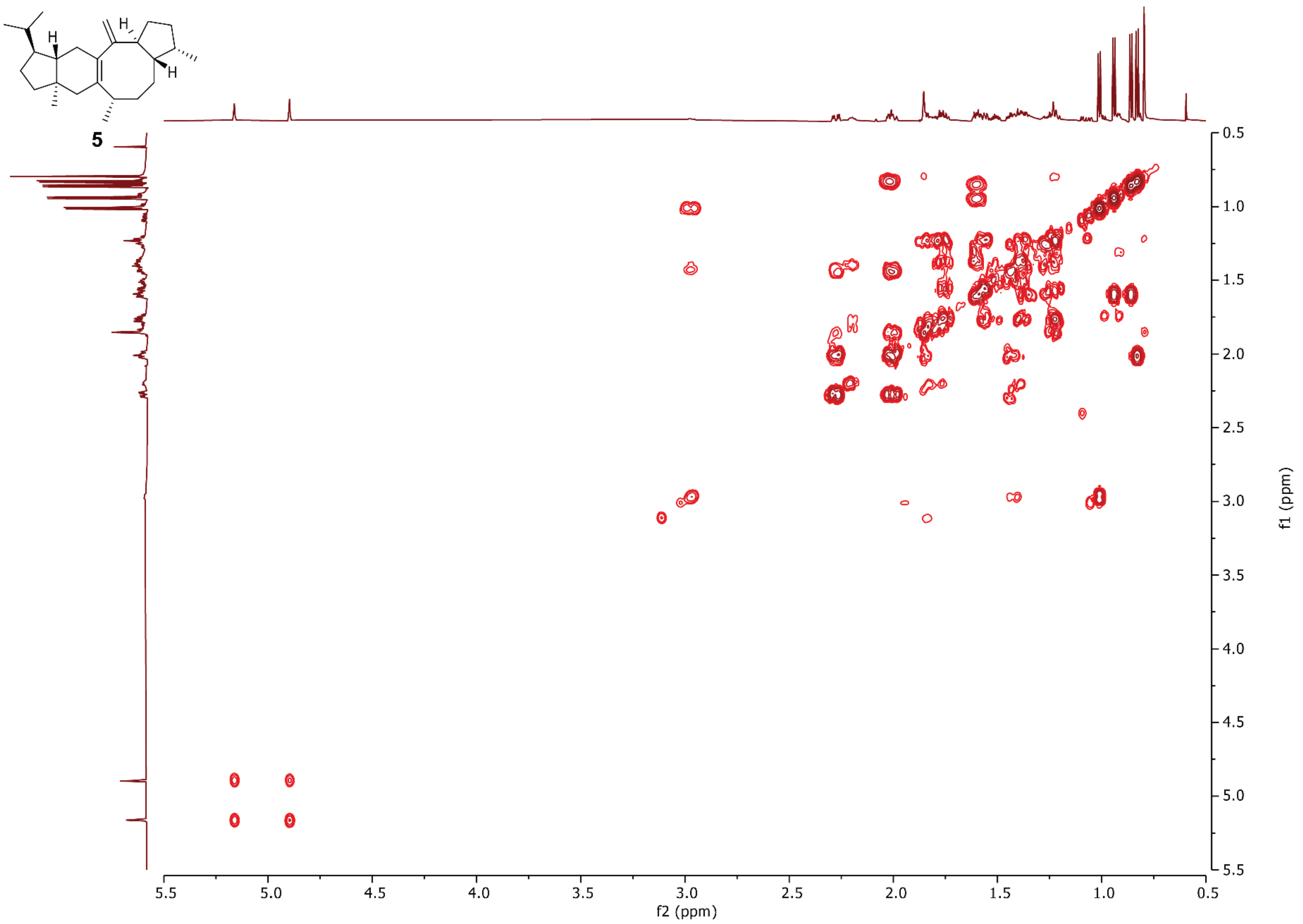

Figure S47. $^{1}H,^{1}H$-COSY spectrum of **5** (700 MHz, C_6D_6).

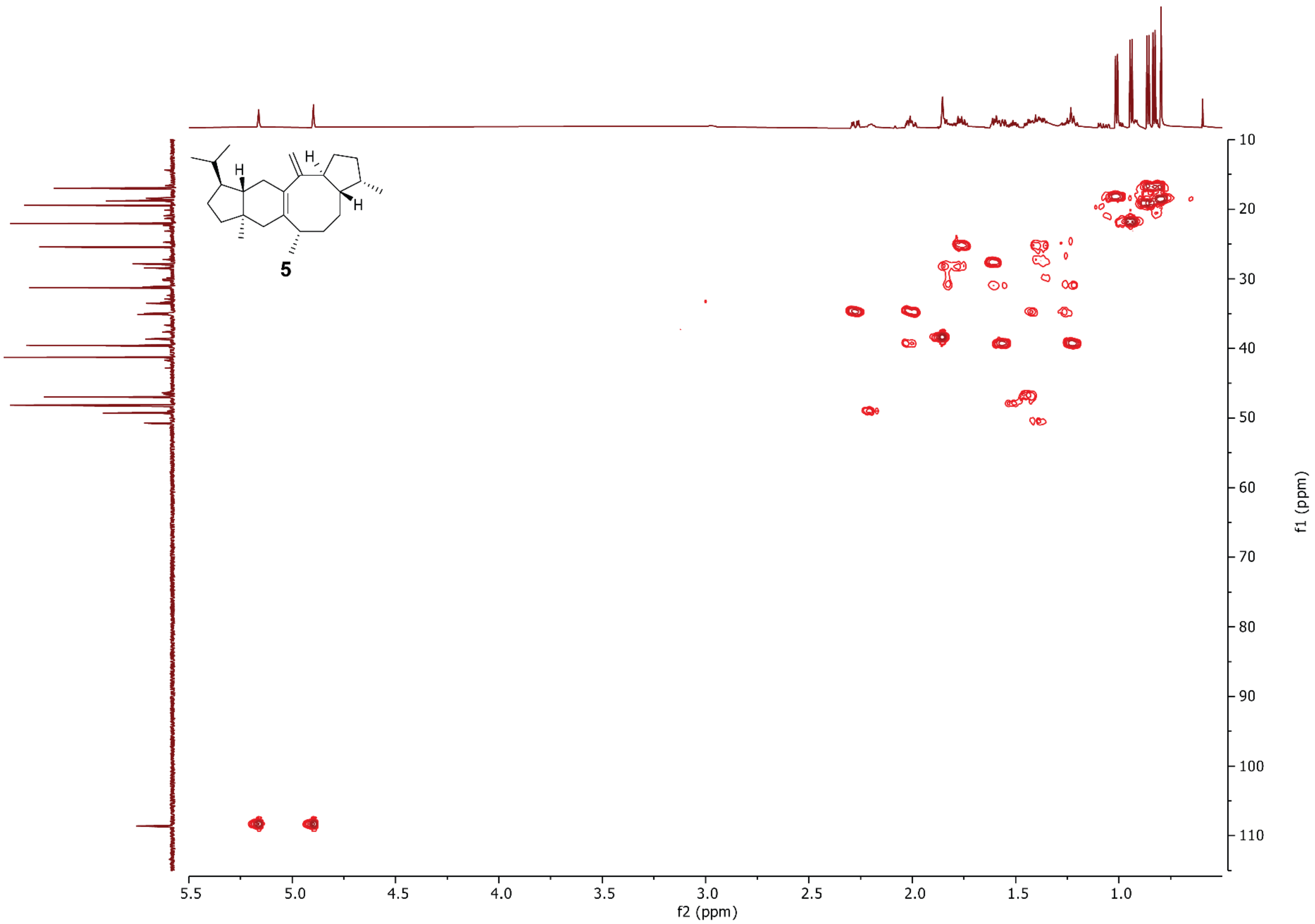

Figure S48. HMQC spectrum of **5** (C_6D_6).

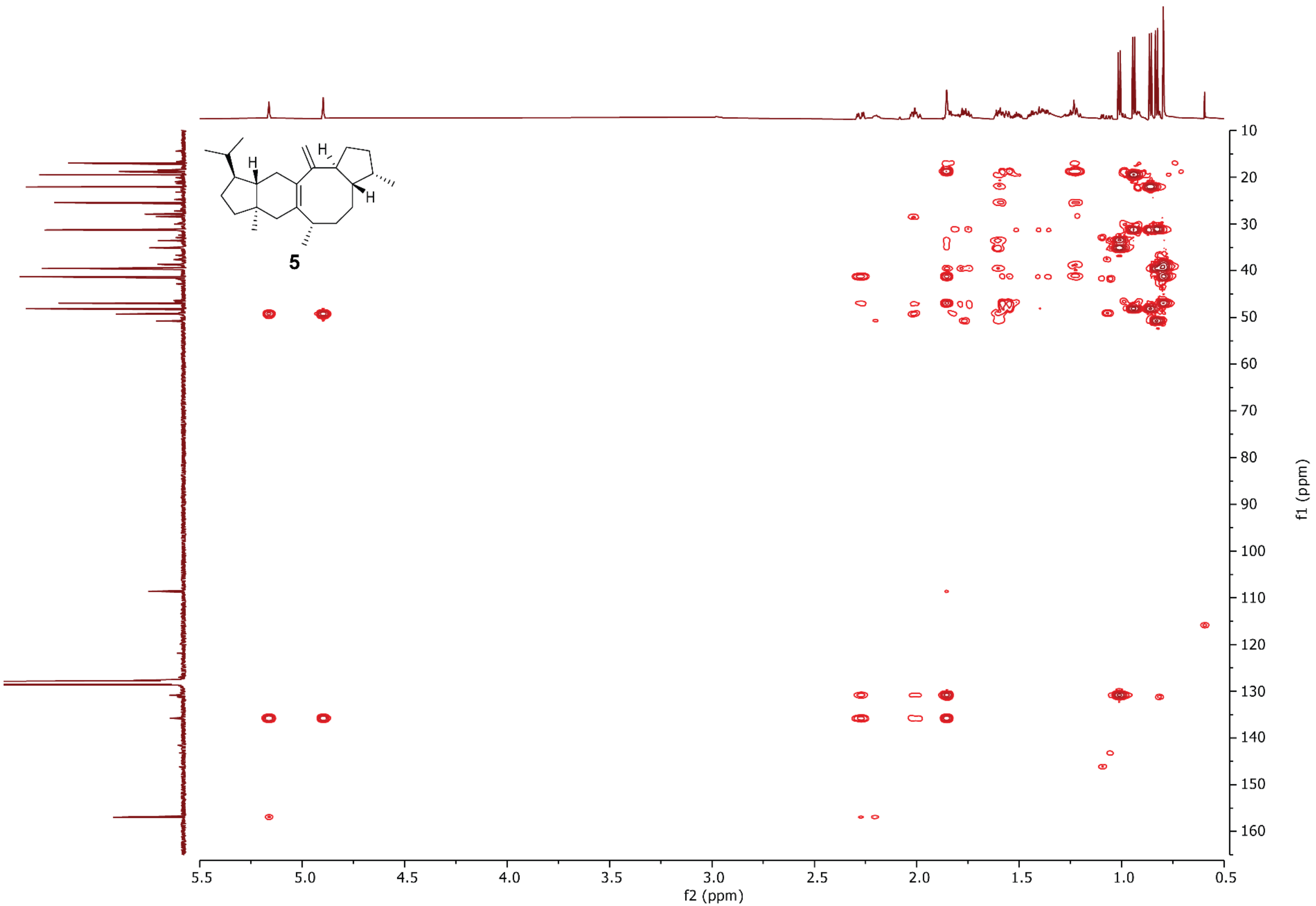

Figure S49. HMBC spectrum of **5** (C_6D_6).

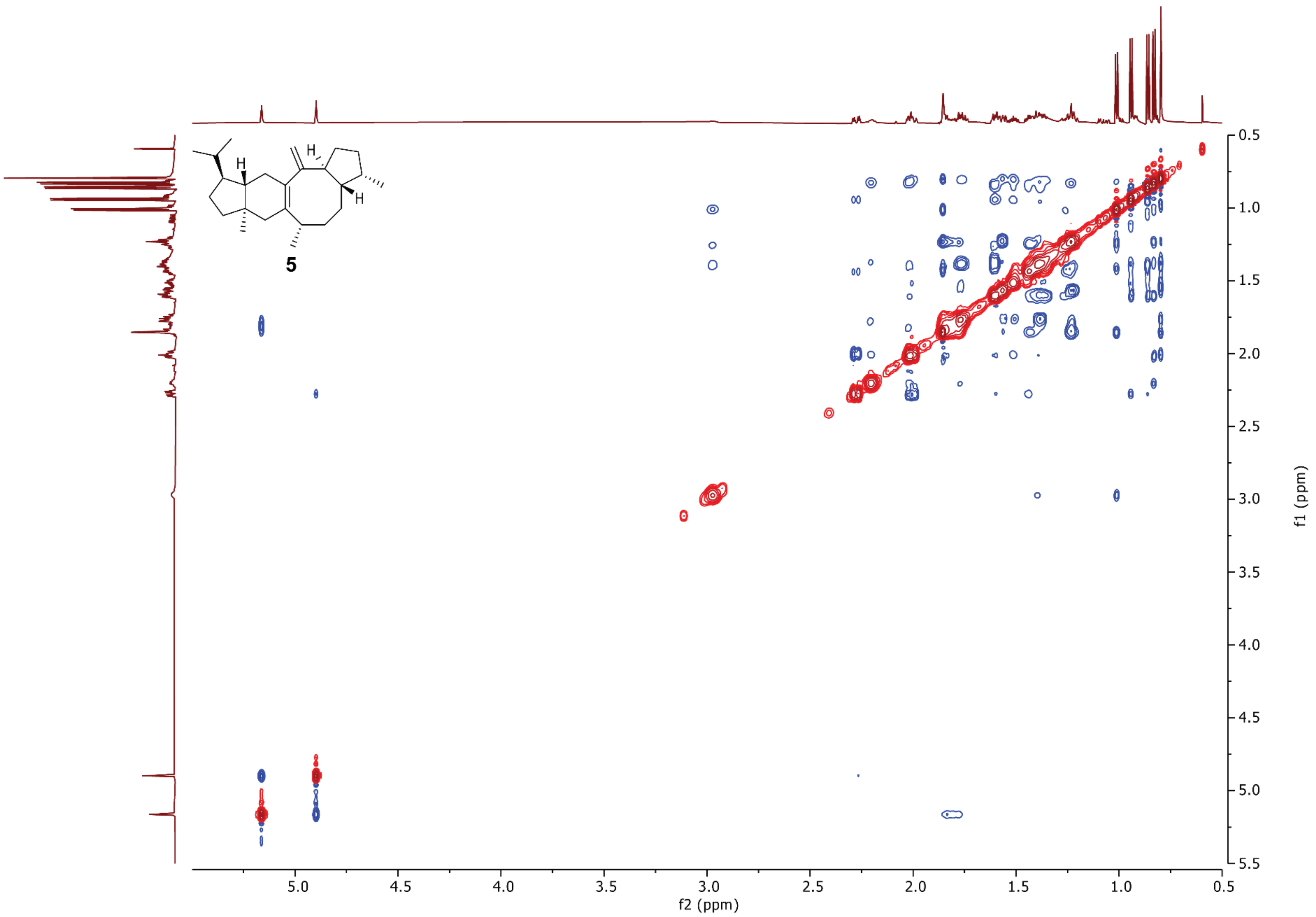

Figure S50. NOESY spectrum of **5** (700 MHz, C_6D_6).

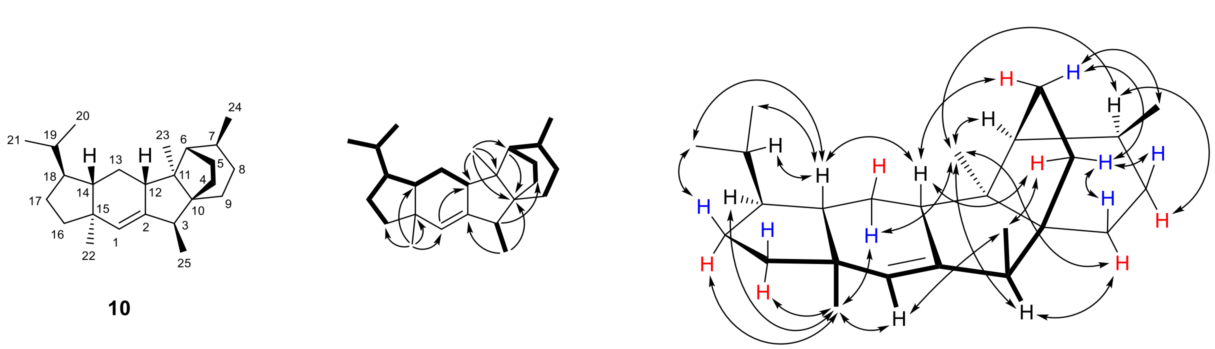

Figure S51. Structure elucidation of **10**. Bold: ^{1}H,^{1}H-COSY correlations, single-headed arrows: key HMBC correlations, and double headed arrows: NOESY correlations.

Table S7. NMR data of calidoustene (**10**) in C_6D_6 recorded at 298 K.

C		δ_C[a]	δ_H[a]
1	CH	129.2	5.69 (t, $J_{H,H}$ = 2.6, 1H)
2	C_q	147.8	–
3	CH	42.4	2.06 (m, 1H)
4	CH_2	30.6	1.08 (m, 1H, H)<br>1.65 (m, 1H, H)
5	CH_2	20.7	1.41 (m, 1H, H)<br>1.57 (m, 1H, H)
6	CH	48.0	1.70 (m, 1H)
7	CH	29.9	1.90 (m, 1H)
8	CH_2	27.6	0.97 (m, 1H, H_β)<br>1.39 (m, 1H, H_α)
9	CH_2	31.0	1.27 (m, 1H, H_β)<br>1.32 (m, 1H, H_α)
10	C_q	51.9	–
11	C_q	52.6	–
12	CH	50.8	2.56 (m, 1H)
13	CH_2	22.6	1.27 (m, 1H, H_α)<br>1.66 (m, 1H, H_β)
14	CH	49.9	1.64 (m, 1H)
15	C_q	44.9	–
16	CH_2	36.1	1.42 (m, 1H, H_β)<br>1.51 (m, 1H, H_α)
17	CH_2	24.2	1.43 (m, 1H, H_β)<br>1.74 (m, 1H, H_α)
18	CH	44.8	1.64 (m, 1H)
19	CH	29.8	1.78 (m, 1H)
20	CH_3	22.3	0.99 (d, $^3J_{H,H}$ = 6.8, 3H)
21	CH_3	18.2	0.87 (d, $^3J_{H,H}$ = 6.8, 3H)
22	CH_3	23.6	0.90 (s, 3H)
23	CH_3	13.3	0.81 (s, 3H)
24	CH_3	20.6	0.83 (d, $^3J_{H,H}$ = 6.6, 3H)
25	CH_3	14.4	1.06 (d, $^3J_{H,H}$ = 7.1, 3H)

[a] Carbon numbering indicating the origin for each carbon from GFPP by same number and colour code for diastereotopic hydrogens as shown in Figure S51. [b] Chemical shifts δ in ppm, multiplicity: s = singlet, d = doublet, t = triplet, m = multiplet. Coupling constants J are given in Hertz.

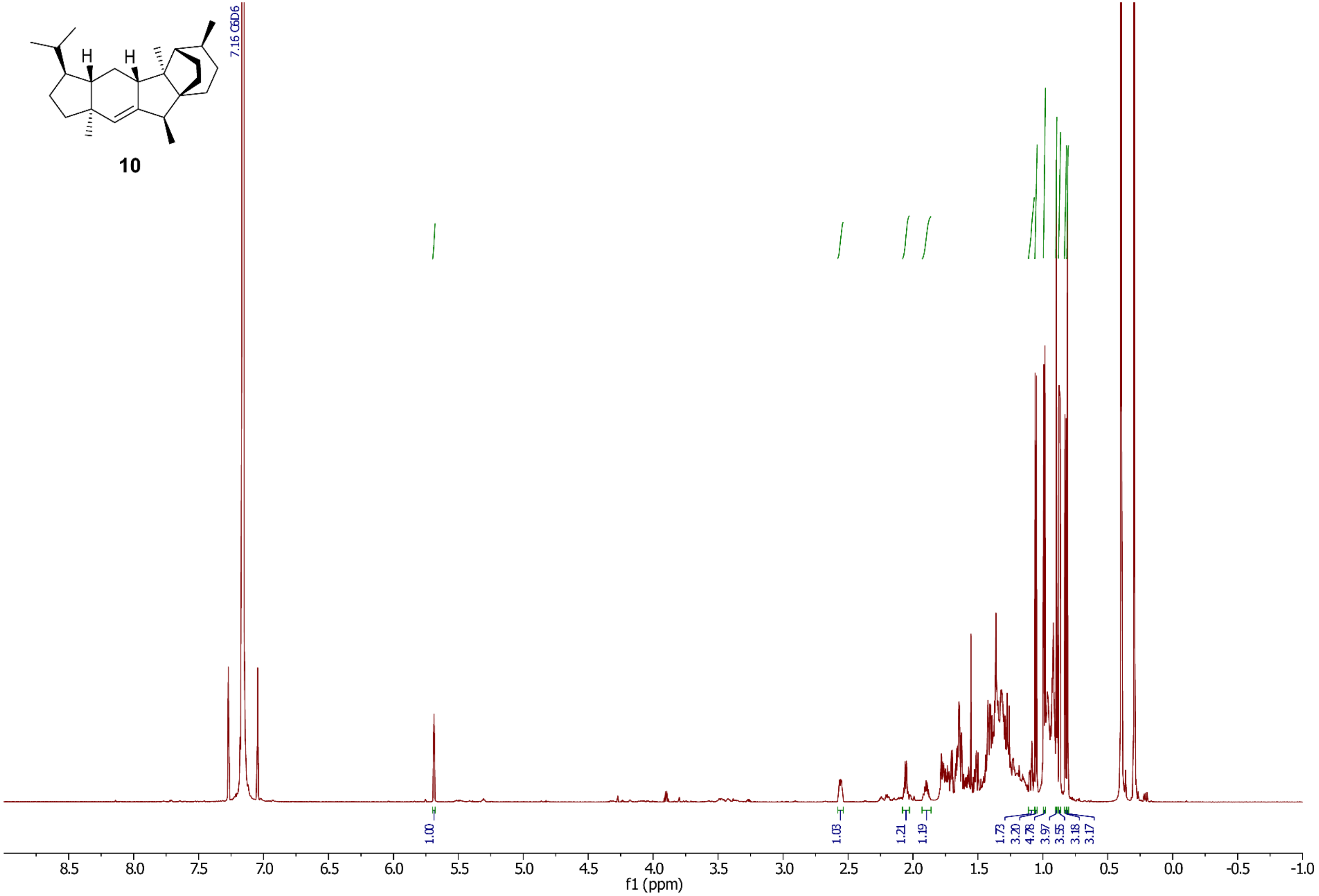

Figure S52. ^{1}H-NMR spectrum of **10** (700 MHz, C_6D_6).

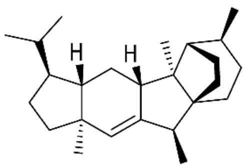

10

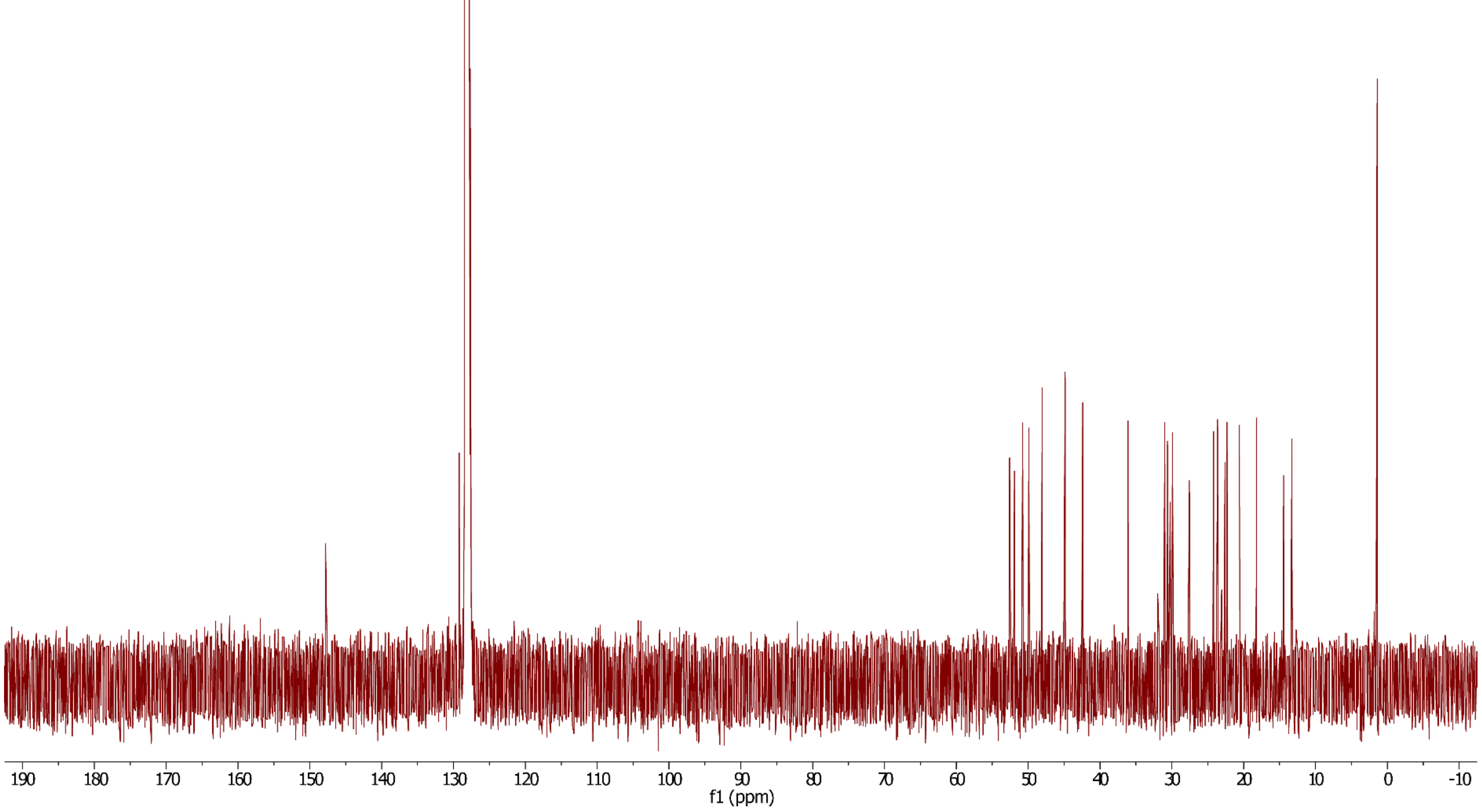

Figure S53. ^{13}C-NMR spectrum of **10** (176 MHz, C_6D_6).

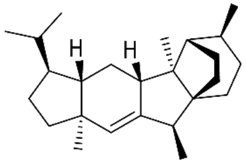

10

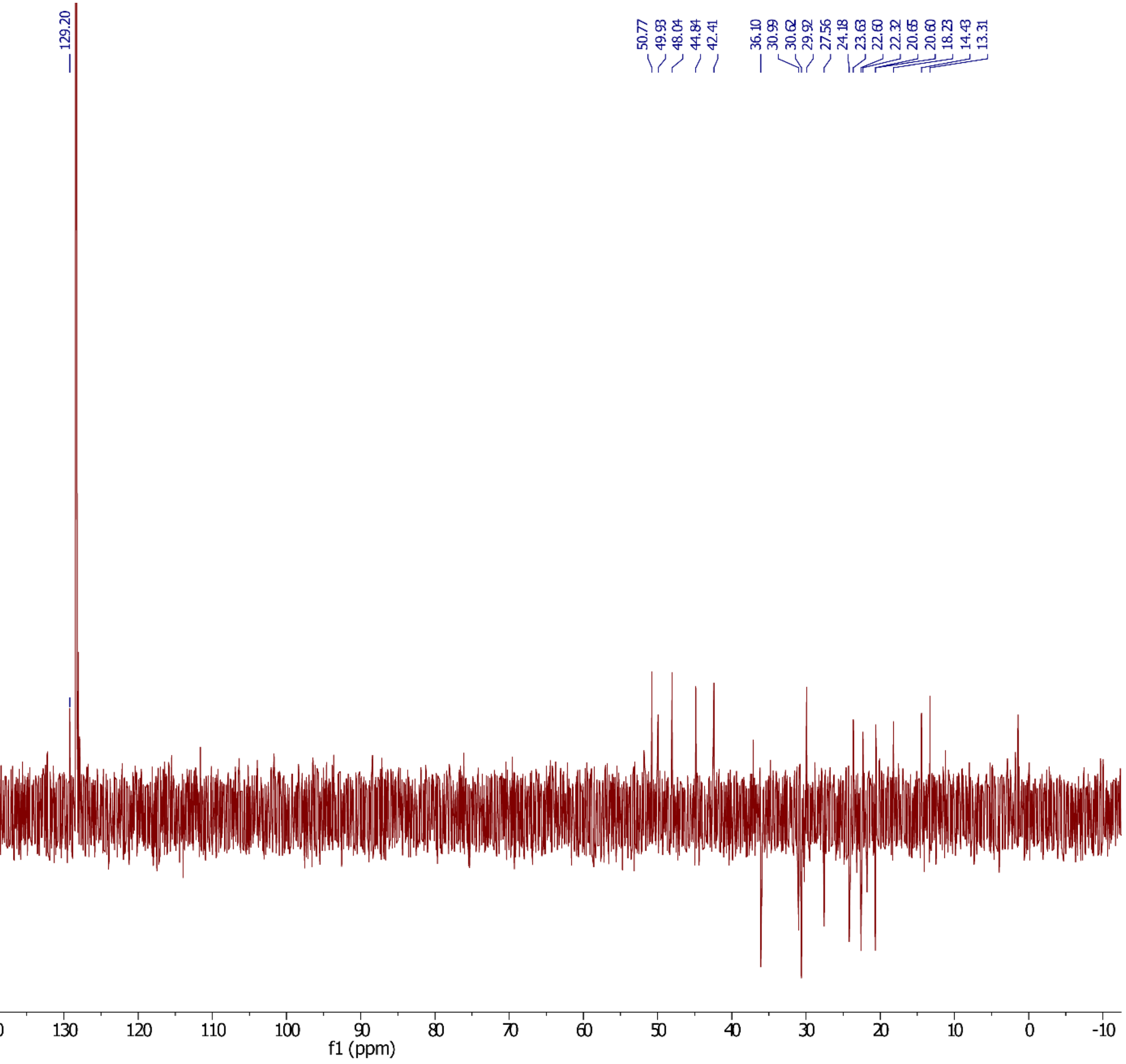

Figure S54. DEPT spectrum of **10** (176 MHz, C_6D_6).

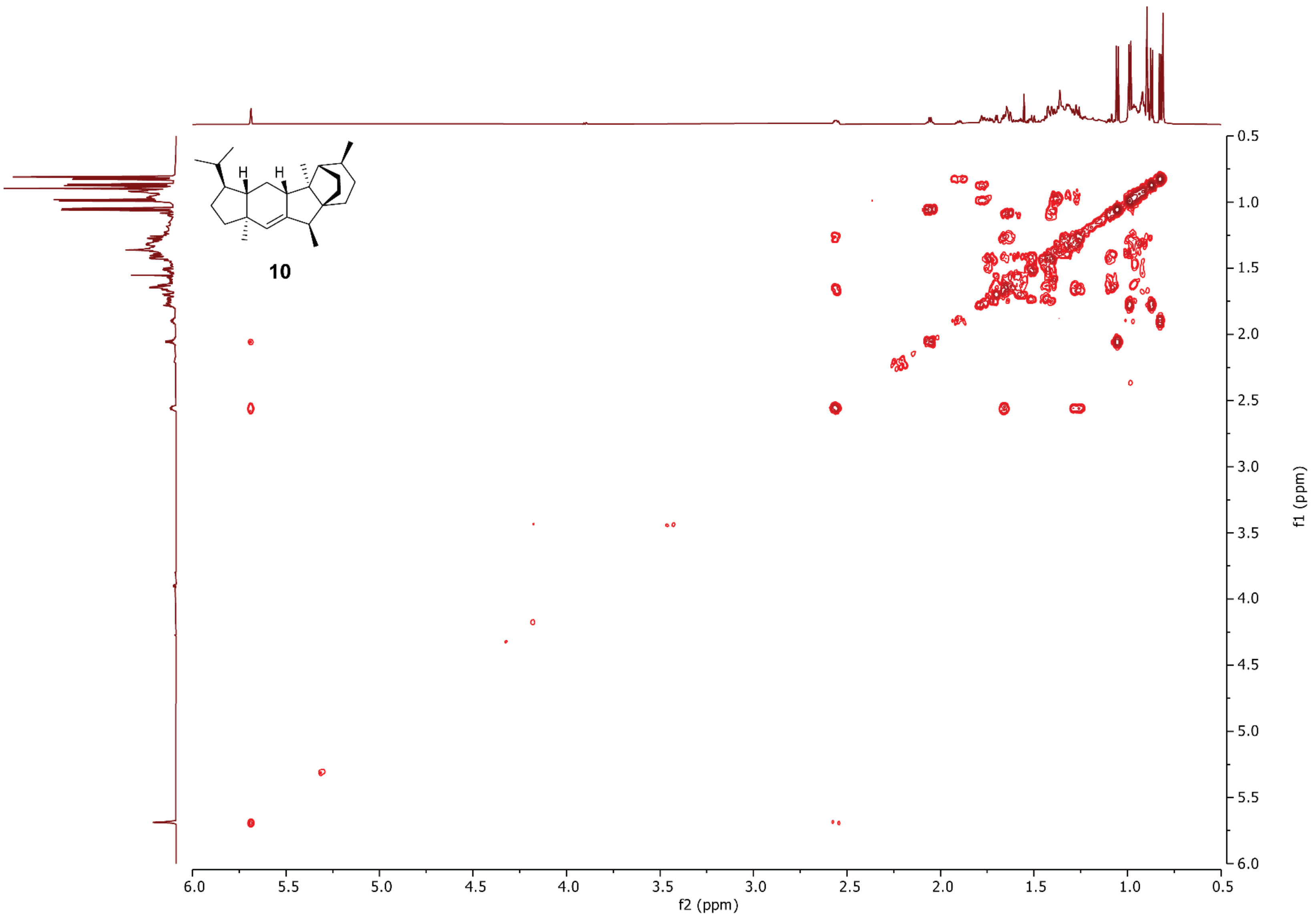

Figure S55. $^1H,^1H$-COSY spectrum of **10** (700 MHz, C_6D_6).

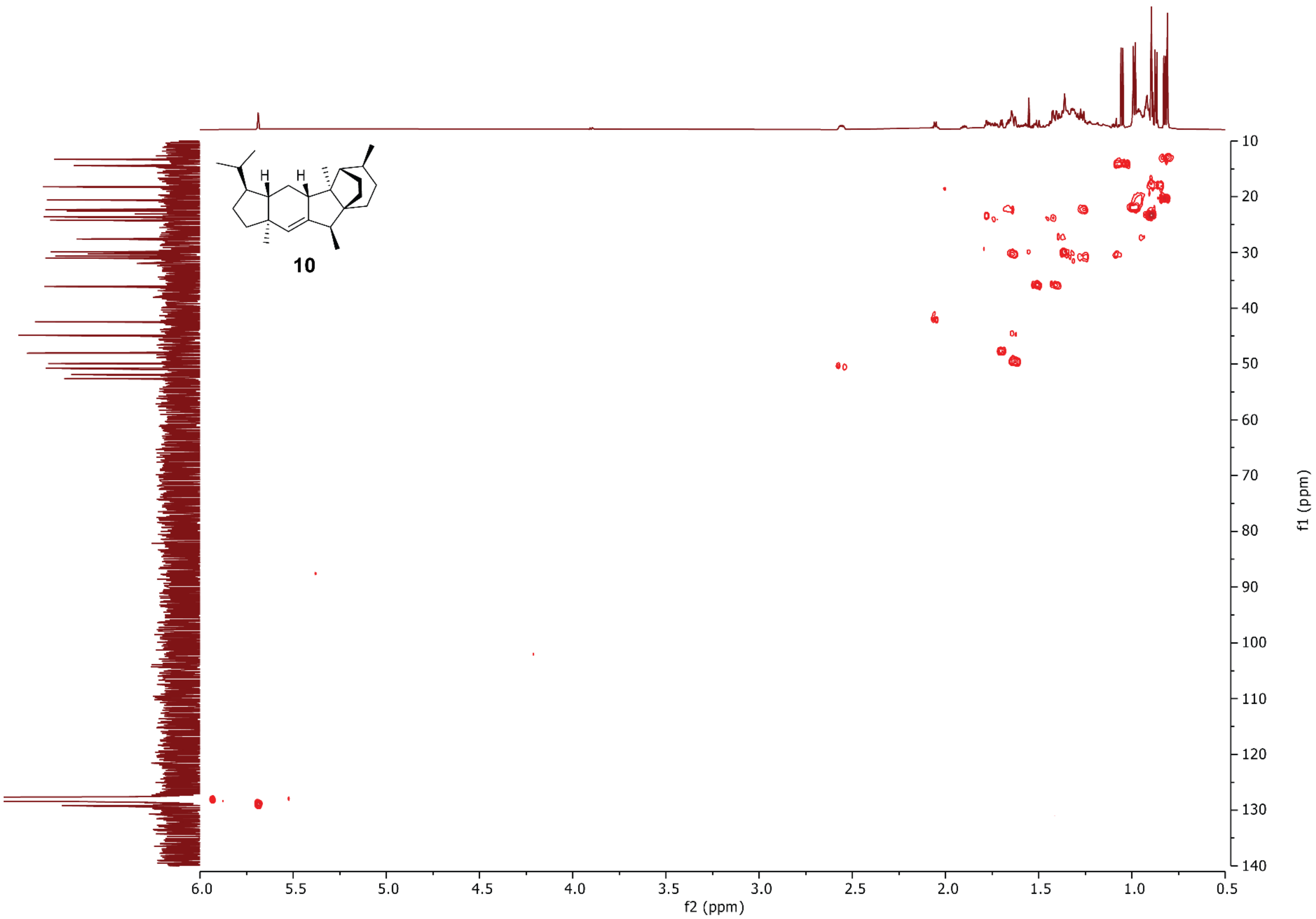

Figure S56. HMQC spectrum of **10** (C_6D_6).

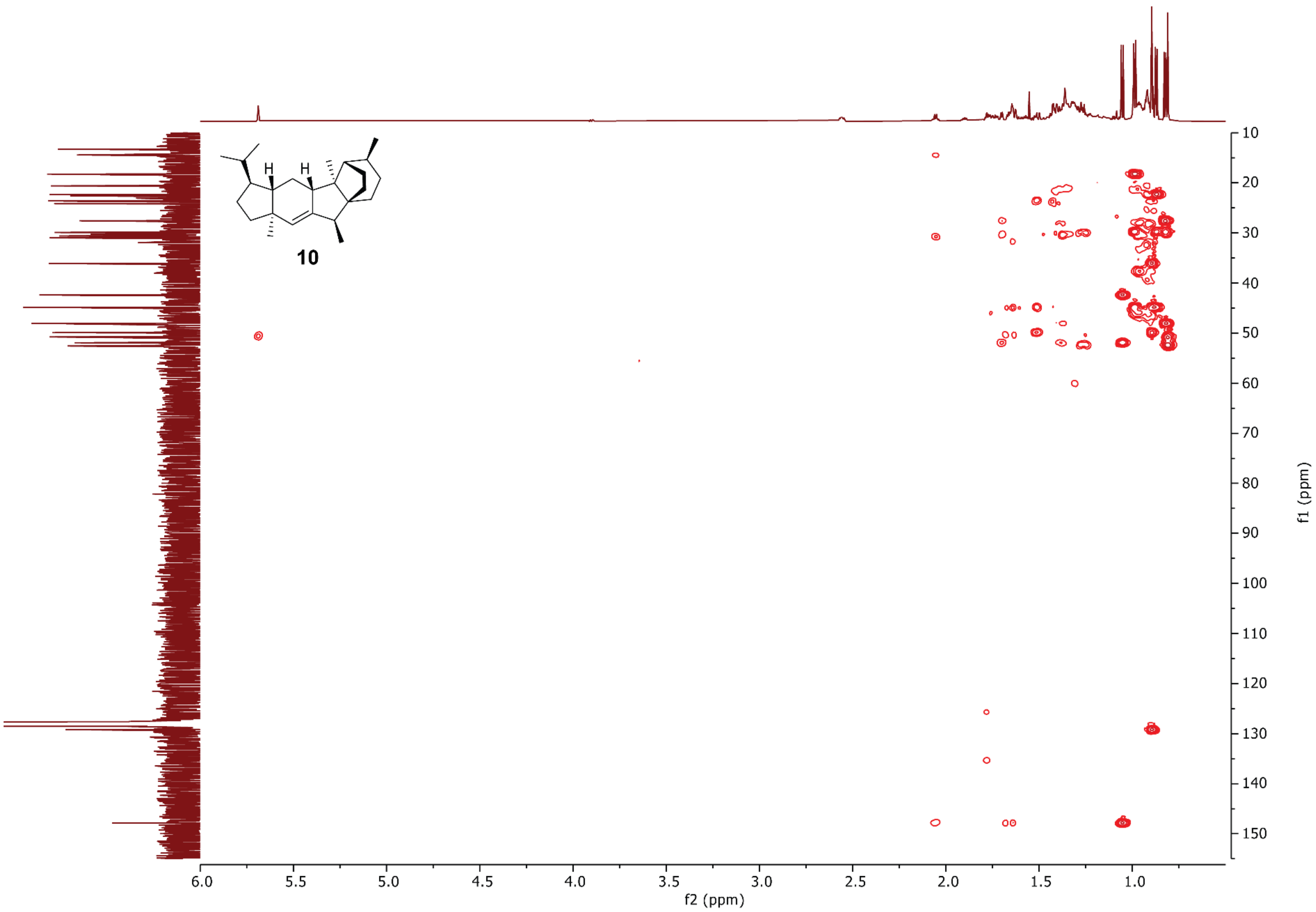

Figure S57. HMBC spectrum of **10** (C_6D_6).

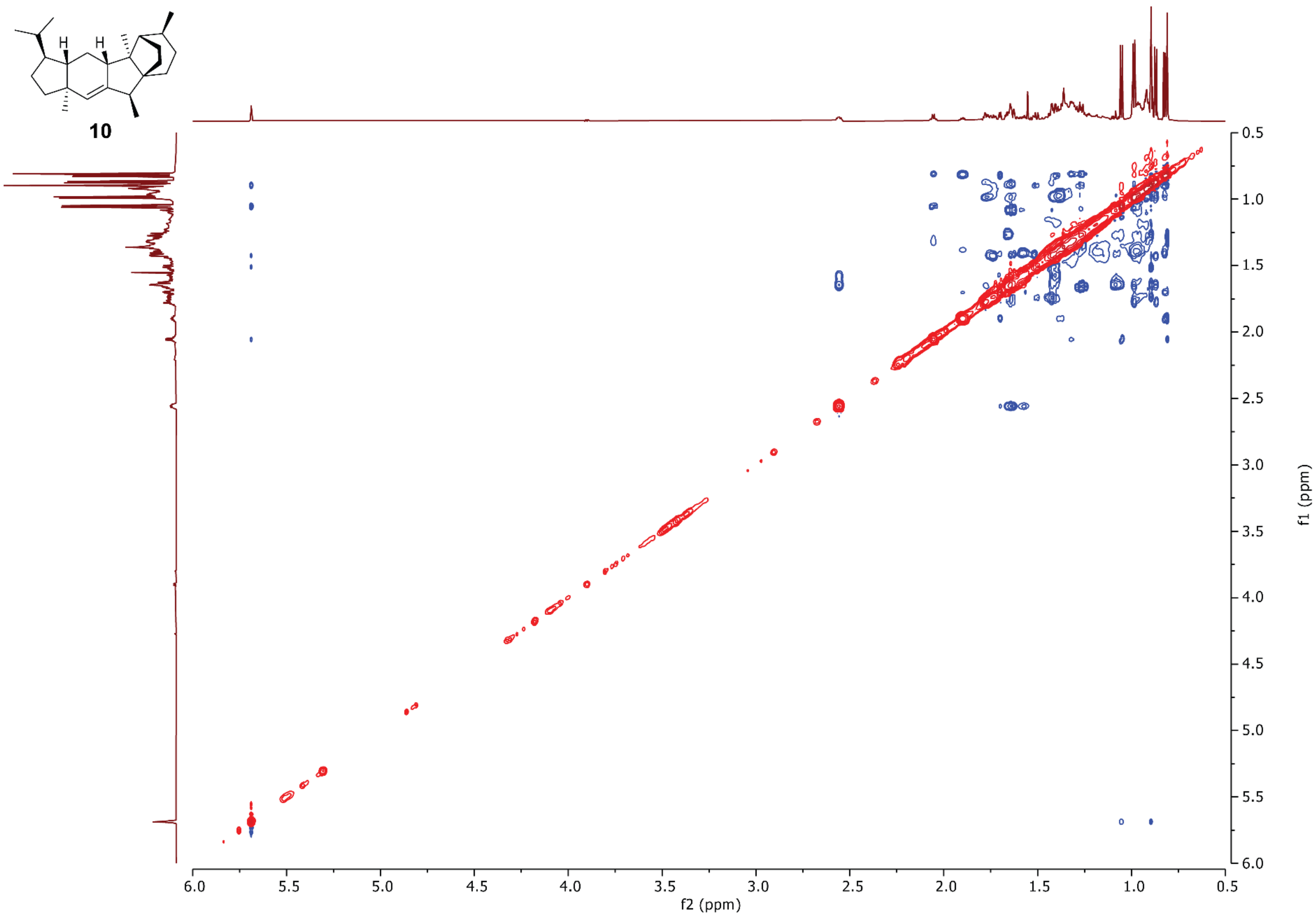

Figure S58. NOESY spectrum of **10** (700 MHz, C_6D_6).

Incubation experiments with labelled substrates

Isotopic labelling experiments were performed with substrates (each ca. 0.5 mg) and enzyme preparations as listed in Table S8. For enzyme preparations and incubation conditions the same protocol as mentioned above for small scale reactions was used (pages 9 and 15). If necessary, isopentenyl diphosphate isomerase (IDI)[23] was added with a final concentration of 0.1 mg mL^{-1}. In the cases of low production of minor products, multiple trials were carried out to accumulate enough amounts of products for NMR analysis. After overnight incubation, the reaction mixtures were extracted three times with benzene and analysed by GC/MS. The extracts were then concentrated to dryness with a stream of Argon. C_6D_6 (0.5 mL) was added to the dried extract and the samples were subjected to NMR analyses.

Table S8. Labelling experiments with AcAS.

entry	substrates	enzymes	results shown in
1	DMAPP + (*E*)-(4-^{13}C,4-^{2}H)IPP[24]	AcIdOS-PT, AcAS-TC	Figures S59 – S64
2	DMAPP + (*Z*)-(4-^{13}C,4-^{2}H)IPP[24]	AcIdOS-PT, AcAS-TC	Figures S59 – S64
3	FPP + (*R*)-(1-^{13}C,1-^{2}H)IPP[25]	AcAS	Figures S65 – S70
4	FPP + (*S*)-(1-^{13}C,1-^{2}H)IPP[25]	AcAS	Figures S65 – S70
5	(*R*)-(1-^{13}C,1-^{2}H)FPP[26] + IPP	AcAS	Figures S65 – S70
6	(*S*)-(1-^{13}C,1-^{2}H)FPP[26] + IPP	AcAS	Figures S65 – S70
7	GGPP + (1-^{13}C)IPP[26]	AcAS	Figure S71
8	GGPP + (2-^{13}C)IPP[27]	AcAS	Figure S72
9	GGPP + (3-^{13}C)IPP[26]	AcAS	Figures S73, S103 – S105
10	GGPP + (4-^{13}C)IPP[26]	AcAS	Figure S74
11	(1-^{13}C)GGPP + IPP[8]	AcAS	Figure S75
12	(2-^{13}C)GGPP + IPP[26]	AcAS	Figures S76, S107
13	(3-^{13}C)GGPP + IPP[8]	AcAS	Figures S77, S100, S101
14	(4-^{13}C)GGPP + IPP[8]	AcAS	Figure S78
15	(1-^{13}C)FPP[28] + IPP	AcAS	Figure S79
16	(2-^{13}C)FPP[28] + IPP	AcAS	Figure S80
17	(3-^{13}C)FPP[28] + IPP	AcAS	Figure S81
18	(4-^{13}C)FPP[28] + IPP	AcAS	Figure S82
19	(5-^{13}C)FPP[28] + IPP	AcAS	Figure S83
20	(6-^{13}C)FPP[28] + IPP	AcAS	Figure S84
21	(7-^{13}C)FPP[28] + IPP	AcAS	Figure S85
22	(8-^{13}C)FPP[28] + IPP	AcAS	Figure S86
23	(9-^{13}C)FPP[28] + IPP	AcAS	Figure S87
24	(10-^{13}C)FPP[28] + IPP	AcAS	Figure S88
25	(11-^{13}C)FPP[28] + IPP	AcAS	Figure S89
26	(12-^{13}C)FPP[28] + IPP	AcAS	Figure S90
27	(9-^{13}C)GPP + IPP	AcAS	Figure S91
28	(14-^{13}C)FPP[28] + IPP	AcAS	Figure S92
29	(15-^{13}C)FPP[28] + IPP	AcAS	Figure S93
30	(20-^{13}C)GGPP[25] + IPP	AcAS	Figure S94
31	GGPP + (5-^{13}C)IPP[29]	AcAS	Figure S95
32	(7-^{13}C)GPP[5] + IPP	AcAS	Figures S96 – S99
33	(7-^{13}C)GPP + (*Z*)-(4-^{13}C,4-^{2}H)IPP	AcAS	Figures S96 – S99
34	(7-^{13}C)GPP + (*E*)-(4-^{13}C,4-^{2}H)IPP	AcAS	Figures S96 – S99

35	FPP + (3-^{13}C,2-^{2}H)DMAPP[6]	IDI, AcAS	Figures S100 – S103
36	GGPP + (3-^{13}C,2-^{2}H)DMAPP	IDI, AcAS	Figures S104, S105
37	(3-^{13}C)FPP + (2-^{2}H)DMAPP	IDI, AcAS	Figure S106
38	(2-^{2}H)FPP[25] + (2-^{13}C)IPP	AcAS	Figure S107
39	(4,4,15,15,15-^{2}H$_5$)FPP + (5-^{13}C)IPP	AcAS	Figure S111
40	8-OH-FPP + (*E*)-(4-^{13}C,4-^{2}H)IPP	AcAS	Figures S152 – 155
41	8-OH-FPP + (*Z*)-(4-^{13}C,4-^{2}H)IPP	AcAS	Figures S152 – 155
42	8-OH-FPP + (*R*)-(1-^{13}C,1-^{2}H)IPP	AcAS	Figures S156 – 159
43	8-OH-FPP + (*S*)-(1-^{13}C,1-^{2}H)IPP	AcAS	Figures S156 – 159

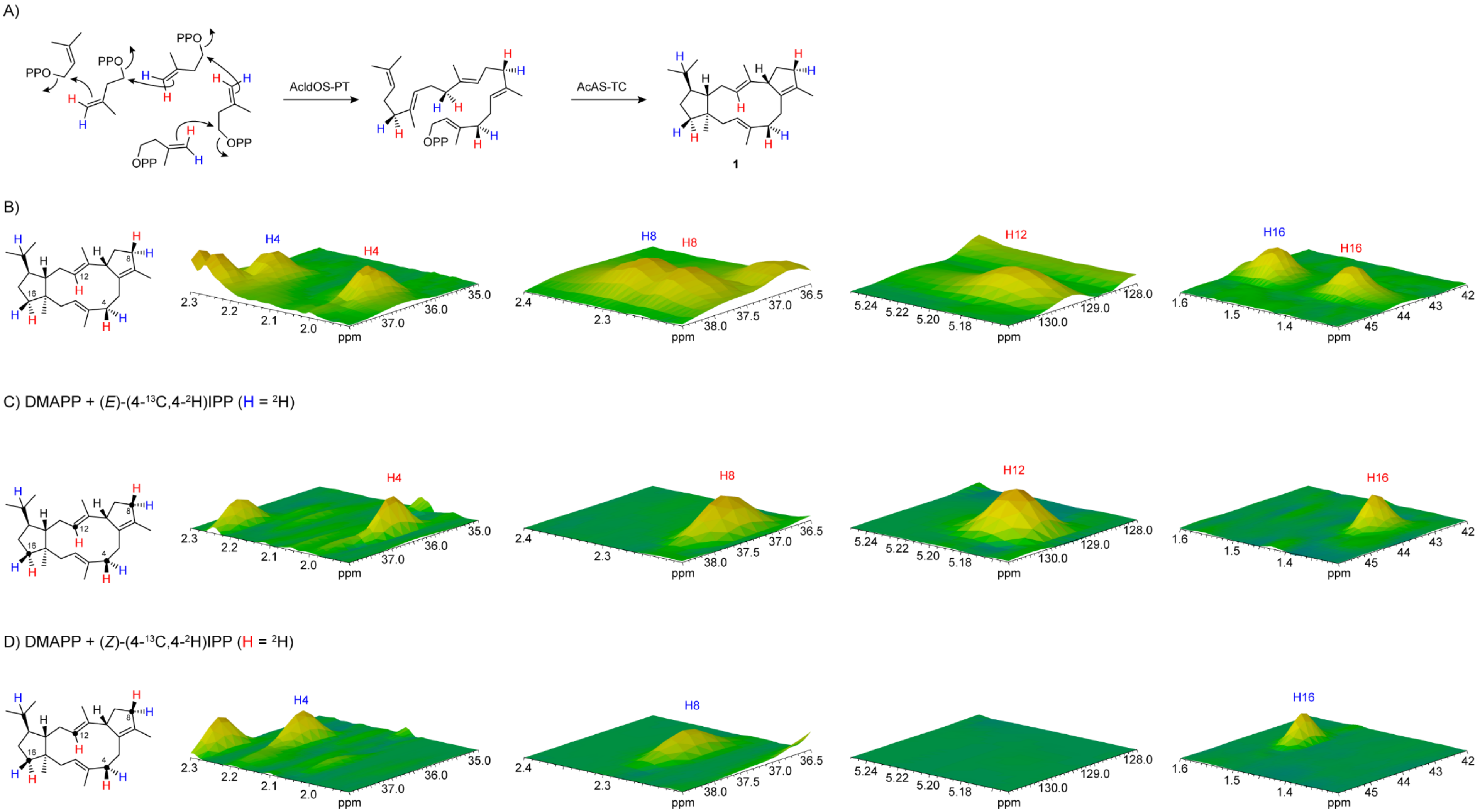

Figure S59. The absolute configuration of **1**. A) Enzymatic reaction of DMAPP and (*E*)- or (*Z*)-(4-^{13}C,4-^{2}H)IPP with AcldOS-PT and AcAS-TC. Partial HSQC spectra showing the regions for C4, C8, C12 and C16 of B) unlabelled **1**, C) labelled **1** obtained from DMAPP and (*E*)-(4-^{13}C,4-^{2}H)IPP (blue H = ^{2}H), and D) labelled **1** obtained from DMAPP and (*Z*)-(4-^{13}C,4-^{2}H)IPP (red H = ^{2}H). Colour code of hydrogens corresponds to the same colour code used in Table S2.

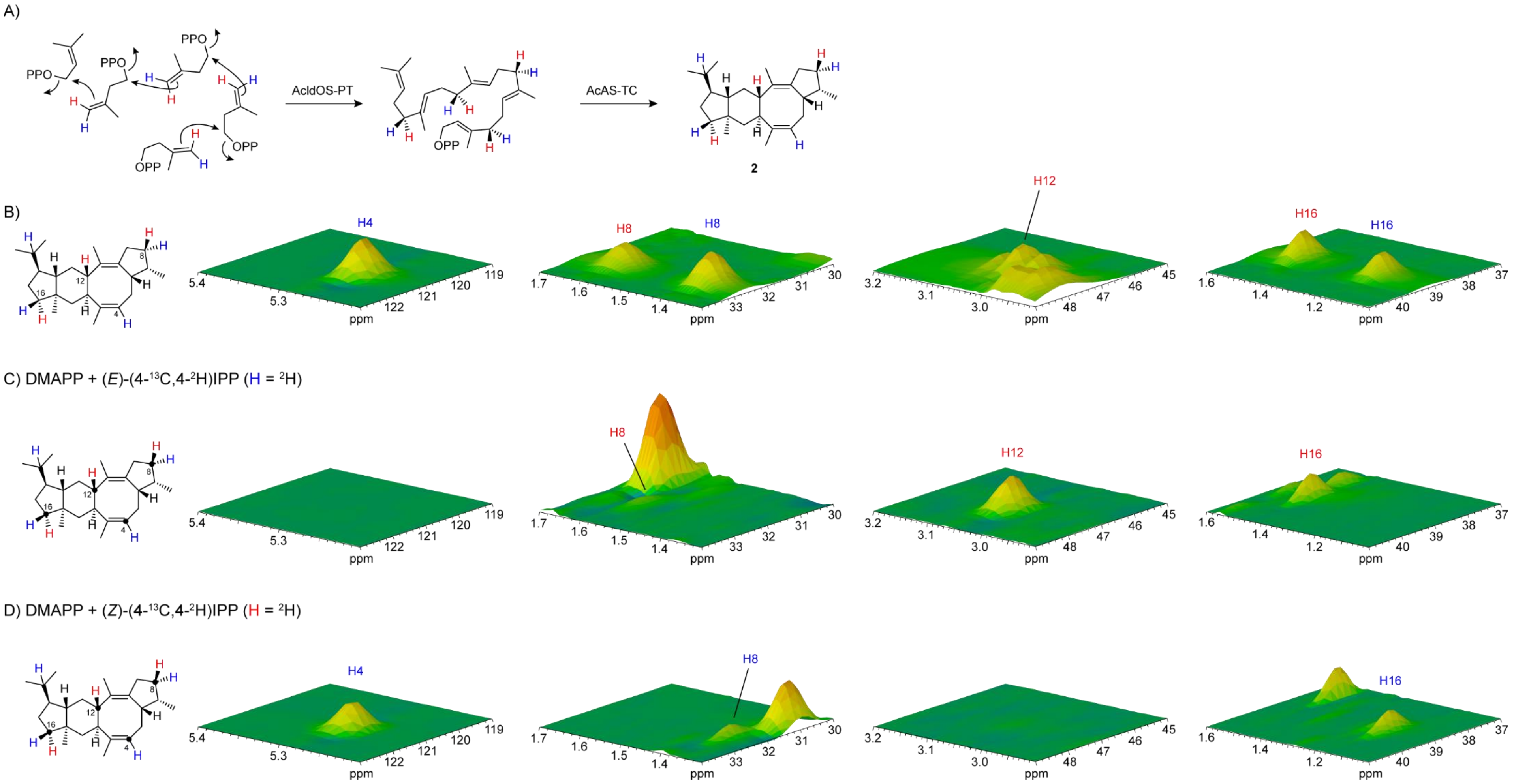

Figure S60. The absolute configuration of **2**. A) Enzymatic reaction of DMAPP and (*E*)- or (*Z*)-(4-^{13}C,4-^{2}H)IPP with AcldOS-PT and AcAS-TC. Partial HSQC spectra showing the regions for C4, C8, C12 and C16 of B) unlabelled **2**, C) labelled **2** obtained from DMAPP and (*E*)-(4-^{13}C,4-^{2}H)IPP (blue H = ^{2}H), and D) labelled **2** obtained from DMAPP and (*Z*)-(4-^{13}C,4-^{2}H)IPP (red H = ^{2}H). Colour code of hydrogens corresponds to the same colour code used in Table S3.

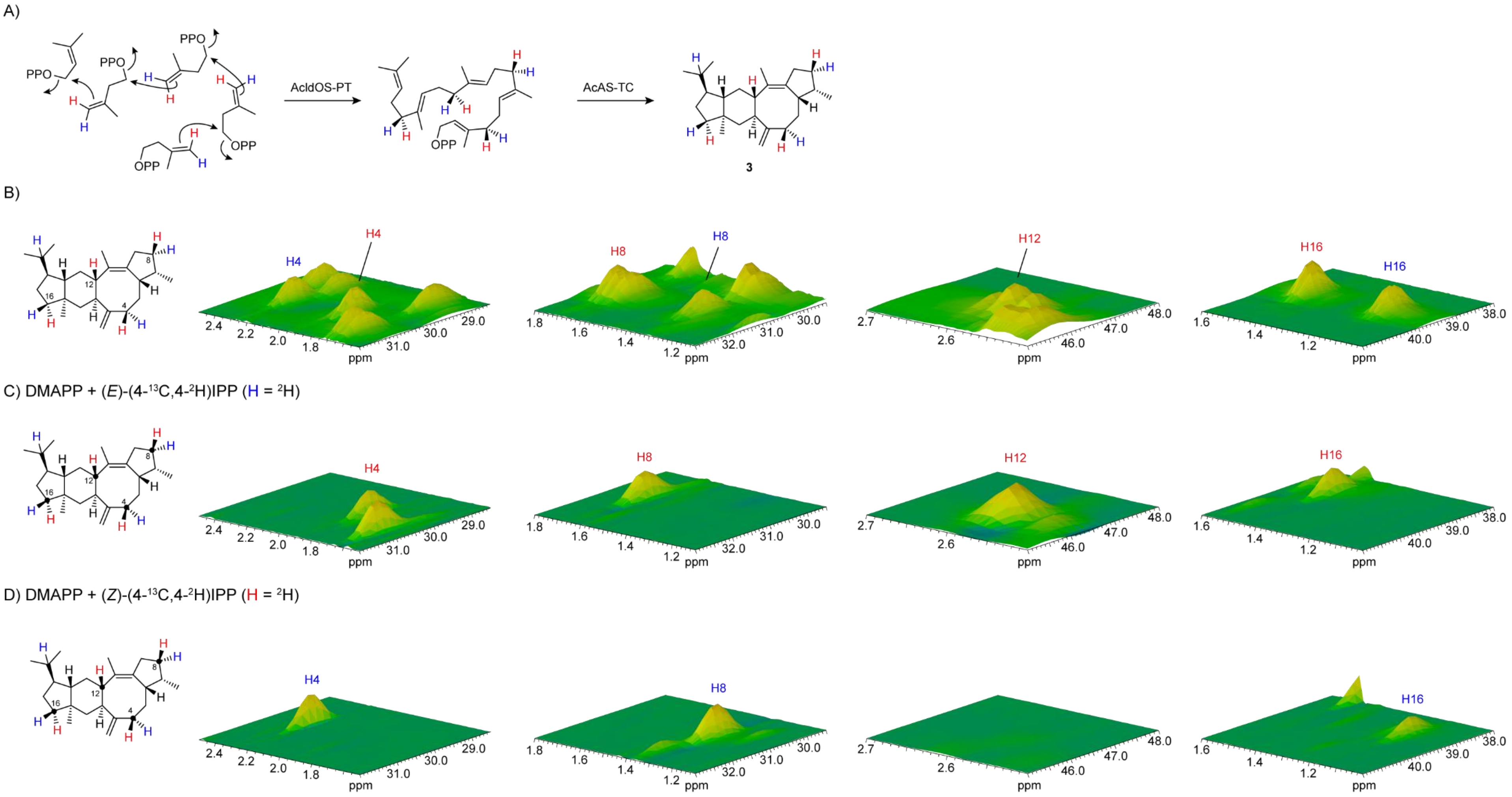

Figure S61. The absolute configuration of **3**. A) Enzymatic reaction of DMAPP and (*E*)- or (*Z*)-(4-^{13}C,4-^{2}H)IPP with AcldOS-PT and AcAS-TC. Partial HSQC spectra showing the regions for C4, C8, C12 and C16 of B) unlabelled **3**, C) labelled **3** obtained from DMAPP and (*E*)-(4-^{13}C,4-^{2}H)IPP (blue H = ^{2}H), and D) labelled **3** obtained from DMAPP and (*Z*)-(4-^{13}C,4-^{2}H)IPP (red H = ^{2}H). Colour code of hydrogens corresponds to the same colour code used in Table S4.

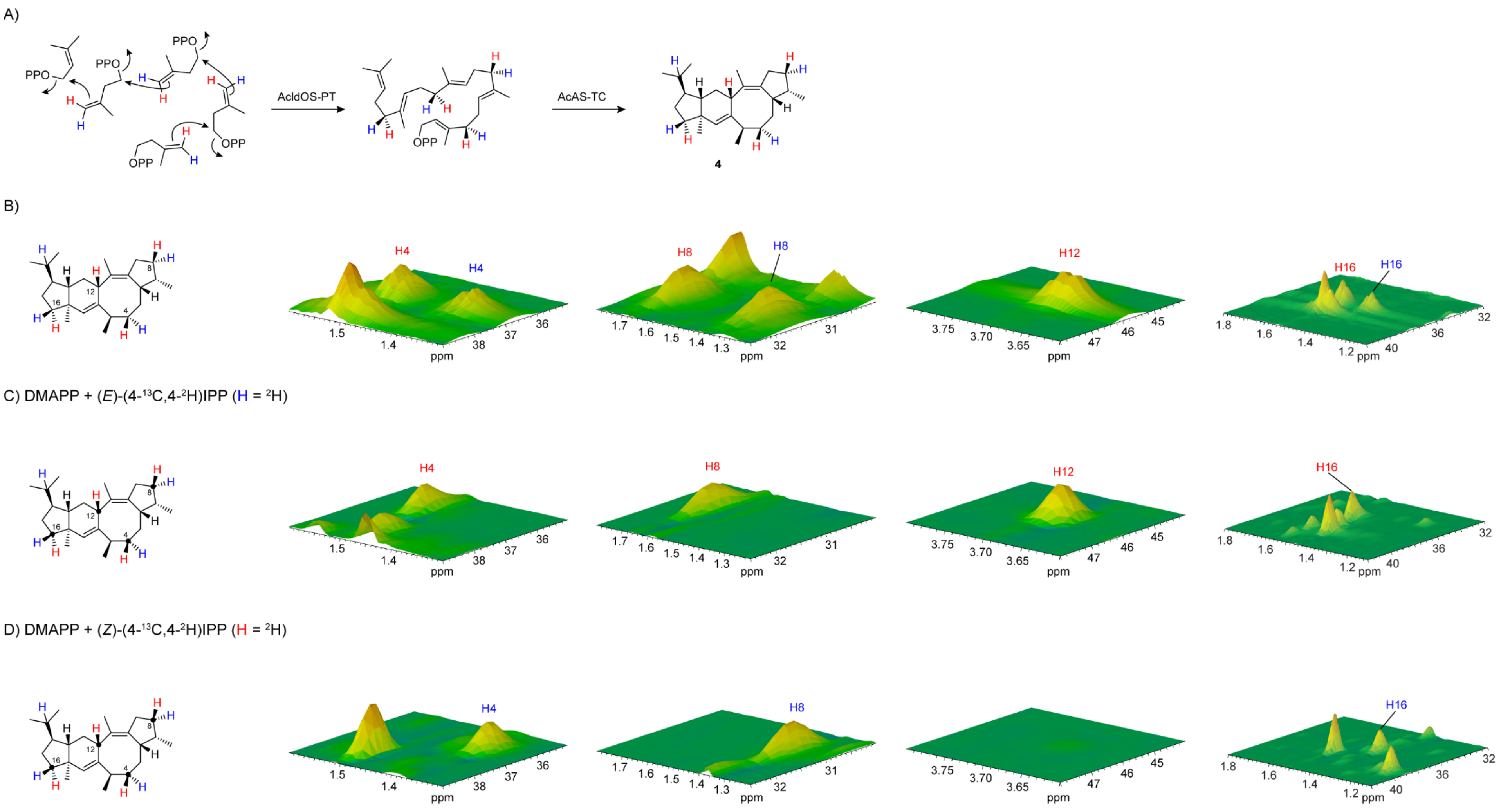

Figure S62. The absolute configuration of **4**. A) Enzymatic reaction of DMAPP and (*E*)- or (*Z*)-(4-^{13}C,4-^{2}H)IPP with AcldOS-PT and AcAS-TC. Partial HSQC spectra showing the regions for C4, C8, C12 and C16 of B) unlabelled **4**, C) labelled **4** obtained from DMAPP and (*E*)-(4-^{13}C,4-^{2}H)IPP (blue H = ^{2}H), and D) labelled **4** obtained from DMAPP and (*Z*)-(4-^{13}C,4-^{2}H)IPP (red H = ^{2}H). Colour code of hydrogens corresponds to the same colour code used in Table S5.

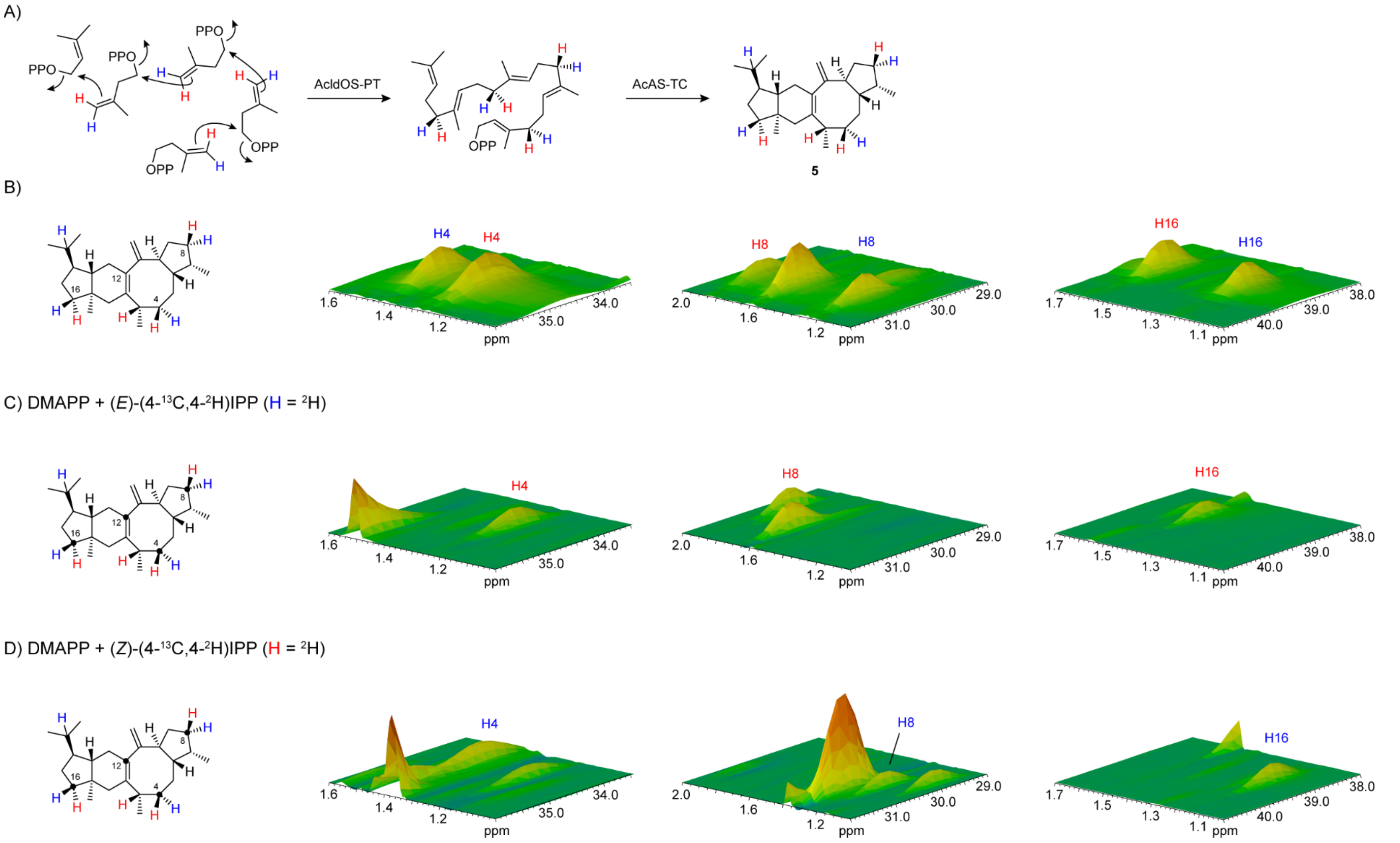

Figure S63. The absolute configuration of **5**. A) Enzymatic reaction of DMAPP and (*E*)- or (*Z*)-(4-^{13}C,4-^{2}H)IPP with AcldOS-PT and AcAS-TC. Partial HSQC spectra showing the regions for C4, C8, C12 and C16 of B) unlabelled **5**, C) labelled **5** obtained from DMAPP and (*E*)-(4-^{13}C,4-^{2}H)IPP (blue H = ^{2}H), and D) labelled **5** obtained from DMAPP and (*Z*)-(4-^{13}C,4-^{2}H)IPP (red H = ^{2}H). Colour code of hydrogens corresponds to the same colour code used in Table S6.

A)

B)

C) DMAPP + (*E*)-(4-^{13}C,4-^{2}H)IPP (H = ^{2}H)

D) DMAPP + (*Z*)-(4-^{13}C,4-^{2}H)IPP (H = ^{2}H)

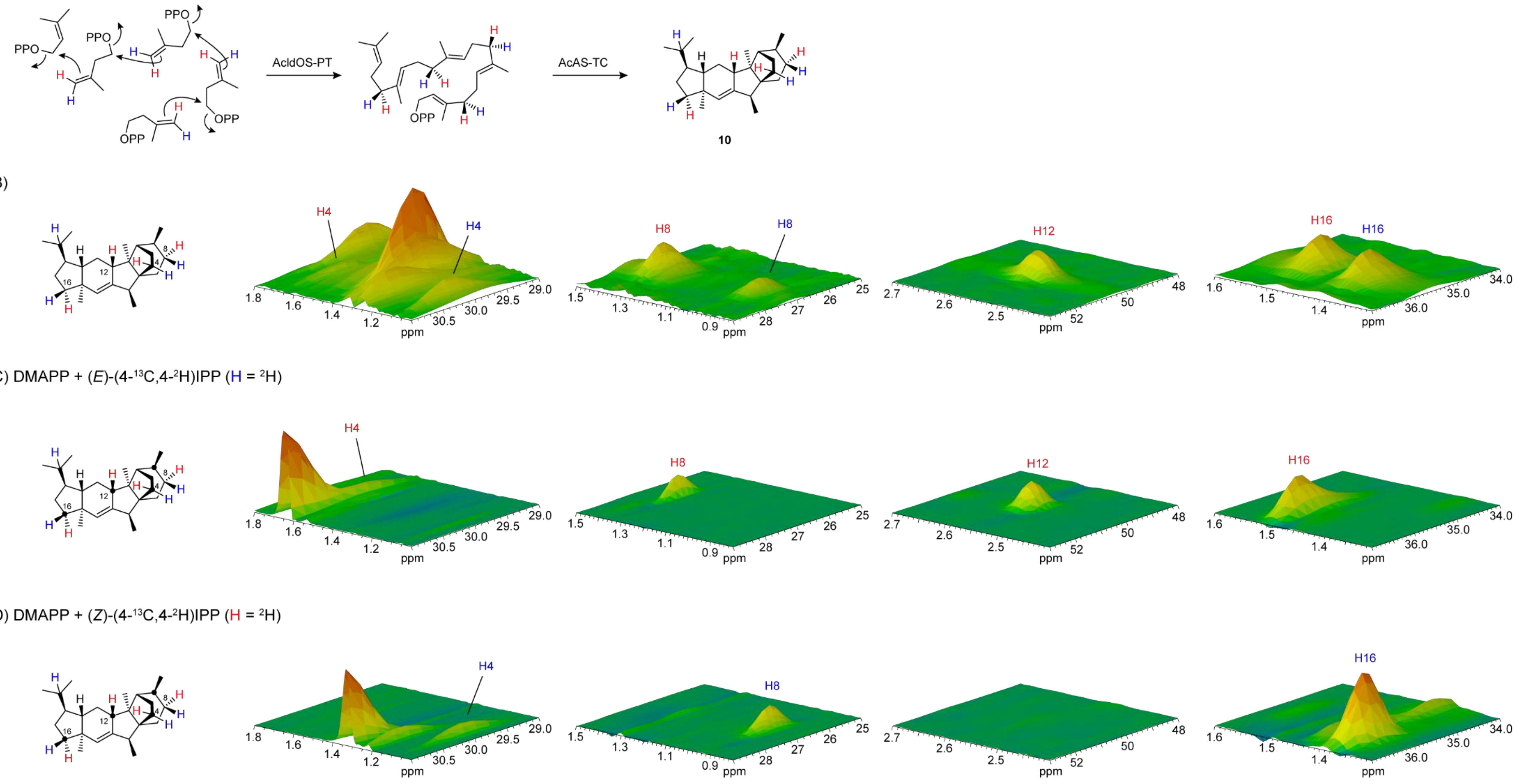

Figure S64. The absolute configuration of **10**. A) Enzymatic reaction of DMAPP and (*E*)- or (*Z*)-(4-^{13}C,4-^{2}H)IPP with AcldOS-PT and AcAS-TC. Partial HSQC spectra showing the regions for C4, C8, C12 and C16 of B) unlabelled **10**, C) labelled **10** obtained from DMAPP and (*E*)-(4-^{13}C,4-^{2}H)IPP (blue H = ^{2}H), and D) labelled **10** obtained from DMAPP and (*Z*)-(4-^{13}C,4-^{2}H)IPP (red H = ^{2}H). Colour code of hydrogens corresponds to the same colour code used in Table S7.

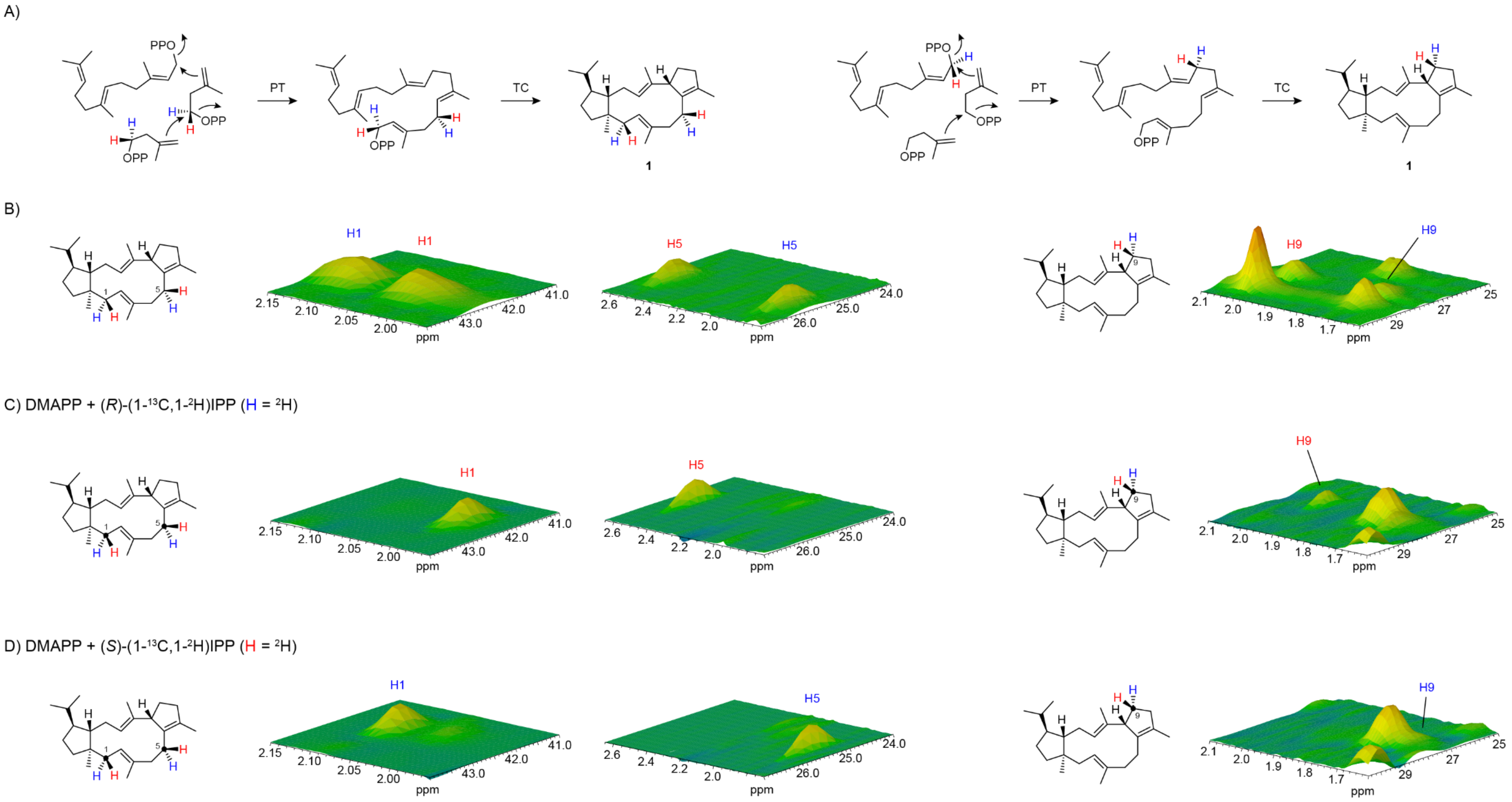

Figure S65. The absolute configuration of **1**. A) Enzymatic reactions of FPP and (*R*)- or (*S*)-(1-^{13}C,1-^{2}H)IPP and of (*R*)- or (*S*)-(1-^{13}C,1-^{2}H)FPP and IPP with AcAS. Partial HSQC spectra showing the regions for C1, C5, and C9 of B) unlabelled **1**, C) labelled **1** obtained from FPP and (*R*)-(1-^{13}C,1-^{2}H)IPP or (*R*)-(1-^{13}C,1-^{2}H)FPP and IPP (blue H = ^{2}H), and D) labelled **1** obtained from FPP and (*S*)-(1-^{13}C,1-^{2}H)IPP or (*S*)-(1-^{13}C,1-^{2}H)FPP and IPP (red H = ^{2}H). Colour code of hydrogens corresponds to the same colour code used in Table S2.

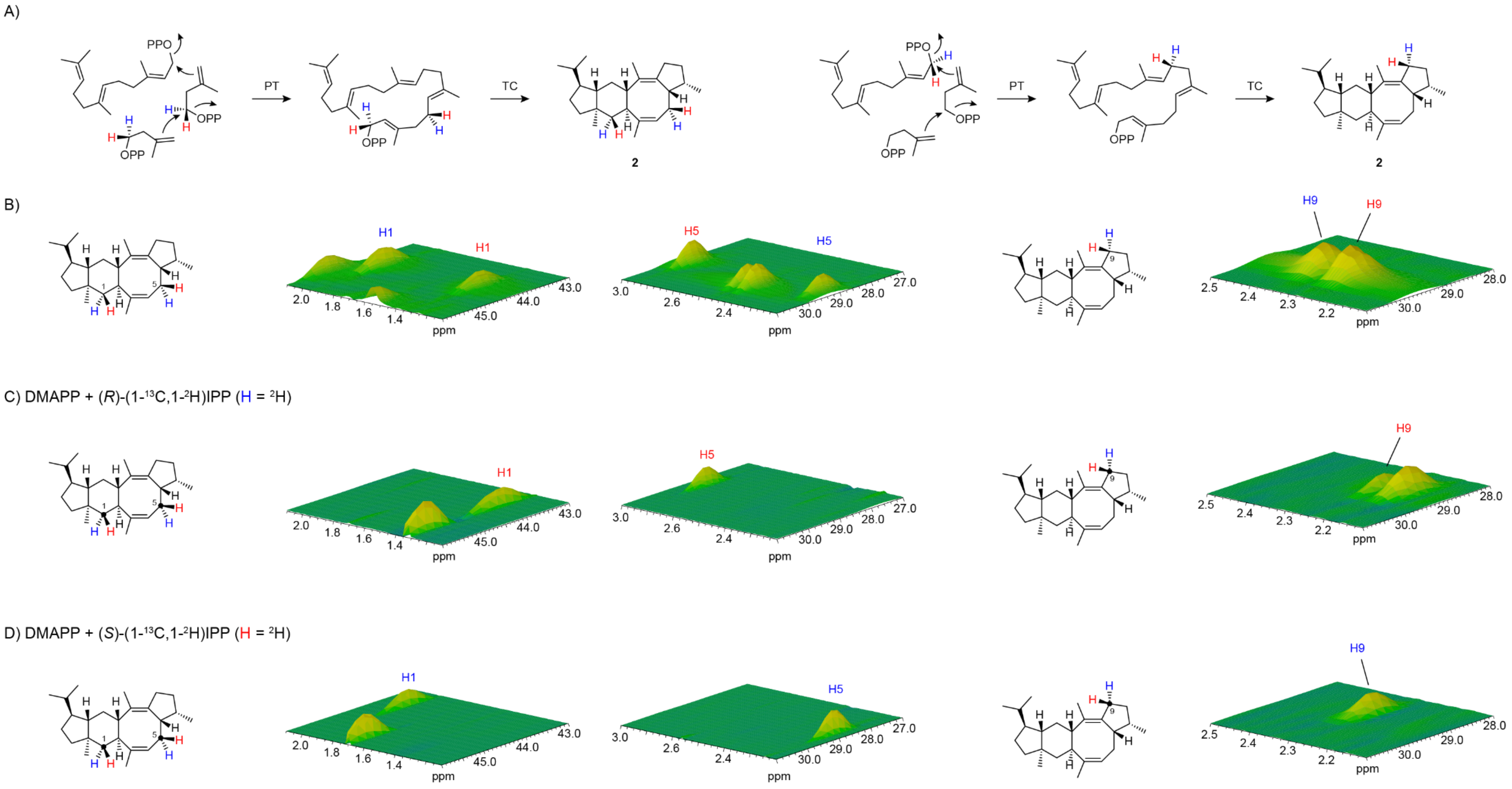

Figure S66. The absolute configuration of **2**. A) Enzymatic reactions of FPP and (*R*)- or (*S*)-(1-^{13}C,1-^{2}H)IPP and of (*R*)- or (*S*)-(1-^{13}C,1-^{2}H)FPP and IPP with AcAS. Partial HSQC spectra showing the regions for C1, C5, and C9 of B) unlabelled **2**, C) labelled **2** obtained from FPP and (*R*)-(1-^{13}C,1-^{2}H)IPP or (*R*)-(1-^{13}C,1-^{2}H)FPP and IPP (blue H = ^{2}H), and D) labelled **2** obtained from FPP and (*S*)-(1-^{13}C,1-^{2}H)IPP or (*S*)-(1-^{13}C,1-^{2}H)FPP and IPP (red H = ^{2}H). Colour code of hydrogens corresponds to the same colour code used in Table S3.

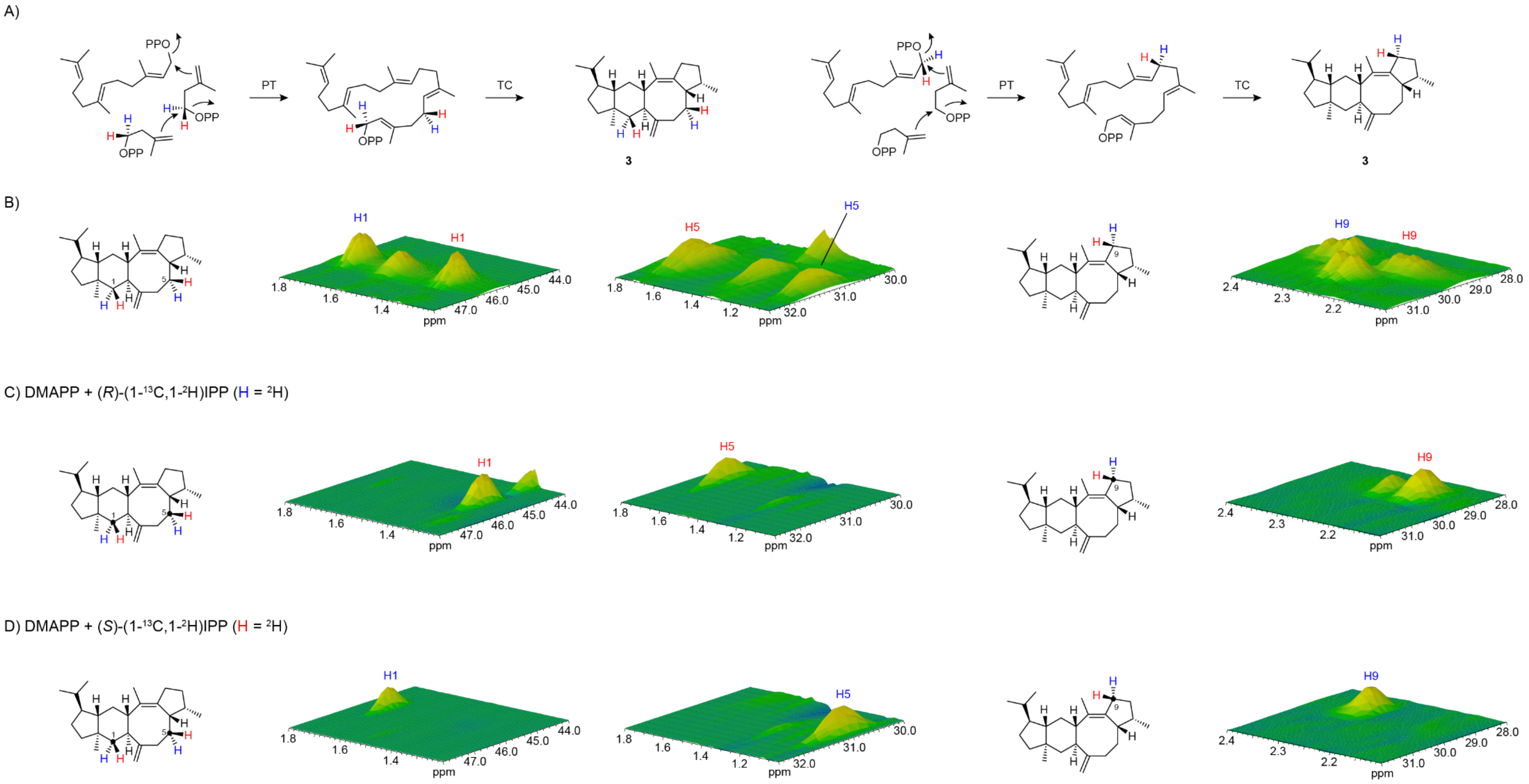

Figure S67. The absolute configuration of **3**. A) Enzymatic reactions of FPP and (*R*)- or (*S*)-(1-^{13}C,1-^{2}H)IPP and of (*R*)- or (*S*)-(1-^{13}C,1-^{2}H)FPP and IPP with AcAS. Partial HSQC spectra showing the regions for C1, C5, and C9 of B) unlabelled **3**, C) labelled **3** obtained from FPP and (*R*)-(1-^{13}C,1-^{2}H)IPP or (*R*)-(1-^{13}C,1-^{2}H)FPP and IPP (blue H = ^{2}H), and D) labelled **3** obtained from FPP and (*S*)-(1-^{13}C,1-^{2}H)IPP or (*S*)-(1-^{13}C,1-^{2}H)FPP and IPP (red H = ^{2}H). Colour code of hydrogens corresponds to the same colour code used in Table S4.

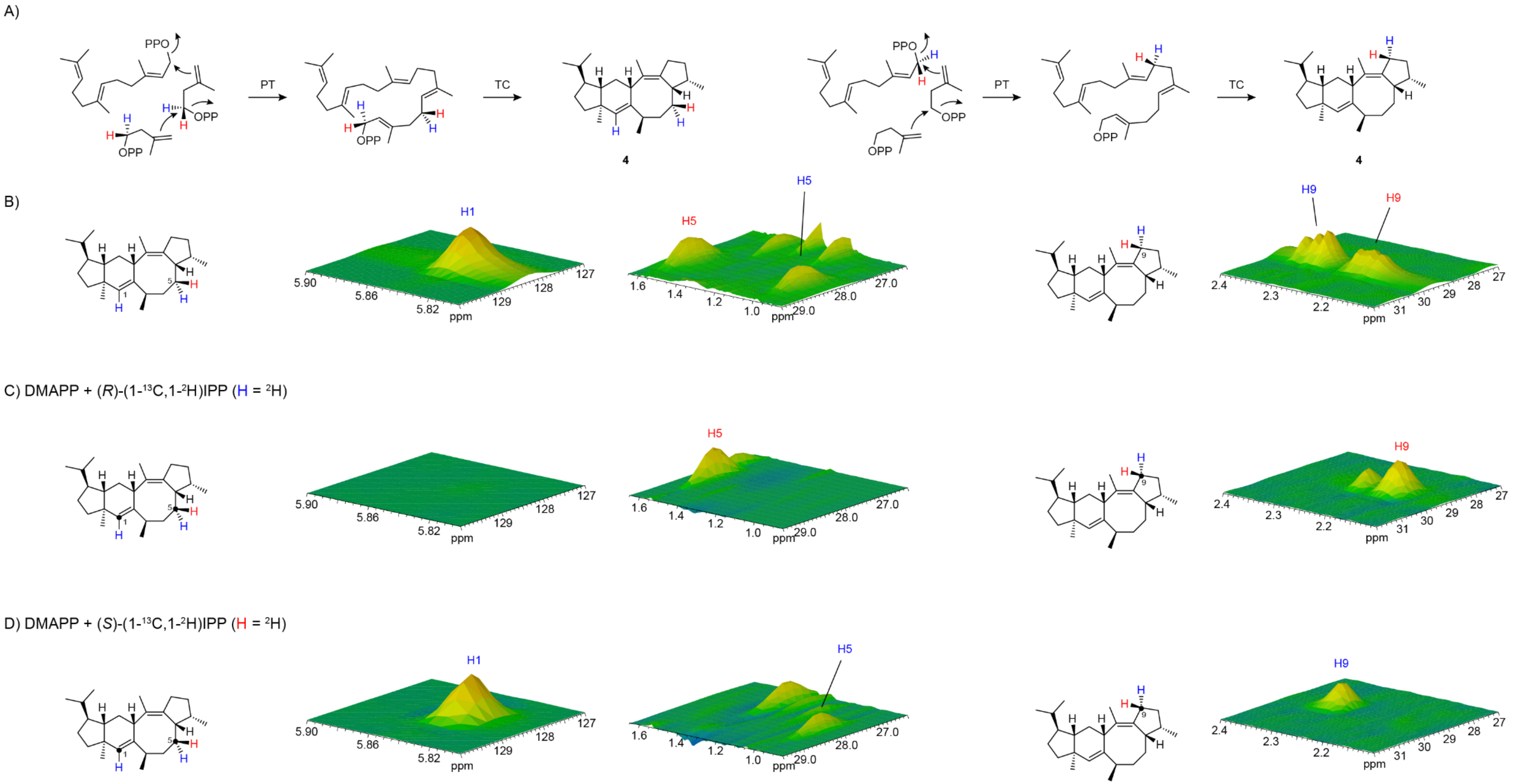

Figure S68. The absolute configuration of **4**. A) Enzymatic reactions of FPP and (*R*)- or (*S*)-(1-^{13}C,1-^{2}H)IPP and of (*R*)- or (*S*)-(1-^{13}C,1-^{2}H)FPP and IPP with AcAS. Partial HSQC spectra showing the regions for C1, C5, and C9 of B) unlabelled **4**, C) labelled **4** obtained from FPP and (*R*)-(1-^{13}C,1-^{2}H)IPP or (*R*)-(1-^{13}C,1-^{2}H)FPP and IPP (blue H = ^{2}H), and D) labelled **4** obtained from FPP and (*S*)-(1-^{13}C,1-^{2}H)IPP or (*S*)-(1-^{13}C,1-^{2}H)FPP and IPP (red H = ^{2}H). Colour code of hydrogens corresponds to the same colour code used in Table S5.

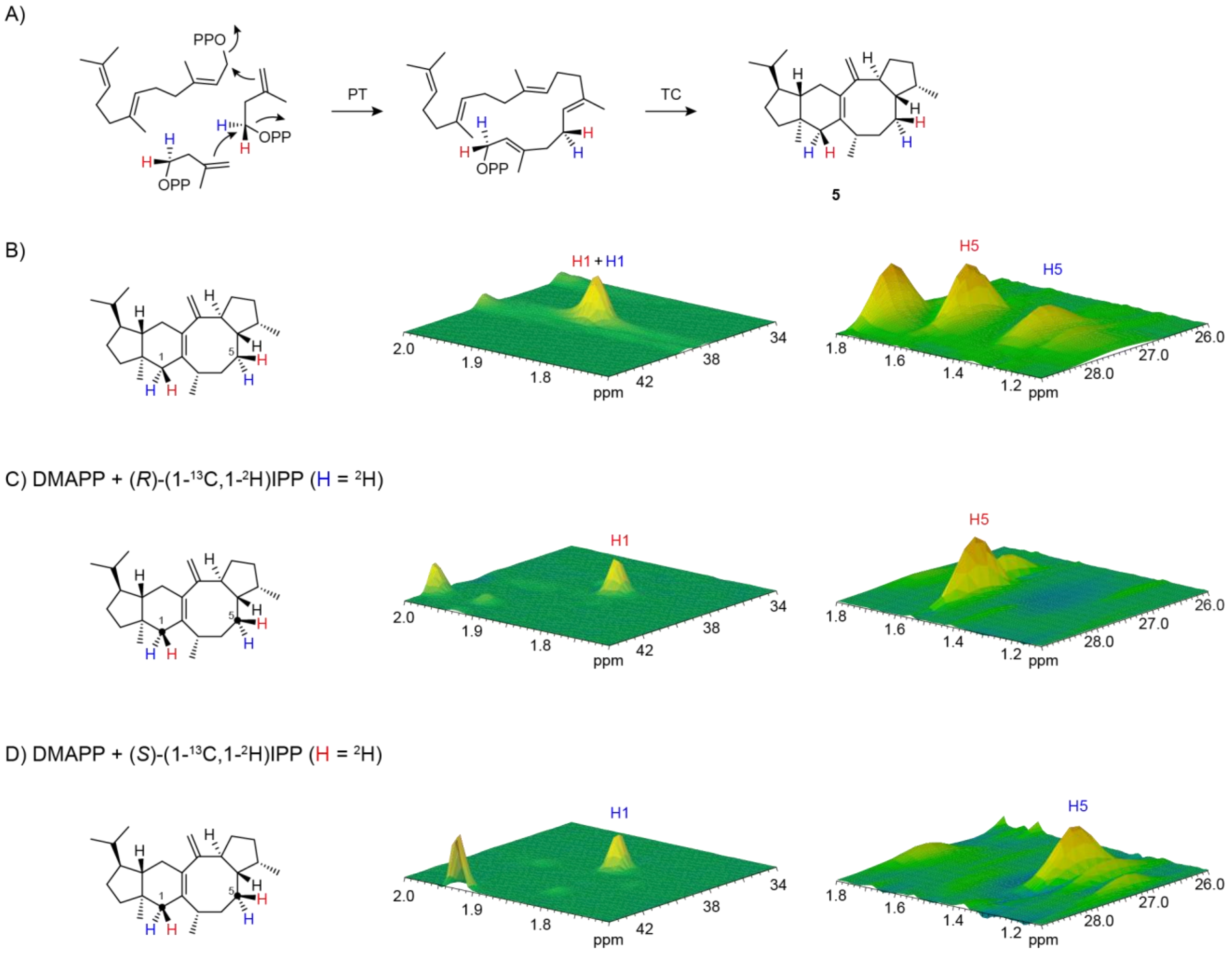

Figure S69. The absolute configuration of **5**. A) Enzymatic reaction of FPP and (*R*)- or (*S*)-(1-^{13}C,1-^{2}H)IPP with AcAS. Partial HSQC spectra showing the regions for C1 and C5 of B) unlabelled **5**, C) labelled **5** obtained from FPP and (*R*)-(1-^{13}C,1-^{2}H)IPP (blue H = ^{2}H), and D) labelled **5** obtained from FPP and (*S*)-(1-^{13}C,1-^{2}H)IPP (red H = ^{2}H). Colour code of hydrogens corresponds to the same colour code used in Table S6.

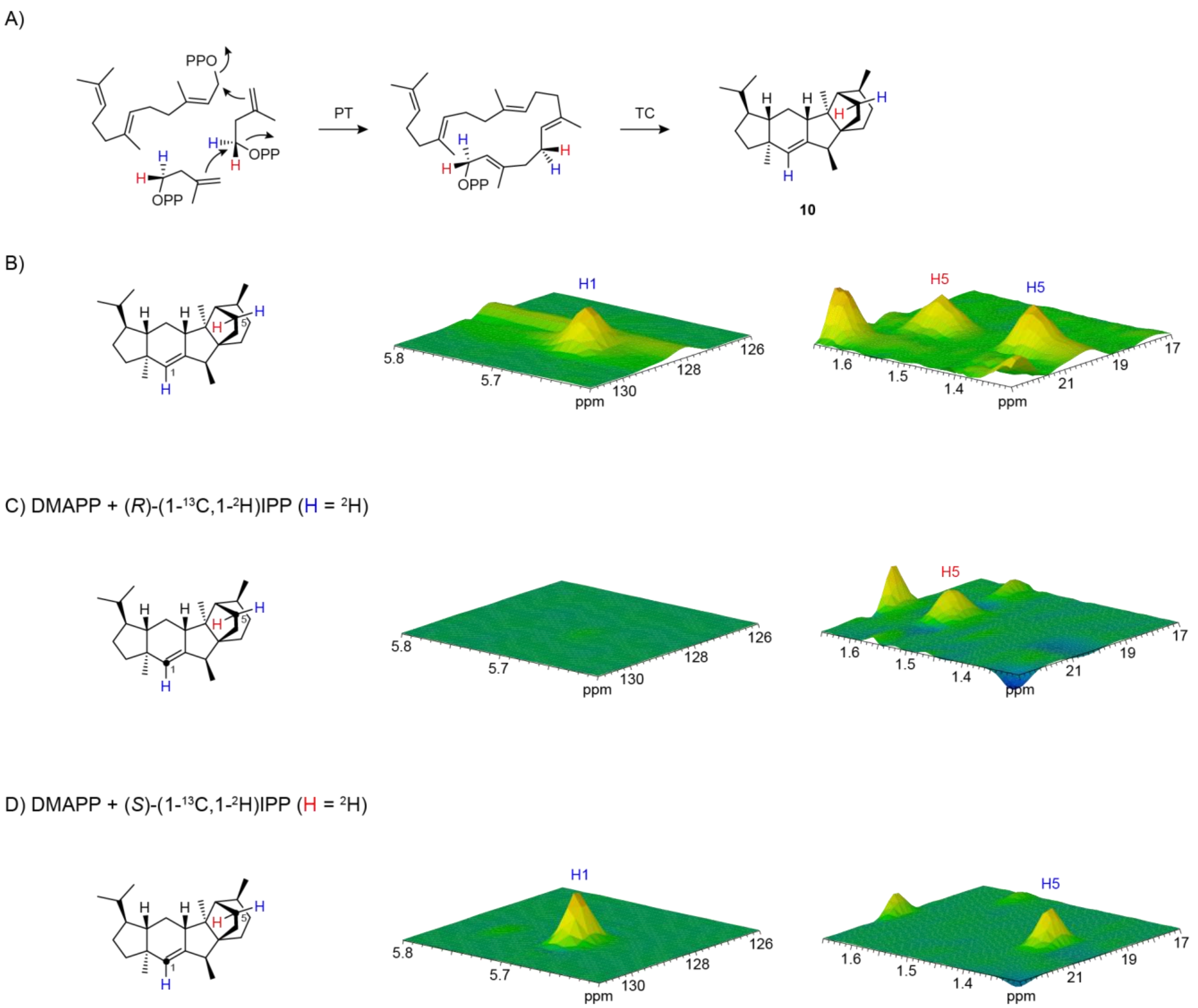

Figure S70. The absolute configuration of **10**. A) Enzymatic reaction of FPP and (*R*)- or (*S*)-(1-^{13}C,1-^{2}H)IPP with AcAS. Partial HSQC spectra showing the regions for C1 and C5 of B) unlabelled **10**, C) labelled **10** obtained from FPP and (*R*)-(1-^{13}C,1-^{2}H)IPP (blue H = ^{2}H), and D) labelled **10** obtained from FPP and (*S*)-(1-^{13}C,1-^{2}H)IPP (red H = ^{2}H). Colour code of hydrogens corresponds to the same colour code used in Table S7.

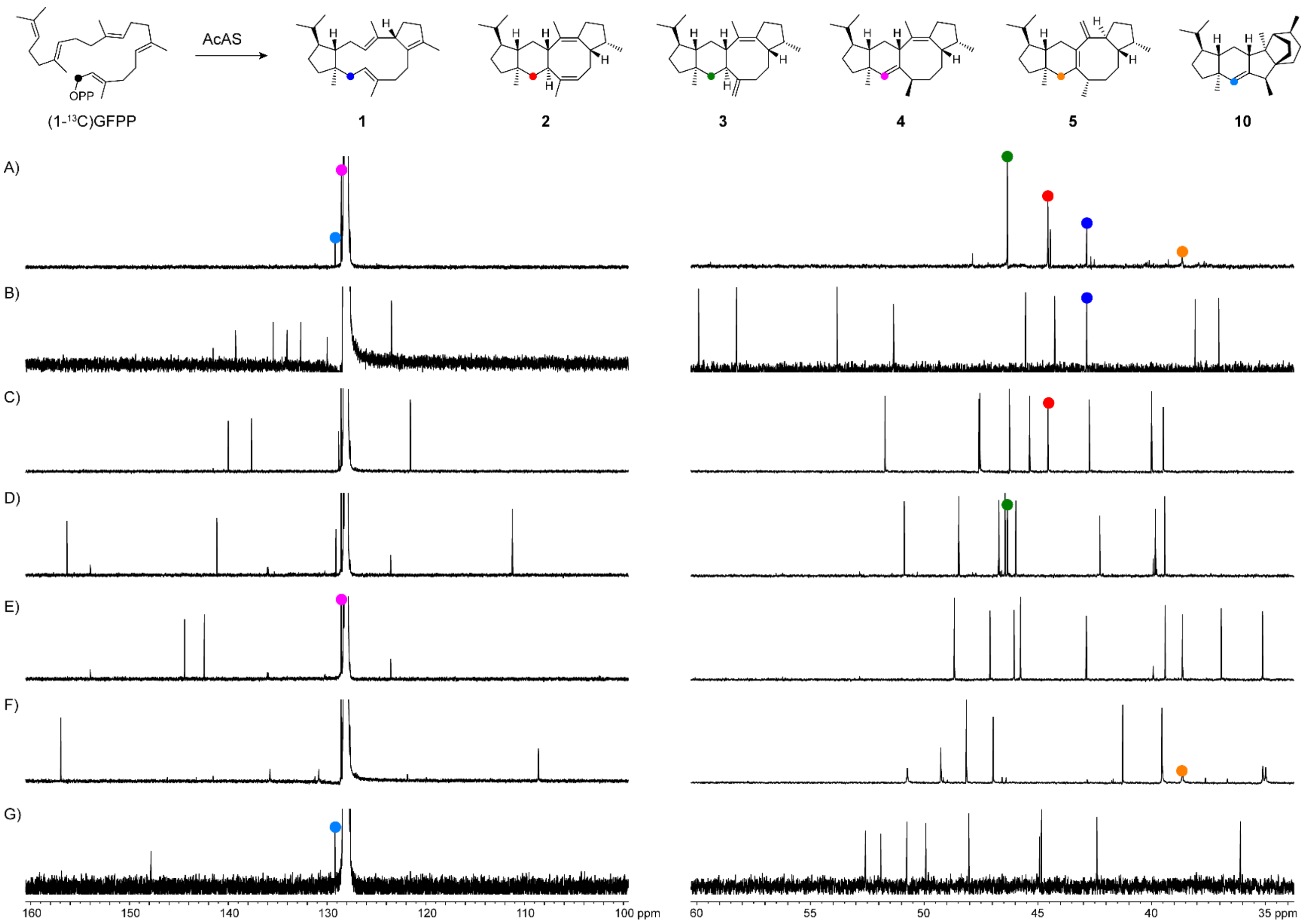

Figure S71. Partial ^{13}C-NMR spectra showing the regions for C1 of A) the mixture of labelled **1** – **5** and **10** obtained from (1-^{13}C)GFPP, and of the unlabelled compounds B) **1**, C) **2**, D) **3**, E) **4**, F) **5**, and G) **10**. Coloured dots at peaks correspond to the dots at the structures. Each of the parts A) – G) was produced from one ^{13}C-NMR spectrum.

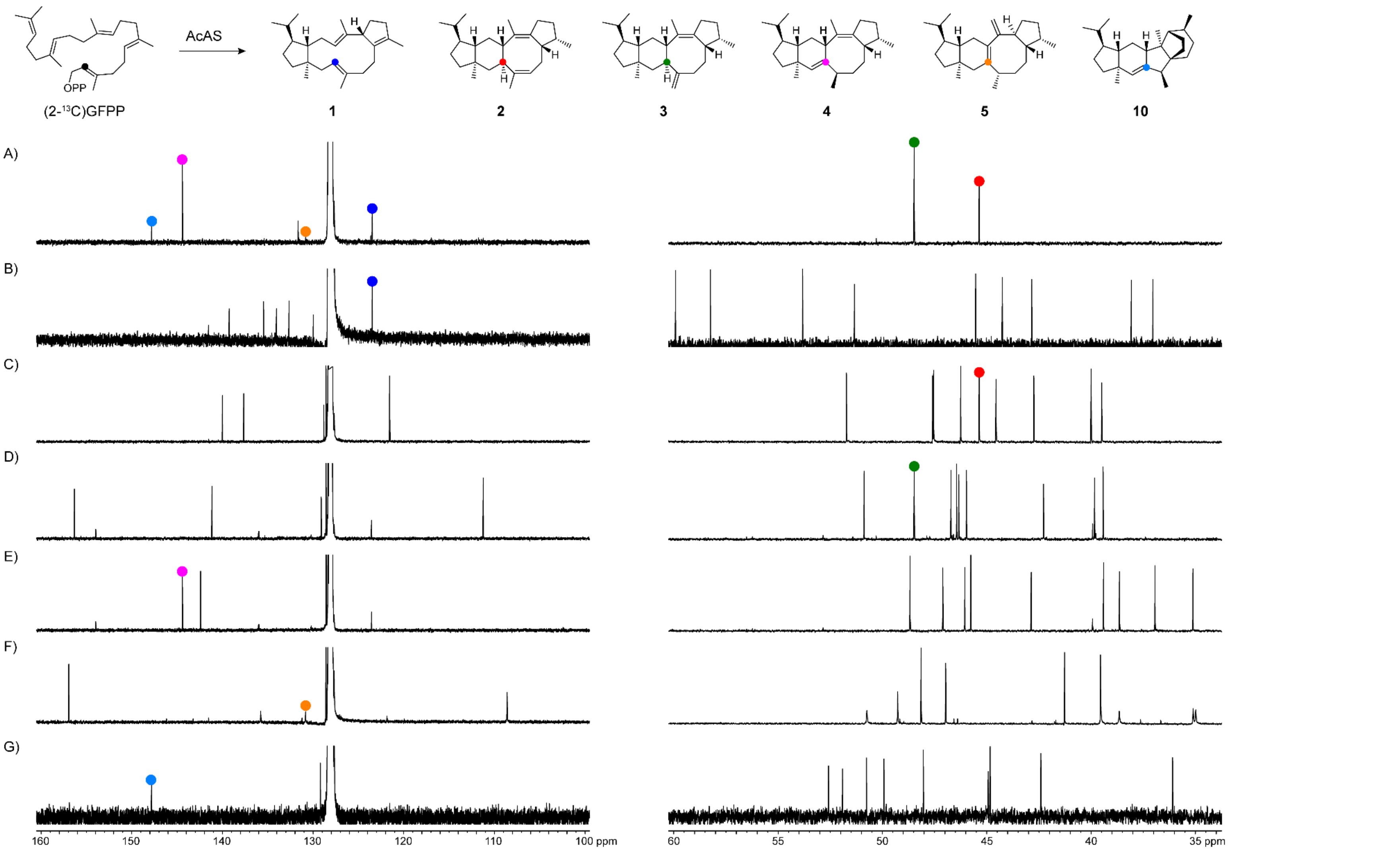

Figure S72. Partial ^{13}C-NMR spectra showing the regions for C2 of A) the mixture of labelled **1** – **5** and **10** obtained from (2-^{13}C)GFPP, and of the unlabelled compounds B) **1**, C) **2**, D) **3**, E) **4**, F) **5**, and G) **10**. Coloured dots at peaks correspond to the dots at the structures. Each of the parts A) – G) was produced from one ^{13}C-NMR spectrum.

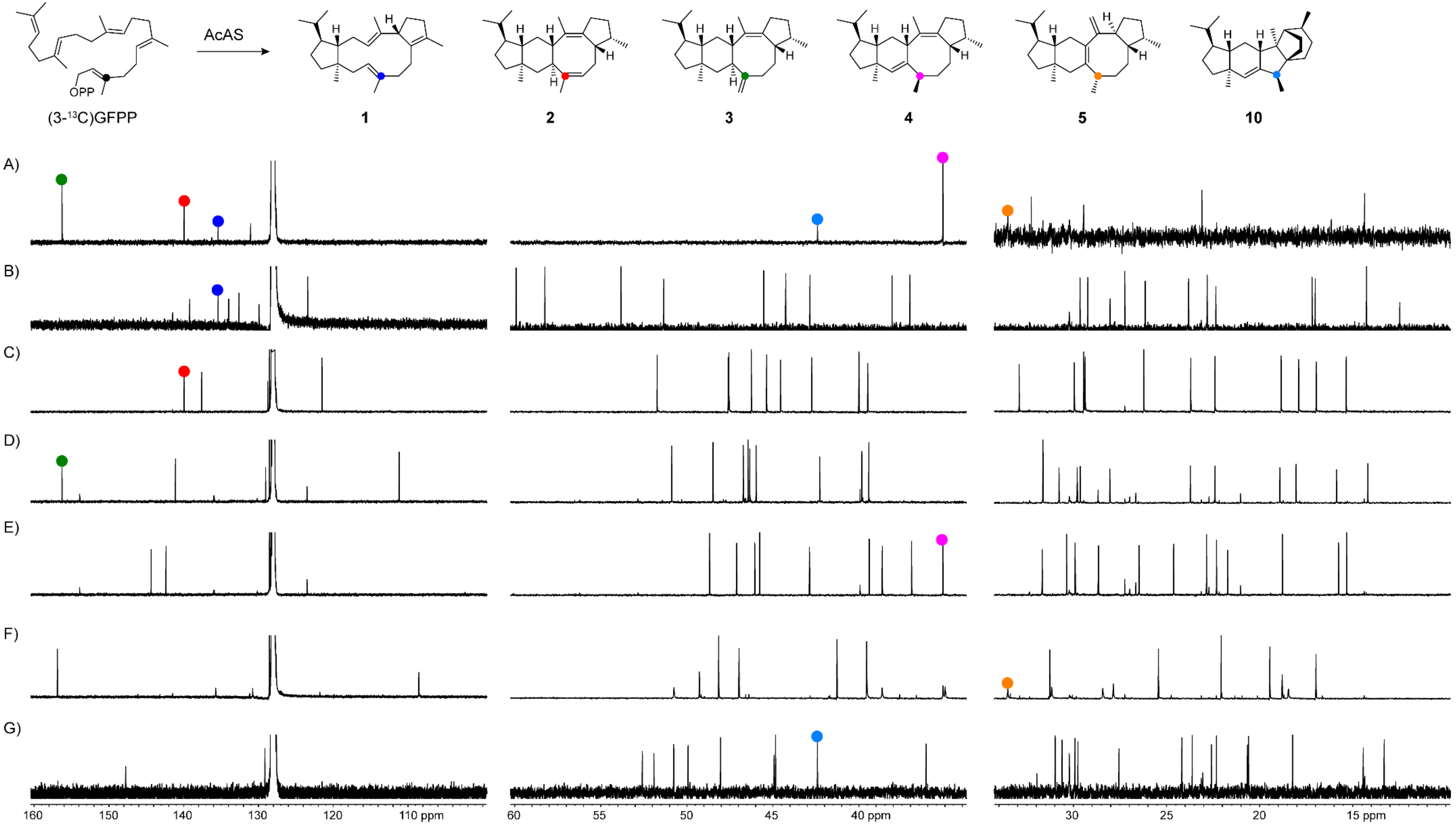

Figure S73. Partial ^{13}C-NMR spectra showing the regions for C3 of A) the mixture of labelled **1** – **5** and **10** obtained from (3-^{13}C)GFPP, and of the unlabelled compounds B) **1**, C) **2**, D) **3**, E) **4**, F) **5**, and G) **10**. Coloured dots at peaks correspond to the dots at the structures. Each of the parts A) – G) was produced from one ^{13}C-NMR spectrum.

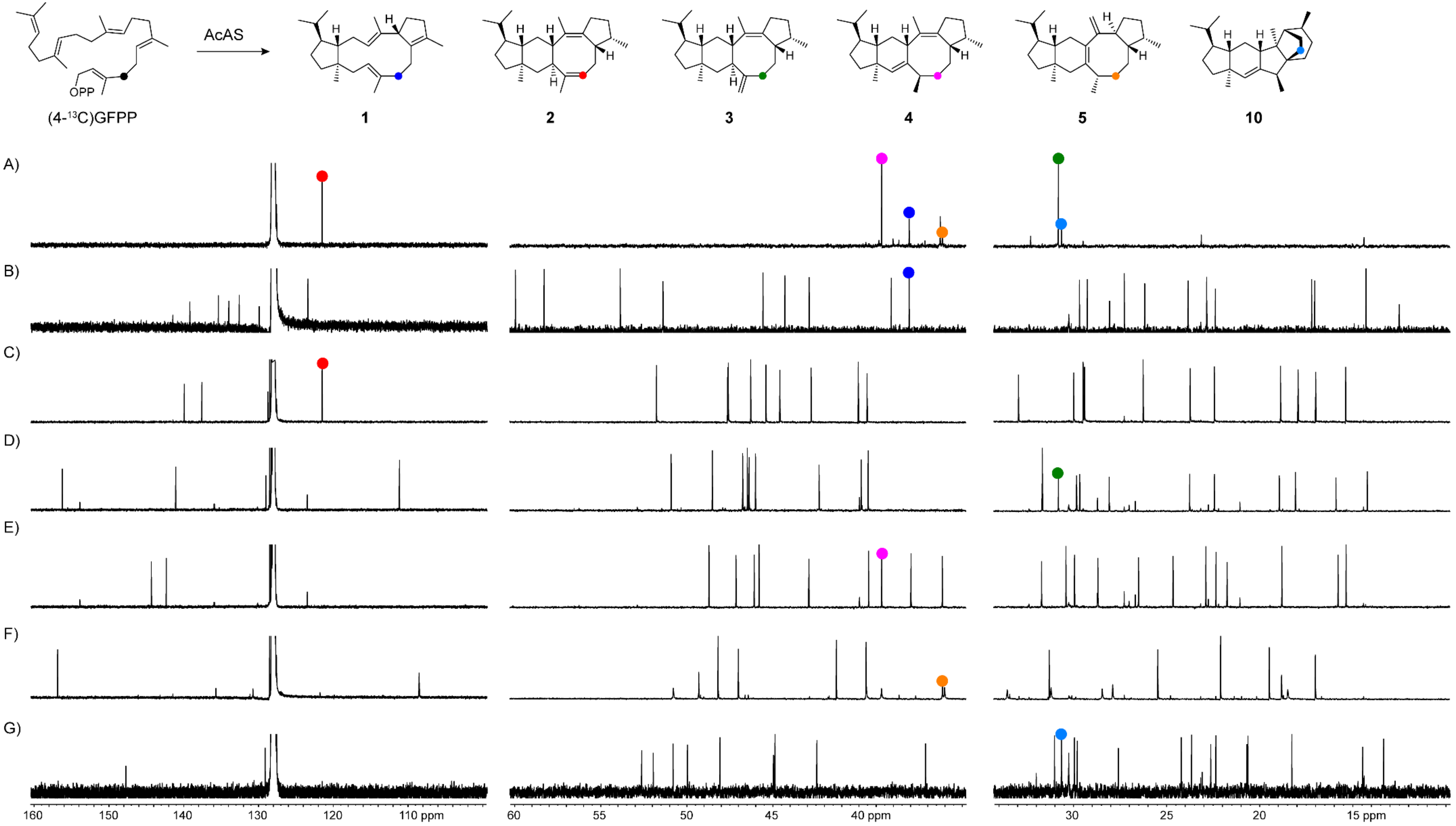

Figure S74. Partial ^{13}C-NMR spectra showing the regions for C4 of A) the mixture of labelled **1** – **5** and **10** obtained from (4-^{13}C)GFPP, and of the unlabelled compounds B) **1**, C) **2**, D) **3**, E) **4**, F) **5**, and G) **10**. Coloured dots at peaks correspond to the dots at the structures. Each of the parts A) – G) was produced from one ^{13}C-NMR spectrum.

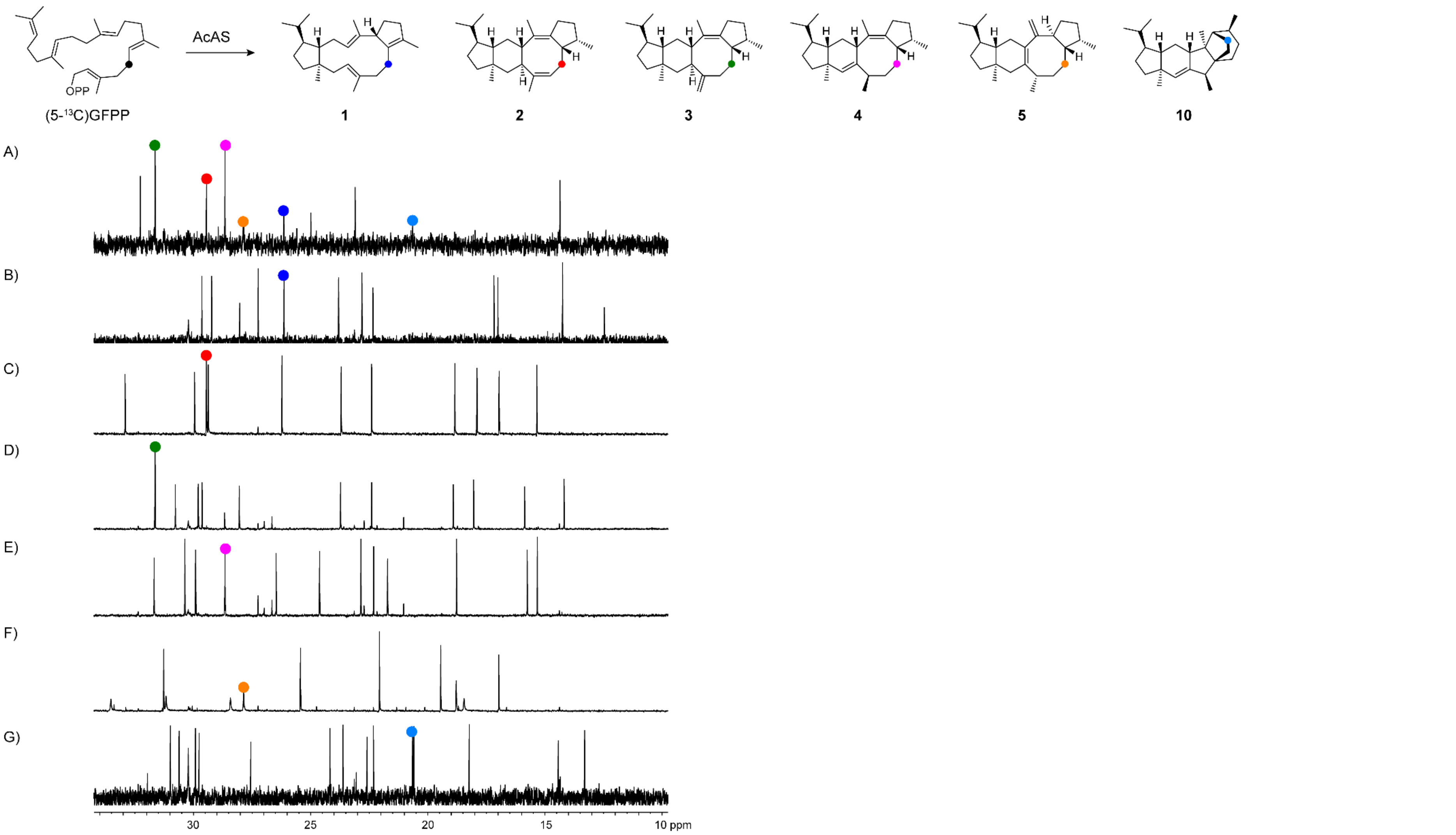

Figure S75. Partial ^{13}C-NMR spectra showing the regions for C5 of A) the mixture of labelled **1** – **5** and **10** obtained from (5-^{13}C)GFPP, and of the unlabelled compounds B) **1**, C) **2**, D) **3**, E) **4**, F) **5**, and G) **10**. Coloured dots at peaks correspond to the dots at the structures.

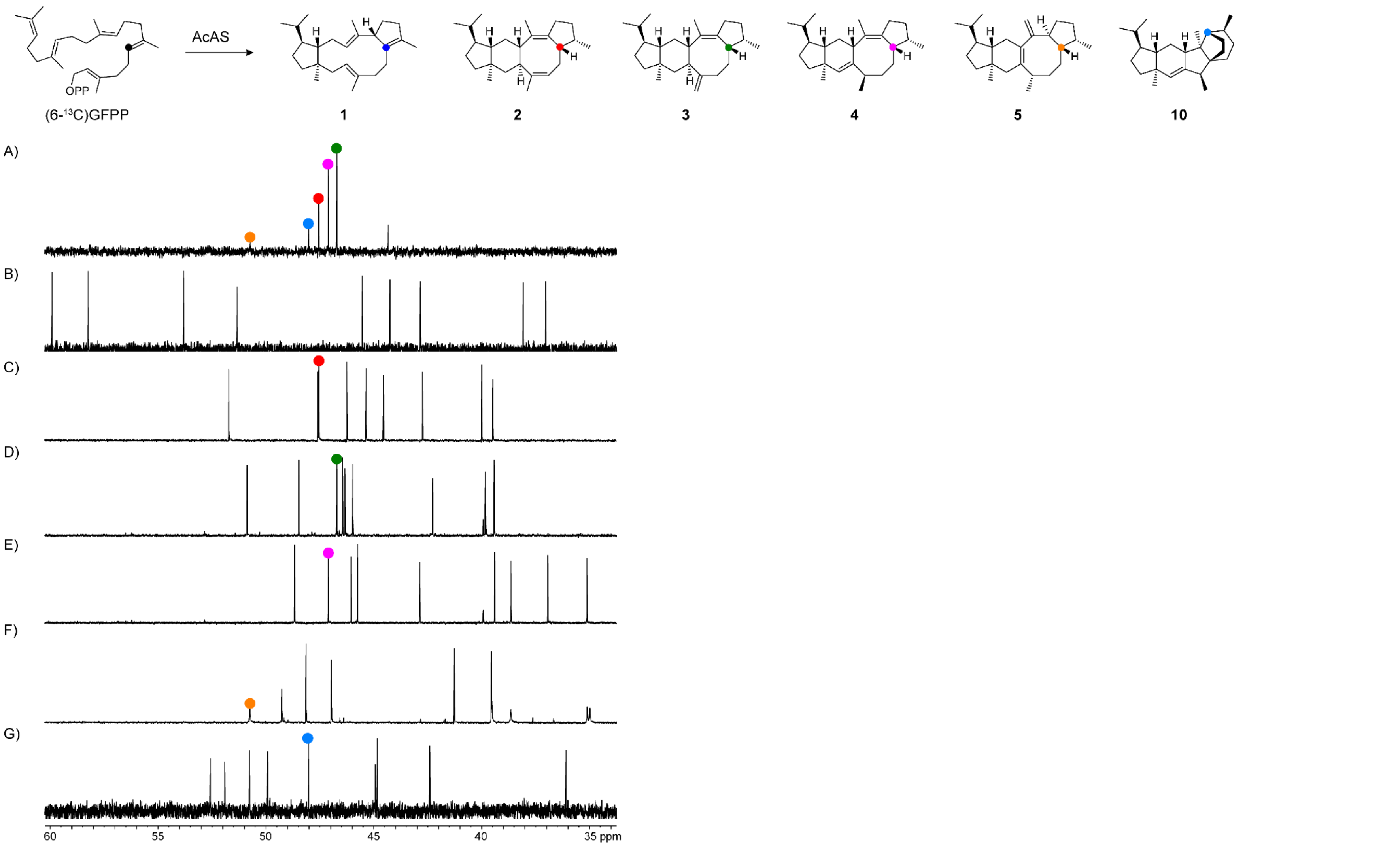

Figure S76. Partial ^{13}C-NMR spectra showing the regions for C6 of A) the mixture of labelled **1 – 5** and **10** obtained from (6-^{13}C)GFPP, and of the unlabelled compounds B) **1**, C) **2**, D) **3**, E) **4**, F) **5**, and G) **10**. Coloured dots at peaks correspond to the dots at the structures.

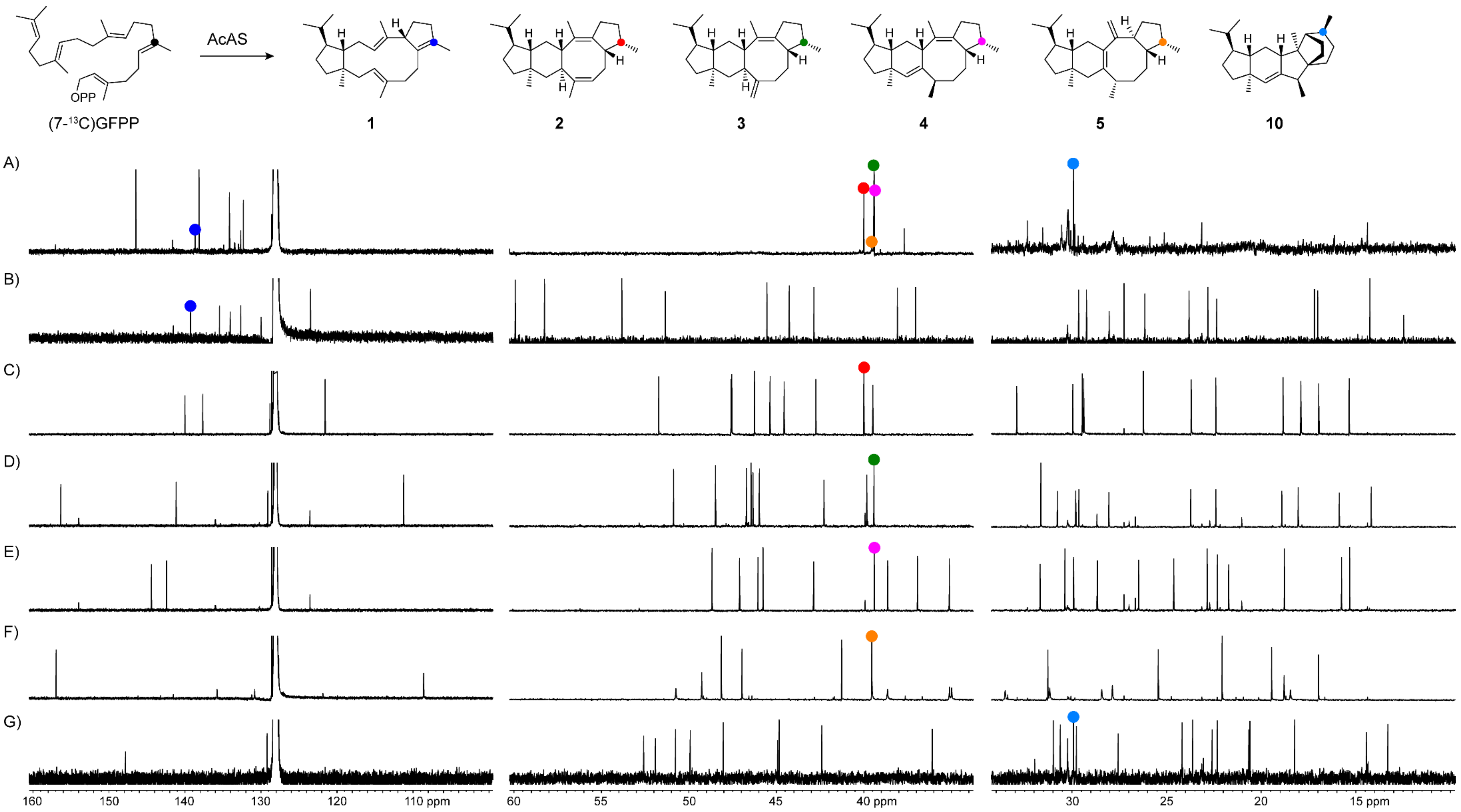

Figure S77. Partial ^{13}C-NMR spectra showing the regions for C7 of A) the mixture of labelled **1 – 5** and **10** obtained from (7-^{13}C)GFPP, and of the unlabelled compounds B) **1**, C) **2**, D) **3**, E) **4**, F) **5**, and G) **10**. Coloured dots at peaks correspond to the dots at the structures. Each of the parts A) – G) was produced from one ^{13}C-NMR spectrum.

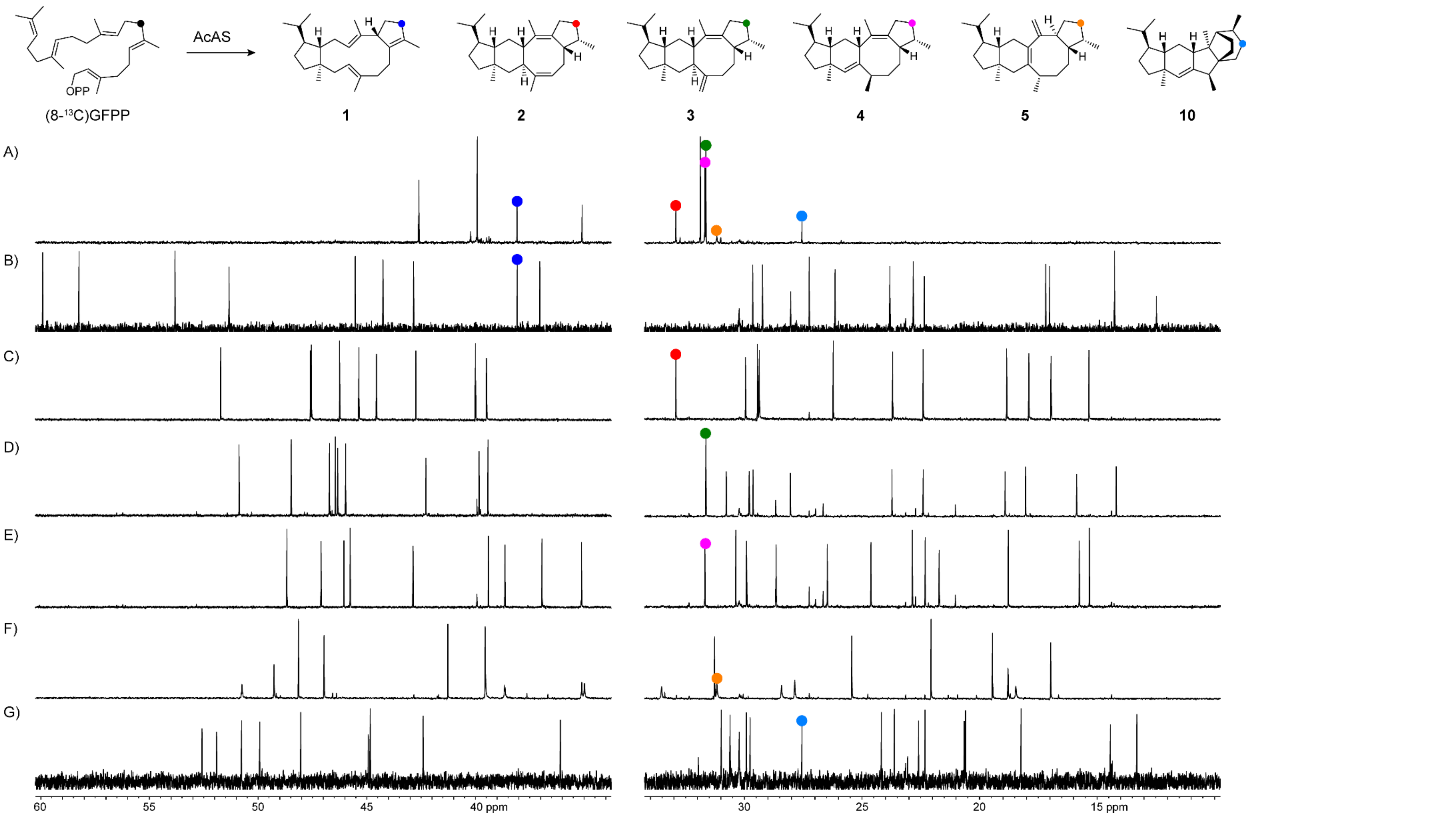

Figure S78. Partial ^{13}C-NMR spectra showing the regions for C8 of A) the mixture of labelled **1 – 5** and **10** obtained from (8-^{13}C)GFPP, and of the unlabelled compounds B) **1**, C) **2**, D) **3**, E) **4**, F) **5**, and G) **10**. Coloured dots at peaks correspond to the dots at the structures. Each of the parts A) – G) was produced from one ^{13}C-NMR spectrum.

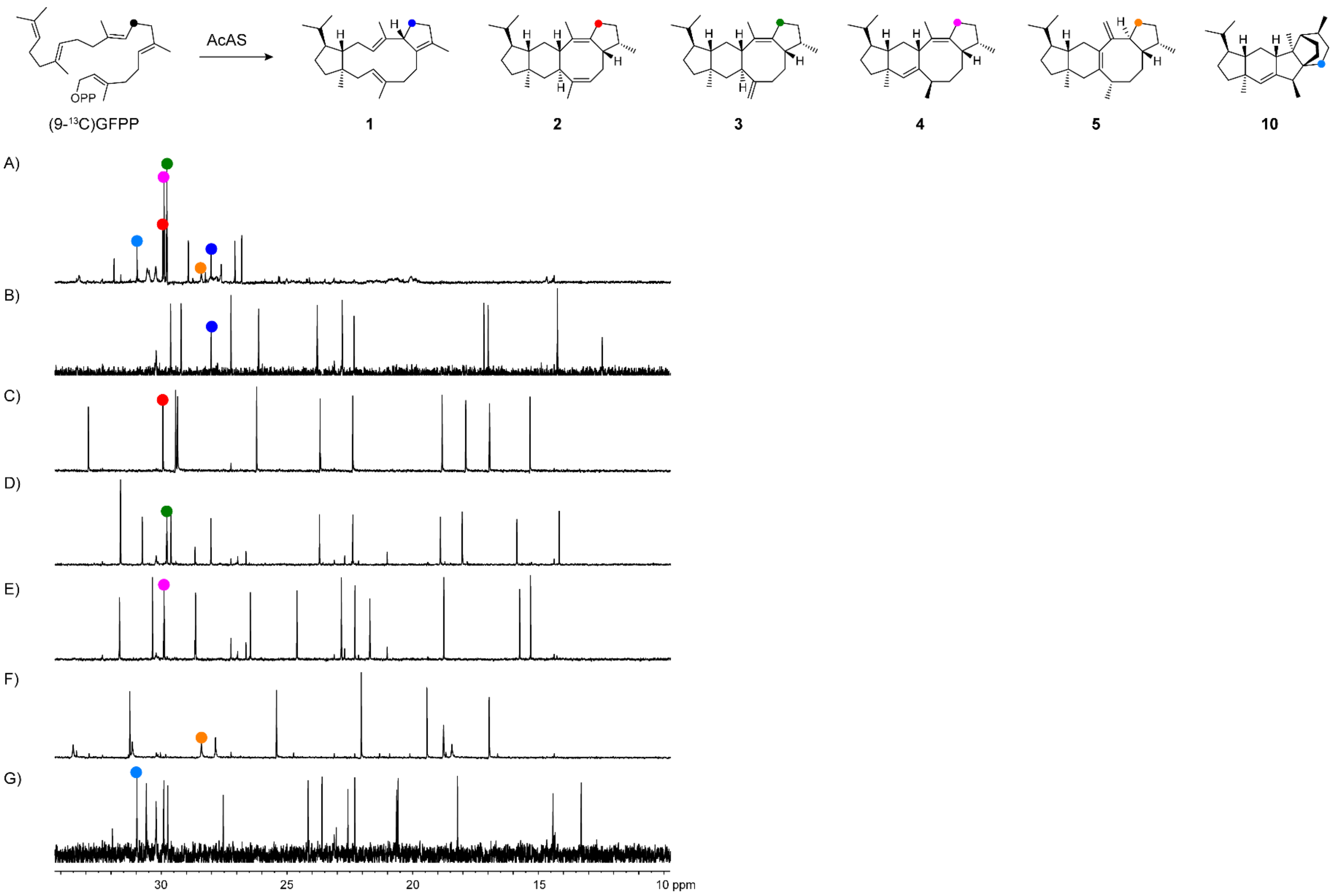

Figure S79. Partial ^{13}C-NMR spectra showing the regions for C9 of A) the mixture of labelled **1 – 5** and **10** obtained from (9-^{13}C)GFPP, and of the unlabelled compounds B) **1**, C) **2**, D) **3**, E) **4**, F) **5**, and G) **10**. Coloured dots at peaks correspond to the dots at the structures.

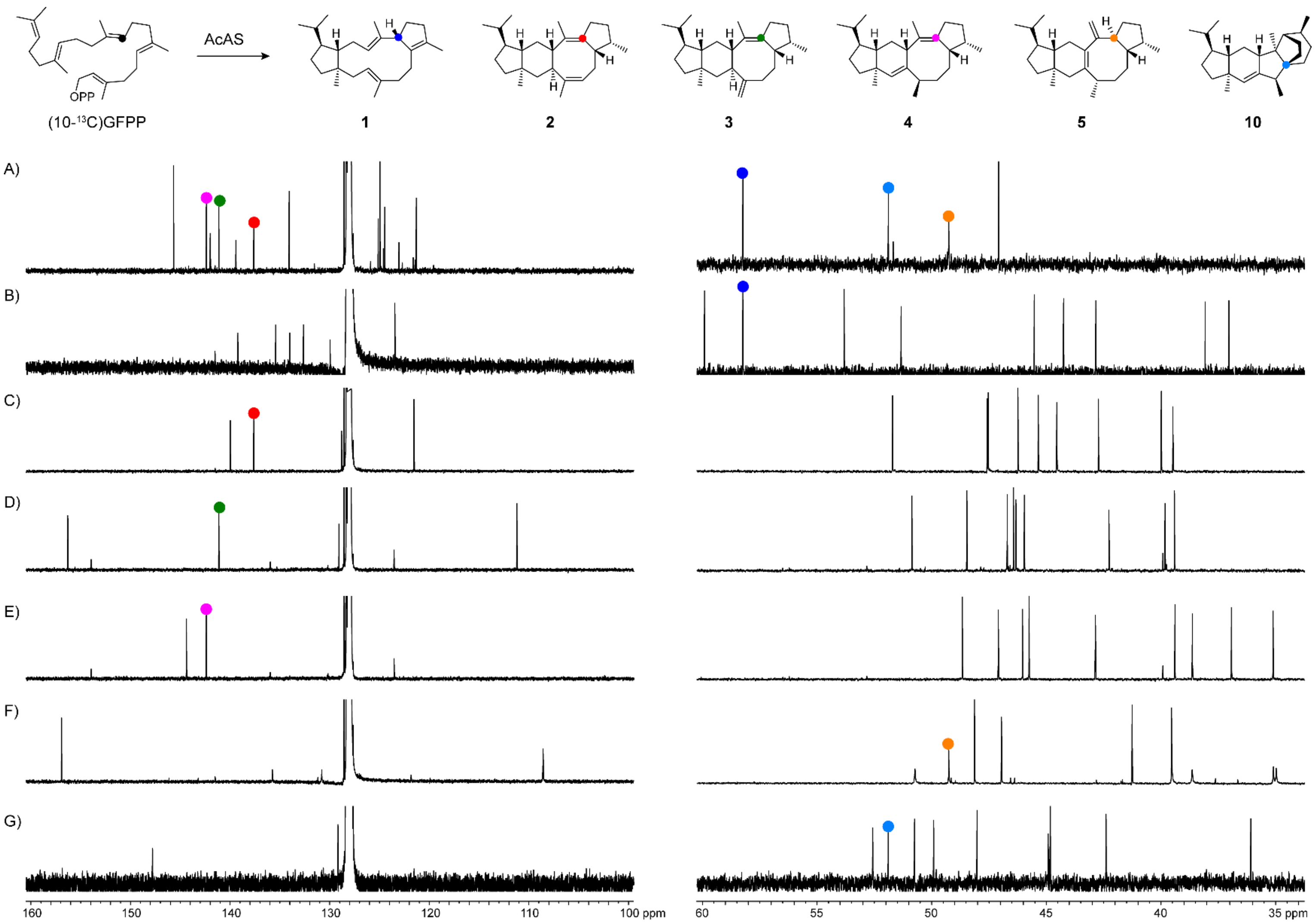

Figure S80. Partial ^{13}C-NMR spectra showing the regions for C10 of A) the mixture of labelled **1 – 5** and **10** obtained from (10-^{13}C)GFPP, and of the unlabelled compounds B) **1**, C) **2**, D) **3**, E) **4**, F) **5**, and G) **10**. Coloured dots at peaks correspond to the dots at the structures. Each of the parts A) – G) was produced from one ^{13}C-NMR spectrum.

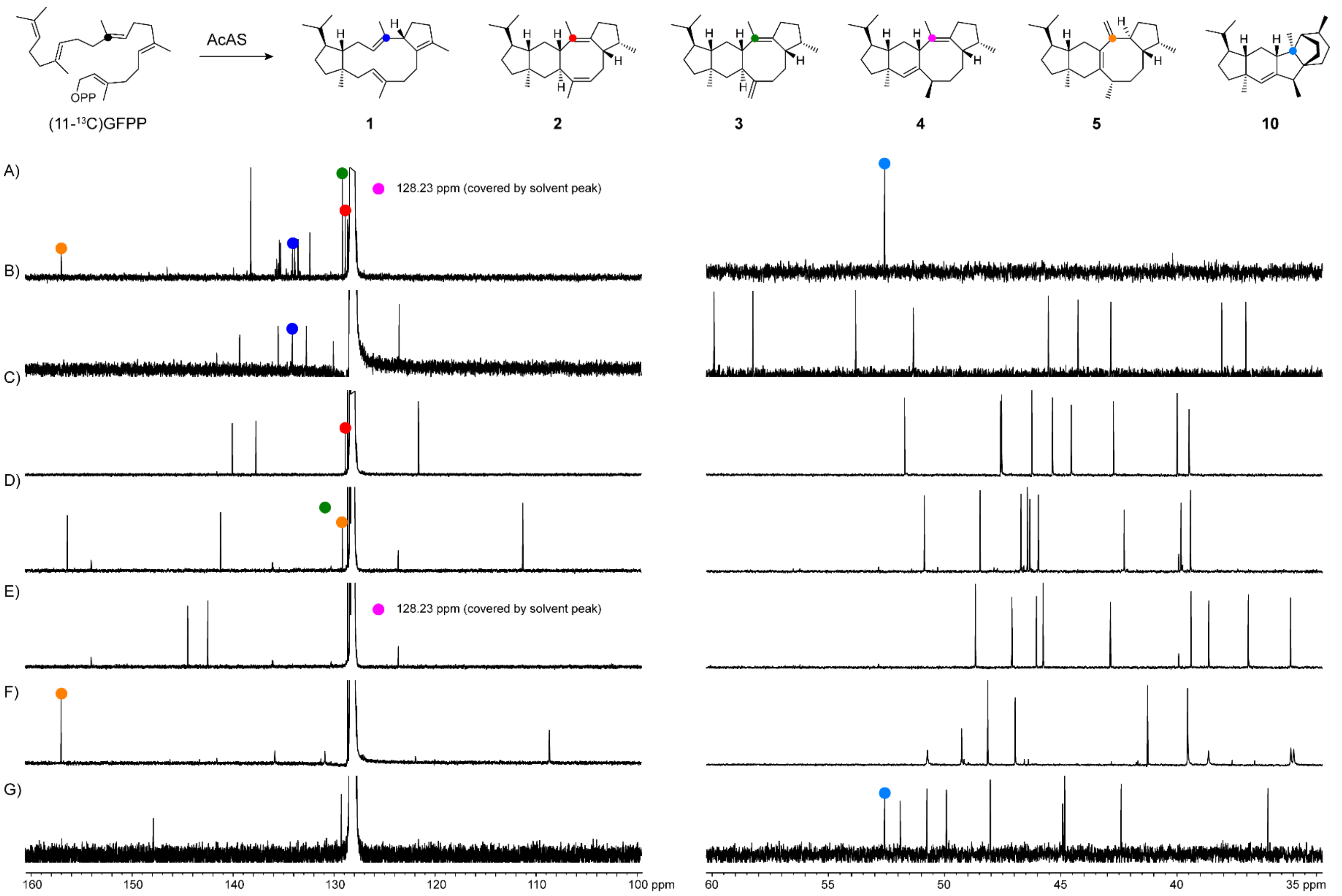

Figure S81. Partial ^{13}C-NMR spectra showing the regions for C11 of A) the mixture of labelled **1 – 5** and **10** obtained from (11-^{13}C)GFPP, and of the unlabelled compounds B) **1**, C) **2**, D) **3**, E) **4**, F) **5**, and G) **10**. Coloured dots at peaks correspond to the dots at the structures. Each of the parts A) – G) was produced from one ^{13}C-NMR spectrum.

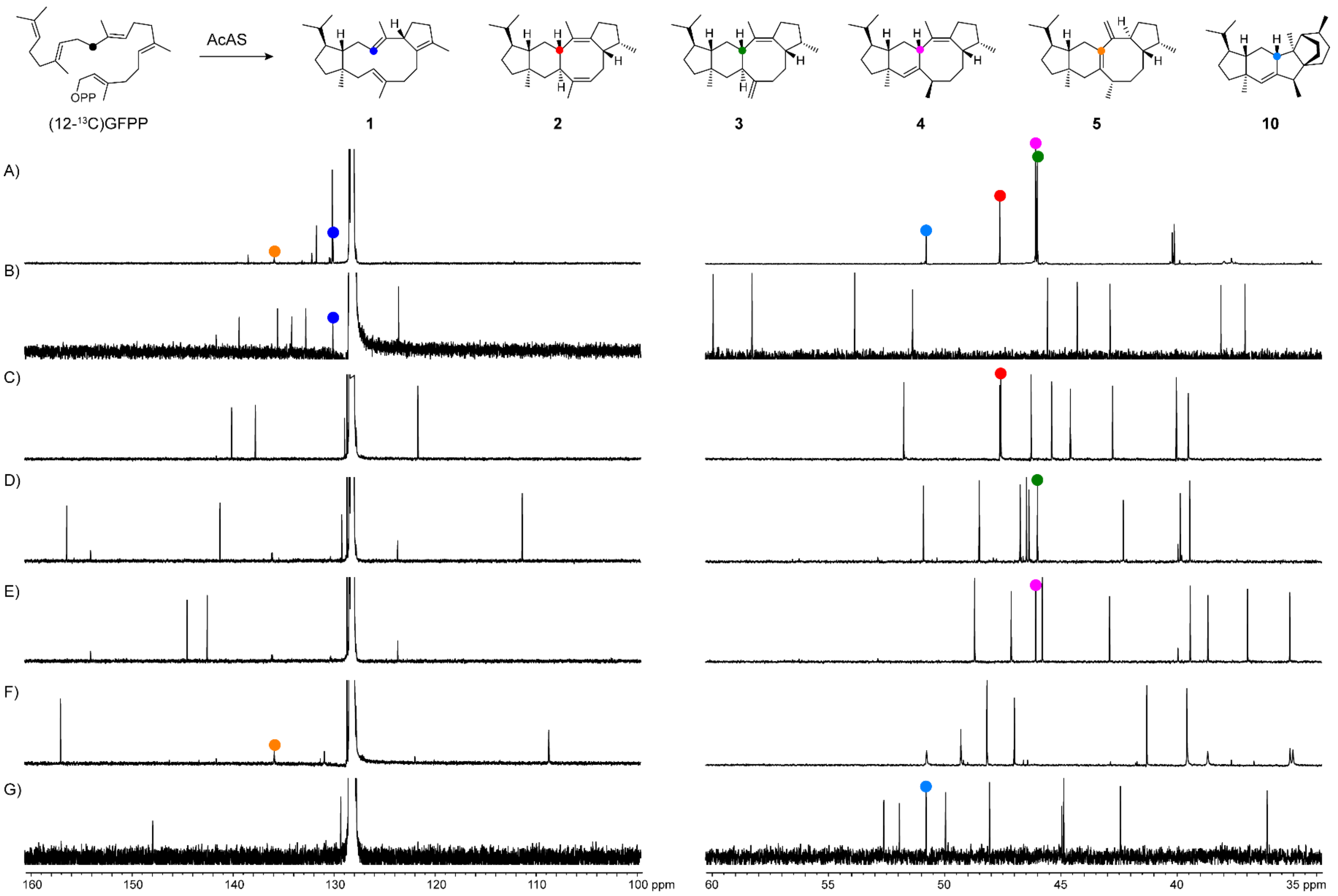

Figure S82. Partial ^{13}C-NMR spectra showing the regions for C12 of A) the mixture of labelled **1 – 5** and **10** obtained from (12-^{13}C)GFPP, and of the unlabelled compounds B) **1**, C) **2**, D) **3**, E) **4**, F) **5**, and G) **10**. Coloured dots at peaks correspond to the dots at the structures. Each of the parts A) – G) was produced from one ^{13}C-NMR spectrum.

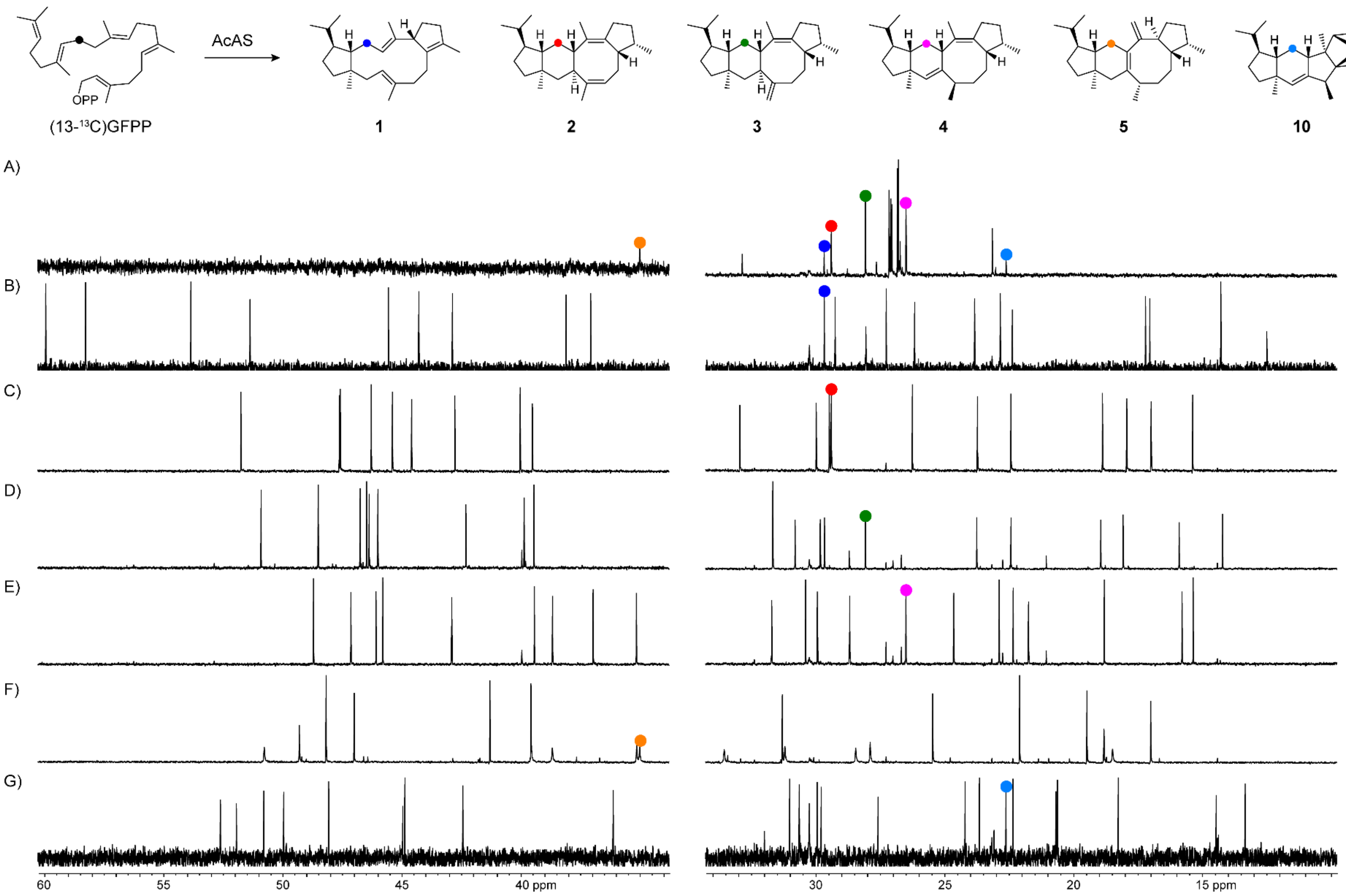

Figure S83. Partial ^{13}C-NMR spectra showing the regions for C13 of A) the mixture of labelled **1 – 5** and **10** obtained from (13-^{13}C)GFPP, and of the unlabelled compounds B) **1**, C) **2**, D) **3**, E) **4**, F) **5**, and G) **10**. Coloured dots at peaks correspond to the dots at the structures. Each of the parts A) – G) was produced from one ^{13}C-NMR spectrum.

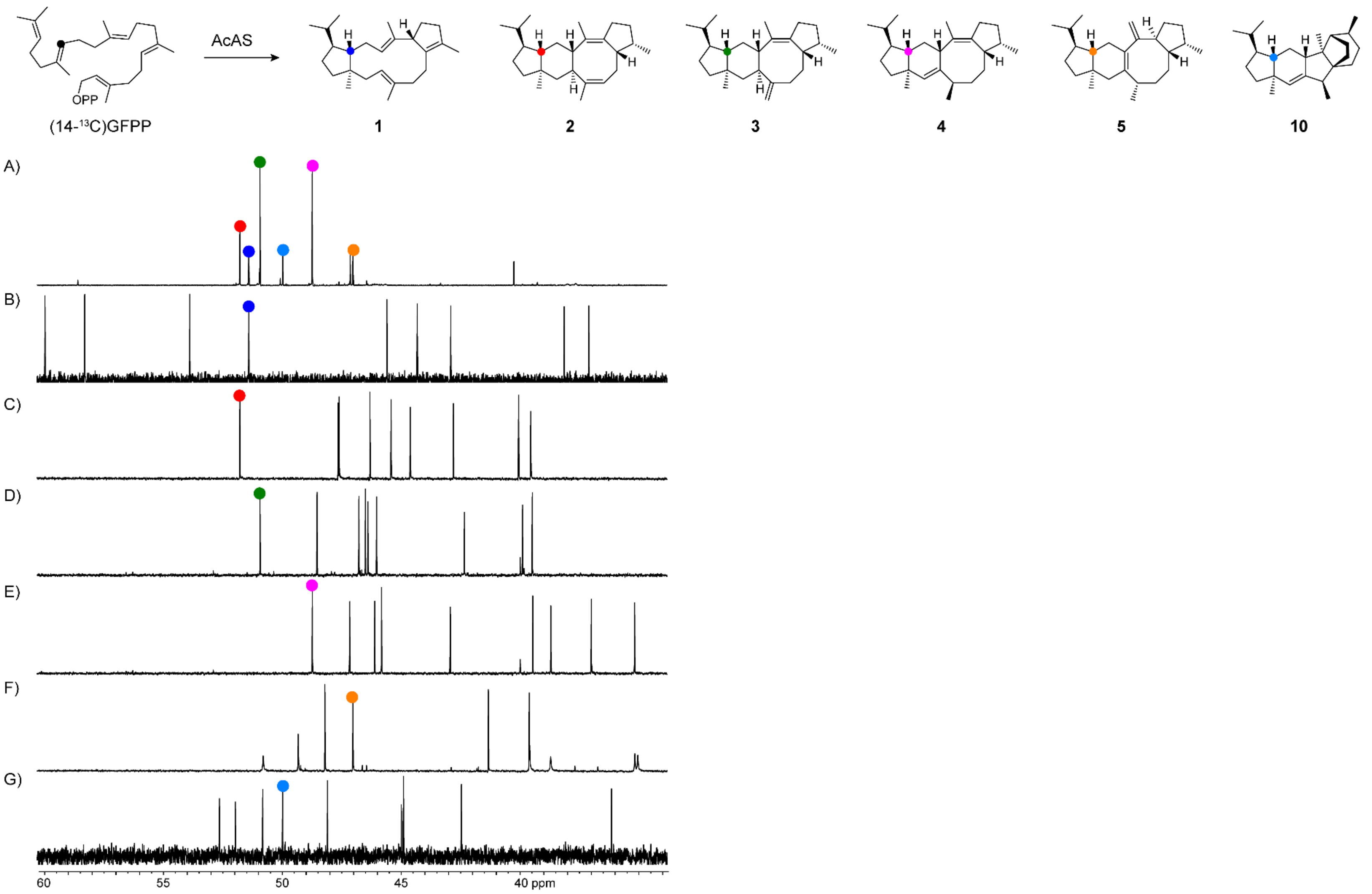

Figure S84. Partial ^{13}C-NMR spectra showing the regions for C14 of A) the mixture of labelled **1 – 5** and **10** obtained from (14-^{13}C)GFPP, and of the unlabelled compounds B) **1**, C) **2**, D) **3**, E) **4**, F) **5**, and G) **10**. Coloured dots at peaks correspond to the dots at the structures.

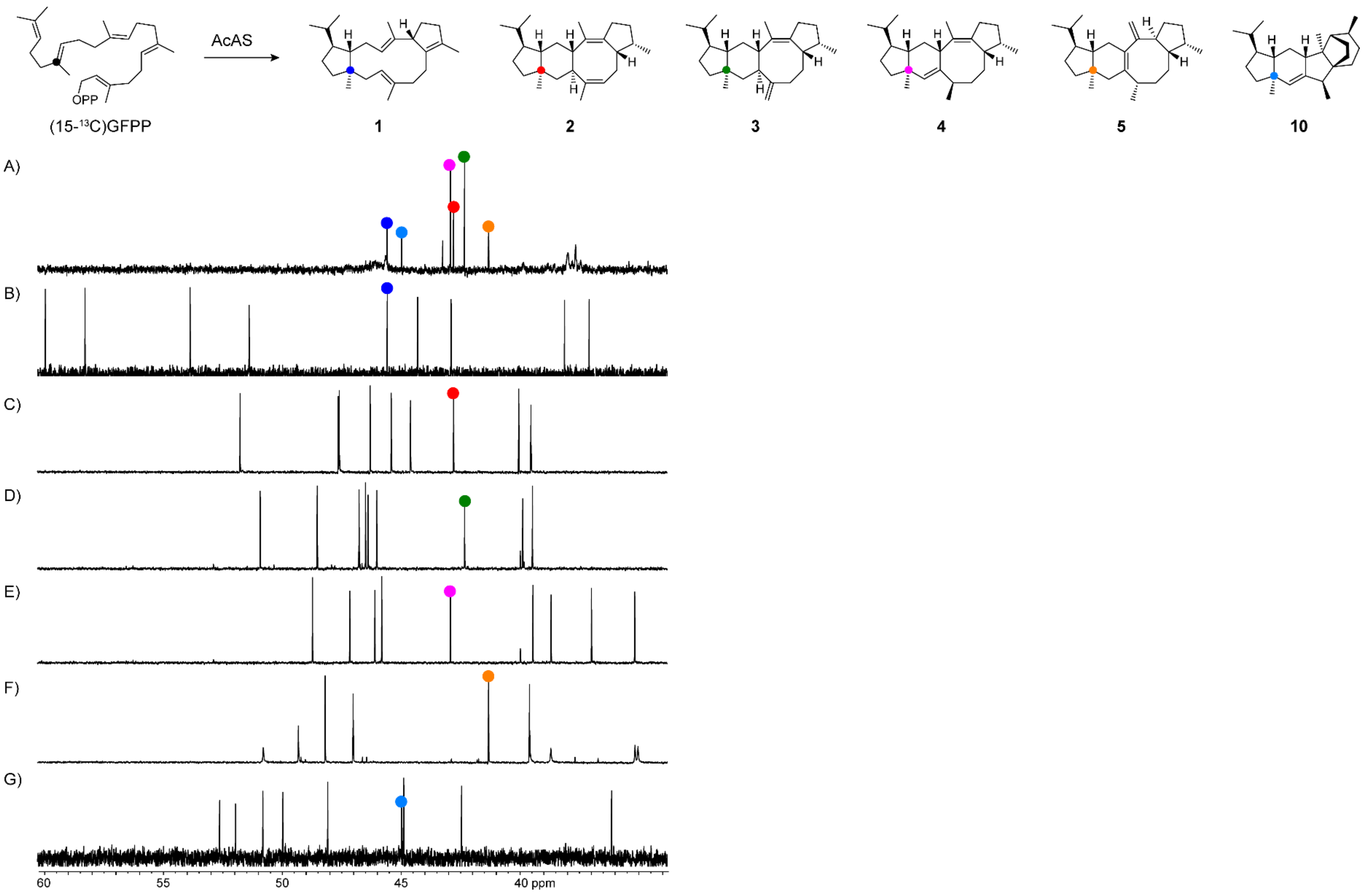

Figure S85. Partial ^{13}C-NMR spectra showing the regions for C15 of A) the mixture of labelled **1 – 5** and **10** obtained from (15-^{13}C)GFPP, and of the unlabelled compounds B) **1**, C) **2**, D) **3**, E) **4**, F) **5**, and G) **10**. Coloured dots at peaks correspond to the dots at the structures.

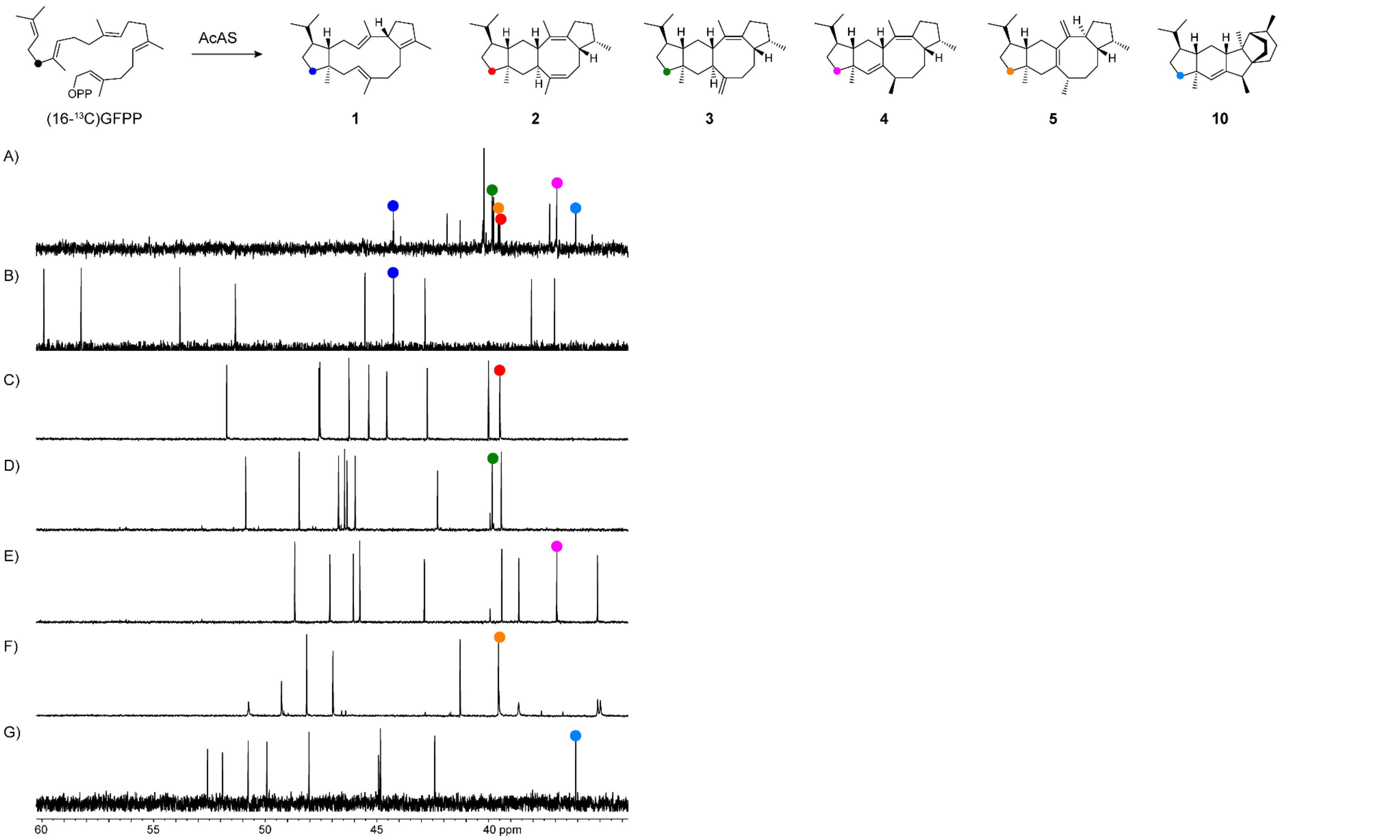

Figure S86. Partial ^{13}C-NMR spectra showing the regions for C16 of A) the mixture of labelled **1 – 5** and **10** obtained from (16-^{13}C)GFPP, and of the unlabelled compounds B) **1**, C) **2**, D) **3**, E) **4**, F) **5**, and G) **10**. Coloured dots at peaks correspond to the dots at the structures.

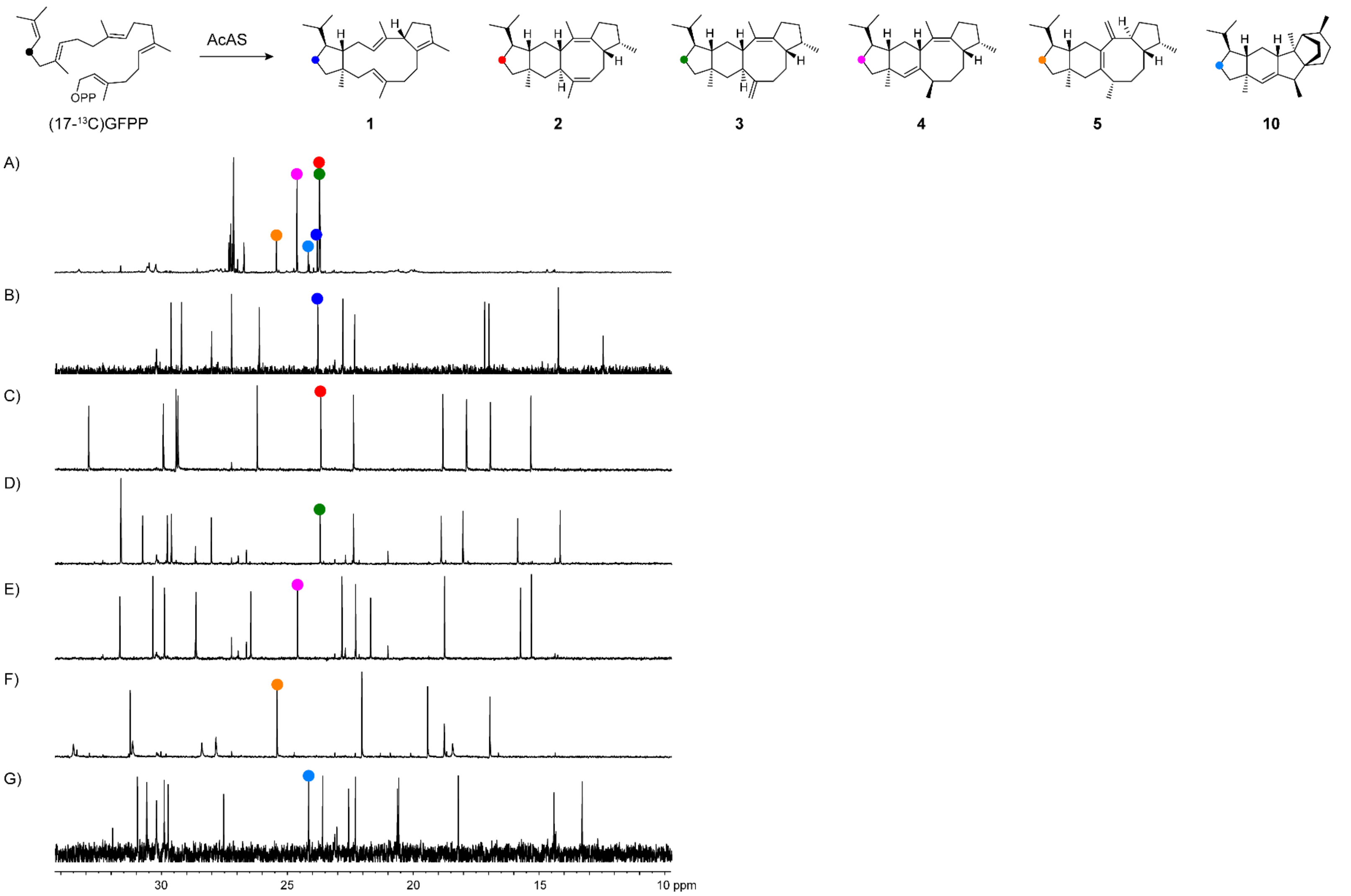

Figure S87. Partial ^{13}C-NMR spectra showing the regions for C17 of A) the mixture of labelled **1 – 5** and **10** obtained from (17-^{13}C)GFPP, and of the unlabelled compounds B) **1**, C) **2**, D) **3**, E) **4**, F) **5**, and G) **10**. Coloured dots at peaks correspond to the dots at the structures.

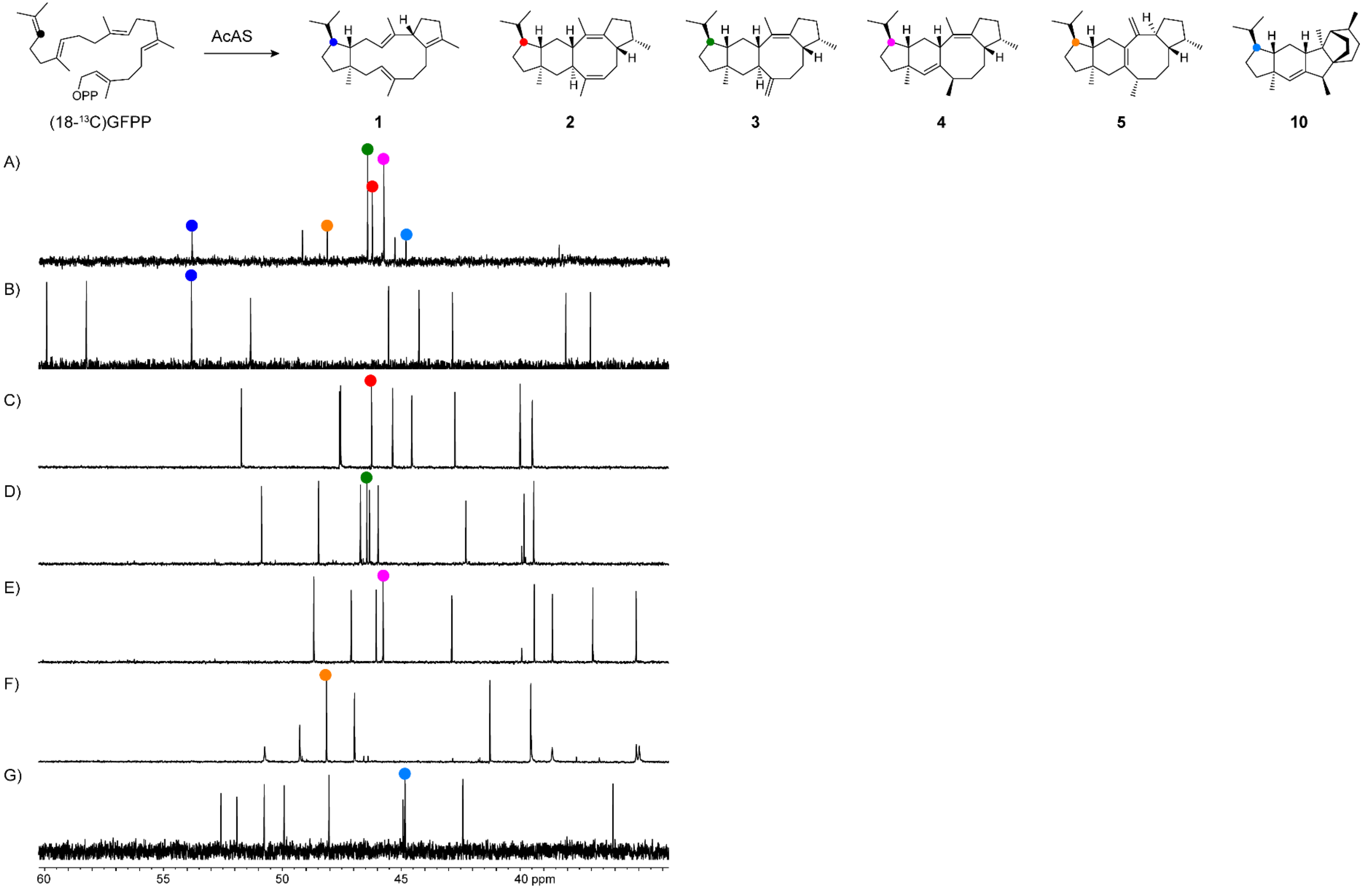

Figure S88. Partial ^{13}C-NMR spectra showing the regions for C18 of A) the mixture of labelled **1 – 5** and **10** obtained from (18-^{13}C)GFPP, and of the unlabelled compounds B) **1**, C) **2**, D) **3**, E) **4**, F) **5**, and G) **10**. Coloured dots at peaks correspond to the dots at the structures.

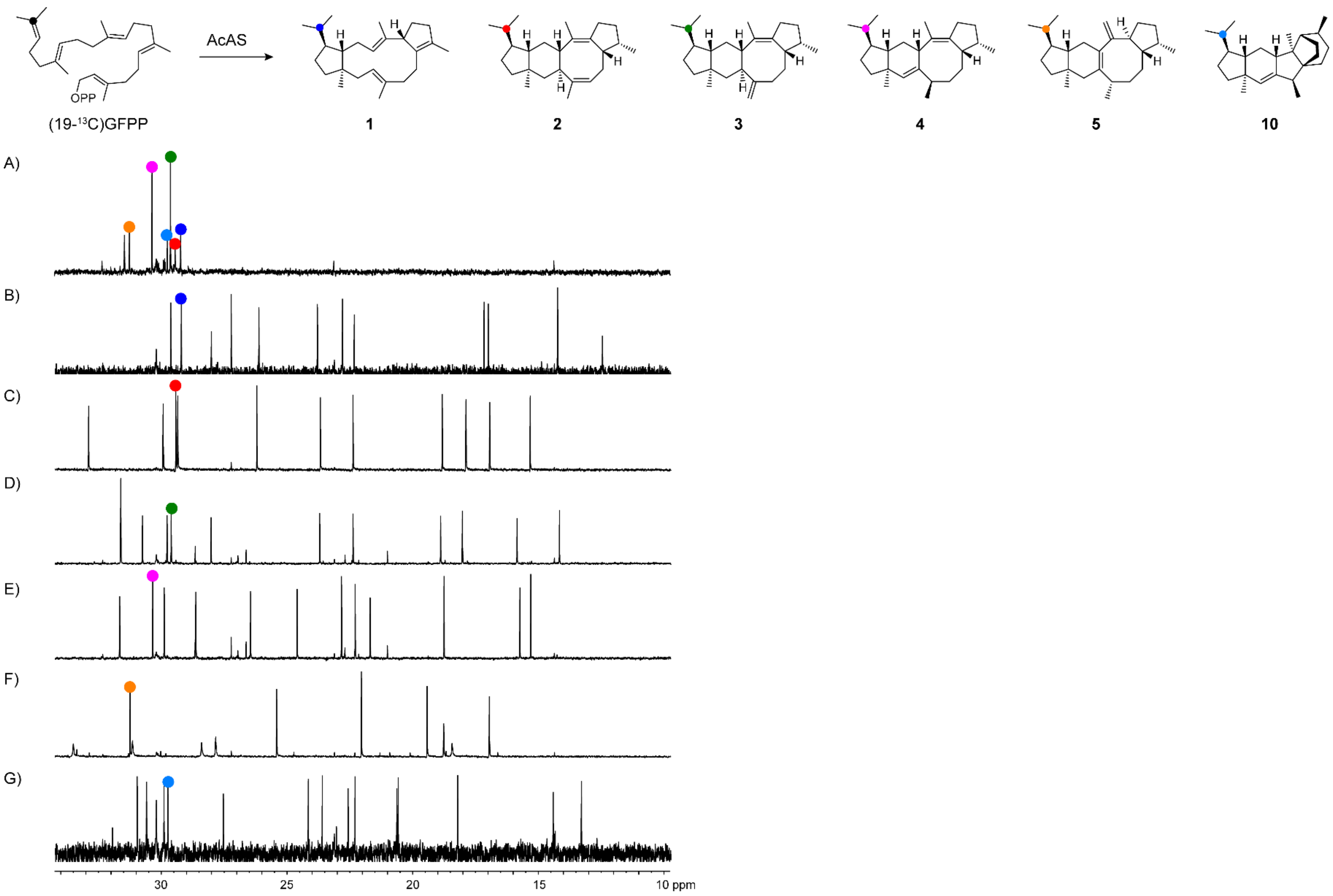

Figure S89. Partial ^{13}C-NMR spectra showing the regions for C19 of A) the mixture of labelled **1 – 5** and **10** obtained from (19-^{13}C)GFPP, and of the unlabelled compounds B) **1**, C) **2**, D) **3**, E) **4**, F) **5**, and G) **10**. Coloured dots at peaks correspond to the dots at the structures.

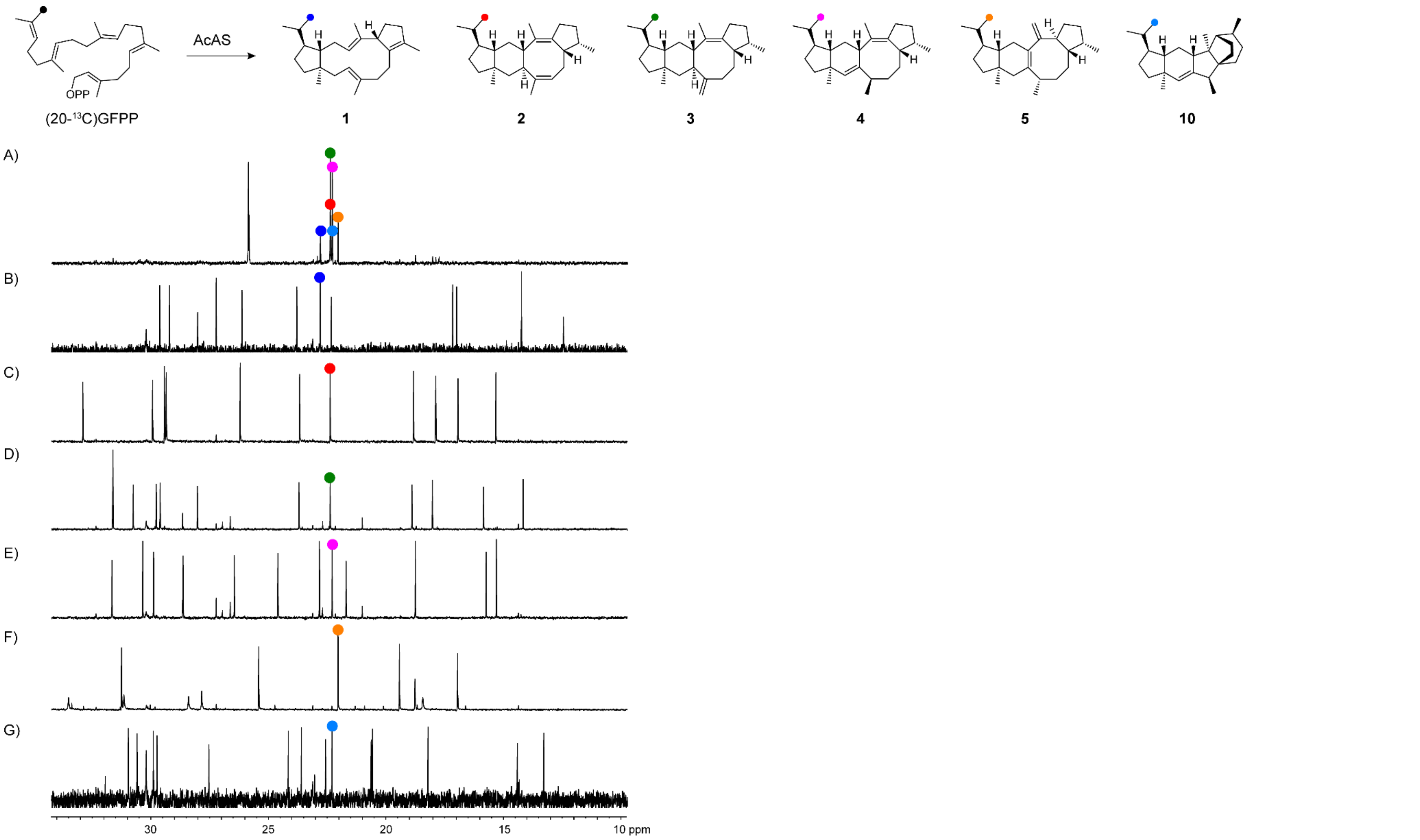

Figure S90. Partial ^{13}C-NMR spectra showing the regions for C20 of A) the mixture of labelled **1** – **5** and **10** obtained from (20-^{13}C)GFPP, and of the unlabelled compounds B) **1**, C) **2**, D) **3**, E) **4**, F) **5**, and G) **10**. Coloured dots at peaks correspond to the dots at the structures.

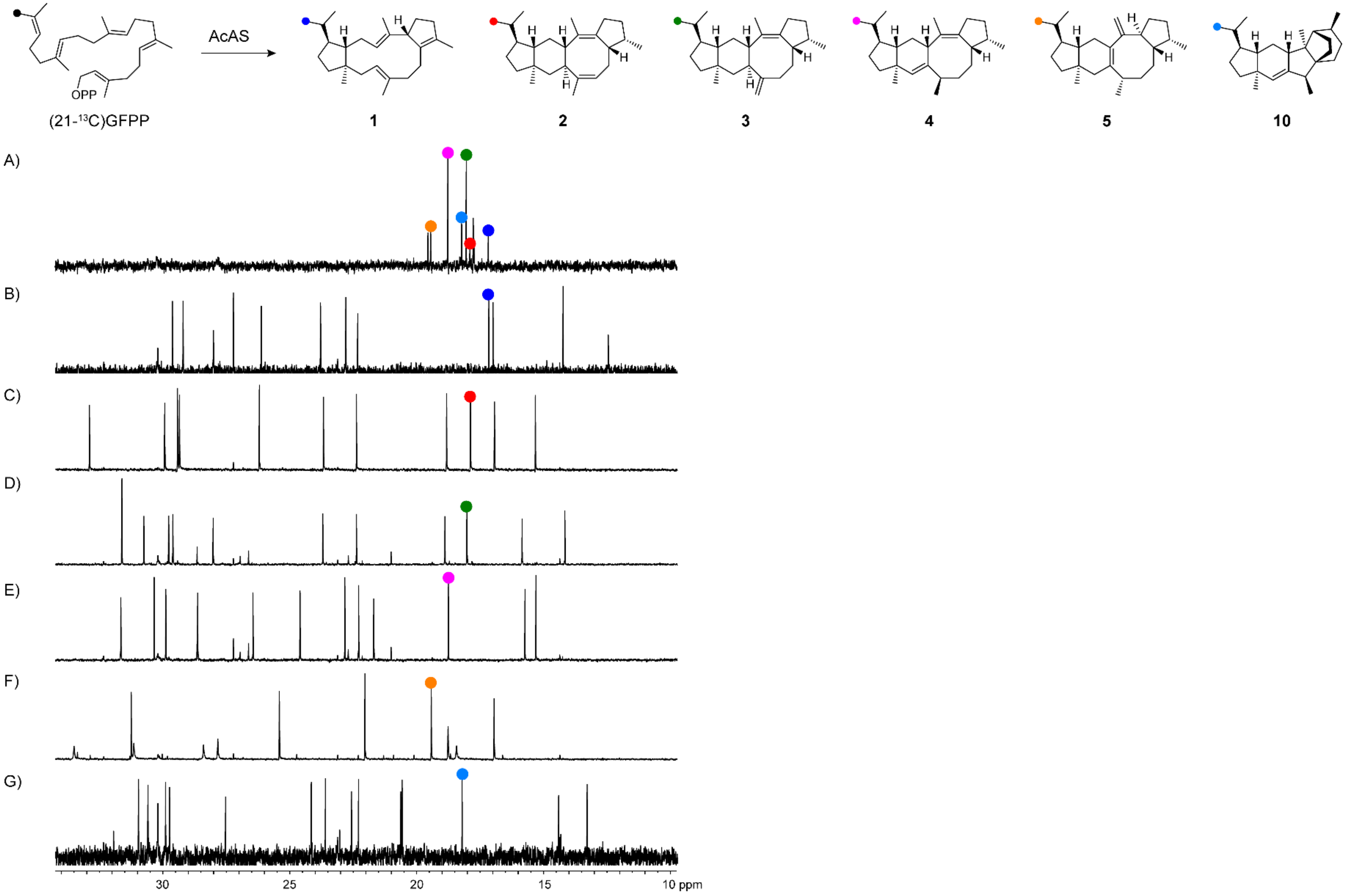

Figure S91. Partial ^{13}C-NMR spectra showing the regions for C21 of A) the mixture of labelled **1 – 5** and **10** obtained from (21-^{13}C)GFPP, and of the unlabelled compounds B) **1**, C) **2**, D) **3**, E) **4**, F) **5**, and G) **10**. Coloured dots at peaks correspond to the dots at the structures.

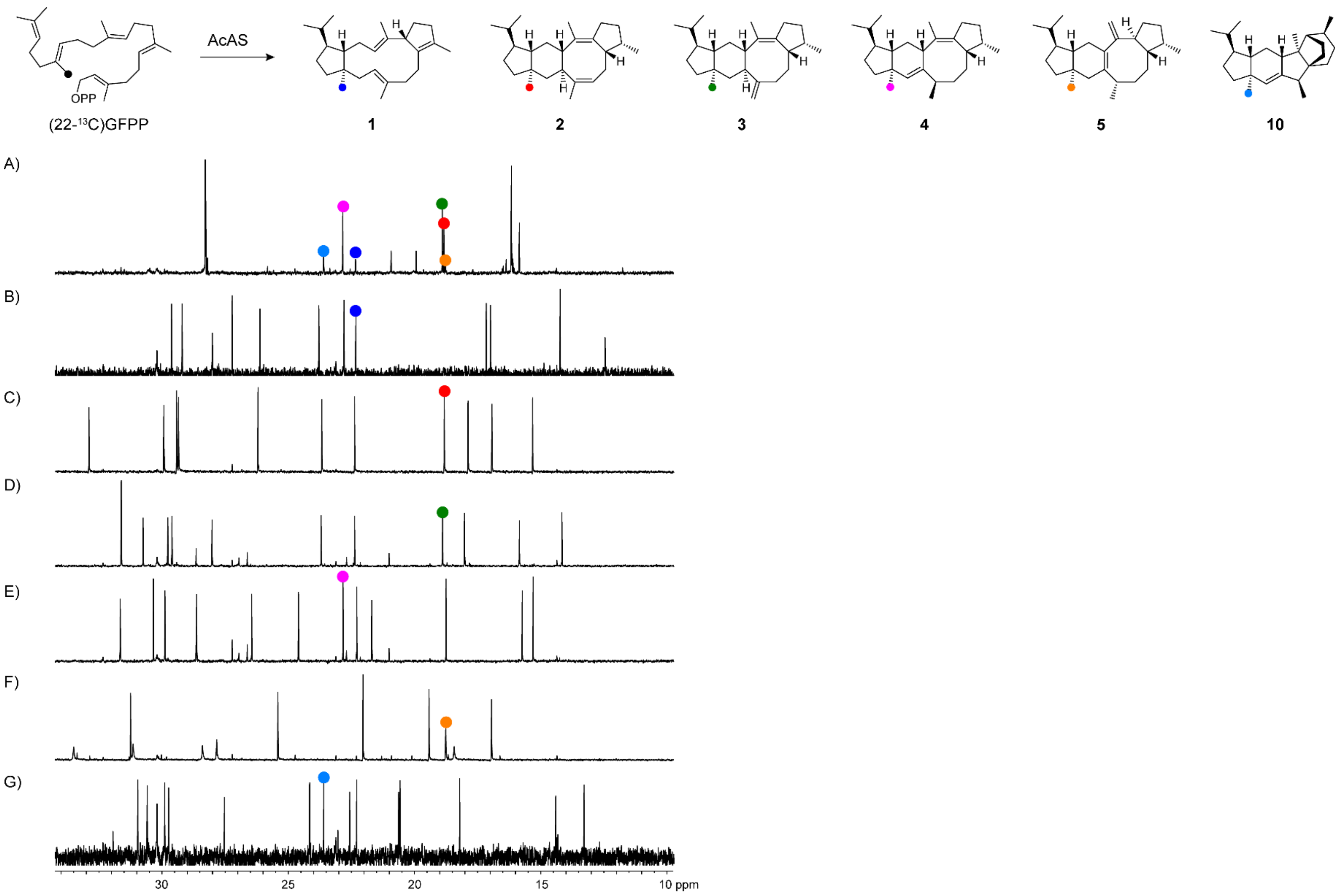

Figure S92. Partial ^{13}C-NMR spectra showing the regions for C22 of A) the mixture of labelled **1 – 5** and **10** obtained from (22-^{13}C)GFPP, and of the unlabelled compounds B) **1**, C) **2**, D) **3**, E) **4**, F) **5**, and G) **10**. Coloured dots at peaks correspond to the dots at the structures.

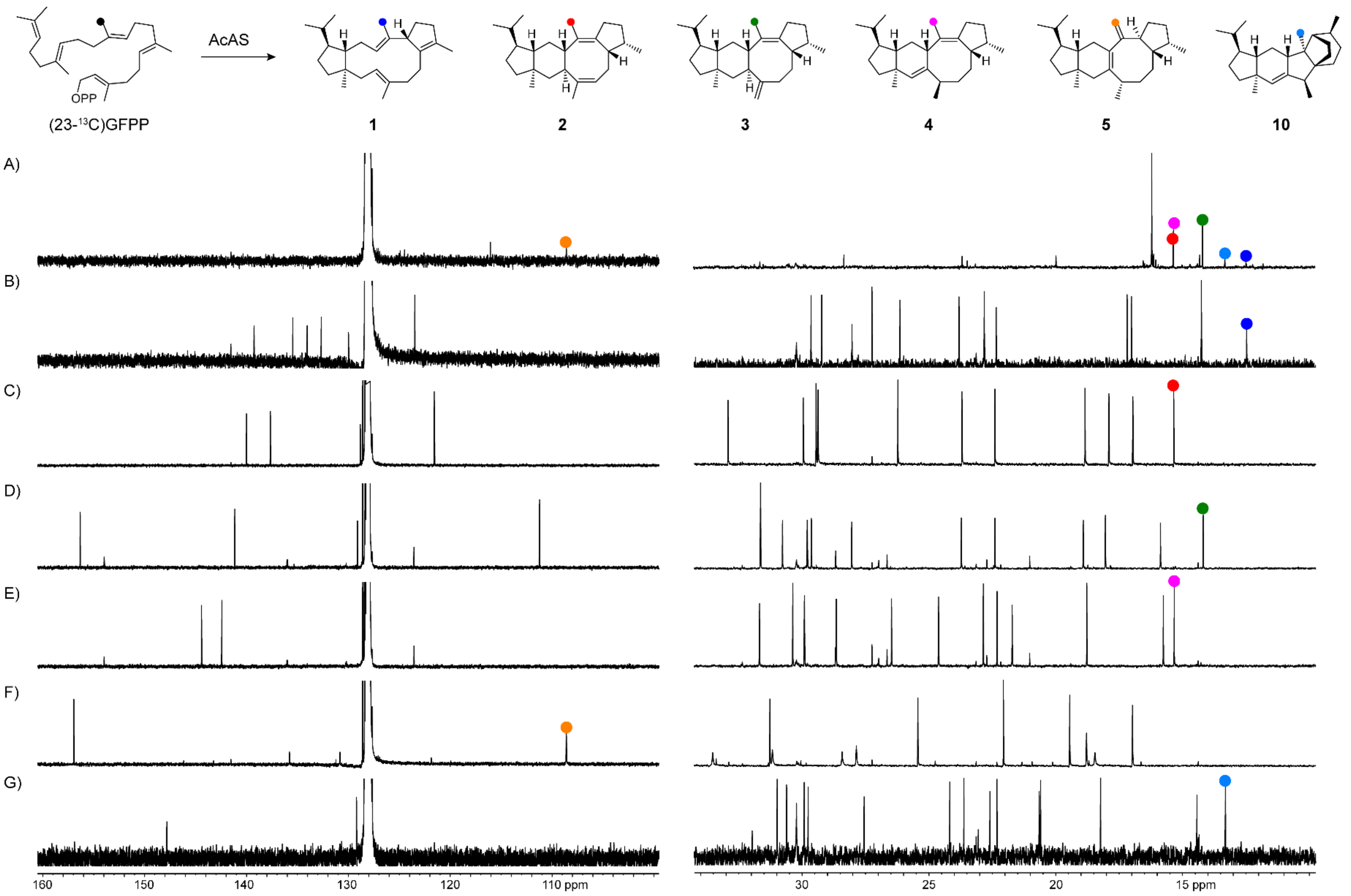

Figure S93. Partial ^{13}C-NMR spectra showing the regions for C23 of A) the mixture of labelled **1 – 5** and **10** obtained from (23-^{13}C)GFPP, and of the unlabelled compounds B) **1**, C) **2**, D) **3**, E) **4**, F) **5**, and G) **10**. Coloured dots at peaks correspond to the dots at the structures. Each of the parts A) – G) was produced from one ^{13}C-NMR spectrum.

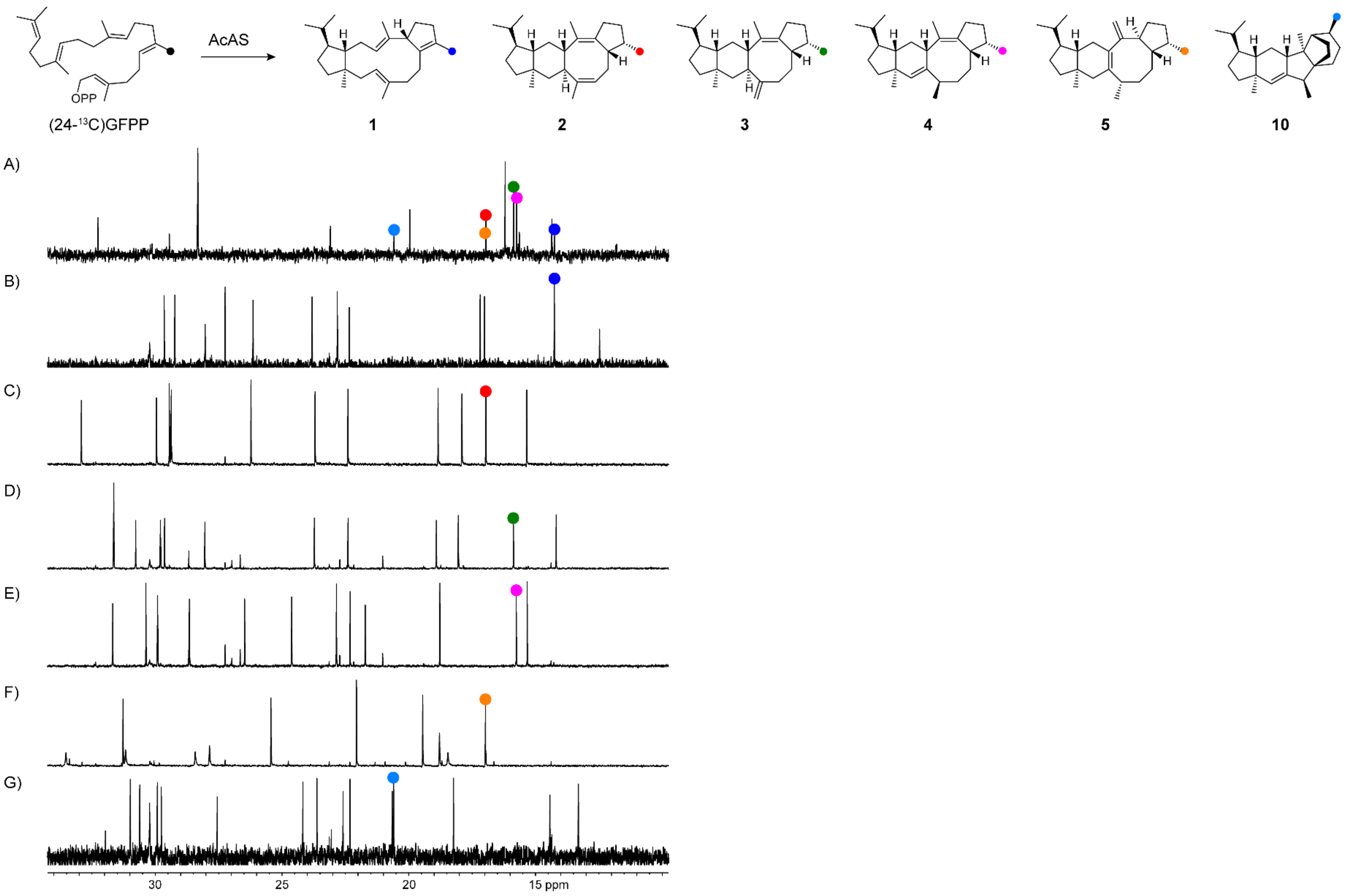

Figure S94. Partial ^{13}C-NMR spectra showing the regions for C24 of A) the mixture of labelled **1 – 5** and **10** obtained from (24-^{13}C)GFPP, and of the unlabelled compounds B) **1**, C) **2**, D) **3**, E) **4**, F) **5**, and G) **10**. Coloured dots at peaks correspond to the dots at the structures.

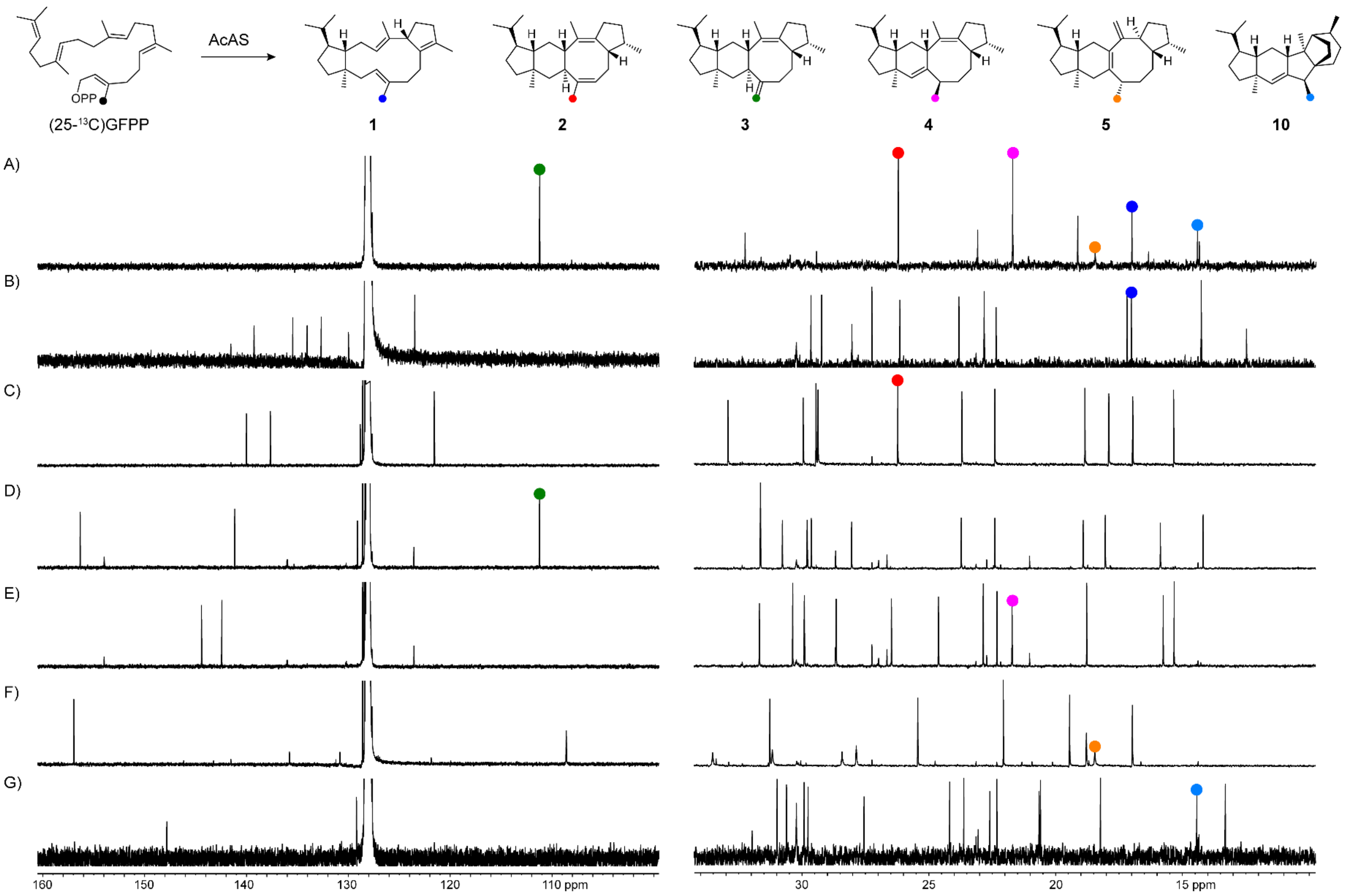

Figure S95. Partial ^{13}C-NMR spectra showing the regions for C25 of A) the mixture of labelled **1 – 5** and **10** obtained from (25-^{13}C)GFPP, and of the unlabelled compounds B) **1**, C) **2**, D) **3**, E) **4**, F) **5**, and G) **10**. Coloured dots at peaks correspond to the dots at the structures. Each of the parts A) – G) was produced from one ^{13}C-NMR spectrum.

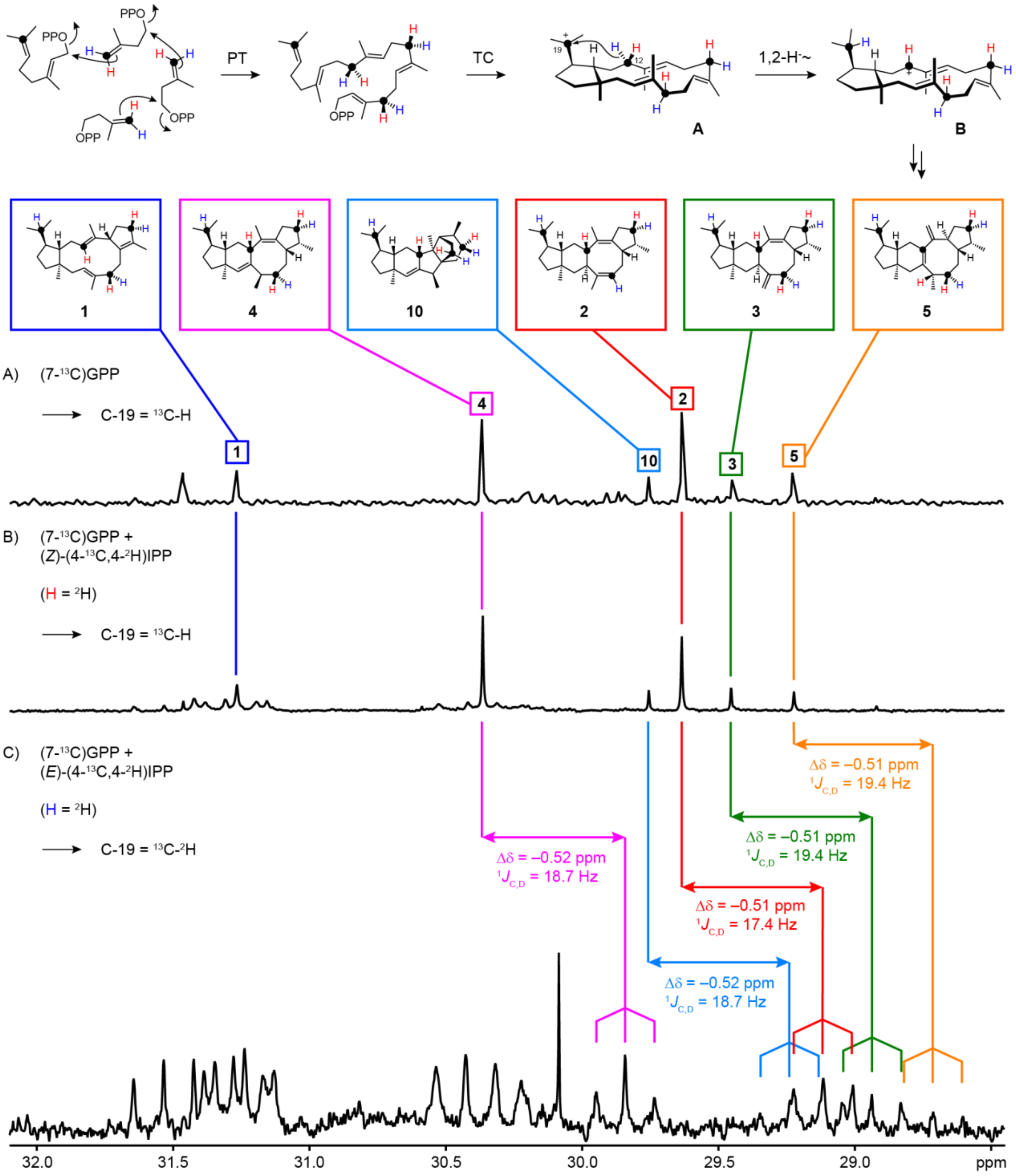

Figure S96. The 1,5-hydride shift from **A** to **B** for the AcAS products by ^{13}C-NMR. Partial ^{13}C-NMR spectra showing the region for C19 of labelled **1** – **5** and **10** obtained with AcAS from A) (7-^{13}C)GPP and unlabelled IPP, B) (7-^{13}C)GPP and (*Z*)-(4-^{13}C,4-^{2}H)IPP (red H = ^{2}H), and C) (7-^{13}C)GPP and (*E*)-(4-^{13}C,4-^{2}H)IPP (blue H = ^{2}H). The upfield shifted triplets in C) indicate a direct ^{13}C-^{2}H bond for C19 of **2** – **5** and **10**. Black dots indicate ^{13}C-labelled carbons.

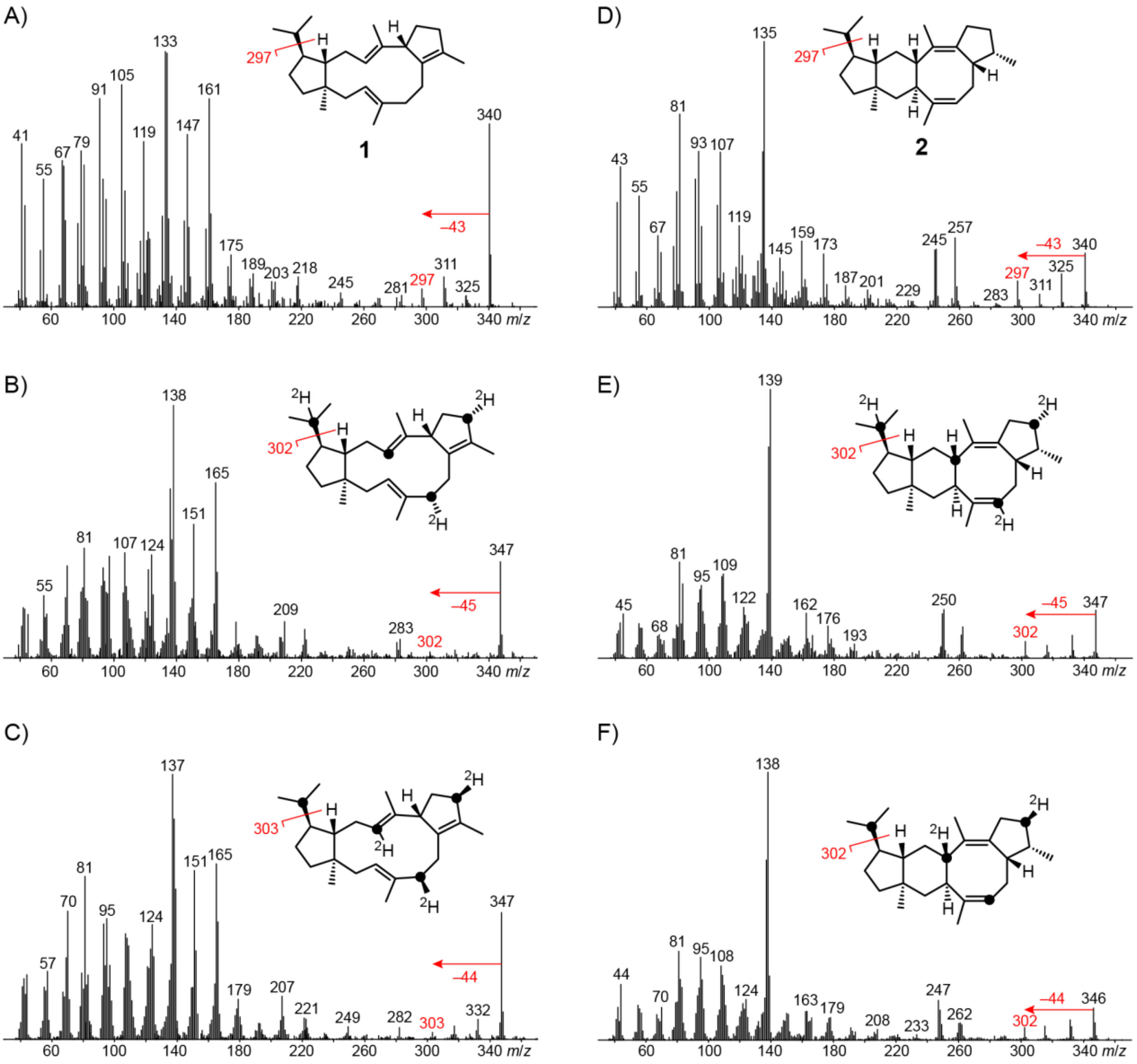

Figure S97. The 1,5-hydride shift from **A** to **B** for the AcAS products **1** and **2** by MS. EI mass spectra of A) unlabelled **1**, B) labelled **1** obtained from (7-^{13}C)GPP and (*E*)-(4-^{13}C,4-^{2}H)IPP, C) labelled **1** obtained from (7-^{13}C)GPP and (*Z*)-(4-^{13}C,4-^{2}H)IPP, D) unlabelled **2**, E) labelled **2** obtained from (7-^{13}C)GPP and (*E*)-(4-^{13}C,4-^{2}H)IPP, and F) labelled **2** obtained from (7-^{13}C)GPP and (*Z*)-(4-^{13}C,4-^{2}H)IPP. The fragment ions arising by cleavage of the *i*Pr group indicate the shift of deuterium to C19 in B) and D). Black dots indicate ^{13}C-labelled carbons.

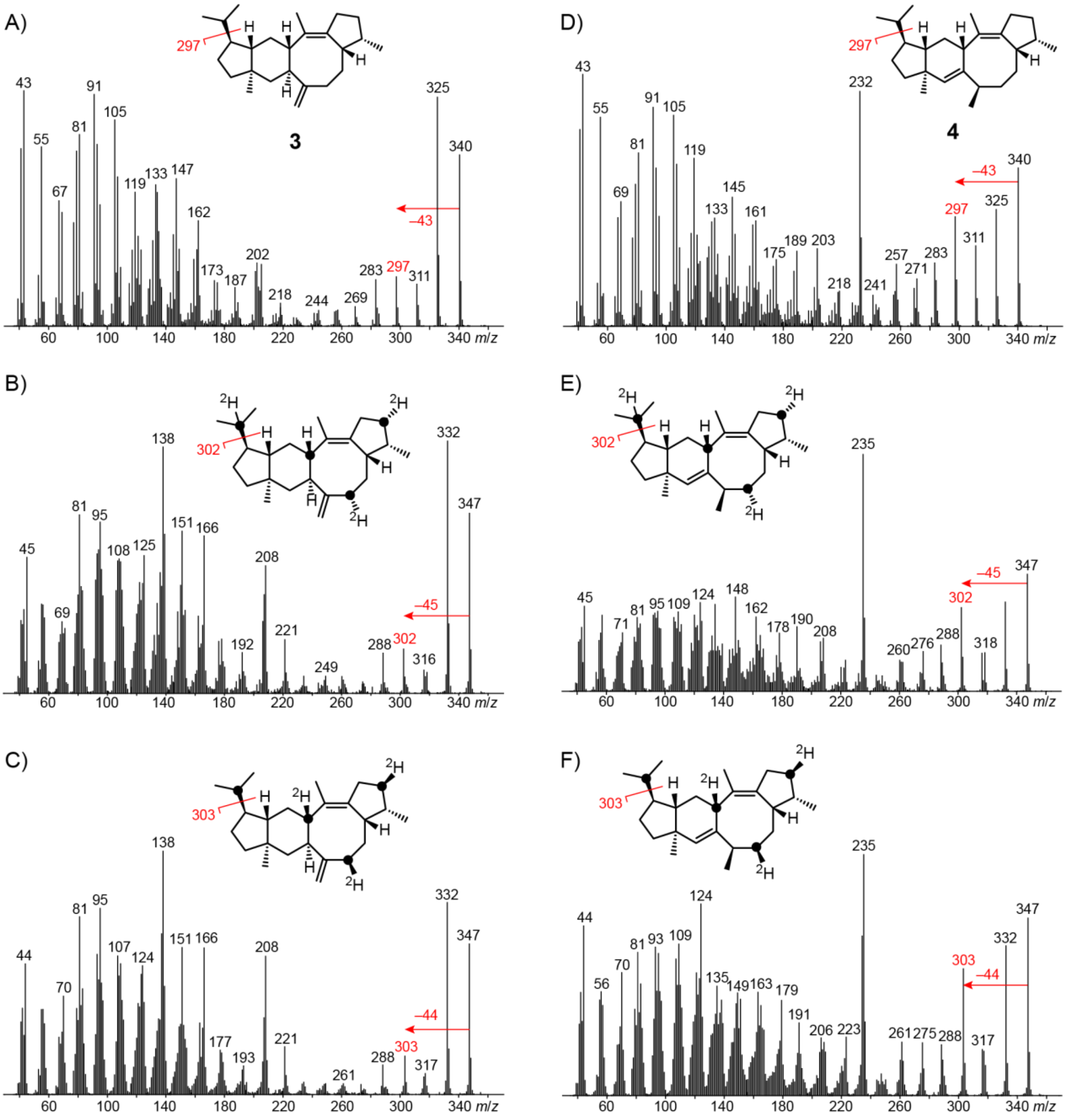

Figure S98. The 1,5-hydride shift from **A** to **B** for the AcAS products **3** and **4** by MS. EI mass spectra of A) unlabelled **3**, B) labelled **3** obtained from (7-^{13}C)GPP and (*E*)-(4-^{13}C,4-^{2}H)IPP, C) labelled **3** obtained from (7-^{13}C)GPP and (*Z*)-(4-^{13}C,4-^{2}H)IPP, D) unlabelled **4**, E) labelled **4** obtained from (7-^{13}C)GPP and (*E*)-(4-^{13}C,4-^{2}H)IPP, and F) labelled **4** obtained from (7-^{13}C)GPP and (*Z*)-(4-^{13}C,4-^{2}H)IPP. The fragment ions arising by cleavage of the *i*Pr group indicate the shift of deuterium to C19 in B) and D). Black dots indicate ^{13}C-labelled carbons.

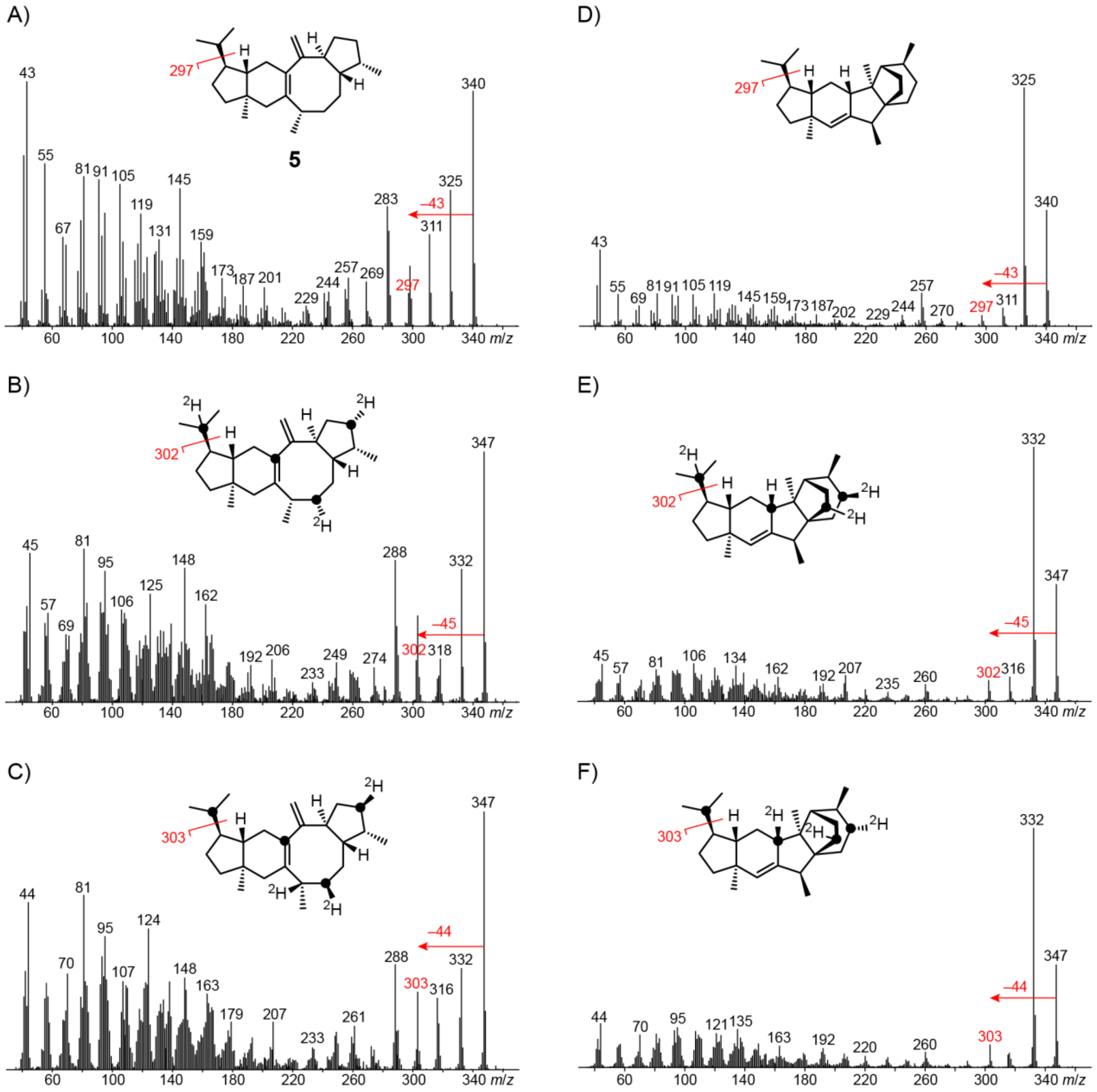

Figure S99. The 1,5-hydride shift from **A** to **B** for the AcAS products **5** and **10** by MS. EI mass spectra of A) unlabelled **5**, B) labelled **5** obtained from (7-^{13}C)GPP and (*E*)-(4-^{13}C,4-^{2}H)IPP, C) labelled **5** obtained from (7-^{13}C)GPP and (*Z*)-(4-^{13}C,4-^{2}H)IPP, D) unlabelled **10**, E) labelled **10** obtained from (7-^{13}C)GPP and (*E*)-(4-^{13}C,4-^{2}H)IPP, and F) labelled **10** obtained from (7-^{13}C)GPP and (*Z*)-(4-^{13}C,4-^{2}H)IPP. The fragment ions arising by cleavage of the *i*Pr group indicate the shift of deuterium to C19 in B) and D). Black dots indicate ^{13}C-labelled carbons.

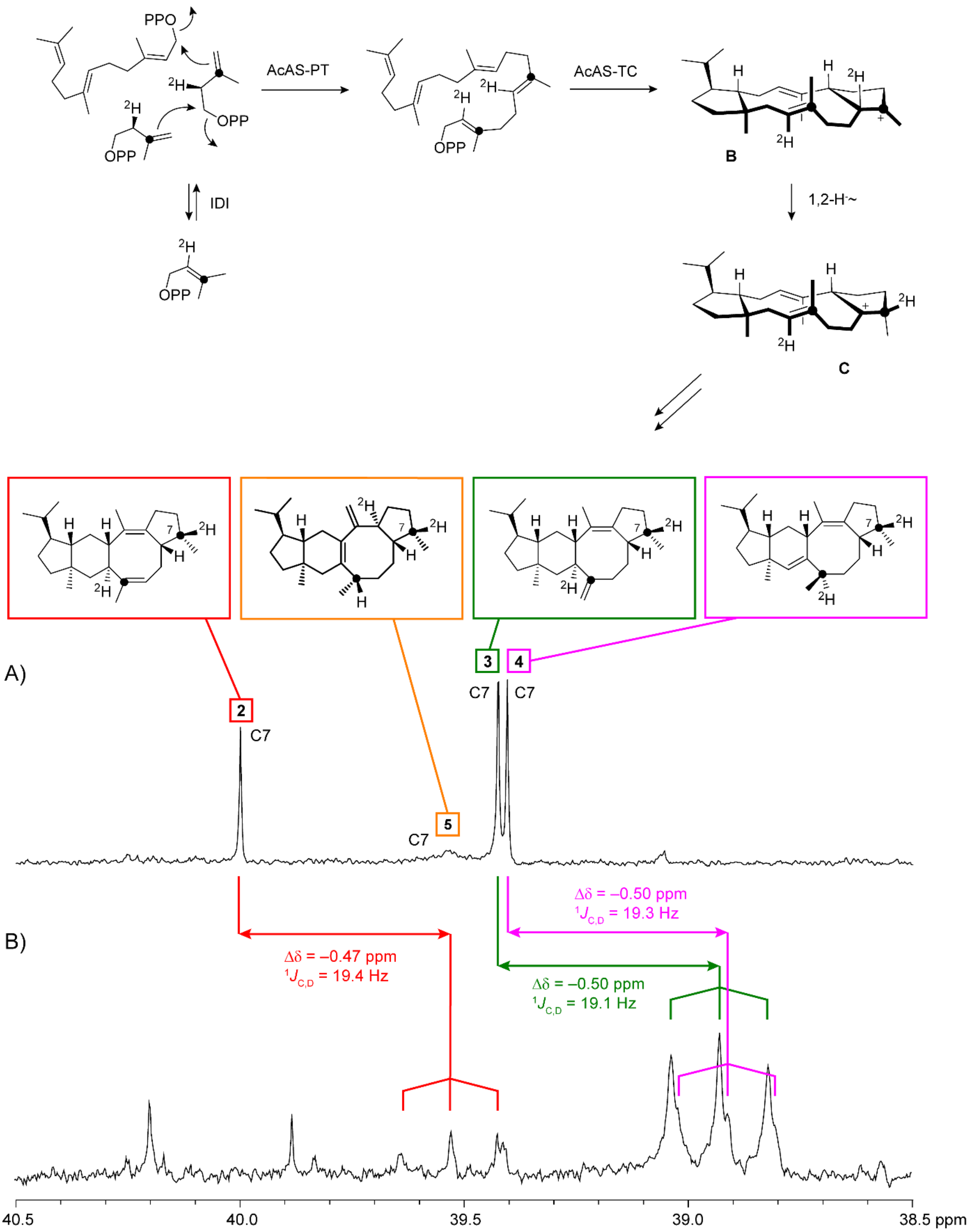

Figure S100. The 1,2-hydride shift from **B** to **C** for the AcAS products **2** – **4** by ^{13}C-NMR. Partial ^{13}C-NMR spectra showing the region for C7 of labelled **2** – **5** obtained with AcAS A) from (3-^{13}C)GGPP and IPP (only C7 is substituted by ^{13}C in the products), and B) from FPP and (3-^{13}C,2-^{2}H)DMAPP with addition of isopentenyl diphosphate isomerase (IDI). The upfield shifted triplets for C7 of **2** – **4** indicates a direct ^{13}C-^{2}H bond and thus supports the 1,2-hydride shift from **B** to **C**, while the broad peak for C7 of **5** prevented the detection of a corresponding triplet signal. Black dots indicate ^{13}C-labelled carbons.

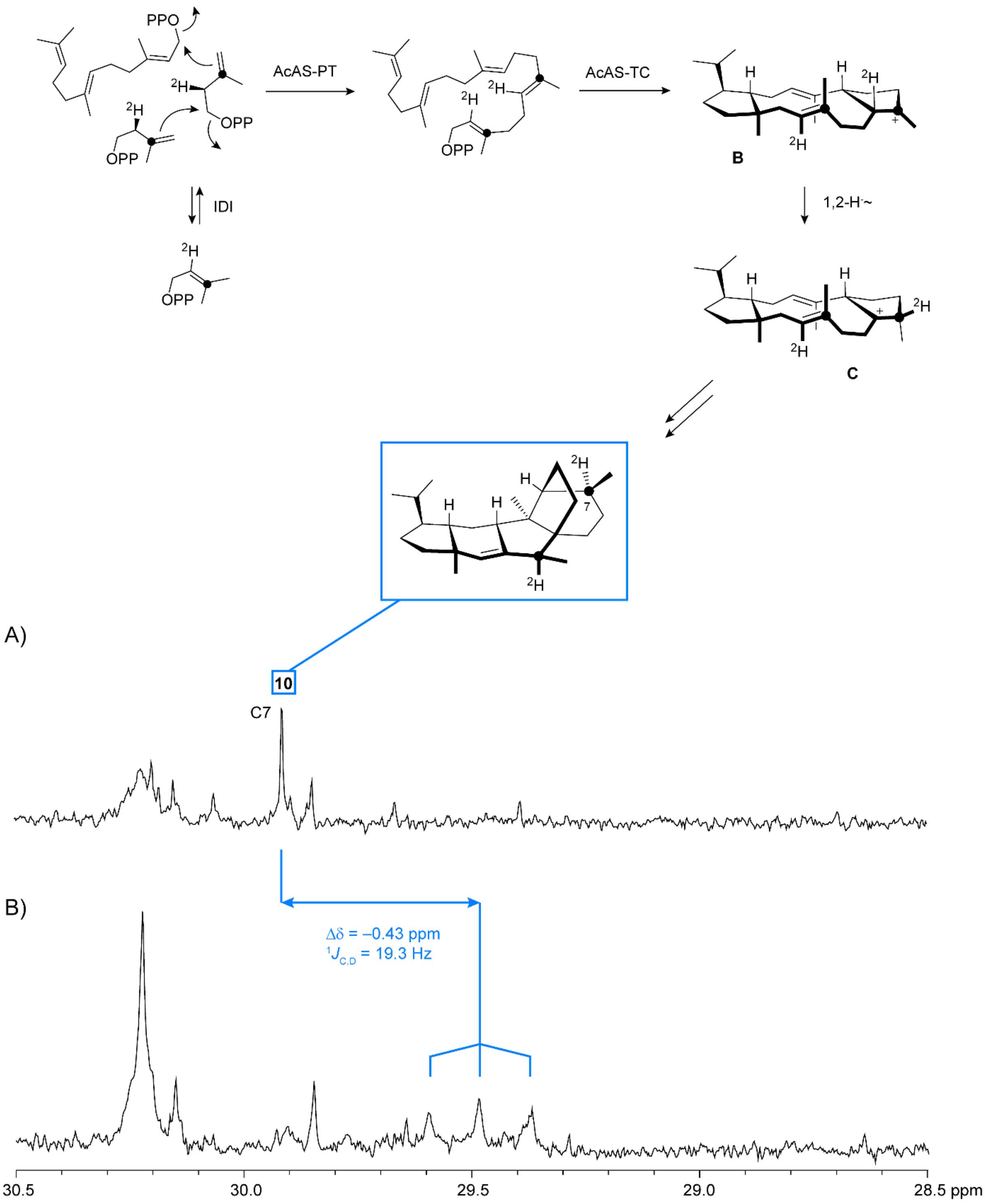

Figure S101. The 1,2-hydride shift from **B** to **C** for the AcAS product **10** by ^{13}C-NMR. Partial ^{13}C-NMR spectra showing the region for C7 of labelled **10** obtained with AcAS A) from (3-^{13}C)GGPP and IPP (only C7 is substituted by ^{13}C in the product), and B) from FPP and (3-^{13}C,2-^{2}H)DMAPP with addition of isopentenyl diphosphate isomerase (IDI). The upfield shifted triplet for C7 of **10** indicates a direct ^{13}C-^{2}H bond and thus supports the 1,2-hydride shift from **B** to **C**. Black dots indicate ^{13}C-labelled carbons.

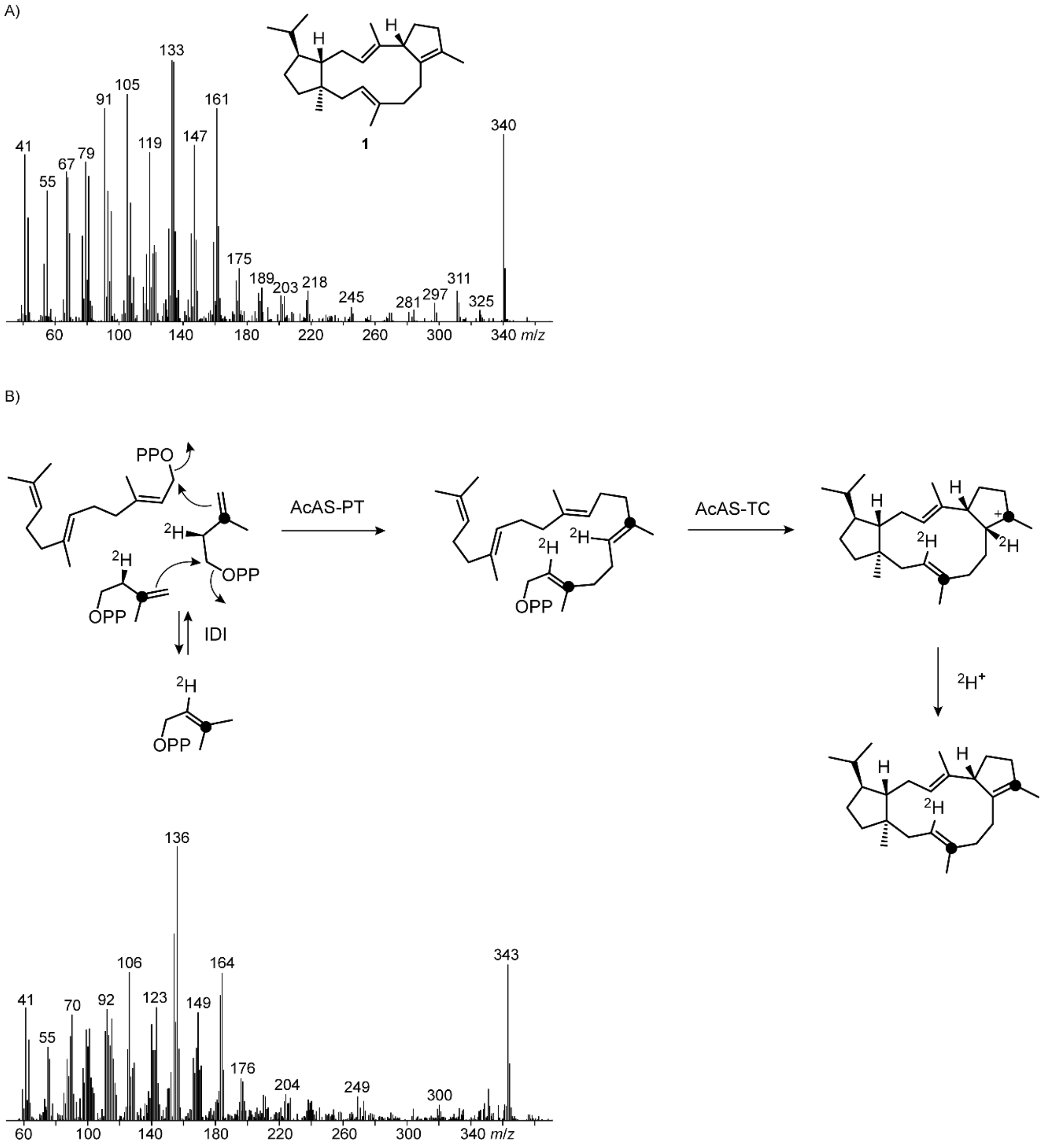

Figure S102. The biosynthesis of **1** proceeds with loss of deuterium from C6. A) EI mass spectrum of unlabelled **1**, B) labelling experiment with the substrates FPP and (3-^{13}C,2-^{2}H)DMAPP and enzymatic conversion with IDI and AcAS, and EI mass spectrum of ($^{13}C_2$,^{2}H)-**1**.

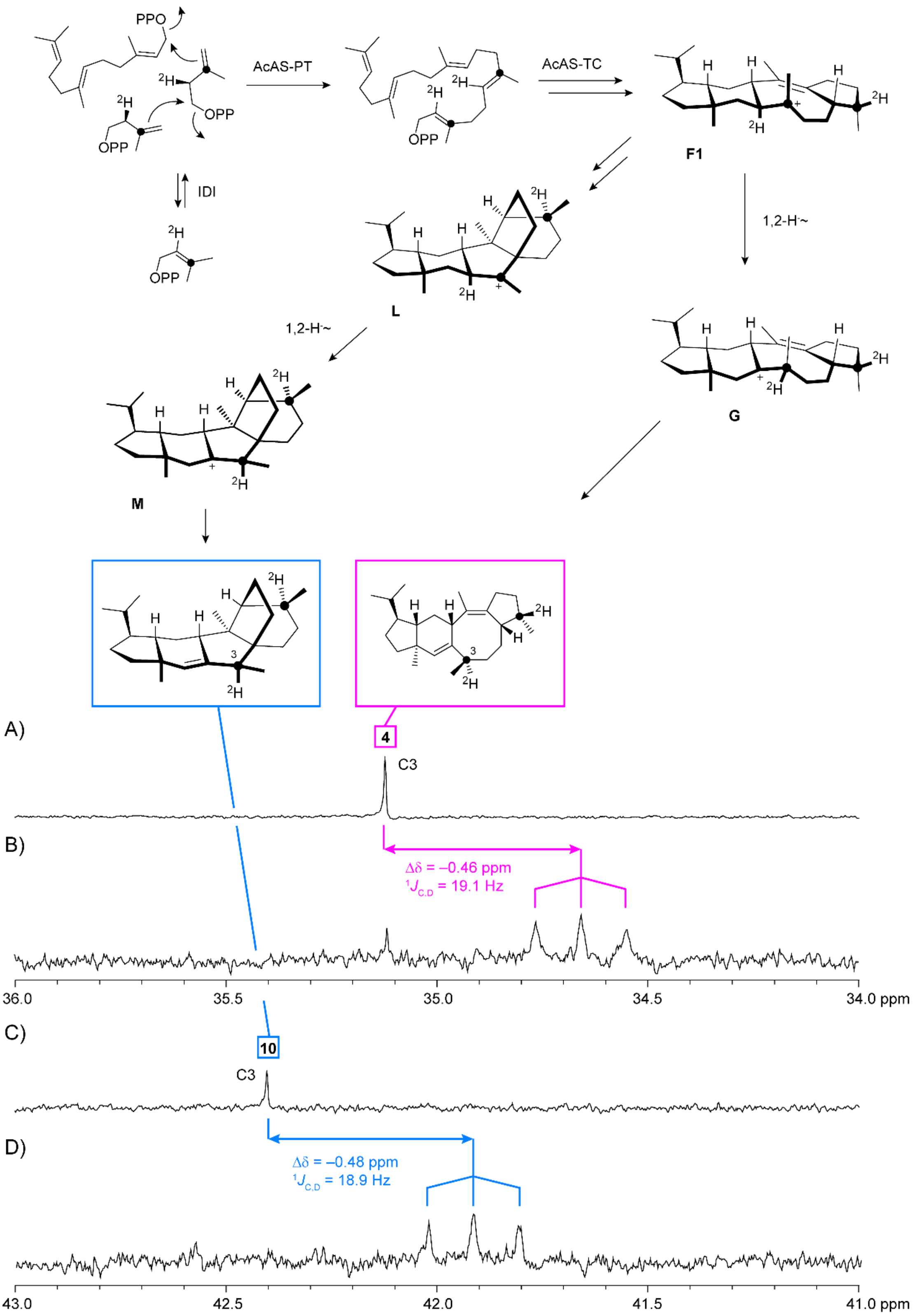

Figure S103. The 1,2-hydride shifts from **F1** to **G** and from **L** to **M**. Partial ^{13}C-NMR spectra of labelled **4** obtained with AcAS A) from GGPP and (3-^{13}C)IPP (only C3 is substituted by ^{13}C) and B) from FPP and (3-^{13}C,2-^{2}H)DMAPP with addition of IDI. Partial ^{13}C-NMR spectra of labelled **10** obtained with AcAS C) from GGPP and (3-^{13}C)IPP (only C3 is substituted by ^{13}C) and D) from FPP and (3-^{13}C,2-^{2}H)DMAPP with addition of IDI. The upfield shifted triplet signals indicate a direct ^{13}C-^{2}H bond and support the 1,2-hydride shifts from **F1** to **G** and from **L** to **M**. Black dots indicate ^{13}C-labelled carbons.

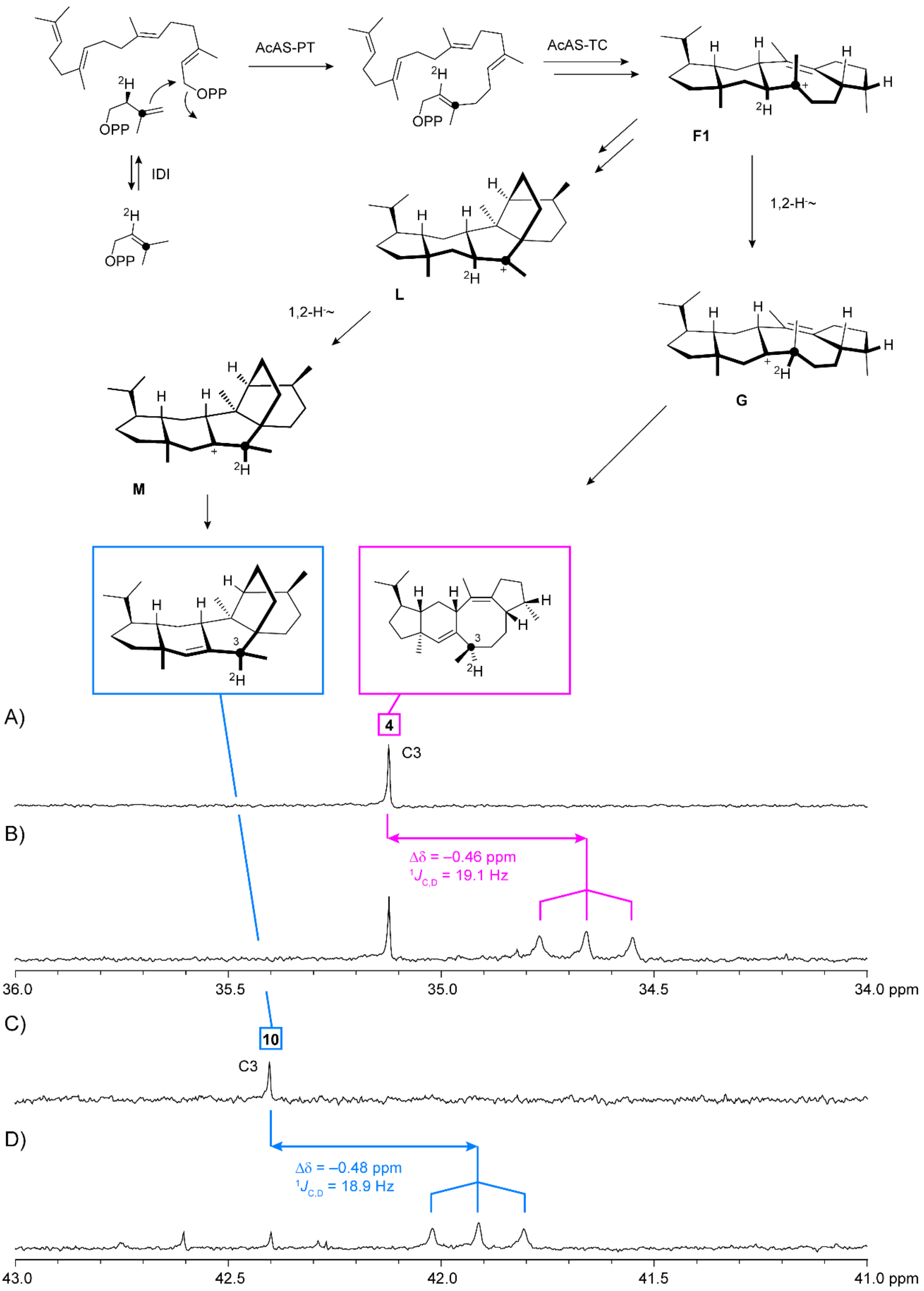

Figure S104. The 1,2-hydride shifts from **F1** to **G** and from **L** to **M**. Partial ^{13}C-NMR spectra of labelled **4** obtained with AcAS A) from GGPP and (3-^{13}C)IPP (only C3 is substituted by ^{13}C) and B) from GGPP and (3-^{13}C,2-^{2}H)DMAPP with addition of IDI. Partial ^{13}C-NMR spectra of labelled **10** obtained with AcAS C) from GGPP and (3-^{13}C)IPP (only C3 is substituted by ^{13}C) and D) from GGPP and (3-^{13}C,2-^{2}H)DMAPP with addition of IDI. The upfield shifted triplet signals indicate a direct ^{13}C-^{2}H bond and support the 1,2-hydride shifts from **F1** to **G** and from **L** to **M**. Black dots indicate ^{13}C-labelled carbons.

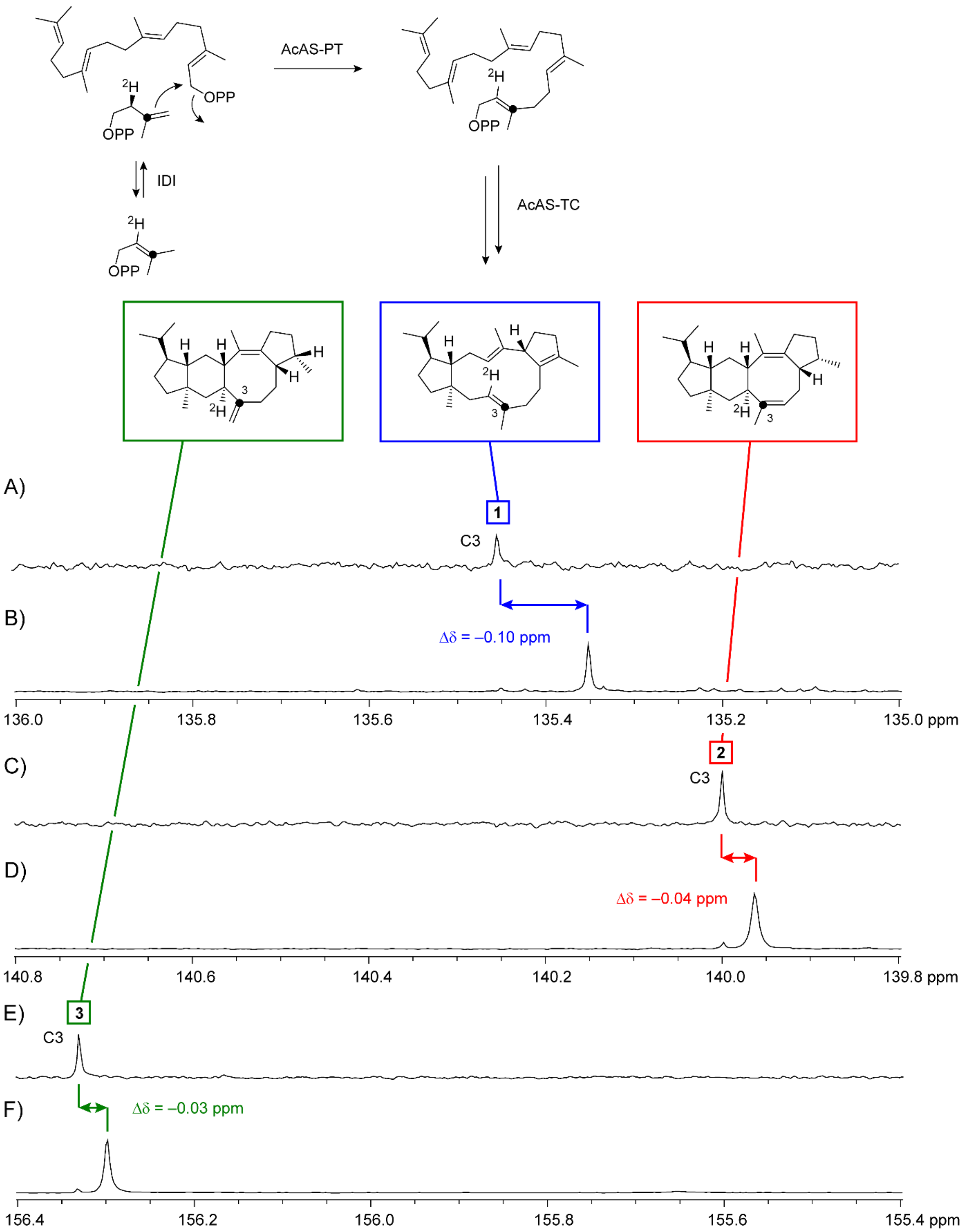

Figure S105. Enzymatic conversion of GGPP and (3-^{13}C,2-^{2}H)DMAPP with IDI and AcAS. Partial ^{13}C-NMR spectra of A) (3-^{13}C)-**1** obtained from GGPP and (3-^{13}C)IPP, B) (3-^{13}C,2-^{2}H)-**1** obtained with IDI and AcAS from GGPP and (3-^{13}C,2-^{2}H)DMAPP, C) (3-^{13}C)-**2** obtained from GGPP and (3-^{13}C)IPP, D) (3-^{13}C,2-^{2}H)-**2** obtained with IDI and AcAS from GGPP and (3-^{13}C,2-^{2}H)DMAPP, E) (3-^{13}C)-**3** obtained from GGPP and (3-^{13}C)IPP, and F) (3-^{13}C,2-^{2}H)-**3** obtained with IDI and AcAS from GGPP and (3-^{13}C,2-^{2}H)DMAPP. The small upfield shifts for the signals of C3 in B), D) and F) indicate a deuterium atom in a neighbouring position of C3 (C2). Black dots indicate ^{13}C-labelled carbons.

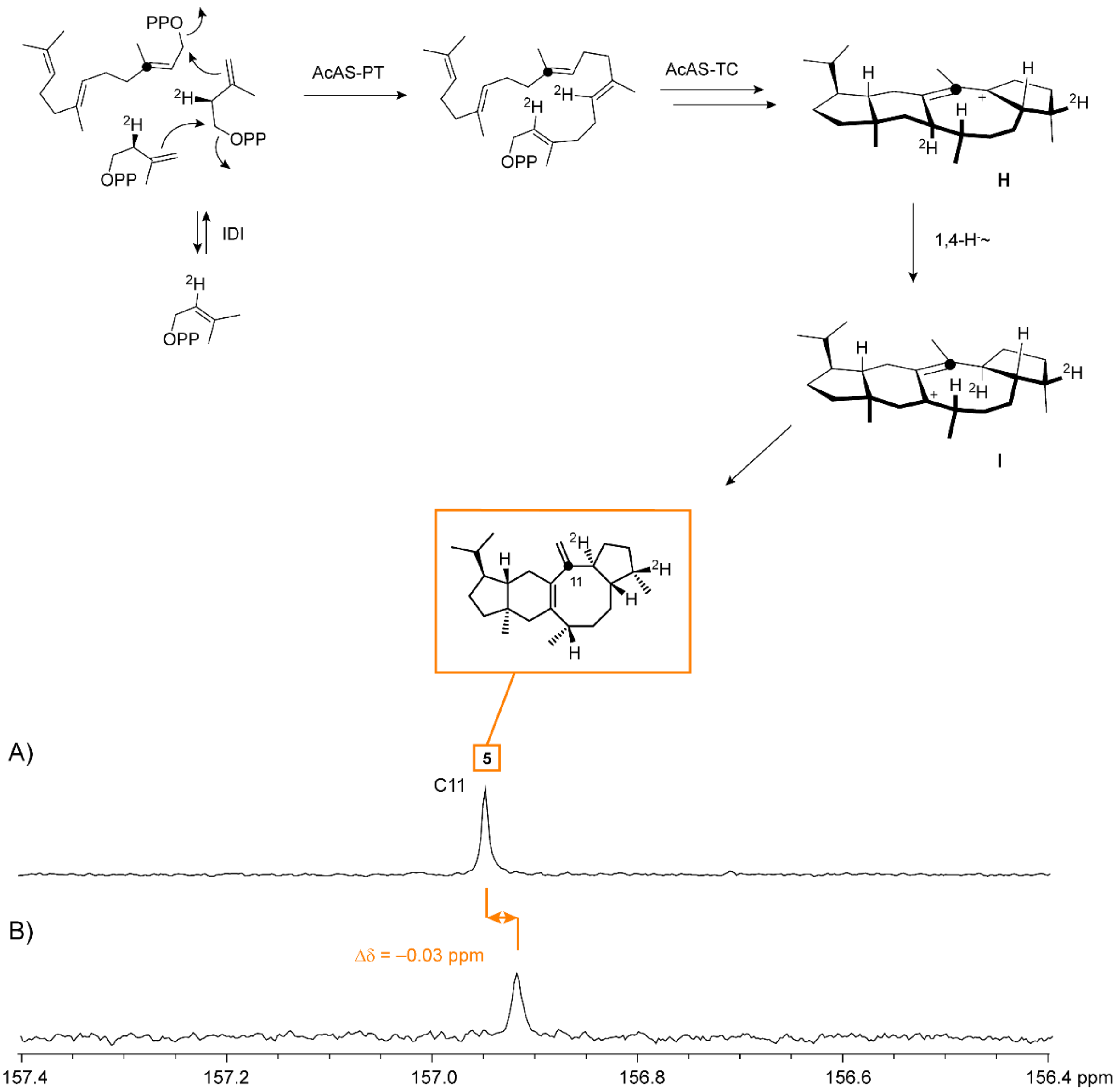

Figure S106. Enzymatic conversion of (3-^{13}C)FPP and (2-^{2}H)DMAPP with IDI and AcAS. Partial ^{13}C-NMR spectra of A) (11-^{13}C)-**5** obtained from (3-^{13}C)FPP and IPP, B) (11-^{13}C,7,10-$^{2}H_2$)-**5** obtained with IDI and AcAS from (3-^{13}C)FPP and (2-^{2}H)DMAPP. The small upfield shift for the signal of C11 in B) indicates a deuterium atom in a neighbouring position of C11 (C10). Black dots indicate ^{13}C-labelled carbons.

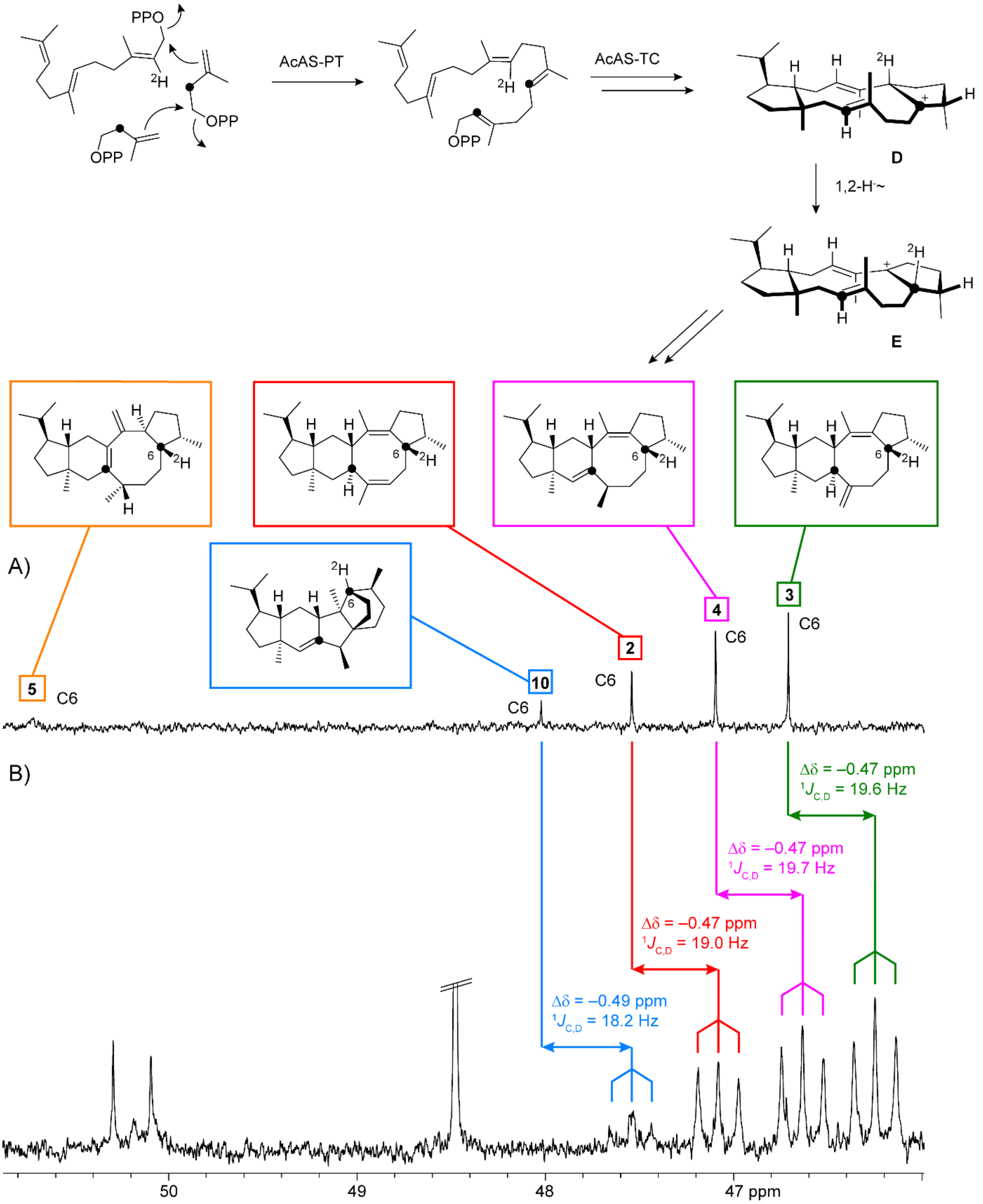

Figure S107. The 1,2-hydride shift from **D** to **E**. Partial ^{13}C-NMR spectra showing the region for C6 of A) the mixture of labelled enzyme products **2** – **5** and **10** obtained from (2-^{13}C)GGPP and IPP with AcAS, B) the mixture of labelled enzyme products **2** – **5** and **10** obtained from (2-^{2}H)FPP and (2-^{13}C)IPP with AcAS. The upfield shifted triplets for C6 of **2** – **4** and **10** indicate a deuterium atom directly bound to C6, giving evidence for the 1,2-hydride shift from **D** to **E**. For **10** and additional $^{3}J_{C,C}$ coupling is observed. The broad peak for C6 of **5** prevented detection of a corresponding triplet signal for this compound. Black dots indicate ^{13}C-labelled carbons.

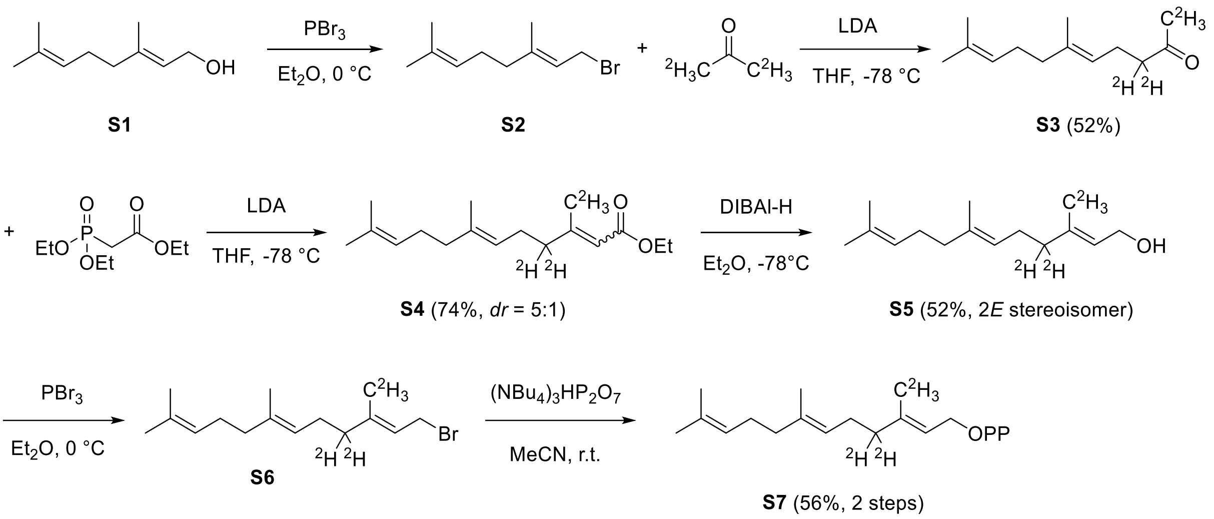

Scheme S1. Synthesis of (4,4,15,15,15-$^2H_5$)FPP.

Synthesis of (4,4,15,15,15-$^2H_5$)FPP

Synthesis of (1,1,1,3,3-$^2H_5$)-(*E*)-6,10-dimethylundeca-5,9-dien-2-one (S3)[30]

Geraniol **S1** (1.54 g, 10 mmol, 1.0 eq) was dissolved in dry diethyl ether (50 mL) and the solution was cooled to 0 °C. PBr_3 (4 mmol, 0.4 eq) was added dropwise to the solution. The reaction mixture was stirred 30 min at 0 °C. Then, the reaction mixture was poured onto ice and extracted two times with cold hexane. The hexane layers were combined and dried with $MgSO_4$ and concentrated under reduced pressure to obtain geranyl bromide (**S2**).
Dry THF (55 mL) was added into a reaction vessel and cooled to –10 °C. Diisopropylamine (557 mg, 5.5 mmol, 1.1 eq.) was dissolved in the cold THF and *n*-butyllithium solution (1.6 M in hexane, 3.4 mL, 2.85 mmol, 1.1 eq.) was added dropwise to the solution. The reaction mixture was stirred 1 h at –10 °C, then cooled to –78 °C. Acetone-d6 (353 mg, 2.85 mmol, 1.1 eq.) was added. The reaction mixture was stirred 2 h at –78 °C. Bromide **S2** (1086 mg, 5 mmol, 1 eq) was added at –78 °C and the reaction mixture was stirred overnight at room temperature. After 14 h, the reaction was quenched by adding H_2O (200 mL), then the aqueous layer was extracted four times with diethyl ether. The combined organic layers were washed with brine and dried with $MgSO_4$ and concentrated under reduced pressure. The residue was purified by silica gel chromatography using petroether/diethyl ether (stepwise from 100/0 to 80/20) to yield the product **S3** (517 mg, 2.6 mmol, 52%) as colorless oil.
TLC (cyclohexane/ethyl acetate [80/20]): R_f = 0.62. GC (HP5-MS): *I* = 1455. ^{1}H-NMR (500 MHz, C_6D_6): δ = 5.20 (tdt, $^3J_{H,H}$ = 7.0 Hz, $^4J_{H,H}$ = 2.8, 1.4 Hz, 1H, CH), 5.12 (tq, $^3J_{H,H}$ = 7.2 Hz, $^4J_{H,H}$ = 1.3 Hz, 1H, CH), 2.20 – 2.25 (m, 2H, CH_2), 2.10 – 2.17 (m, 2H, CH_2),2.00 – 2.05 (m, 2H, CH_2), 1.67 (br, 3H, CH_3), 1.55 (s, 6H, 2xCH_3) ppm. ^{13}C-NMR (125 MHz, C_6D_6): δ = 206.2 (C_q), 135.96 (C_q), 131.26 (C_q), 124.82 (CH), 123.59 (CH), 40.13 (CH_2), 27.11 (CH_2) 25.68 (CH_3), 22.63 (CH_2), 17.75 (CH_3), 16.01 (CH_3) ppm. MS (EI, 70 eV): *m*/*z* (%) = 199 (1), 180 (2), 156 (9), 155 (9), 136 (16), 130 (6), 121 (7), 112 (10), 93 (11), 81 (6), 69 (78), 46(100).

Synthesis of ethyl (4,4-$^2H_2$)-(2*E*,6*E*)-7,11-dimethyl-3-($^2H_3$-methyl)dodeca-2,6,10-trienoate (S4)[30]

Dry THF (5 mL) was added into a reaction vessel and cooled to –10 °C. Diisopropylamine (111 mg, 1.1 mmol, 1.1 eq.) was dissolved in the cold THF and *n*-butyllithium solution (1.6 M in hexane, 0.69 mL, 1.1 mmol, 1.1 eq.) was added dropwise to the solution. The reaction mixture was stirred 1 h at –10 °C, then cooled to –78 °C. Triethyl phosphonoacetate (246 mg, 1.1 mmol, 1.1 eq) was added. The reaction mixture was stirred 2 h at –78 °C. Compound **S3** (199

mg, 1 mmol, 1 eq) in dry THF (5 mL) was added at –78 °C, and stirred overnight at room temperature. After 14 h, the reaction was quenched by adding H_2O (20 mL), then the aqueous layer was extracted four times with diethyl ether. The combined organic layers were washed with brine and dried with $MgSO_4$, then concentrated under reduced pressure. The residue was purified by silica gel chromatography using petrolether/diethyl ether (stepwise from 100/0 to 80/20) to yield the product **S4** (200 mg, 0.74 mmol, 74%, 2*E* : 2*Z* = 5:1) as colorless oil.
TLC (cyclohexane/ethyl acetate [90/10]): R_f (2*E* + 2*Z*)= 0.74 (2*E*), 0.70 (2*Z*). GC (HP5-MS): *I* = 1858 (2*E*), 1815 (2*Z*). ^{1}H-NMR (500 MHz, C_6D_6) for (2*E*)-stereoisomer: δ = 5.82 (s, 1H, CH), 5.20 (tp, $^3J_{H,H}$ = 7.0 Hz, $^4J_{H,H}$ = 1.4 Hz, 1H, CH), 5.08 (tq, $^3J_{H,H}$ = 7.2 Hz, $^4J_{H,H}$ = 1.4 Hz, 1H, CH), 4.05 (q, $^3J_{H,H}$ = 7.2 Hz, 2H, CH_2), 1.98 – 2.15 (m, 6H, 3xCH_2), 1.69 (br, 3H, CH_3), 1.56 (s, 3H, CH_3), 1.50 (s, 3H, CH_3) ppm. ^{1}H-NMR (500 MHz, C_6D_6) for (2*Z*)-stereoisomer: δ = 5.75 (s, 1H, CH), 5.33 (ddq, $^3J_{H,H}$ = 8.5, 7.2 Hz, $^4J_{H,H}$ = 1.4 Hz, 1H, CH), 5.20 (m, 1H, CH), 4.03 (q, $^3J_{H,H}$ = 7.2 Hz, 2H, CH_2), 2.05 – 2.25 (m, 6H, 3xCH_2), 1.68 (br, 3H, CH_3), 1.65 (s, 3H, CH_3), 1.56 (s, 3H, CH_3) ppm. ^{13}C-NMR (125 MHz, C_6D_6) for (2*E*)-stereoisomer: δ = 166.48 (C_q), 159.19 (C_q), 135.98 (C_q), 131.26 (C_q), 124.83 (CH), 123.57 (CH), 116.51 (CH), 59.37 (CH_2), 40.11 (CH_2), 27.14 (CH_2), 26.10 (CH_2), 25.86 (CH_3), 17.75 (CH_3), 16.03 (CH_3), 14.45 (CH_3) ppm. ^{13}C-NMR (125 MHz, C_6D_6) for (2*Z*)-stereoisomer: δ = 166.05 (C_q), 159.75 (C_q), 135.84 (C_q), 131.14 (C_q), 124.99 (CH), 124.22 (CH), 116.97 (CH), 59.36 (CH_2), 40.19 (CH_2), 27.18 (CH_2), 27.04 (CH_2), 25.84 (CH_3), 16.08 (CH_3), 14.41 (CH_3) ppm. MS (EI, 70 eV, (2*E*)-stereoisomer): *m*/*z* (%) = 269 (1), 224 (3), 195 (2), 180 (2), 152 (3), 133 (22), 126, (12), 109 (5), 105 (8), 95 (8), 87 (13), 81 (24), 69 (100), 53 (7), 41 (43). MS (EI, 70 eV, (2*Z*)-stereoisomer): *m*/*z* (%) = 269 (3), 226 (4), 179 (3), 152 (13), 132 (13), 125 (20), 109 (24), 104 (7), 95 (13), 86 (13), 81 (32), 69 (100), 55 (12), 41 (61).

Synthesis of (2*E*,6*E*)-(4,4,15,15,15-$^2H_5$)farnesol (S5)[30]

Compound **S4** (200 mg, 0.74 mmol, 1.0 eq.) was dissolved in dry diethyl ether (10 mL). The solution was cooled to –78 °C. DIBAl-H (1.0 M in hexane, 1.5 mL, 2.0 eq.) was added dropwise to the solution at –78 °C. The reaction mixture was stirred 2 h at room temperature. The reaction mixture was cooled to 0 °C on ice, and quenched by slowly adding saturated sodium potassium tartrate solution while stirring. The mixture was extracted four times with diethyl ether. The organic layers were combined and washed with brine, then dried with $MgSO_4$. The extract was concentrated under reduced pressure. The residue was purified by column chromatography on silica gel eluting stepwise using cyclohexane/ethyl acetate from 100/0 to 80/20 to give product **S5** (88 mg, 0.39 mmol, 52%) as colorless oil. The (2*Z*)-stereoisomer was removed at this stage.
TLC (cyclohexane/ethyl acetate [90/10]): R_f = 0.42. GC (HP5-MS): *I* = 1533. ^{1}H-NMR (500 MHz, C_6D_6) for (2*E*)-stereoisomer: δ = 5.39 (t, $^3J_{H,H}$ = 6.7 Hz, 1H, CH), 5.20 – 5.25 (m, 2H, 2xCH), 3.97 (d, $^3J_{H,H}$ = 6.6 Hz, 2H, CH_2), 2.14 – 2.20 (m, 2H, CH_2), 2.05 – 2.13 (m, 4H, 2xCH_2), 1.68 (br, 3H, CH_3), 1.58 (s, 3H, CH_3), 1.57 (s, 3H, CH_3) ppm. ^{13}C-NMR (125 MHz, C_6D_6) for (2*E*)-stereoisomer: δ = 138.05 (C_q), 135.30 (C_q), 131.23 (C_q), 125.01 (CH), 124.91 (CH), 124.49 (CH), 59.38 (CH_2), 40.21 (CH_2), 27.22 (CH_2), 26.62 (CH_2), 25.88 (CH_3), 17.77 (CH_3), 16.11 (CH_3) ppm. MS (EI, 70 eV, (2*E*)-stereoisomer): *m*/*z* (%) = 220 (5), 205 (24), 136, (17), 123 (34), 109 (34), 94 (49), 81 (26), 69 (100), 55 (41), 41 (86).

Synthesis of trisammonium (2*E*,6*E*)-(4,4,15,15,15-$^2H_5$)FPP (S7)

The substrate **S5** (30 mg, 0.13 mmol, 1.0 eq.) was dissolved in dry diethyl ether (5 mL) and the solution was cooled on ice to 0 °C. PBr_3 (13 mg, 0.05 mmol, 0.4 eq.) was added dropwise to the solution on ice. The reaction mixture was stirred 30 min at 0 °C. Then, the reaction mixture was poured onto ice. The reaction mixture was extracted two times with cold diethyl ether. The organic layers were combined and dried with $MgSO_4$, then concentrated under reduced pressure to obtain (2*E*,6*E*)-(4,4,15,15,15-$^2H_5$)farnesyl bromide (**S6**). $(NBu_4)_3HP_2O_7$ (141 mg, 0.16 mmol, 1.2 eq) was dissolved in dry acetonitrile (5 mL). Crude **S6** was added to

the solution. The reaction solution was stirred overnight at room temperature. After 14 h, the reaction was stopped by concentration under reduced pressure. The residue was loaded onto an ion exchange resin column (DOWEX 50WX8, 100-200 mesh, NH_4^+ form). The product was eluted with 1.5 column volumes of freshly prepared ion exchange buffer (0.025 M NH_4HCO_3 in 2% *i*PrOH/H_2O) and freeze-dried to obtain **S7** (28 mg, 0.07 mmol, 56%, 2 steps) as a colourless solid.
HRMS (ESI negative) *m*/*z*: $[M+2H]^-$ calcd. for $C_{15}H_{20}{}^2H_5O_7P_2H_2^-$ 386.1551; found 386.1548. ^{1}H-NMR (500 MHz, D_2O): δ = 5.49 (t, $^3J_{H,H}$ = 7.1 Hz, 1H, CH), 5.18 – 5.26 (m, 2H, 2xCH), 4.48 (t, $^3J_{H,H}$ = 6.6 Hz, 2H, CH_2), 2.09 – 2.18 (m, 4H, 2xCH_2), 2.01 – 2.06 (m, 2H, CH_2), 1.70 (s, 3H, CH_3), 1.64 (s, 6H, 2xCH_3)ppm. ^{13}C-NMR (125 MHz, D_2O): δ = 142.75 (C_q), 136.77 (C_q), 133.62 (C_q), 124.54 (CH), 124.37 (CH), 120.03 (CH), 62.53 (CH_2), 38.86 (CH_2), 25.85 (CH_2), 25.58 (CH_2), 24.93 (CH_3), 17.04 (CH_3), 15.32 (CH_3) ppm. ^{31}P-NMR (202 MHz, D_2O): δ = –5.83 (d, $^2J_{P,P}$ = 21.5 Hz), –9.95 (d, $^2J_{P,P}$ = 21.9 Hz) ppm.

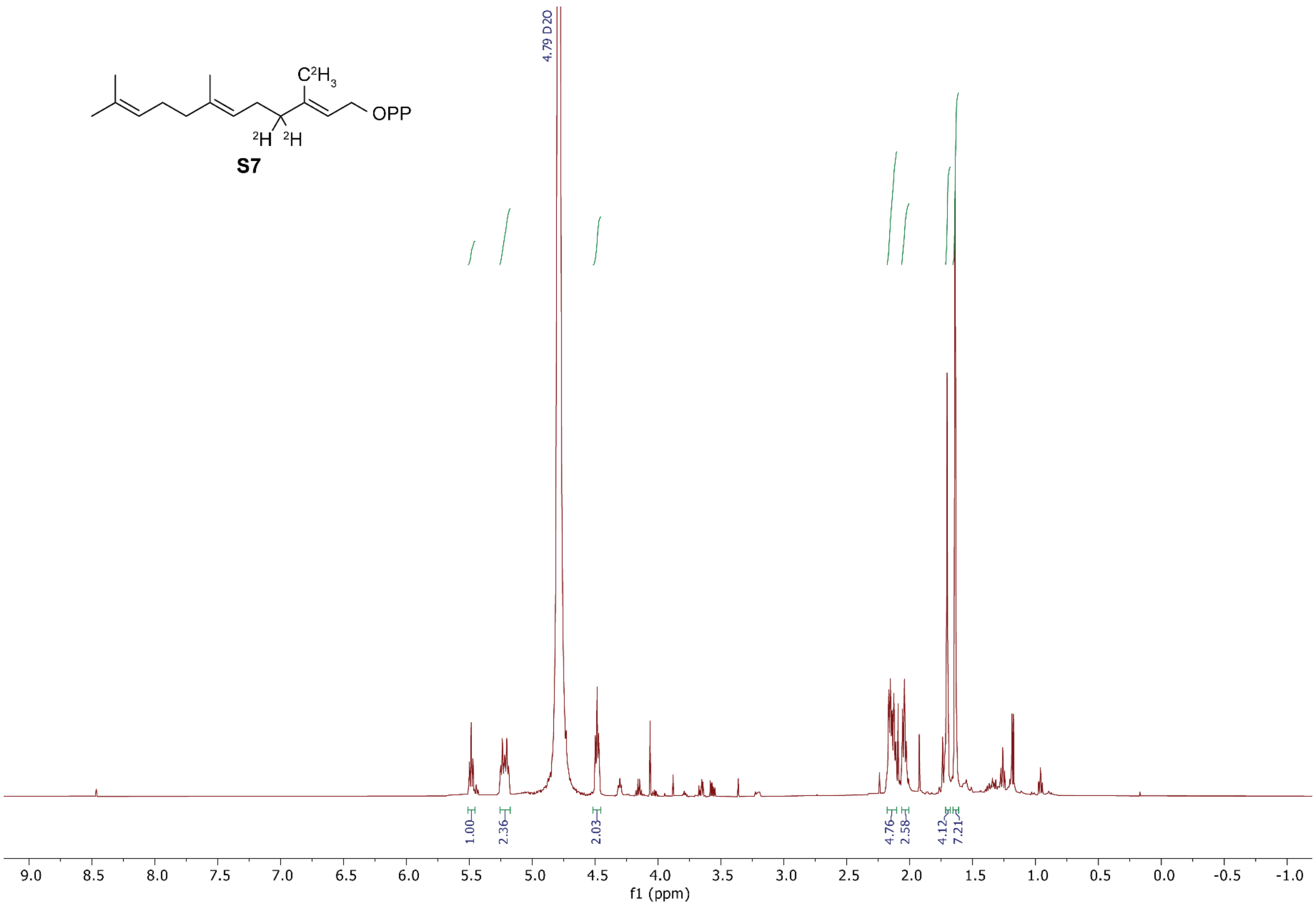

Figure S108. ^{1}H-NMR spectrum of **S7** (500 MHz, D_2O).

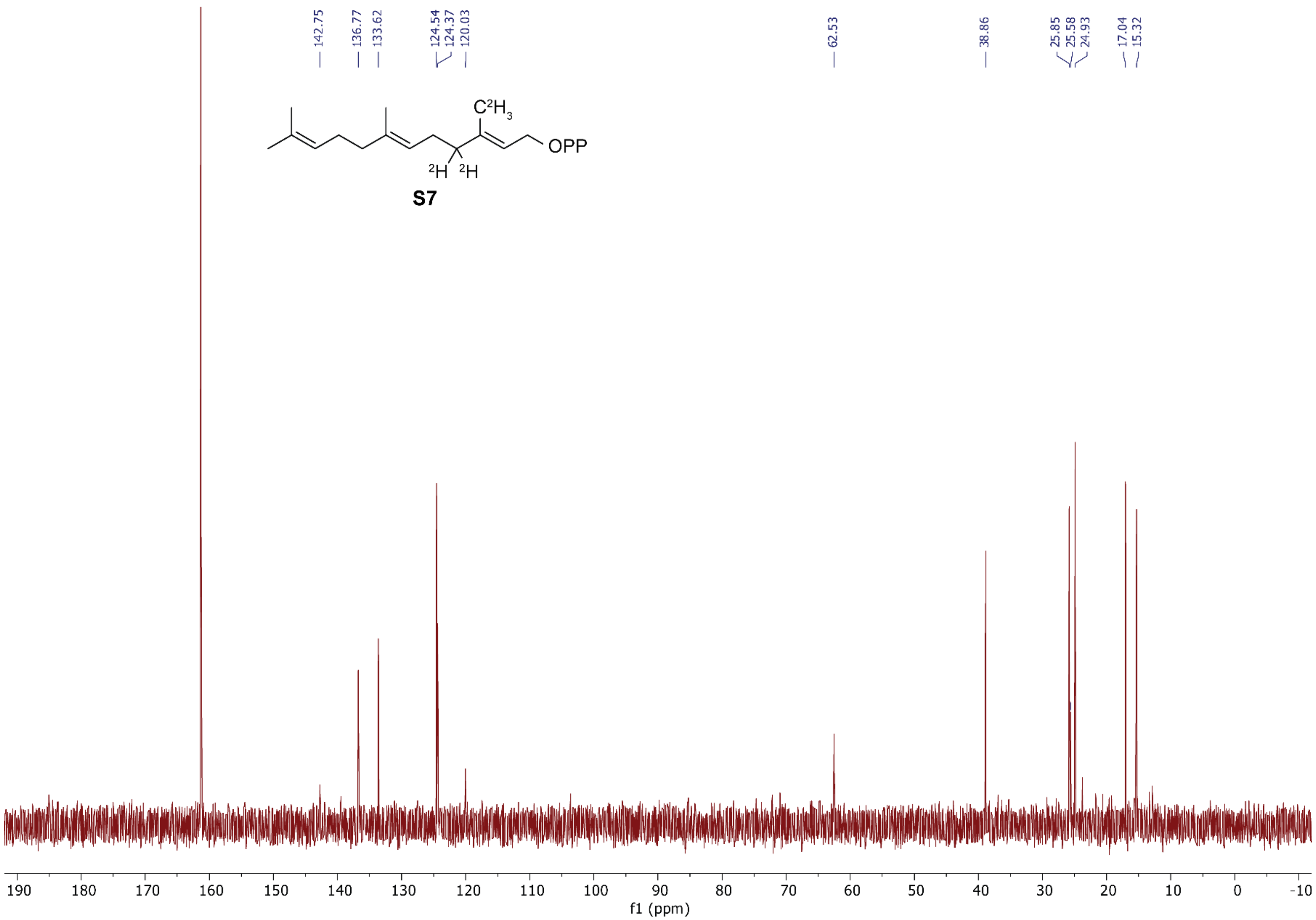

Figure S109. ^{13}C-NMR spectrum of **S7** (125 MHz, D_2O).

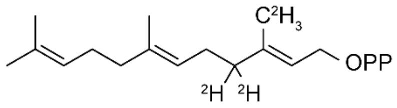

S7

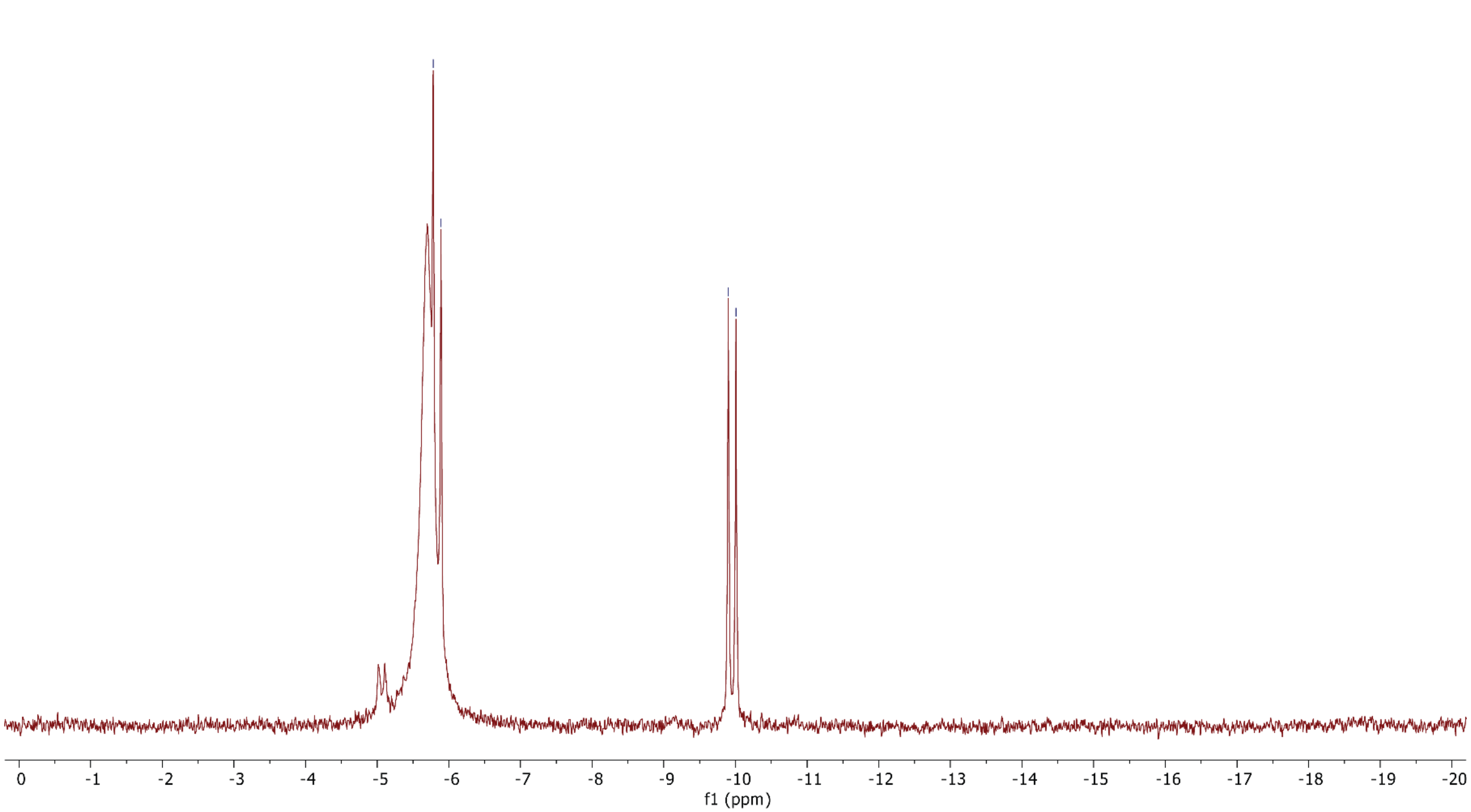

Figure S110. ^{31}P-NMR spectrum of **S7** (202 MHz, D_2O).

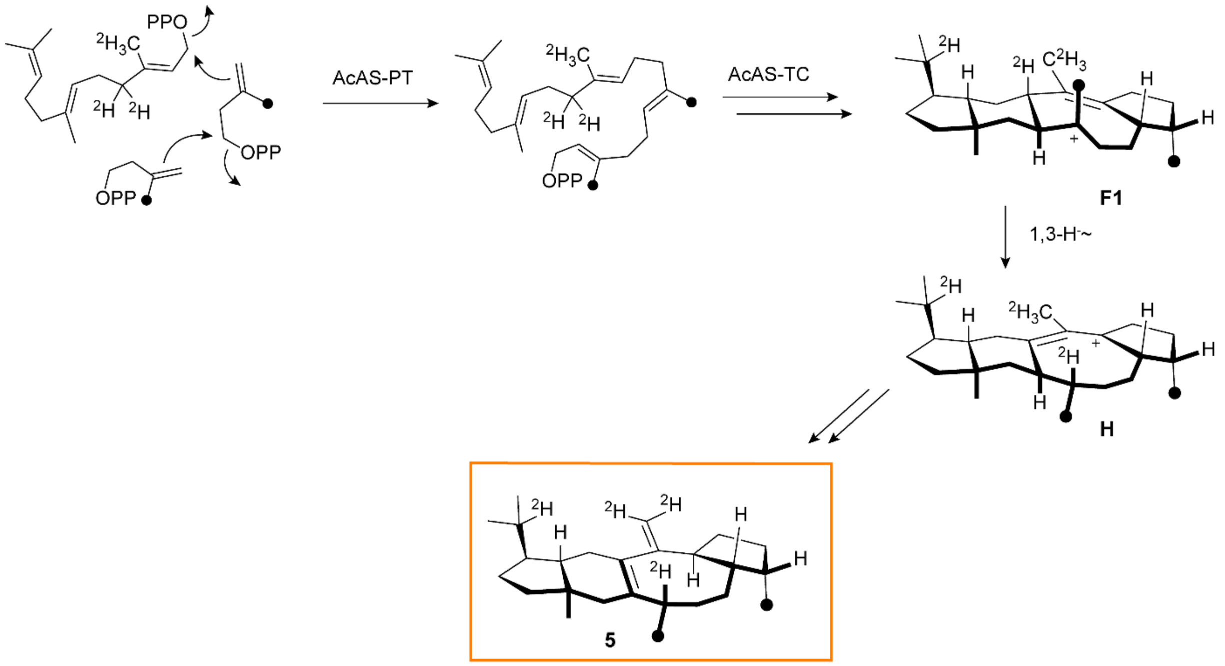

A)

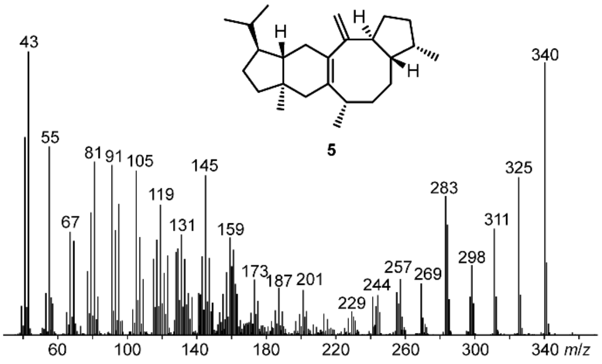

B)

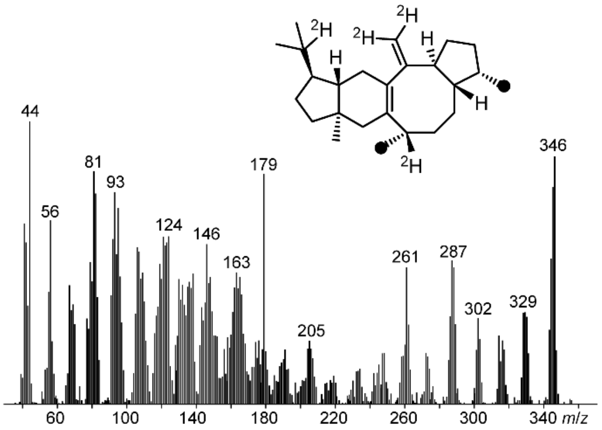

Figure S111. The 1,2-hydride shift from **F1** to **H**. EI mass spectra of A) unlabelled **5**, and B) labelled ($^{13}C_2$,$^{2}H_4$)-**5** obtained from (4,4,15,15,15-$^{2}H_5$)FPP and (5-^{13}C)IPP with AcAS. The molecular ion at *m*/*z* 346 indicates retainment of four of the five deuterium atoms in the substrate. Mass peaks at *m*/*z* 345, 344 and 343 are a result of incomplete deuteration in the substrate. Black dots indicate ^{13}C-labelled carbons.

Computational methods

All computed structures are geometry optimized without restrictions and are characterized as minima or as transition state structures by frequency analyses, also providing Gibbs-corrections, using the B97D3/6-31g(d,p) method with the density fitting approximation for s- and p-functions, including Grimme's empirical D3-dispersion correction[31] in Gaussian16.[32]

For improved single point energies, the mPW1PW91 functional is applied with the 6-311+G(d,p) basis set without density fitting and the ultra-fine integration grid, as this method has shown to be very reliable for examining carbocation cyclisationrearrangement reactions.[33]

The Gibbs-corrections include an entropic quasi-harmonic treatment with a frequency cut-off value of 100.0 wavenumbers, according to Grimme, using a mixture of RRHO and free-rotor vibrational entropies.[34]

Table S9. Results of DFT calculations.

**Structure[a]**	**Single Point Energy with Gibbs Corrections**<br>**Hartree**	**relative to A**<br>**kcal/mol**
**A**	977.059646	0.00
**A-TS**	977.057752	+1.19
**B**	977.072391	–8.00
**B-TS**	977.059274	+0.23
**C**	977.072005	–7.76
**C-TS**	977.071965	–7.73
**D**	977.081858	–13.94
**D-TS**	977.069359	–6.10
**E**	977.116224	–35.50
**E-TS**	977.115153	–34.83
**F1**	977.115940	–35.33
**F1/G-TS**	977.097765	–23.92
**G**	977.110734	–35.92
**F1/H-TS**	977.105860	–29.00
**H**	977.118422	–36.88
**H-TS**	977.092832	–20.82
**I**	977.126947	–42.23
**F1/F2-TS**	977.103976	–27.82
**F2**	977.123609	–40.14
**J**	977.147330	–55.02
**J-TS**	977.138483	–49.47
**K**	977.133042	–46.06
**L**	977.147215	–54.95
**L-TS**	977.139460	–50.09
**M**	977.147531	–55.15

[a] For structural drawings and transition states cf. Scheme 1 of main text and Figure S112.

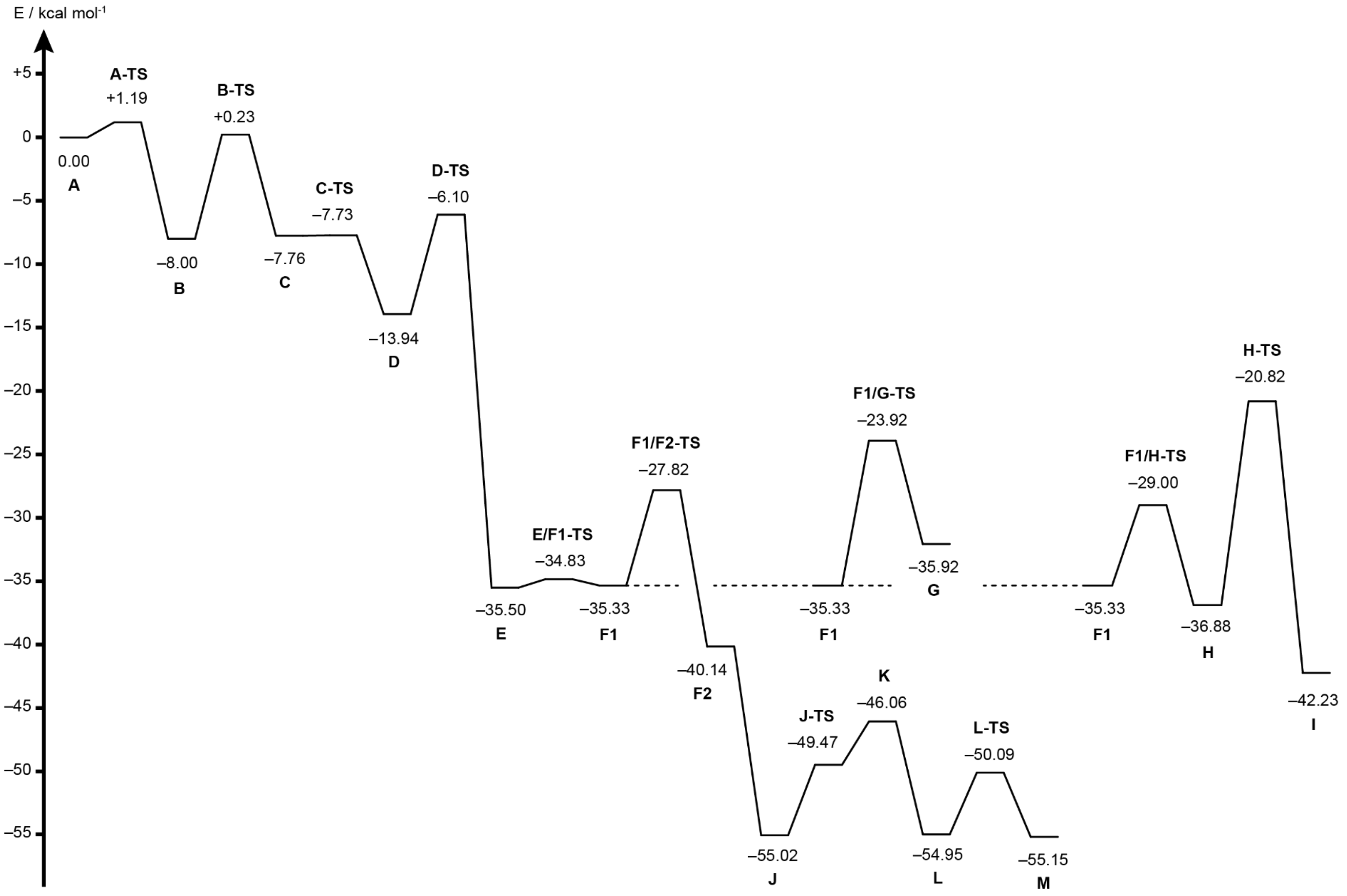

Figure S112. Energy profile for the cyclisation mechanism by AcAS as shown in Scheme 1 of main text based on data from DFT calculations (Table S9). The dashed lines connect identical structures.

```
AcAS        MDAALRDICQLSDPCDDPRSFEPPIKDFFCIYPMYRSRYEAKAIQGSNEFLDGWNKAIEKD    60
PbSS        -MDFLSGAFHYSDSVNPSKYSPRPSDYFGTLPFRTSRFEREAADVTADYLRKWQKAVKAD    59
                *      **   *    *   * *   *   ** *  *       *  * **   *

AcAS        GLRNDGRPFLGCNTIYGNYVAWAYPECLPERAAHVAAYCDWGFFWDDATDAMSMEKNHEA    120
PbSS        NPERKDLVFHGSTTTLGHFVSWAYPECIPDRVDLCTQICDFGFYWDDVTDSVNVQENAEI    119
                    * *  *  *  * ****** * *       ** ** *** **      * *

AcAS        TKDLILTIMSTVGIGQKHEPLLAVNKLVVPFVLNKLAGTDGDLGLNHMKAWKAHLDGQAR    180
PbSS        TQDLALALLSELTLGQRLEPKLEINKIVVQMLWGVL-DKDRKSGLEMIKFWKGHLDGQAE    178
            * ** *   *    **  ** *  ** **      *   *   **   * ** ******

AcAS        SSHANMSWEELKQHRLVEGGPEWAIRLGAWGAGIRCTAEEIESVREIIDIGGIAGVLAND    240
PbSS        SAHNNMSFEEYTKHRLSEVGARWAVEVGCWSLGINLSREKKDSVAHFVNKGLLAAALMND    238
            * * *** **   *** * *  **   * *  **    *   **      *  *  * **

AcAS        YYSFNKEFDEHSRAGTIERMQNGVALLMREYGYSEEEAREILKKEINKMEQQFMDMYLTW    300
PbSS        YYSFNKEFDEHQRAGSMDRLQNGLGILMREYGYTETEARSILREEIRKGERAIMDGYIAW    298
            *********** ***   * ***   ******* * *** **  ** * *   ** *  *

AcAS        LNGPV--QKSRGLIQYLTMVLCLYSGTMFWMAHGARYHRTDLITTAEDRATIIGKCQGDA    358
PbSS        RESADSSSESHELNRYIVMIILMIGGITFWSSHASRYHRDDLITTAGDRAMIVGKFQC-S    357
                     *  *  *  *      *  **  *  **** ****** *** * ** *

AcAS        FRVMEGYPPPKGLKRTASSPESAPKRRASK----------------------ANNINQS    395
PbSS        MRLLDGYPPPNRWKSATSSNDISGRKRKSWSDSNGVDTHGACYTNGSSNRAKRNGTEAGH    417
             *   *****   *   **       * *

AcAS        NGRGGDPMVAFSGPFVKAPSHICDAPYEYIDSLQSKNMRDKFINILNSWLNVPSDSLQII    455
PbSS        KANGHDSMDIYTAPFLKAPSEVCEAPYEYINSLQGKNMRNKFMDALNHWLCVPAPSMQII    477
               * * *     ** ****  * ****** *** **** **   ** ** **  * ***

AcAS        KNIVQMLHNSSLMLDDIEDASPLRRGQPATHIFYGASQTINSANFSYVKTVIEATHLKNP    515
PbSS        KNIVQMLHNSSLMLDDIEDESPLRRGQPVAHTFYGISQTINSANFVYVKSVKETSRLKNP    537
            ******************* ********  * *** ********* *** * *   ****

AcAS        QCLQIFLEEVSDLHRGQSLDLHWRHHGRCPTTDEYIMMVDNKTGGLFRLMARLMEAESPS    575
PbSS        ICMEIFTDELSNLHTGQSLDLYWRYHGRCPSINEYIMMVDNKTGGLFRLMLRLMEAESPA    597
             *  **  * * ** ****** ** *****   ***************** ********

AcAS        PITIPHLSRLLTLIGRYYQIRDDYMNLTSADYTTKKGYCEDLDEGKFSLPLIHLLLHTSC    635
PbSS        ASS-ASLVKLLTLTGRYYQIRDDYLNLTSVEYTSKKGFCEDLDEGKFSLPLLHLLNHTRH    656
                  *  **** ********** ****  ** *** ************* *** **

AcAS        PDRITSALYNRVPS-TGLQDEVKTYILDAMQSARTFEYVREVLSHLHGEIMKTLDEAEKT    694
PbSS        PDRITAPLFNRASGARSLAREVKVHIIQAMDEAGTFEYAQGVLKYLHEEIMRTLDEVEAD    716
            *****  * **      *  ***  *  **  * ****   **  ** *** **** *

AcAS        LGINNGVRMLLVGLGL 710
PbSS        LGRNTEARILLLGLGL 732
            ** *   * ** ****
```

Figure S113. Amino acid sequence alignment of AcAS and PbSS. Positions with identical amino acid residues are indicated by asterisks, highly conserved residues and motifs that are important for enzyme function are marked in yellow, amino acid residues of AcAS targeted by site-directed mutagenesis are highlighted in bold red.

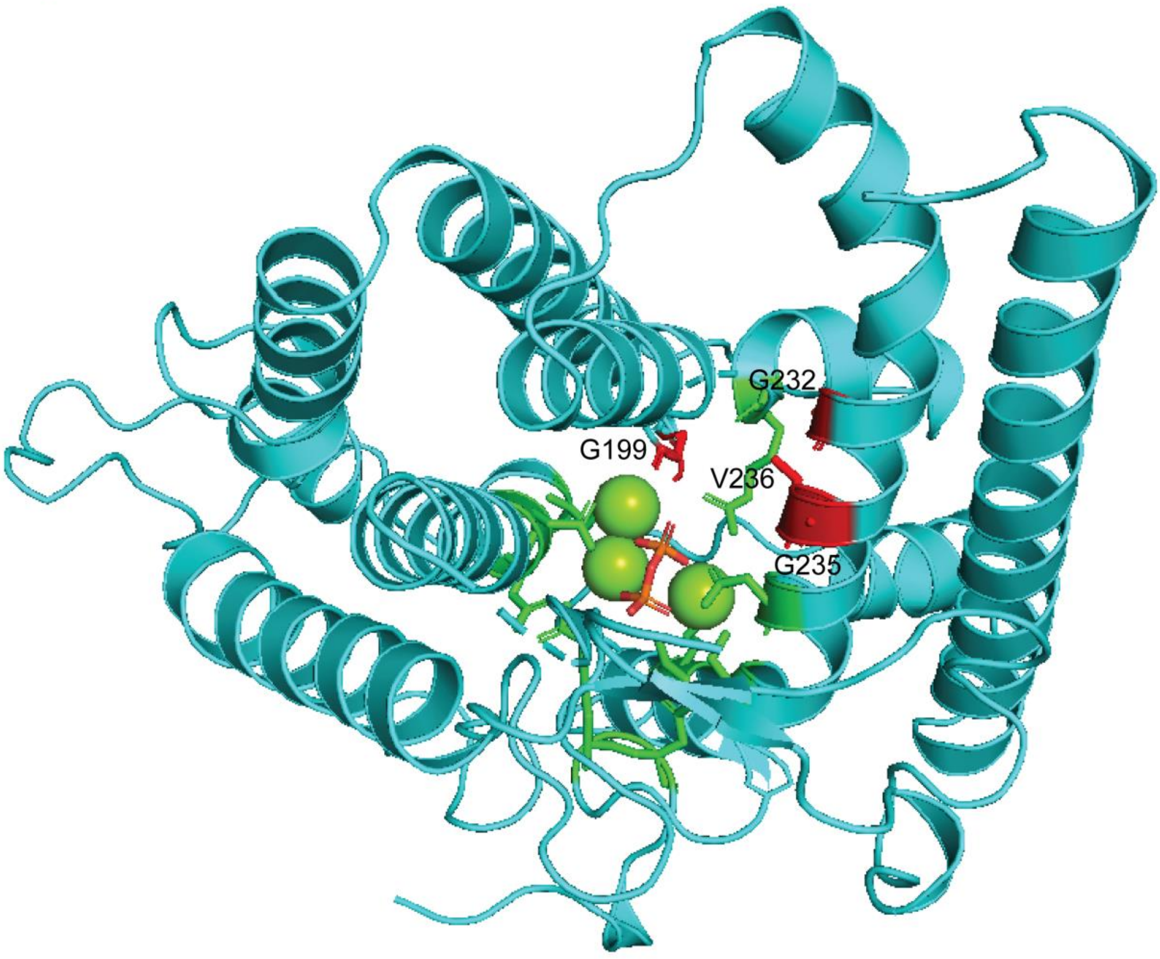

Figure S114. Homology model of the AcAS-TC domain based on the TC domain of *Fusasrium graminearum* GJ1012 synthase (FgGS).[35] The residues G199, G232, G235, and V236 marked in red reside in the active site pocket.

Site-directed mutagenesis of AcAS

The primers used for site-directed mutagenesis are listed in Table S1. The single and double mutations were performed by the overlap extension PCR method.[36] Plasmid pYE-AcAS-cDNA was used as the template for mutagenesis. Q5 DNA polymerase was used in PCR. Fragment 1s of mutation gene were amplified by using primers pairs of AcAS-pYE-Fw + AcAS-V197S-Rv, AcAS-pYE-Fw + AcAS-G199V-Rv, AcAS-pYE-Fw + AcAS-G199A-Rv, AcAS-pYE-Fw + AcAS-E202R-Rv, AcAS-pYE-Fw + AcAS-G232L-Rv, AcAS-pYE-Fw + AcAS-G232A-Rv, AcAS-G235A-Fw + AcAS-pYE-Rv, AcAS-V236A-Rv, and AcAS-pYE-Fw + AcAS-A238M-Rv. Fragment 2s of mutation gene were amplified by using primers pairs of AcAS-V197S-Fw + AcAS-pYE-Rv, AcAS-G199V-Fw + AcAS-pYE-Rv, AcAS-G199A-Fw + AcAS-pYE-Rv, AcAS-E202R-Fw + AcAS-pYE-Rv, AcAS-G232L-Fw + AcAS-pYE-Rv, AcAS-G232A-Fw + AcAS-pYE-Rv, AcAS-G235A-Fw + AcAS-pYE-Rv, AcAS-V236A-Fw + AcAS-pYE-Rv, and AcAS-A238M-Fw + AcAS-pYE-Rv. Two corresponding fragments were mixed and used as the templates. PCRs were performed through following process to yield AcAS-V197S, AcAS-G199V, AcAS-G199A, AcAS-E202R, AcAS-G232L, AcAS-G232A, AcAS-G235A, AcAS-V236A, and AcAS-A238M as mutated fragments. Step 1 were performed without primers: 1) 98 °C for 30 s; 2) 98 °C for 10 s, 60 °C for 30 s, 72 °C for 65 s; repeated 5 times; 3) 72 °C for 2 min. Step 2: AcAS-pYE-Fw + AcAS-pYE-Rv were used as the primer pair, 1) 98 °C for 30 s; 2) 98 °C for 10 s, 65 °C for 30 s, 72 °C for 65 s; repeated 35 times; 3) 72 °C for 2 min.
The mutated fragments were integrated into linearized pYE-Express by homologous recombination in yeast using the standard PEG/LiOAc/salmon sperm protocol.[22] After culturing transformed *S. cerevisiae* FY834 on selective agar medium for three days, the plasmid integrated with DNA fragment was isolated from the yeast by using the NucleoSpin Plasmid (NoLid) kit, followed by transformation of *E. coli* BL21 (DE3). *E. coli* BL21 (DE3) was grown on selective LB medium with kanamycin (50 μg mL^{-1}) at 37 °C overnight. A single colony was selected to grow a liquid culture, from which plasmid DNA was isolated and verified by sequencing.

Relative activity of enzyme variants

The activities of mutated AcASs were performed by using freshly prepared enzymes (concentrated to 2.0 mg mL^{-1}, 100 μL) and FPP (0.5 mg) plus IPP (0.5 mg) dissolved in 25 mM NH_4HCO_3 buffer (100 μL). Incubation buffer (870 μL) were added. The reaction mixtures were incubated at 30 °C overnight and then extracted with benzene for GC/MS analysis. Each enzymatic reaction was repeated for three times to take average value. The wildtype AcAS was used as the negative control and also repeated for three times. Relative activities of enzymes were calculated by comparing average peak area in GC/MS profiles with that of the wildtype AcAS.

The measuring of enzymatic activity in addition of (2-hydroxypropyl)-β-cyclodextrin (β-CD) was conducted by using freshly prepared AcAS-wildtype, AcAS-E202R, and AcAS-A238M enzymes (concentrated to 2.0 mg mL^{-1}). The buffer of β-CD (160 mM β-CD, 0.2 % SDS, 10 % glycerol, 10 mM $MgCl_2$, pH = 8.2) was prepared for further using. The combination of FPP (0.5 mg) and IPP (0.5 mg) or GFPP (0.5 mg) was dissolved in 25 mM NH_4HCO_3 solution (100 μL) and used as substrate. The reaction mixtures in negative control group were including substrate solution (FPP + IPP or GFPP, 100 μL), concentrated enzyme (2.0 mg $mL^{-}1$, 100 μL), and incubation buffer (800 μL). The reaction mixtures in β-CD adding group were composed of substrate solution (100 μL), concentrated enzyme (2.0 mg $mL^{-}1$, 100 μL), β-CD buffer (62.5 μL), and incubation buffer (737.5 μL). The reaction mixtures were incubated at 30 °C overnight and then extracted with benzene for GC/MS analysis. Each enzymatic reaction was repeated for three times to take average value. Relative activities of enzymes were calculated by comparing average peak area in GC/MS profiles with that of the wildtype AcAS without β-CD.

Table S10. Relative activity of AcAS enzyme variants.

Mutation	Total[a]	**1**	**2** + **3**[b]	**4**	**5**	**10**
wildtype	100±10	7.2±0.5	50±8	27±2	9.4±0.7	6.7±0.5
V197S	123±9	4.9±0.3	53±7	23±0.6	29±1.1	12.3±0.4
G199V	0.0±0.0	0.0±0.0	0.0±0.0	0.0±0.0	0.0±0.0	0.0±0.0
G199A	23±4	3.4±1.5	16±3	2.8±0.1	0.0±0.0	0.9±0.1
E202R	207±10	17.0±0.8	87±7	61±1.5	26±0.4	15.8±0.5
G232L	4.5±0.7	0.0±0.0	4.5±0.7	0.0±0.0	0.0±0.0	0.0±0.0
G232A	56±4	5.3±0.6	37±3	9.2±0.7	1.3±0.1	2.6±0.2
G235A	49±2	6.8±0.4	22±1	4.0±0.3	7.5±0.2	9.0±0.3
E202R+G235A	0.0±0.0	0.0± 0.0	0.0±0.0	0.0±0.0	0.0±0.0	0.0±0.0
V236A	129±1	15.5±0.2	79±0.5	28±0.4	0.0±0.0	6.0±0.1
E202R+V236A	48±3	7.9±0.6	30±2	8.5±0.6	0.0±0.0	1.8±0.1
A238M	194±12	13.3±0.8	79±4	65±7	24±1.7	13.1±0.9
E202R+A238M	50±2	3.5±0.5	22±1	15±0.6	6.4±0.2	3.6±0.1

[a] All data in % (total wildtype activity is set to 100%) by integration of peaks in GC. [b] Peak area for coeluting compounds **2** and **3**. Data represent mean and standard deviations from triplicates.

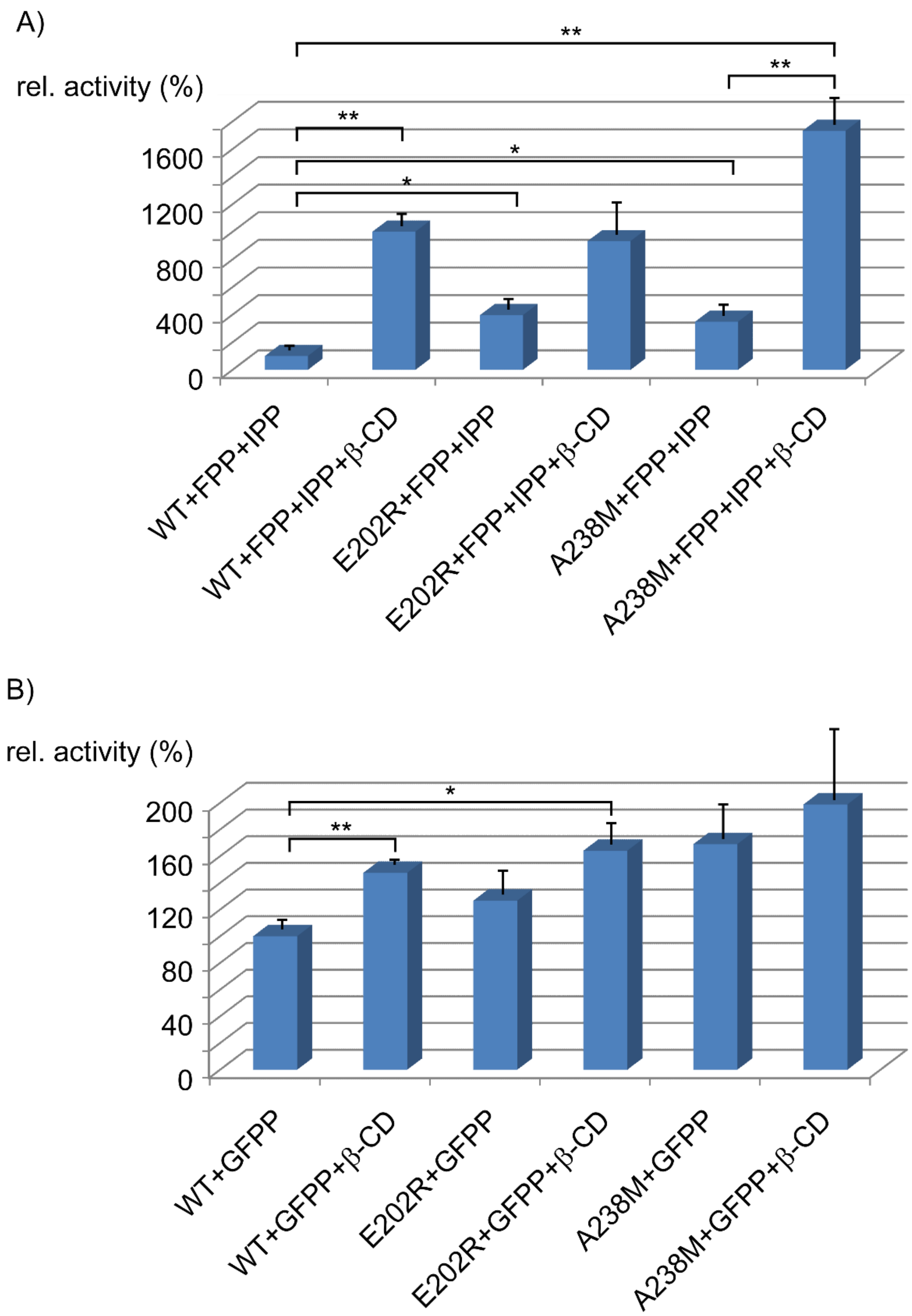

Figure S115. The activity of AcAS wildtype and the E202R and A238M enzyme variants with and without addition of β-cyclodextrin (β-CD). A) Activity with FPP and IPP, B) activity with GFPP. Student's *t*-tests were performed to investigate the significant difference between groups, n = 3. *: t < 0.05, **: t < 0.01.

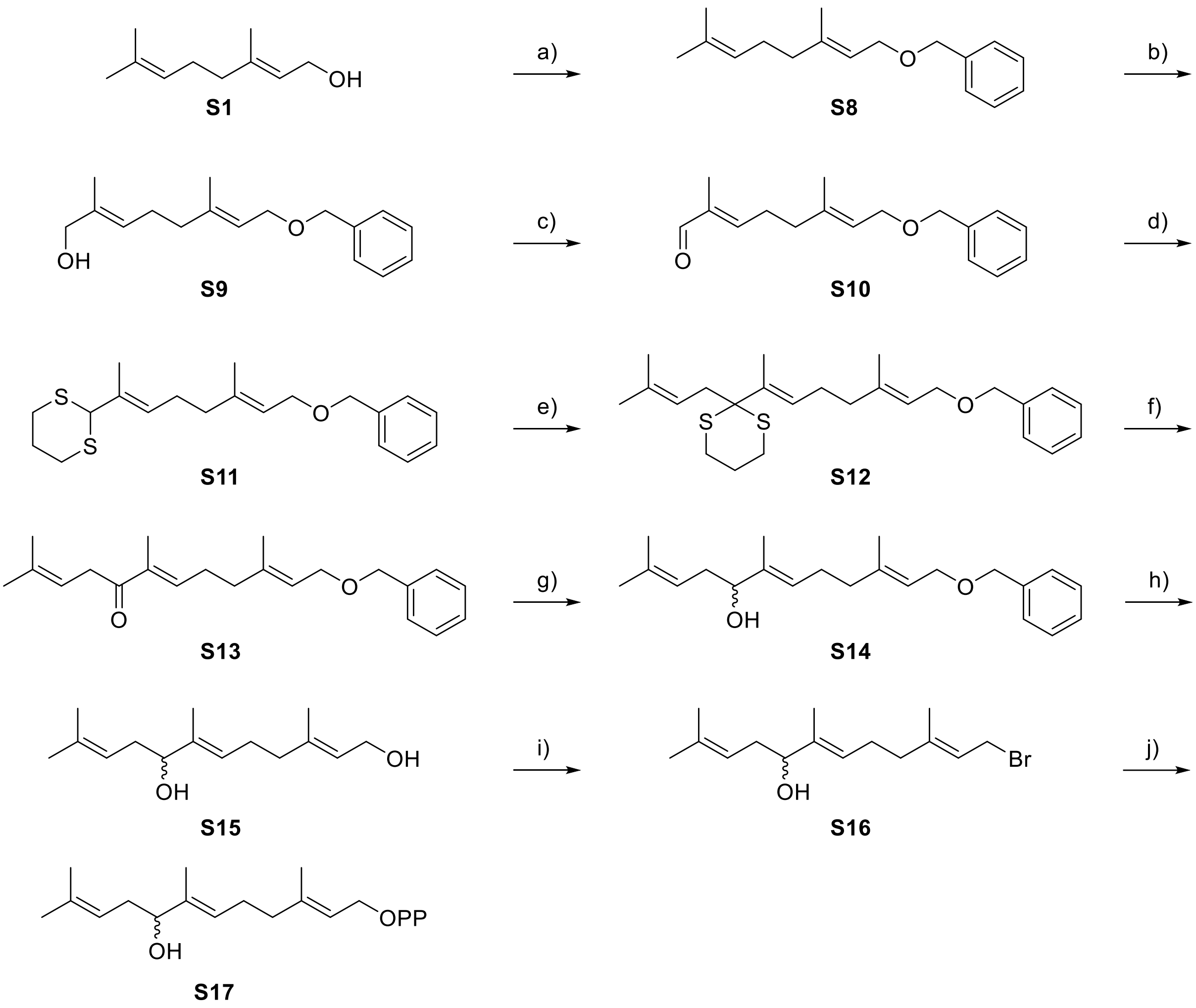

Scheme S2. Synthesis of 8-OH-FPP (**S17**). Reaction conditions: a) NaH, BnBr, DMF, 0 °C to room temperature, overnight, 95%; b) SeO_2, salicylic acid, *t*-BuOOH, CH_2Cl_2, 0 °C to room temperature, 24 h, 27%; c) IBX, DMSO, room temperature, 40 min, 78%; d) 1,3-propanedithiol, $BF_3{\cdot}OEt_2$, CH_2Cl_2, 0 °C, 1 h, 57%; e) *n*-BuLi, 1-bromo-3-methylbut-2-ene, THF, 0 °C to room temperature, overnight, 47%; f) $CaCO_3$, I_2, THF/H_2O, 0 °C, 2 h, 36%; g) DIBAL-H, CH_2Cl_2, 0 °C, 2 h, 73%; h) naphthalene, Li, THF, –78 °C to –50 °C, 2 h, 76%; i) PBr_3, THF, –15 °C, 1 h; j) $(NBu_4)_3HP_2O_7$, MeCN, room temperature, overnight, 73% over 2 steps.

Synthesis of (*E*)-(((3,7-dimethylocta-2,6-dien-1-yl)oxy)methyl)benzene (S8)

NaH (3.60 g, 60% in mineral oil, 90 mmol, 1.5 eq) was suspended in DMF (60 mL) at 0 °C. Geraniol **S1** (9.26 g, 60 mmol) was added dropwise and the mixture stirred for 0.5 h, followed by the dropwise addition of BnBr (12.31 g, 72 mmol, 1.2 eq) at 0 °C. The mixture was stirred overnight and allowed to warm to room temperature. The reaction was quenched by pouring into ice-water (200 mL) and extracted with Et_2O (3 x 100 mL). The organic layers were combined, washed with brine and dried with $MgSO_4$, the solvent was removed under reduced pressure. Compound **S8** (14.0 g, 30:1, 53.3 mmol, 95 %) was obtained via silica gel chromatography (cyclohexane/ethyl acetate, R_f = 0.35) as a colourless oil.

EI-MS (70 eV): *m*/*z* (%) = 173 (1), 162 (1), 153 (1), 136 (3), 123 (11), 107 (8), 91 (100), 77 (18), 69 (63), 53 (11), 41 (45). GC (HP5-MS): *I* = 1891. ^1H NMR (500 MHz, C_6D_6): δ = 7.35 – 7.31 (m, 2H), 7.21 – 7.13 (m, 2H), 7.12 – 7.07 (m, 1H), 5.57 – 5.50 (m, 1H), 5.19 – 5.12 (m, 1H), 4.39 (s, 2H), 3.99 (d, $^3J_{H,H}$ = 6.8 Hz, 2H), 2.14 – 2.07 (m, 2H), 2.06 – 1.99 (m, 2H), 1.64 (q, $^4J_{H,H}$ = 1.3 Hz, 3H), 1.52 (d, $^4J_{H,H}$ = 1.3 Hz, 3H), 1.50 (d, $^4J_{H,H}$ = 1.3 Hz, 3H) ppm. ^{13}C NMR (126 MHz, C_6D_6): δ = 139.64 (C_q), 139.27 (C_q), 131.44 (C_q), 128.53 (2 x CH), 127.83 (2 x CH),

127.55 (CH), 124.62 (CH), 122.26 (CH), 71.94 (CH_2), 66.93 (CH_2), 39.95 (CH_2), 26.84 (CH_2), 25.85 (CH_3), 17.75 (CH_3), 16.48 (CH_3) ppm.

Synthesis of (2*E*,6*E*)-8-(benzyloxy)-2,6-dimethylocta-2,6-dien-1-ol (S9)

To a mixture of SeO_2 (0.40 g, 3.63 mmol, 0.3 eq) and salicylic acid (0.62 g, 4.49 mmol, 0.37 eq) in CH_2Cl_2 (72 mL) was added *t*-BuOOH (10.78 mL, 5.5 M in decane, 59.29 mmol, 4.9 eq) dropwise. The mixture was stirred at room temperature for 0.5 h and cooled to 0 °C, followed by the addition of **S8** (2.93 g, 11.99 mmol) dropwise. The mixture was stirred for 24 h and without further cooling. The solvent was removed under reduced pressure. The residue was redissolved in MeOH/THF (1/4, 100 mL) and cooled to 0 °C, followed by the addition of $NaBH_4$ (2.74 g, 72.43 mmol, 6.0 eq) in 6 batches. The mixture was stirred at 0 °C for 1 h and then quenched by pouring into NH_4Cl solution (200 mL, 100 mL sat. NH_4Cl with 100 mL ice-water). The product was extracted with Et_2O (3 x 100 mL) and dried with $MgSO_4$. The solvent was removed under reduced pressure. Silica gel chromatography (cyclohexane/ethyl acetate, 5:1, R_f = 0.17) yielded **S9** (0.83 g, 3.19 mmol, 27%) as a colourless oil.

EI-MS (70 eV): *m/z* (%) = 229 (1), 174 (2), 151 (3), 139 (3), 134 (3), 123 (6), 107 (10), 95 (10), 91 (100), 81 (12), 77 (11), 65 (13), 55 (7), 43 (26). GC (HP5-MS): *I* = 2153. ^{1}H NMR (500 MHz, C_6D_6): δ = 7.38 – 7.31 (m, 2H), 7.22 – 7.15 (m, 2H), 7.14 – 7.06 (m, 1H), 5.52 (tq, $^3J_{H,H}$ = 6.6 Hz, $^4J_{H,H}$ = 1.3 Hz, 1H), 5.32 (tq, $^3J_{H,H}$ = 7.0 Hz, $^4J_{H,H}$ =1.4 Hz, 1H), 4.39 (s, 2H), 3.99 – 3.96 (d, $^3J_{H,H}$ = 6.5 Hz, 2H), 3.79 (d, $^3J_{H,H}$ = 3.8 Hz, 2H), 2.12 – 2.04 (m, 2H), 2.02 – 1.93 (m, 2H), 1.53 (d, $^4J_{H,H}$ = 1.2 Hz, 3H), 1.48 (d, $^4J_{H,H}$ = 1.3 Hz, 3H), 0.95 (br s, 1H) ppm. ^{13}C NMR (126 MHz, C_6D_6): δ = 139.49 (C_q), 139.04 (C_q), 135.83 (C_q), 128.57 (2 x CH), 127.90 (2 x CH), 127.64 (CH) 124.92 (CH), 122.42 (CH), 72.11 (CH_2), 68.74 (CH_2), 66.88 (CH_2), 39.52 (CH_2), 26.10 (CH_2), 16.42 (CH_3), 13.71 (CH_3) ppm.

Synthesis of (2*E*,6*E*)-8-(benzyloxy)-2,6-dimethylocta-2,6-dienal (S10)

2-Iodoxybenzoic acid (1.25 g, 4.47 mmol, 1.4 eq) was dissolved in DMSO (15 mL), followed by the addition of **S9** (0.83 g, 3.19 mmol) dropwise. The mixture was stirred at room temperature for 40 min, then diluted with Et_2O (50 mL) and cooled to 0 °C. Sat. $NaHCO_3$ (50 mL) was added to the mixture. The mixture was separated into two layers, and the aqueous phase was extracted with Et_2O (2 x 60 mL). The organic layers were combined and dried with $MgSO_4$. The solvent was removed under reduced pressure. Aldehyde **S10** (0.64 g, 2.48 mmol, 78%) was obtained via flash chromatography (cyclohexane/ethyl acetate, 5:1, R_f = 0.37) as a colourless oil.

EI-MS (70 eV): *m/z* (%) = 174 (4), 167 (2), 159 (1), 150 (11), 137 (8), 121 (6), 107 (7), 95 (9), 91 (100), 84 (13), 77 (14), 65 (16), 55 (20), 39 (9). GC (HP5-MS): *I* = 2148. ^{1}H NMR (500 MHz, C_6D_6): δ = 9.27 (s, 1H), 7.36 – 7.32 (m, 2H), 7.22 – 7.17 (m, 2H), 7.13 – 7.07 (m, 1H), 5.84 (tq, $^3J_{H,H}$ = 7.2 Hz, $^4J_{H,H}$ = 1.4 Hz, 1H), 5.45 – 5.39 (m, 1H), 4.38 (s, 2H), 3.94 (d, $^3J_{H,H}$ = 6.5 Hz, 2H), 2.00 – 1.93 (m, 2H), 1.84 – 1.76 (m, 2H), 1.60 (q, $^4J_{H,H}$ = 1.0 Hz, 3H), 1.38 (d, $^4J_{H,H}$ = 1.2 Hz, 3H) ppm. ^{13}C NMR (126 MHz, C_6D_6): δ = 193.84 (CH), 152.14 (CH), 139.81 (C_q), 139.37 (C_q), 137.74 (C_q), 128.63 (2 x CH), 127.82 (2 x CH), 127.72 (CH) 123.04 (CH), 72.25 (CH_2), 66.78 (CH_2), 37.90 (CH_2), 26.99 (CH_2), 16.29 (CH_3), 9.24 (CH_3).

Synthesis of 2-((2*E*,6*E*)-8-(benzyloxy)-6-methylocta-2,6-dien-2-yl)-1,3-dithiane (S11)

To a CH_2Cl_2 (25 mL, 0 °C) solution of **S10** (1.60 g, 6. 20 mmol) and 1,3-propanedithiol (0.80 g, 7.43 mmol, 1.2 eq) was added $BF_3{\cdot}OEt_2$ (0.26 g, 1.86mmol, 0.3 eq) dropwise. The mixture was stirred at 0 °C for 1 h and then quenched by pouring into ice-cold $NaHCO_3$ solution (50 mL $NaHCO_3$ with 100 mL ice-water). The product was extracted with Et_2O (3 x 60 mL). The organic extracts were combined, dried with $MgSO_4$ and concentrated by vacuum evaporation. Purification via silica gel chromatography (cyclohexane/ethyl acetate, 20:1, R_f = 0.26) provided compound **S11** (1.27 g, 3.54 mmol, 57%) as a colourless oil.

EI-MS (70 eV): m/z (%) = 257 (7), 242 (5), 227 (2), 174 (37), 165 (6), 159 (3), 148 (5), 133 (5), 119 (8), 119 (8), 99 (50), 91 (100), 77 (12), 65 (18), 55 (5), 41 (9). GC (HP5-MS): I = 2957. ^{1}H NMR (500 MHz, C_6D_6): δ = 7.40 – 7.34 (m, 2H), 7.22 – 7.16 (m, 2H), 7.14 – 7.07 (m, 1H), 5.80 (td, $^3J_{H,H}$ = 7.0 Hz, $^4J_{H,H}$ = 1.7 Hz, 1H), 5.50 (tq, $^3J_{H,H}$ = 6.6 Hz, $^4J_{H,H}$ =1.4 Hz, 1H), 4.50 (s, 1H), 4.42 (s, 2H), 3.99 (d, $^3J_{H,H}$ = 6.5 Hz, 2H), 2.54 – 2.40 (m, 2H), 2.40 – 2.28 (m, 2H), 2.09 – 1.98 (m, 2H), 1.96 – 1.90 (m, 2H), 1.82 (d, $^4J_{H,H}$ = 1.5 Hz, 3H), 1.65 – 1.53 (m, 1H), 1.43 (s, 3H), 1.42 – 1.37 (m, 1H) ppm. ^{13}C NMR (126 MHz, C_6D_6): δ = 139.72 (C_q), 138.78 (C_q), 133.87 (C_q), 129.53 (CH), 128.52 (2 x CH), 127.87 (2 x CH), 127.52 (CH), 122.62 (CH), 71.92 (CH_2), 66.88 (CH_2), 55.66 (CH), 39.09 (CH_2), 31.44 (2 x CH_2), 26.59 (CH_2), 25.69 (CH_2), 16.42 (CH_3), 15.35 (CH_3) ppm.

Synthesis of 2-((2*E*,6*E*)-8-(benzyloxy)-6-methylocta-2,6-dien-2-yl)-2-(3-methylbut-2-en-1-yl)-1,3-dithiane (S12)

Dithiane **S11** (1.27 g, 3.54 mmol) was dissolved in THF (15 mL) and cooled to 0 °C, followed by the addition of *n*-BuLi (2.43 mL, 1.6 M in hexane, 3.89 mmol, 1.1 eq) dropwise. After stirring at 0 °C for 1.5 h, 1-bromo-3-methylbut-2-ene (0.58 g, 3.89 mmol, 1.1 eq) was added dropwise and the mixture was stirred overnight without further cooling. The reaction was quenched by pouring onto aq. NH_4Cl (50 mL sat. NH_4Cl with 100 mL ice-water), the product was extracted with Et_2O (3 x 60 mL) and the combined extracts were dried with $MgSO_4$. The solvent was removed under reduced pressure, and the product **S12** (0.7 g, 1.68 mmol, 47%) was obtained via flash chromatography (cyclohexane/ethyl acetate, 20:1, R_f = 0.30).

EI-MS (70 eV): m/z (%) = 415 (1), 405 (1), 389 (1), 357 (1), 347 (36), 281 (3), 255 (4), 239 (22), 225 (3), 209 (2), 197 (8), 185 (2), 173 (2), 165 (6), 159 (11), 146 (4), 133 (9), 125 (17), 107 (10), 91 (100), 79 (11), 69 (8), 59 (3), 41 (12). GC (HP5-MS): I = 3237. HRMS (ESI): m/z = 439.2102 (calc. for $[C_{25}H_{36}OS_2 + Na]^+$: 439.2100). ^{1}H NMR (700 MHz, C_6D_6): δ = 7.36 – 7.31 (m, 2H), 7.20 – 7.17 (m, 2H), 7.12 – 7.08 (m, 1H), 6.19 (dq, $^3J_{H,H}$ = 7.0 Hz, $^4J_{H,H}$ = 1.2 Hz, 1H), 5.54 (tq, $^3J_{H,H}$ = 6.5 Hz, $^4J_{H,H}$ =1.3 Hz, 1H), 5.38 (tp, $^3J_{H,H}$ = 6.9 Hz, $^4J_{H,H}$ = 1.4 Hz, 1H), 4.39 (s, 2H), 3.98 (d, $^3J_{H,H}$ = 6.5 Hz, 2H), 2.78 (dt, $^3J_{H,H}$ = 6.9 Hz, $^4J_{H,H}$ = 1.2 Hz, 2H), 2.61 (ddd, $^2J_{H,H}$ = 14.5 Hz, $^3J_{H,H}$ = 11.8 Hz, $^4J_{H,H}$ = 2.7 Hz, 2H), 2.28 – 2.23 (m, 2H), 2.19 (q, $^3J_{H,H}$ = 7.3 Hz, 2H), 2.10 – 2.00 (m, 2H), 1.83 (d, $^4J_{H,H}$ = 1.1 Hz, 3H), 1.71 – 1.65 (m, 1H), 1.64 (d, $^4J_{H,H}$ = 1.4 Hz, 3H), 1.55 (d, $^4J_{H,H}$ = 1.4 Hz, 3H), 1.54 – 1.50 (m, 1H), 1.49 (d, $^4J_{H,H}$ = 1.3 Hz, 3H) ppm. ^{13}C NMR (176 MHz, C_6D_6): δ = 139.51 (C_q), 138.85 (C_q), 134.12 (C_q), 133.38 (C_q), 131.71 (CH), 128.56 (2 x CH), 127.85 (2 x CH), 127.64 (CH) 122.64 (CH), 119.14 (CH), 72.18 (CH_2), 66.99 (CH_2), 61.07 (C_q), 39.54 (CH_2), 39.24 (CH_2), 27.56 (2 x CH_2), 27.40 (CH_2), 26.05 (CH_3), 25.70 (CH_2), 18.32 (CH_3), 16.36 (CH_3), 13.93 (CH_3) ppm.

Synthesis of (6*E*,10*E*)-12-(benzyloxy)-2,6,10-trimethyldodeca-2,6,10-trien-5-one (S13)

Compound **S12** (0.70 g, 1.68 mmol) and $CaCO_3$ (0.84 g, 8.40 mmol, 5.0 eq) were added into THF/H_2O (19 mL THF with 4.8 mL H_2O) and cooled to 0 °C. I_2 (0.85 g, 3.36 mmol, 2.0 eq, in 4.8 mL THF) was added dropwise into the mixture followed by stirring at 0 °C for 2 h. The reaction was quenched by the addition of sat. $Na_2S_2O_3$ (20 mL) at 0 °C, and extracted with Et_2O (3 x 60 mL). The combined organic layers were washed with brine and dried with $MgSO_4$. The solvent was removed under reduced pressure. Compound **S13** (0.20 g, 0.61 mmol, 36%) was obtained via silica gel chromatography (cyclohexane/ethyl acetate, 10:1, R_f = 0.29) as a colourless oil.

EI-MS (70 eV): m/z (%) = 218 (1), 203 (2), 175 (4), 152 (5), 137 (1), 121 (3), 109 (16), 97 (7), 91 (100), 79 (8), 69 (8), 55 (4), 41 (12). GC (HP5-MS): I = 2564. ^{1}H NMR (500 MHz, C_6D_6): δ = 7.34 (d, $^3J_{H,H}$ = 7.2 Hz, 2H), 7.36 – 7.32 (m, 2H), 7.13 – 7.07 (m, 1H), 6.36 (td, $^3J_{H,H}$ = 7.1 Hz, $^4J_{H,H}$ = 1.6 Hz, 1H), 5.55 (tp, $^3J_{H,H}$ = 7.2 Hz, $^4J_{H,H}$ = 1.4 Hz, 1H), 5.51 – 5.45 (m, 1H), 4.39 (s, 2H), 3.96 (d, $^3J_{H,H}$ = 6.5 Hz, 2H), 3.29 (d, $^3J_{H,H}$ = 7.0 Hz, 2H), 2.07 – 1.99 (m, 2H), 1.92 – 1.85 (m, 2H), 1.79 (d, $^4J_{H,H}$ = 1.5 Hz, 3H), 1.63 (d, $^4J_{H,H}$ = 1.6 Hz, 3H), 1.52 (s, 3H), 1.42 (s, 3H) ppm. ^{13}C NMR (126 MHz, C_6D_6): δ = 198.47 (C_q), 140.99 (CH), 139.40 (C_q), 138.10 (C_q), 137.56

(C_q), 133.92 (C_q), 128.61 (2 x CH), 127.82 (2 x CH), 127.70 (CH), 123.00 (CH), 118.56 (CH), 72.22 (CH_2), 66.81 (CH_2), 38.33 (CH_2), 37.62 (CH_2), 27.22 (CH_2), 25.80 (CH_3), 18.05 (CH_3), 16.30 (CH_3), 11.71 (CH_3) ppm.

Synthesis of (6*E*,10*E*)-12-(benzyloxy)-2,6,10-trimethyldodeca-2,6,10-trien-5-ol (S14)
To a CH_2Cl_2 (5 mL, 0 °C) solution of **S13** (100 mg, 0.30 mmol) was added DIBAL-H (0.39 mL, 1 M in hexane, 0.39 mmol, 1.3 eq) dropwise. The mixture was stirred at 0 °C for 2 h, and then quenched by the addition of saturated aq. Na-K-tartrate solution (5 mL). The mixture was stirred at room temperature for 30 min and extracted with Et_2O (3 x 50 mL). The combined organic layers were dried with $MgSO_4$ and concentrated by vacuum evaporation. The product **S14** (72 mg, 0.22 mmol, 73%) was purified by silica gel chromatography (cyclohexane/ethyl acetate, 3:1, R_f = 0.46) and obtained as a colourless oil.
EI-MS (70 eV): *m*/*z* (%) = 259 (2), 241 (2), 151 (17), 133 (6), 123 (23), 107 (9), 91 (100), 81 (27), 69 (29), 55 (20), 71 (23). GC (HP5-MS): *I* = 2549. HRMS (ESI): *m*/*z* = 351.2300 (calc. for $[C_{22}H_{32}O_2 + Na]^+$: 351.2295). ^{1}H NMR (700 MHz, C_6D_6): δ = 7.36 – 7.34 (m, 2H), 7.21 – 7.17 (m, 2H), 7.12 – 7.08 (m, 1H), 5.53 (tq, $^3J_{H,H}$ = 6.6 Hz, $^4J_{H,H}$ = 1.3 Hz, 1H), 5.39 (tp, $^3J_{H,H}$ = 7.1 Hz, $^4J_{H,H}$ = 1.4 Hz, 1H), 5.24 – 5.18 (m, 1H), 4.40 (s, 2H), 3.99 – 3.97 (m, 2H), 3.97 – 3.93 (m, 1H), 2.39 – 2.32 (m, 1H), 2.29 – 2.23 (m, 1H), 2.16 – 2.06 (m, 2H), 2.03 – 1.96 (m, 2H), 1.64 (d, $^4J_{H,H}$ = 1.3 Hz, 3H), 1.60 (d, $^4J_{H,H}$ = 1.0 Hz, 3H), 1.53 (d, $^4J_{H,H}$ = 1.3 Hz, 3H), 1.49 (d, $^4J_{H,H}$ = 1.3 Hz, 3H), 1.28 (d, $^3J_{H,H}$ = 3.2 Hz, 1H) ppm. ^{13}C NMR (176 MHz, C_6D_6): δ = 139.52 (C_q), 139.04 (C_q), 138.11 (C_q), 133.61 (C_q), 128.56 (2 x CH), 127.89 (2 x CH) 127.62 (CH), 125.32 (CH), 122.45 (CH), 121.41 (CH), 77.42 (CH), 72.13 (CH_2), 66.88 (CH_2), 39.54 (CH_2), 34.83 (CH_2), 26.11 (CH_2), 25.97 (CH_3), 17.97 (CH_3), 16.42 (CH_3), 11.80 (CH_3) ppm.

Synthesis of (2*E*,6*E*)-3,7,11-trimethyldodeca-2,6,10-triene-1,8-diol (S15)
Naphthalene (1.92 g, 14.96 mmol, 37.4 eq) was dissolved in THF (10 mL), followed by the addition of freshly cut lithium metal (69 mg, 10 mmol, 25 eq). The mixture was stirred at room temperature for 1.5 h to form a black solution, and then transferred into a syringe. Compound **S14** (0.13 g, 0.4 mmol) was dissolved in THF (5 mL) and cooled to –78 °C, followed by the slow addition of the black lithium naphthalenide solution. The mixture was stirred for 2 h, and the temperature was allowed to warm to –50 °C. The mixture was cooled to –78 °C, and sat. NH_4Cl (5 mL) was added dropwise to the mixture. The reaction was diluted with H_2O (50 mL) and extracted with Et_2O (3 x 50 mL). The combined organic layers were dried with $MgSO_4$. The solvent was removed under reduced pressure. Compound **S15** (70 mg, 0.29 mmol, 76%) was obtained via flash chromatography (pentane/Et_2O, 1:4, R_f = 0.31) as a colourless oil.
EI-MS (70 eV): *m*/*z* (%) = 151 (36), 131 (2), 123 (29), 107 (14), 93 (100), 81 (68), 69 (54), 55 (59), 41 (86). GC (HP5-MS): *I* = 1925. ^{1}H NMR (500 MHz, C_6D_6): δ = 5.40 – 5.34 (m, 2H), 5.23 – 5.18 (m, 1H), 4.01 – 3.92 (m, 3H), 2.41 – 2.31 (m, 1H), 2.30 – 2.22 (m, 1H), 2.16 – 2.02 (m, 2H), 1.99 – 1.93 (m, 2H), 1.64 (d, $^4J_{H,H}$ = 1.3 Hz, 3H), 1.60 (d, $^4J_{H,H}$ = 1.3 Hz, 3H), 1.54 (d, $^4J_{H,H}$ = 1.3 Hz, 3H), 1.46 (d, $^4J_{H,H}$ = 1.3 Hz, 3H), 1.43 (br s, 1H), 0.85 (br s, 1H) ppm. ^{13}C NMR (126 MHz, C_6D_6): δ = 138.04 (C_q), 137.68 (C_q), 133.71 (C_q), 125.42 (CH), 125.31 (CH), 121.34 (CH), 77.45 (CH), 59.32 (CH_2), 39.44 (CH_2), 34.81 (CH_2), 26.03 (CH_2), 25.97 (CH_3), 17.98 (CH_3), 16.10 (CH_3), 11.81 (CH_3) ppm.

Synthesis of trisammonium 8-hydroxylfarnesyl diphosphate (S17)
Diol **S15** (30 mg, 0.13 mmol) was dissolved in THF (2 mL) and cooled to –15 °C, followed by the addition of PBr_3 (12 mg, 0.05 mmol, 0. 35 eq). The solution was stirred at –15 °C for 1 h and quenched by pouring onto ice-water (100 mL). The product was extracted with Et_2O (3 x 50 mL), washed with brine and dried with $MgSO_4$. The solvent was removed under reduced pressure and the bromide **S16** (30 mg, 0.10 mmol, 77%) was obtained via flash chromatography (pentane/Et_2O, 1:1, R_f = 0.64).

$(NBu_4)HP_2O_7$ (140 mg, 0.15 mmol) was dissolved in MeCN (0.5 mL), followed by the addition of **S16** (30 mg, 0.10 mmol, in 0.5 mL MeCN) dropwise. The mixture was stirred at room temperature overnight. The solvent was removed under reduced pressure. The residue was loaded onto an ion exchange resin column (DOWEX 50W-X8, 100-200 mesh, NH_4^+ form), followed by washing with two column volumes of elution buffer (25 mm NH_4HCO_3 in 2% *i*PrOH/H_2O). The eluate was freeze-dried to provide compound **S17** (43 mg, 73% over two steps) as a white powder.
HRMS (ESI): *m*/*z* = 397.1189 (calc. for $[C_{15}H_{27}O_8P_2]^-$: 397.1187). ^{1}H NMR (500 MHz, D_2O): δ = 5.41 (tq, $^3J_{H,H}$ = 7.1 Hz, $^4J_{H,H}$ = 1.6 Hz, 1H), 5.38 – 5.33 (m, 1H), 5.03 (tp, $^3J_{H,H}$ = 7.2 Hz, $^4J_{H,H}$ = 1.5 Hz, 1H), 4.41 (t, $^3J_{H,H}$ = 6.6 Hz, 2H), 3.97 (t, $^3J_{H,H}$ = 6.9 Hz, 1H), 2.21 (q, $^3J_{H,H}$ = 6.5 Hz, 2H), 2.16 – 2.09 (m, 2H), 2.10 – 2.03 (m, 2H), 1.67 (d, $^4J_{H,H}$ = 1.3 Hz, 3H), 1.64 (d, $^4J_{H,H}$ = 1.4 Hz, 4H), 1.57 (d, $^4J_{H,H}$ = 1.3 Hz, 3H), 1.55 (d, $^4J_{H,H}$ = 1.3 Hz, 3H) ppm. ^{13}C NMR (126 MHz, D_2O): δ = 142.52 (C_q), 136.23 (C_q), 135.15 (C_q), 126.78 (CH), 119.92 (CH), 119.89 (d, $^3J_{C,P}$ = 8.3 Hz), 77.33 (CH), 62.40 (d, $^2J_{C,P}$ = 5.2 Hz), 38.44 (CH_2), 32.89 (CH_2), 25.26 (CH_2), 24.98 (CH_3), 17.18 (CH_3), 15.61 (CH_3), 10.58 (CH_3) ppm. ^{31}P NMR (202 MHz, D_2O): δ = –6.46 (d, $^2J_{P,P}$ = 21.4 Hz), –10.24 (d, $^2J_{P,P}$ = 21.6 Hz) ppm.

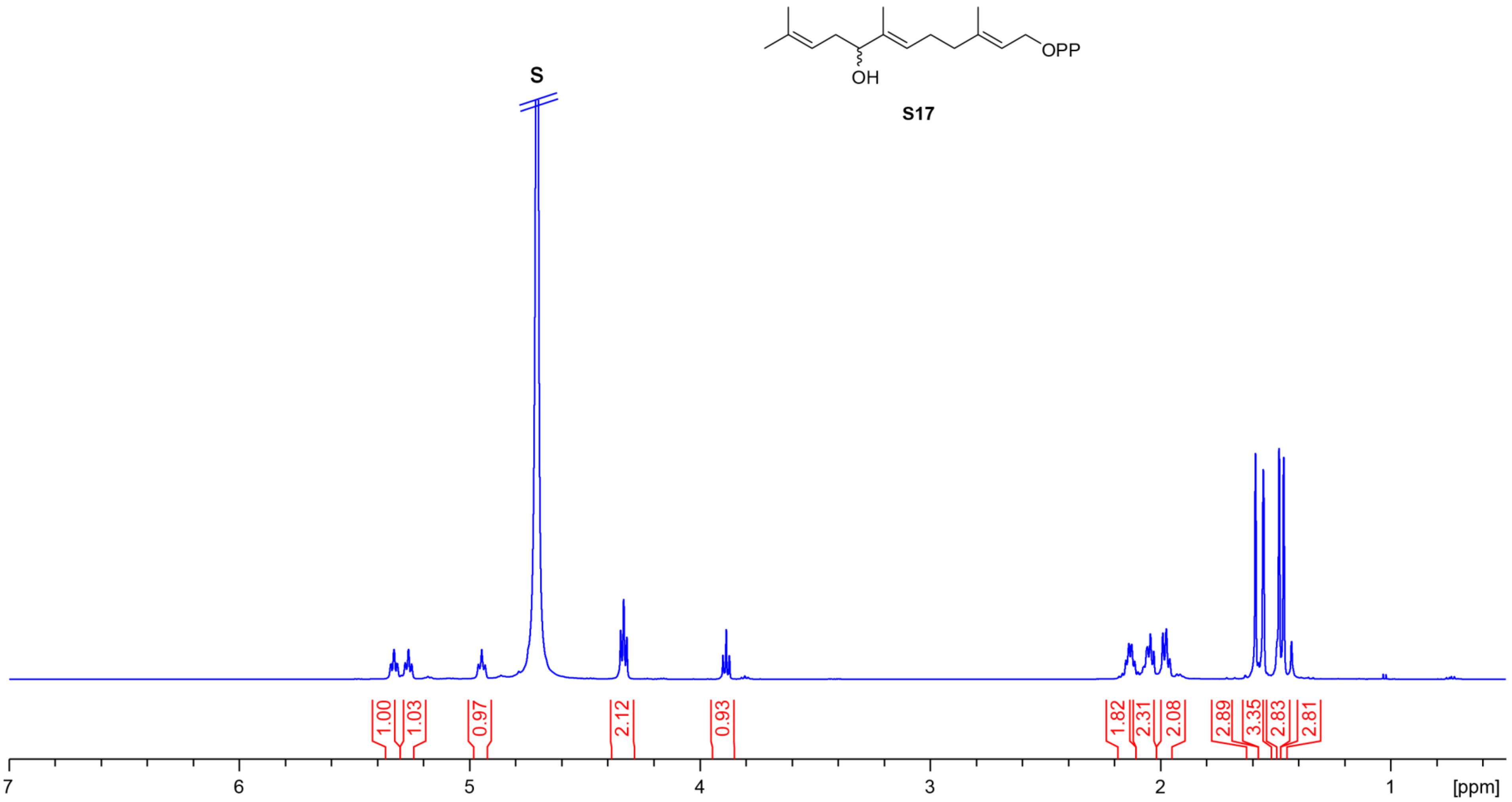

Figure S116. ^{1}H-NMR spectrum of **S17** (500 MHz, D_2O).

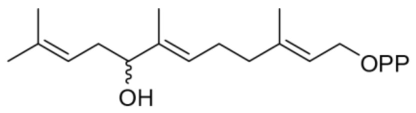

S17

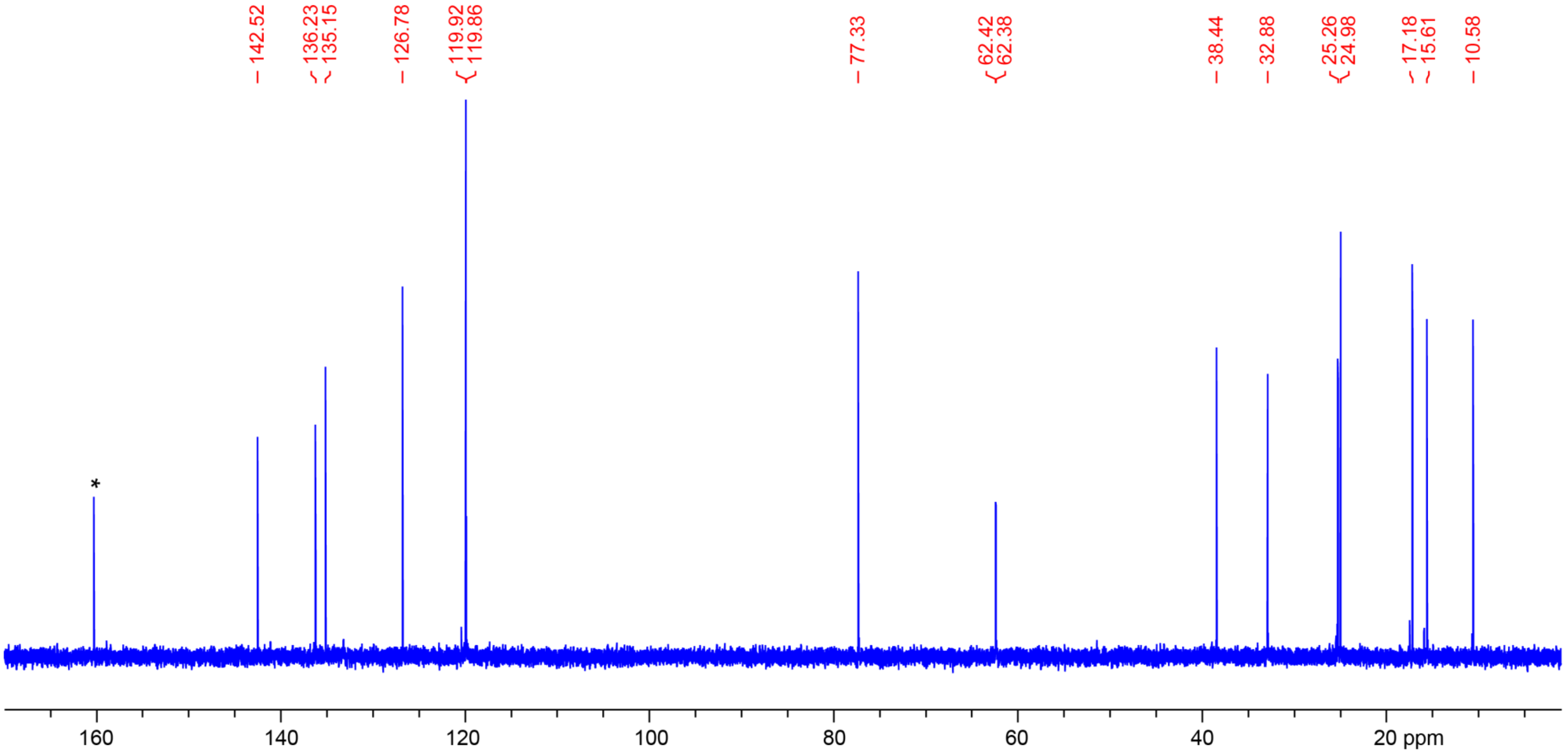

Figure S117. ^{13}C-NMR spectrum of **S17** (126 MHz, D_2O). The asterisk indicates a peak from an unknown contaminant.

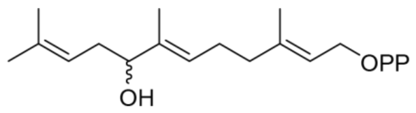

S17

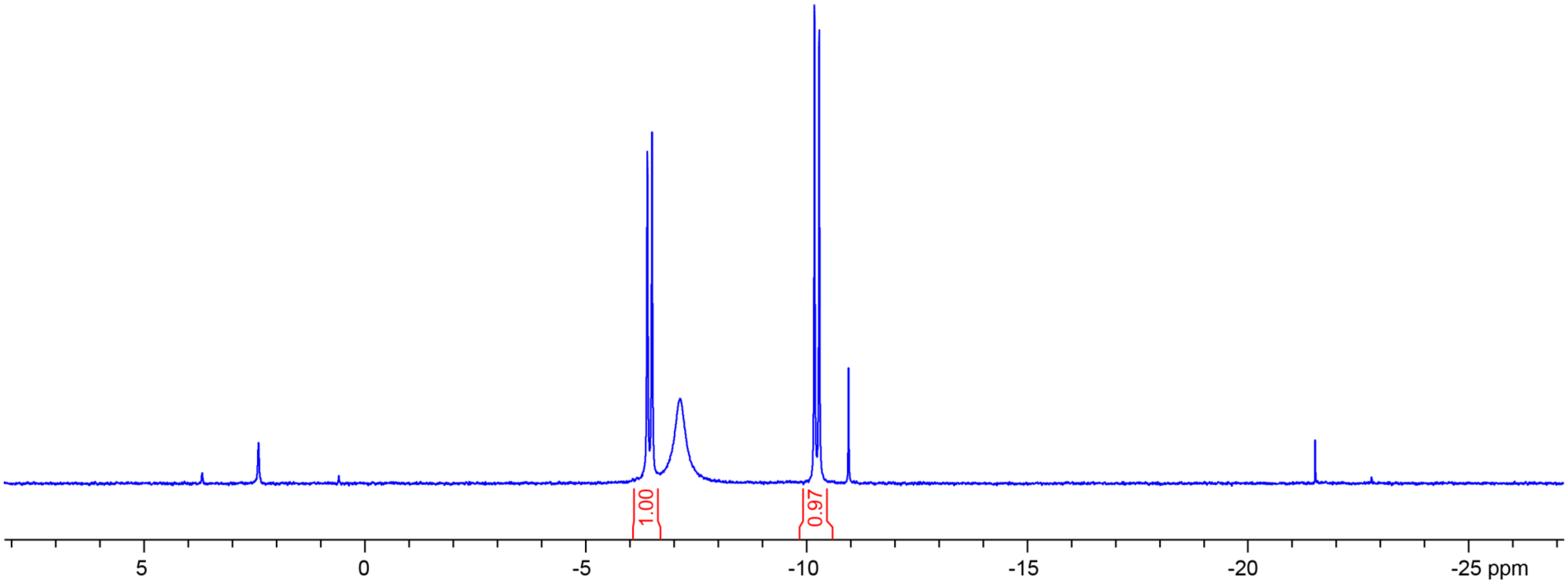

Figure S118. ^{31}P-NMR spectrum of **S17** (202 MHz, D_2O).

Enzymatic conversion of 8-OH-FPP (S17) with AcAS

8-Hydroxy-FPP trisammonium salt (300 mg, 0.67 mmol) and IPP trisammonium salt (300 mg, 1.00 mmol) were each dissolved in 25 mM NH_4HCO_3 (30 mL). For the following enzyme reactions enzyme preparation from 30 L of expression culture was used. A total number of 600 small scale reactions, each containing incubation buffer (800 μL; 50 mM TRIS, 10 mM $MgCl_2$, 20% glycerol, pH = 8.2), AcAS enzyme preparation concentrated by ultrafiltration (100 μL, 2 mg mL^{-1} in elution buffer), (8-OH)FPP solution (50 μL) and IPP solution (50 μL), were incubated at 30 °C for 24 h. The reaction mixtures were combined and extracted with hexane for two times. The combined organic layers were washed with brine, dried with Na_2SO_4, and concentrated under reduced pressure. The residue was subjected to silica gel chromatography using hexane / diethyl ether (90 : 10) for compound elution. The mixture of six sesterterpenes was collected and separated by semi-preparative HPLC on a KNAUER Eurospher II 110-5 C18P column (5 μm, 250 x 8 mm) using acetonitrile (90% isocratic, 5 mL min^{-1}, 166 bar) for elution of the pure compounds.

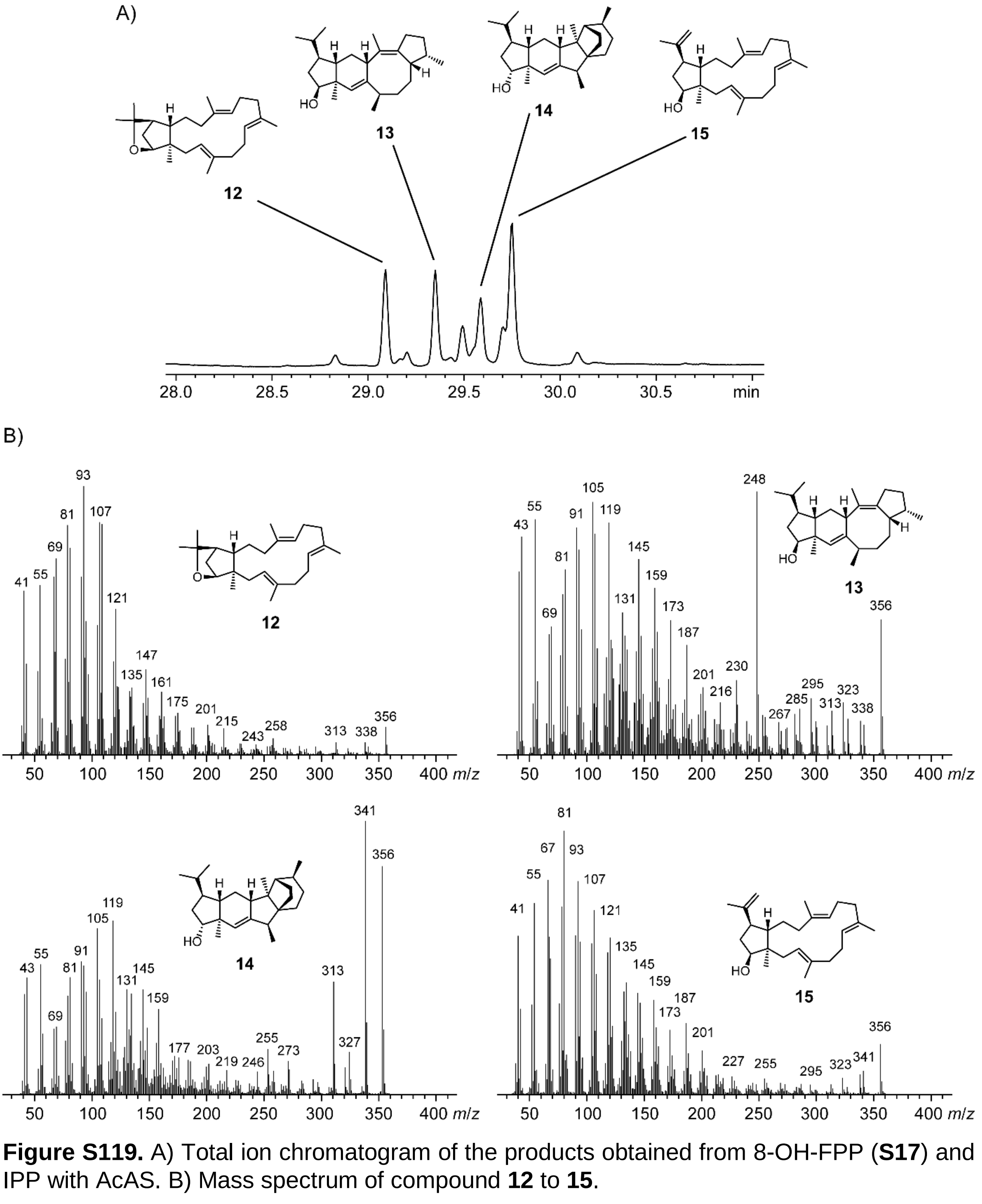

Figure S119. A) Total ion chromatogram of the products obtained from 8-OH-FPP (**S17**) and IPP with AcAS. B) Mass spectrum of compound **12** to **15**.

Calidoustatriene ether (12). TLC (cyclohexane/ethyl acetate [80/20]): R_f = 0.75. $[\alpha]_D^{25}$ = –3.8 (*c* 0.05, cyclohexane). HRMS (EI) *m*/*z*: $[M]^+$ calcd. for $C_{25}H_{40}O^+$ 356.3074; found 356.3078. GC (HP5-MS): *I* = 2598. MS (EI, 70 eV): *m*/*z* (%) = 356 (19), 338 (7), 323 (s), 313 (5), 295 (4), 287 (3), 269 (3), 258 (8), 243 (6), 229 (5), 215 (10), 201 (13), 187 (12), 175 (19), 161 (28), 147 (33), 135 (29), 121 (62), 107 (93), 93 (100), 81 (85), 69 (77), 55 (63), 41 (60). IR (diamond ATR): $\tilde{\nu}$ / cm^{-1} = 2954 (s), 2925 (s), 2853 (s), 2024 (w), 1730 (m), 1441 (m), 1381 (m), 1291 (w), 1261 (w), 1120 (m), 1090 (m), 1037 (m), 1019 (w), 798 (m), 573 (w), 541 (w), 513 (w).

16-*epi*-Aspergilol C (13). TLC (cyclohexane/ethyl acetate [80/20]): R_f = 0.60. $[\alpha]_D^{25}$ = –171.0 (*c* 0.03, cyclohexane). HRMS (EI) *m*/*z*: $[M]^+$ calcd. for $C_{25}H_{40}O^+$ 356.3074; found 356.3077. GC (HP5-MS): *I* = 2631. MS (EI, 70 eV): *m*/*z* (%) = 356 (58), 341 (15), 323 (21), 313 (18), 295 (22), 285 (18), 274 (11), 267 (11), 255 (14), 248 (100), 239 (14), 230 (26), 216 (20), 201 (25), 187 (41), 173 (46), 159 (60), 145 (72), 131 (50), 119 (82), 105 (89), 91 (81), 81 (62), 69 (43), 55 (79), 43 (80). IR (diamond ATR): $\tilde{\nu}$ / cm^{-1} = 3008 (w), 2953 (m), 2930 (m), 2873 (m), 2362 (w), 2335 (w), 2127 (w), 1978 (w), 1739 (s), 1446 (m), 1367 (s), 1217 (s), 1094 (w), 1037 (w), 888 (w), 795 (w), 518 (w).

16-Hydroxycalidoustene (14). TLC (cyclohexane/ethyl acetate [80/20]): R_f = 0.54. $[\alpha]_D^{25}$ = –81.0 (*c* 0.03, cyclohexane). HRMS (EI) *m*/*z*: $[M]^+$ calcd. for $C_{25}H_{40}O^+$ 356.3074; found 356.3077. GC (HP5-MS): *I* = 2662. MS (EI, 70 eV): *m*/*z* (%) = 356 (80), 341 (100), 327 (16), 313 (37), 295 (5), 286 (4), 273 (11), 255 (13), 246 (7), 229 (3), 219 (6), 201 (7), 187 (10), 171 (10), 159 (24), 145 (33), 131 (33), 119 (46), 105 (43), 91 (37), 81 (29), 69 (17), 55 (31), 43 (30). IR (diamond ATR): $\tilde{\nu}$ / cm^{-1} = 2953 (s), 2925 (s), 2869 (m), 2359 (w), 2152 (w), 1994 (w), 1740 (m), 1458 (m), 1375 (m), 1261 (w), 1208 (m), 1039 (m), 796 (w), 669 (w).

16-Hydroxycalidoustatetraene (15). TLC (cyclohexane/ethyl acetate [80/20]): R_f = 0.54. $[\alpha]_D^{25}$ = –31.0 (*c* 0.04, cyclohexane). HRMS (EI) *m*/*z*: $[M]^+$ calcd. for $C_{25}H_{40}O^+$ 356.3074; found 356.3077. GC (HP5-MS): *I* = 2684. MS (EI, 70 eV): *m*/*z* (%) = 356 (18), 341 (10), 323 (5), 313 (4), 295 (3), 287 (3), 269 (3), 255 (5), 248 (5), 241 (4), 227 (7), 215 (7), 201 (16), 187 (27), 173 (23), 159 (36), 145 (39), 133 (40), 121 (59), 107 (72), 93 (81), 81 (100), 67 (86), 55 (78), 41 (60). IR (diamond ATR): $\tilde{\nu}$ / cm^{-1} = 2952(s), 2926 (s), 2855 (m), 2371 (m), 2340 (m), 1730 (w), 1640 (w), 1443 (m), 1378 (m), 1260 (w), 1096 (m), 1017 (m), 885 (m), 798 (m), 669 (m).

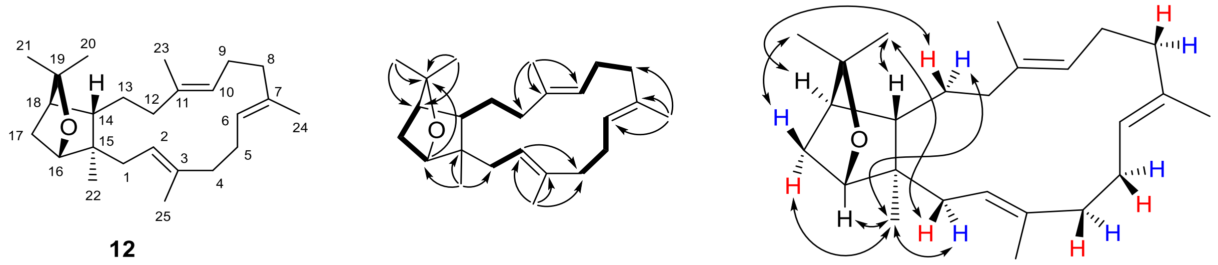

Figure S120. Structure elucidation of **12**. Bold: $^{1}H,^{1}H$-COSY correlations, single-headed arrows: key HMBC correlations, and double headed arrows: NOESY correlations.

Table S11. NMR data of calidoustatriene ether (**12**) in C_6D_6 recorded at 298 K.

C[a]		δ_C[b]	δ_H[a,b]
1	CH_2	35.5	1.86 (m, 1H, H_α)<br>3.08 (dd, $J_{H,H}$ = 16.3, 6.4, 1H, H_β)
2	CH	123.5	5.15 (m, 1H)
3	C_q	134.8	–
4	CH_2	39.9	2.05 (m, 1H, H_α*)<br>2.16 (m, 1H, H_β*)
5	CH_2	25.5	2.11 (m, 1H, H_α*)<br>2.23 (m, 1H, H_β*)
6	CH	123.7	5.18 (m, 1H)
7	C_q	133.4	–
8	CH_2	39.2	1.96 (m, 1H, H_β*)<br>2.13 (m, 1H, H_α*)
9	CH_2	24.9	2.04 (m, 1H)<br>2.36 (dtd, $^2J_{H,H}$ = 14.2, $^3J_{H,H}$ = 10.1, 1.7, 1H)
10	CH	126.3	5.17 (m, 1H)
11	C_q	133.8	–
12	CH_2	38.4	1.92 (m, 1H)<br>2.05 (m, 1H)
13	CH_2	28.8	1.08 (m, 1H, H_β)<br>1.48 (ddt, $^2J_{H,H}$ = 14.3, $^3J_{H,H}$ = 10.9, 3.6, 1H, H_α)
14	C_q	39.0	–
15	C_q	46.5	–
16	CH	86.5	3.63 (d, $J_{H,H}$ = 2.0, 1H)
17	CH_2	33.5	1.61 (m, 1H, H_α)<br>1.87 (m, 1H, H_β)
18	CH	50.3	1.74 (s, 1H)
19	C_q	80.2	–
20	CH_3	24.5	1.32 (s, 3H)
21	CH_3	30.2	1.21 (s, 3H)
22	CH_3	21.0	0.77 (d, $J_{H,H}$ = 0.9, 3H)
23	CH_2	17.0	1.62 (s, 3H)
24	CH_3	16.8	1.57 (s, 3H)
25	CH_3	15.9	1.59 (s, 3H)

[a] Carbon numbering and colour code for hydrogens as in Figure S119, [b] chemical shifts δ in ppm, multiplicity: s = singlet, d = doublet, t = triplet, m = multiplet, coupling constants J are given in Hertz. Asterisks indicate diastereotopic hydrogen assignments based on labelling experiments.

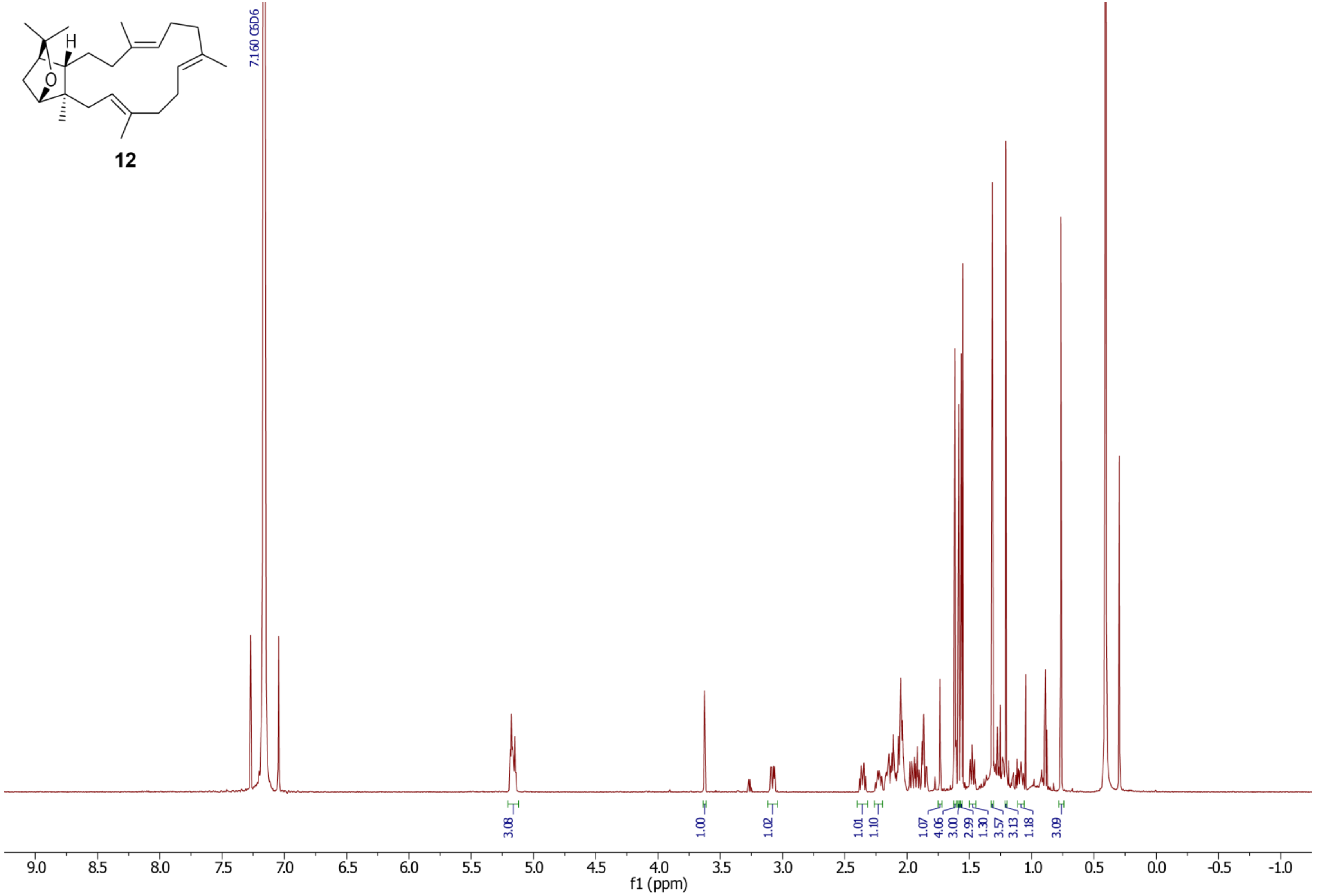

Figure S121. ^{1}H-NMR spectrum of **12** (700 MHz, C_6D_6).

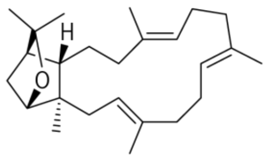

12

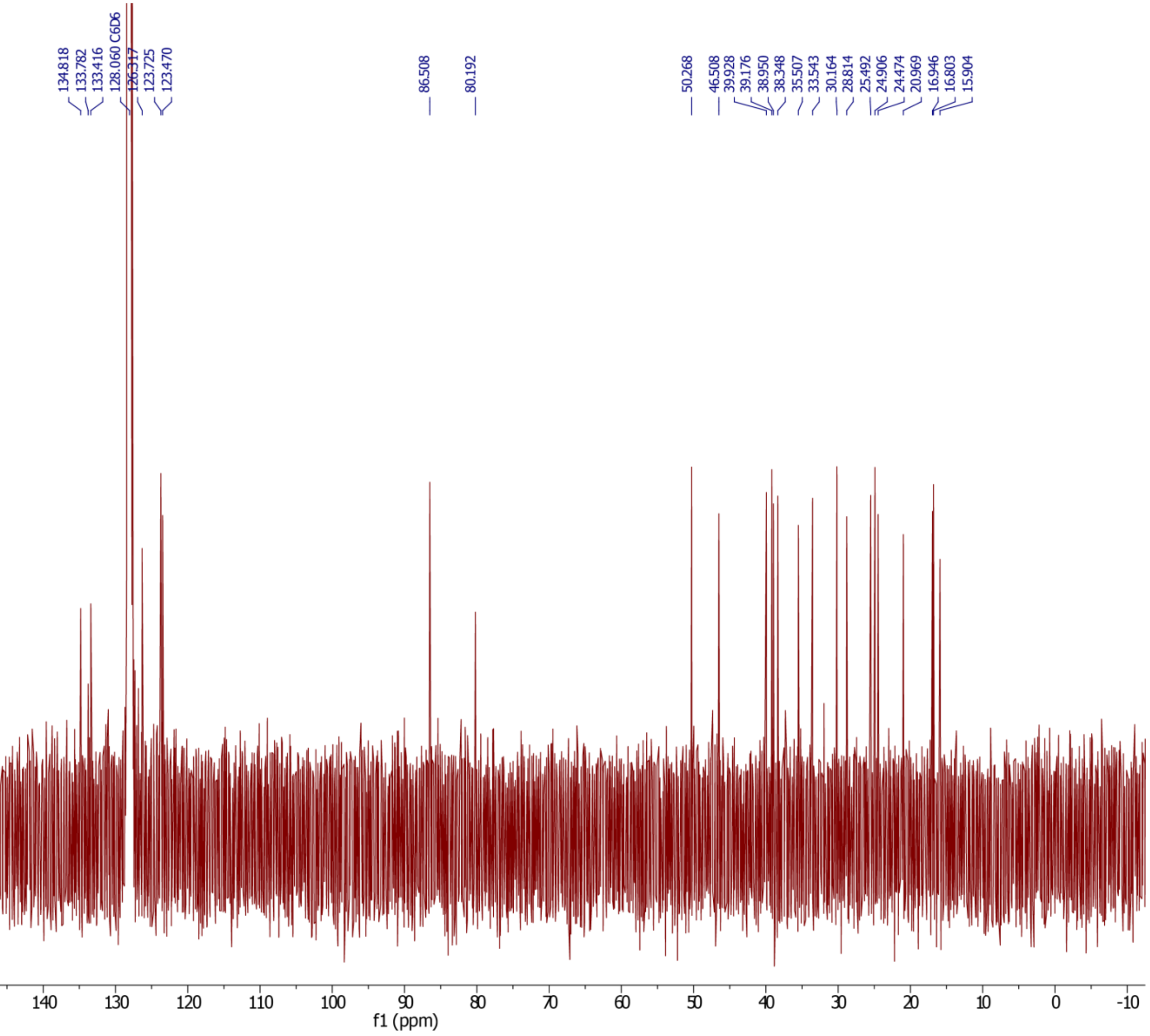

Figure S122. ^{13}C-NMR spectrum of **12** (176 MHz, C_6D_6).

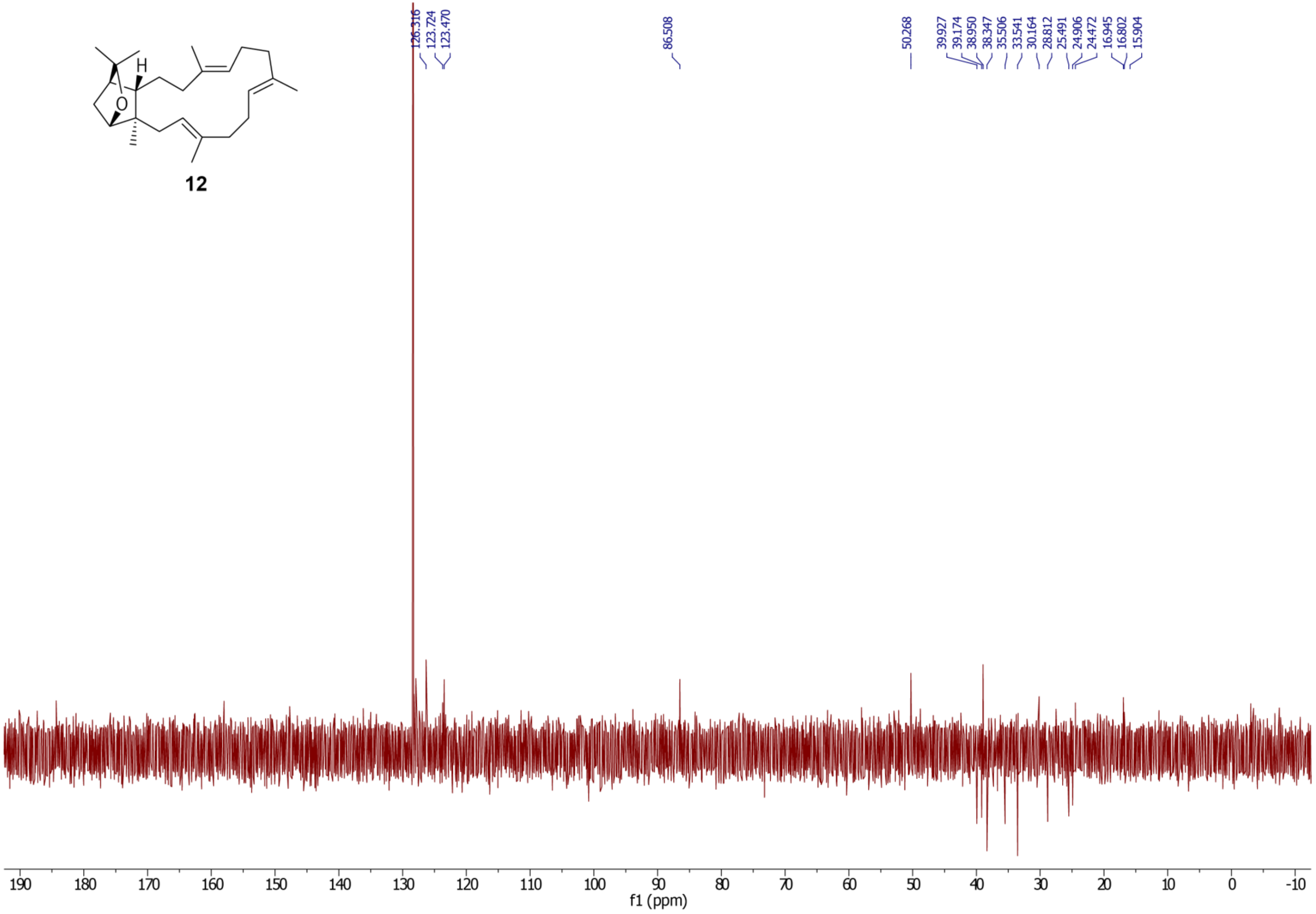

Figure S123. DEPT spectrum of **12** (176 MHz, C_6D_6).

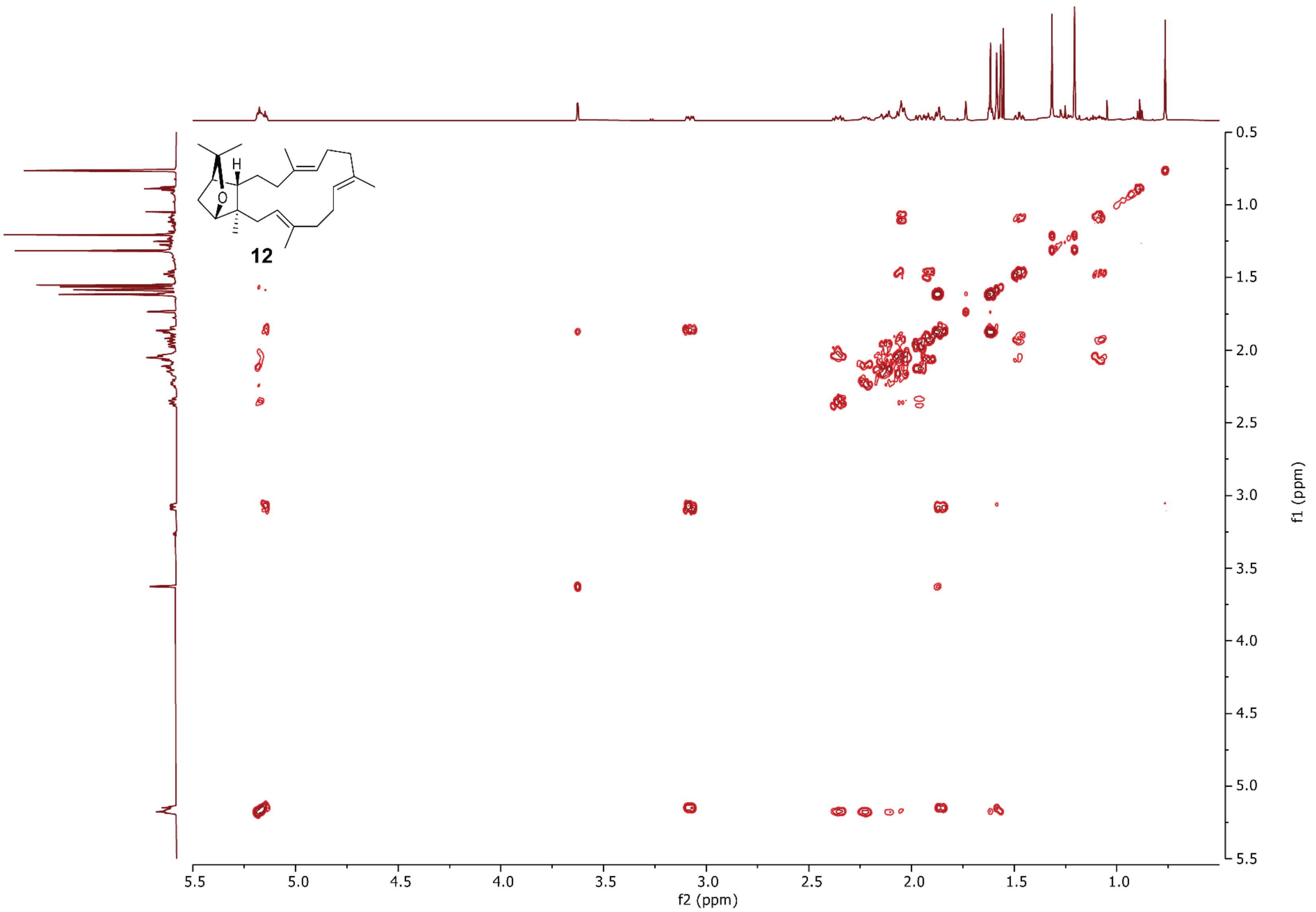

Figure S124. $^{1}H,^{1}H$-COSY spectrum of **12** (700 MHz, C_6D_6).

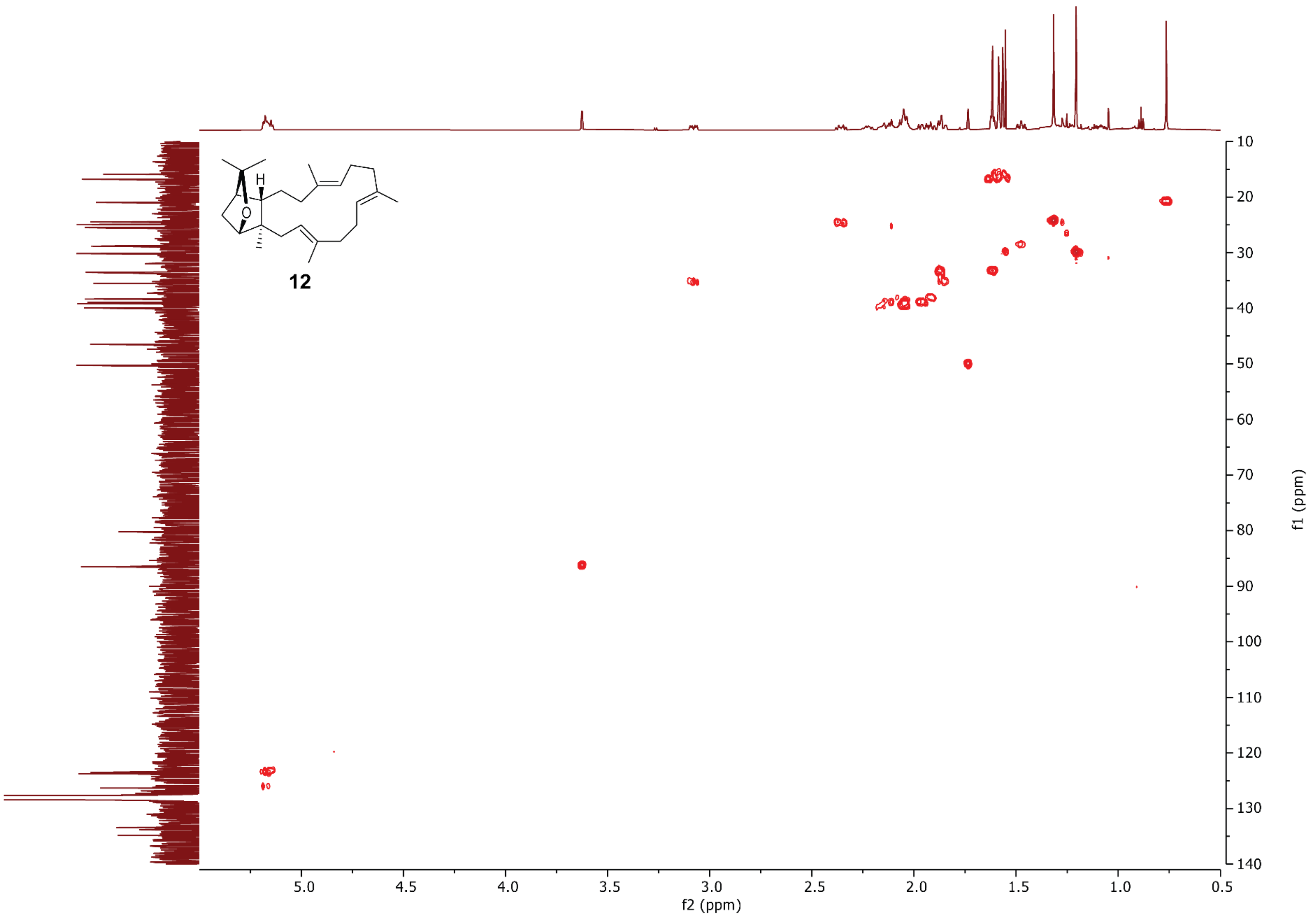

Figure S125. HMQC spectrum of **12** (C_6D_6).

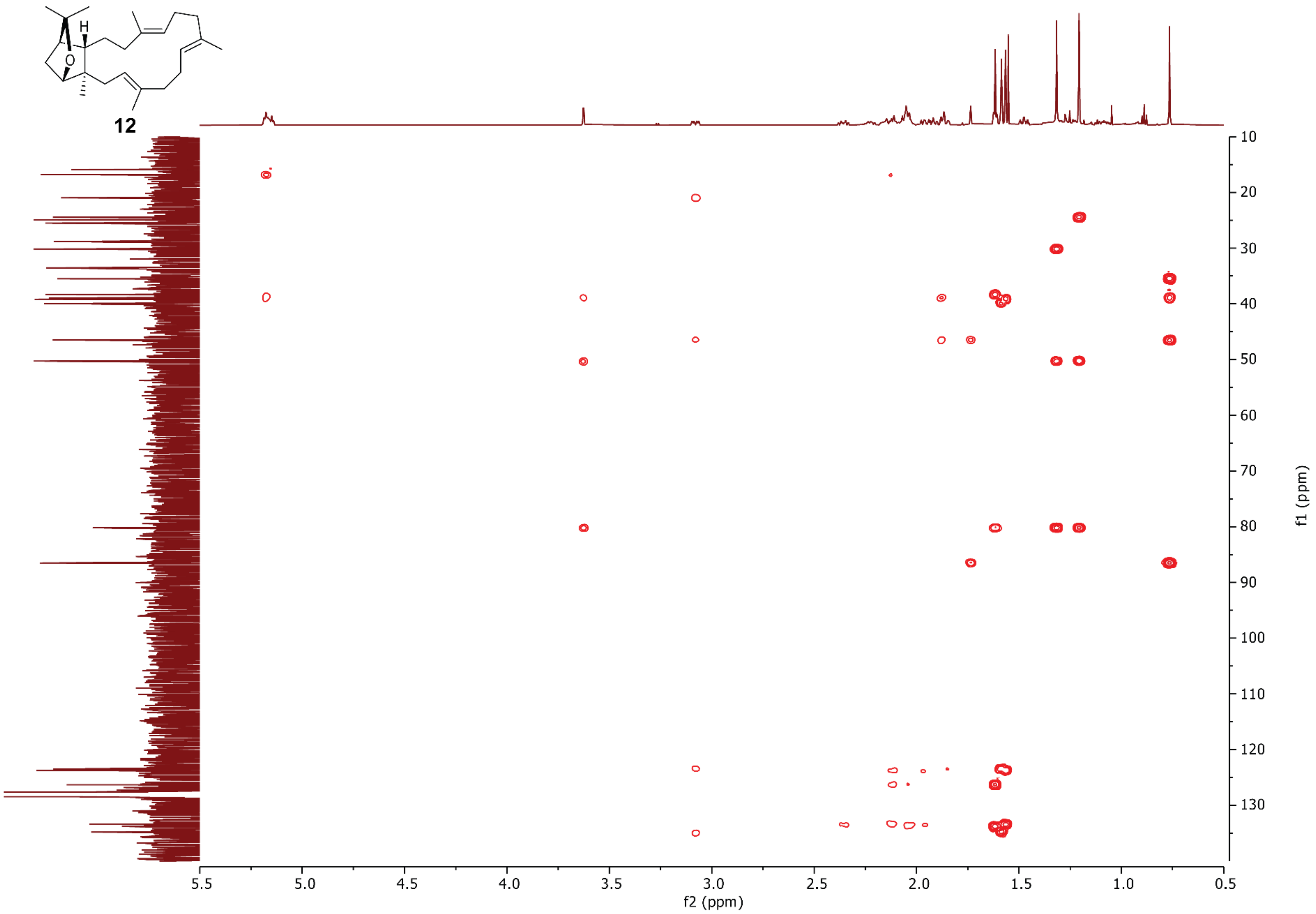

Figure S126. HMBC spectrum of **12** (C_6D_6).

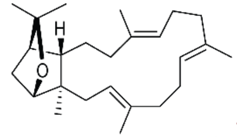

12

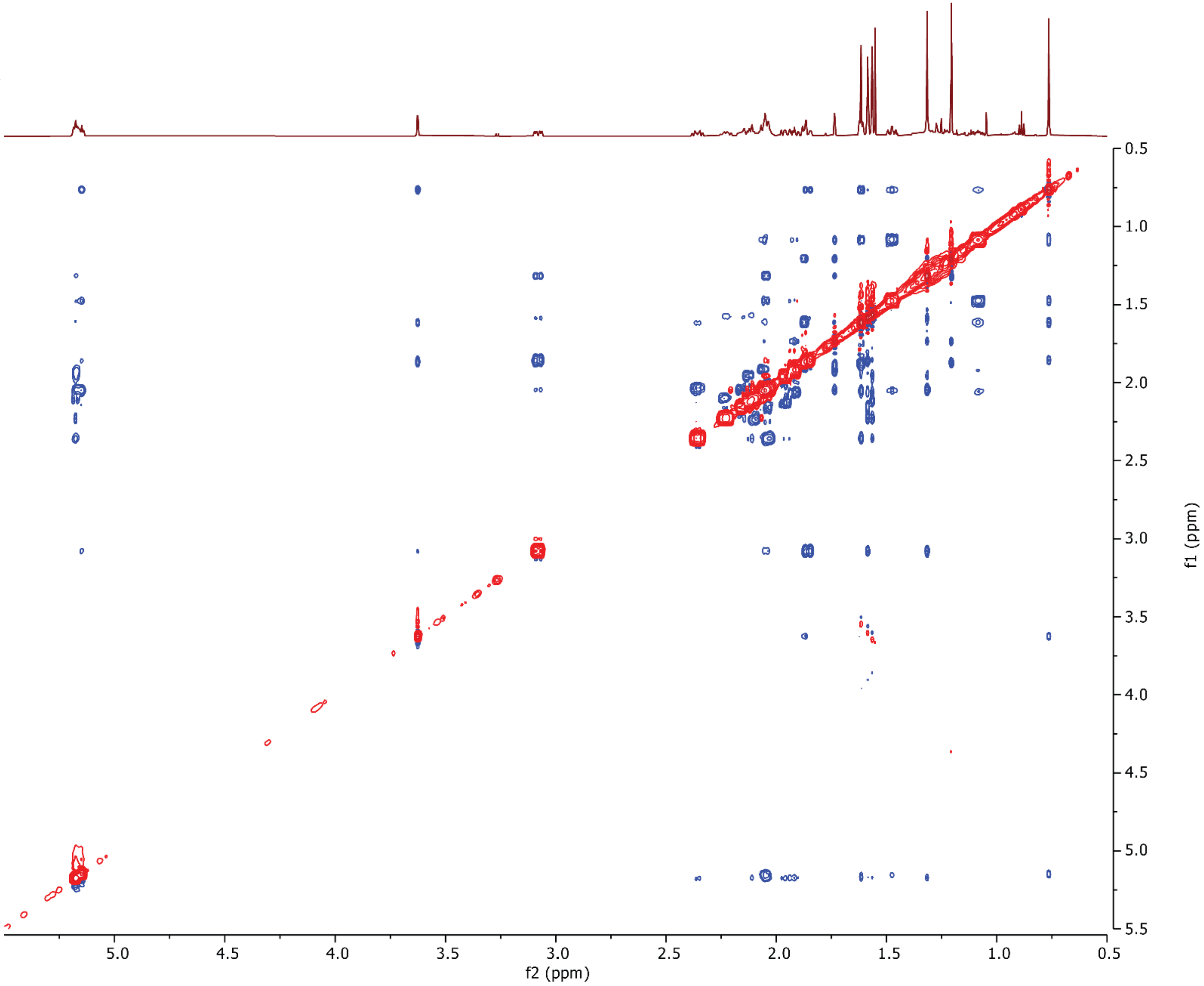

Figure S127. NOESY spectrum of **12** (700 MHz, C_6D_6).

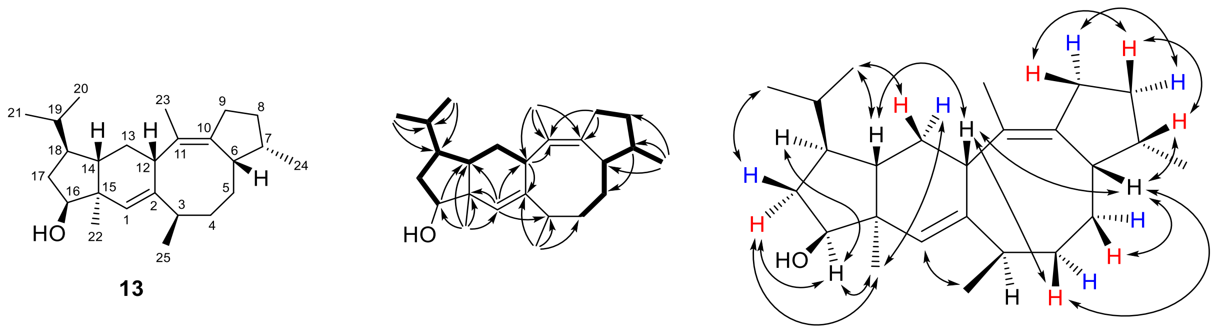

Figure S128. Structure elucidation of **13**. Bold: ^{1}H,^{1}H-COSY correlations, single-headed arrows: key HMBC correlations, and double headed arrows: NOESY correlations.

Table S12. NMR data of 16-*epi*-aspergilol C (**13**) in C_6D_6 recorded at 298 K.

$C^{[a]}$		$\delta_C^{[b]}$	$\delta_H^{[a,b]}$
1	CH	124.0	5.61 (t, $^4J_{H,H}$ = 2.0, 1H)
2	C_q	148.7	–
3	CH	35.5	–
4	CH_2	38.7	1.37 (m, 1H, H_β)<br>1.46 (m, 1H, H_α)
5	CH_2	28.5	0.97 (m, 1H, H_α)<br>1.53 (m, 1H, H_β)
6	CH	47.1	2.57 (m, 1H)
7	CH	39.4	1.94 (m, $1H_\beta$)
8	CH_2	31.6	1.34 (m, 1H, H_α)<br>1.67 (m, 1H, H_β)
9	CH_2	29.9	2.22 (m, 1H, H_β)<br>2.30 (m, 1H, H_α)
10	C_q	142.7	–
11	C_q	128.0	–
12	CH	46.1	3.66 (t, $^3J_{H,H}$ = 8.6, 1H)
13	CH_2	26.1	1.82 (m, 1H, H_α)<br>1.90 (m, 1H, H_β)
14	CH	43.1	2.12 (m, 1H)
15	C_q	48.3	–
16	CH	77.6	3.71 (d, $^3J_{H,H}$ = 5.6, 1H)
17	CH_2	36.4	1.47 (m, 1H, H_β)<br>2.09 (m, 1H, H_α)
18	CH	45.4	1.55 (s, 1H)
19	CH	30.5	1.77 (m, 1H)
20	CH_3	22.4	0.97 (d, $^3J_{H,H}$ = 7.0, 3H)
21	CH_3	19.2	0.97 (d, $^3J_{H,H}$ = 7.0, 3H)
22	CH_3	21.6	0.80 (s, 3H)
23	CH_3	15.3	1.56 (br, 3H)
24	CH_3	15.8	0.93 (d, $^3J_{H,H}$ = 6.9, 3H)
25	CH_3	21.7	1.04 (d, $^3J_{H,H}$ = 7.0, 3H)

[a] Carbon numbering and colour code for hydrogens as in Figure S127, [b] chemical shifts δ in ppm, multiplicity: s = singlet, d = doublet, t = triplet, m = multiplet, coupling constants J are given in Hertz.

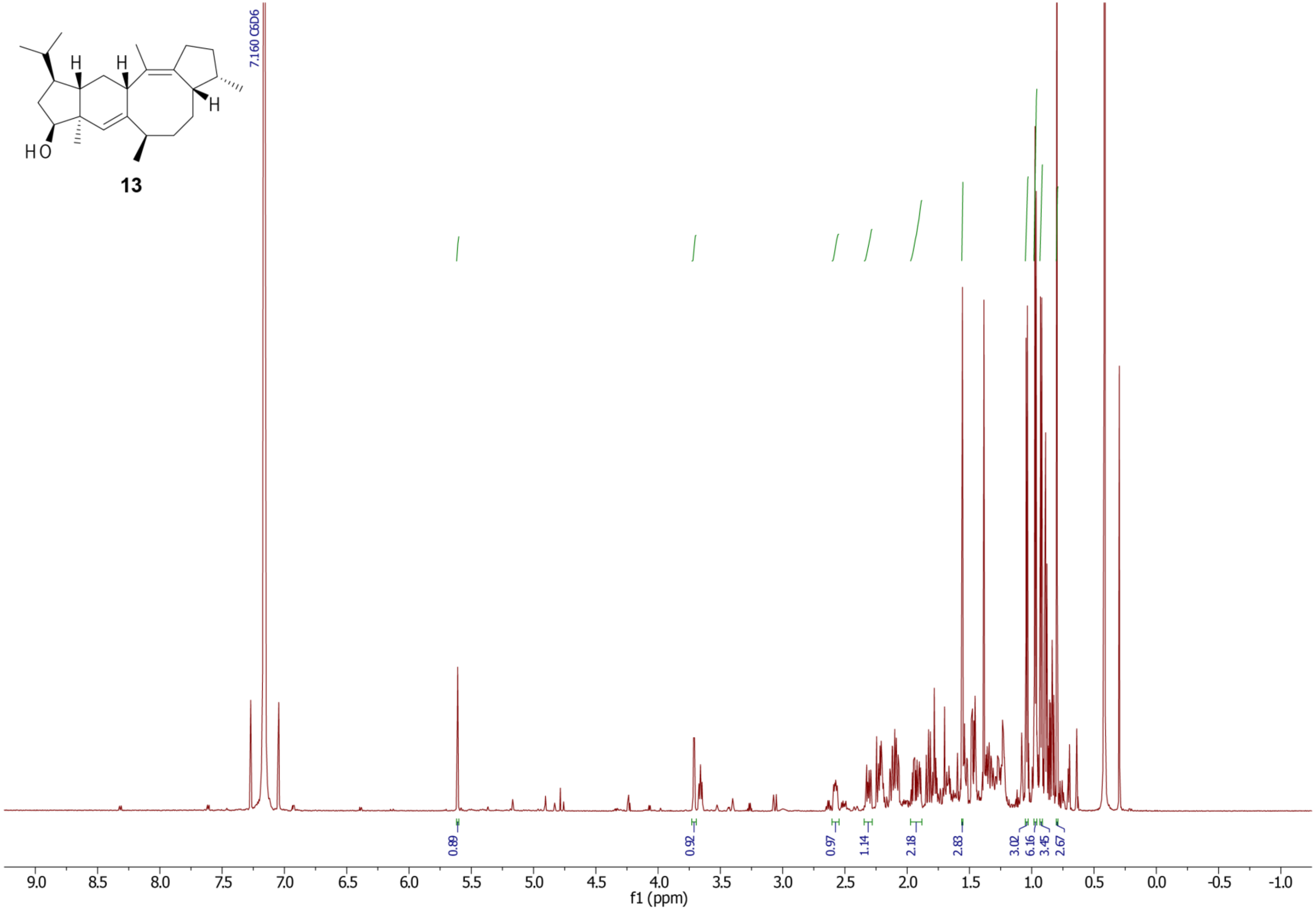

Figure S129. ^{1}H-NMR spectrum of **13** (700 MHz, C_6D_6).

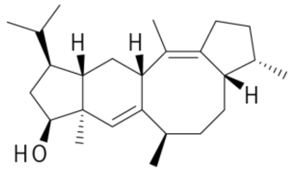

13

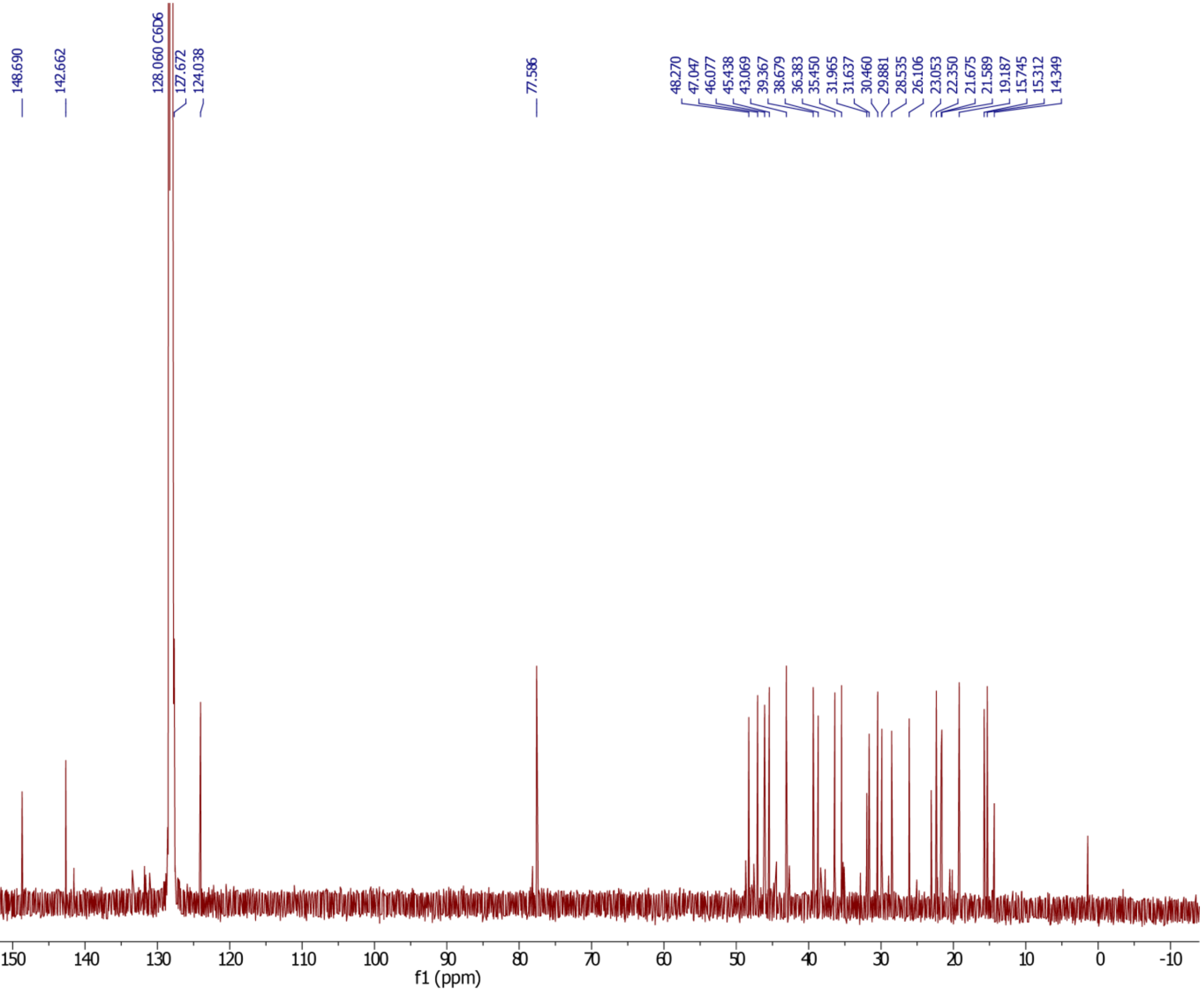

Figure S130. ^{13}C-NMR spectrum of **13** (176 MHz, C_6D_6).

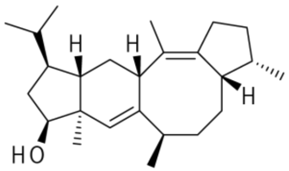

13

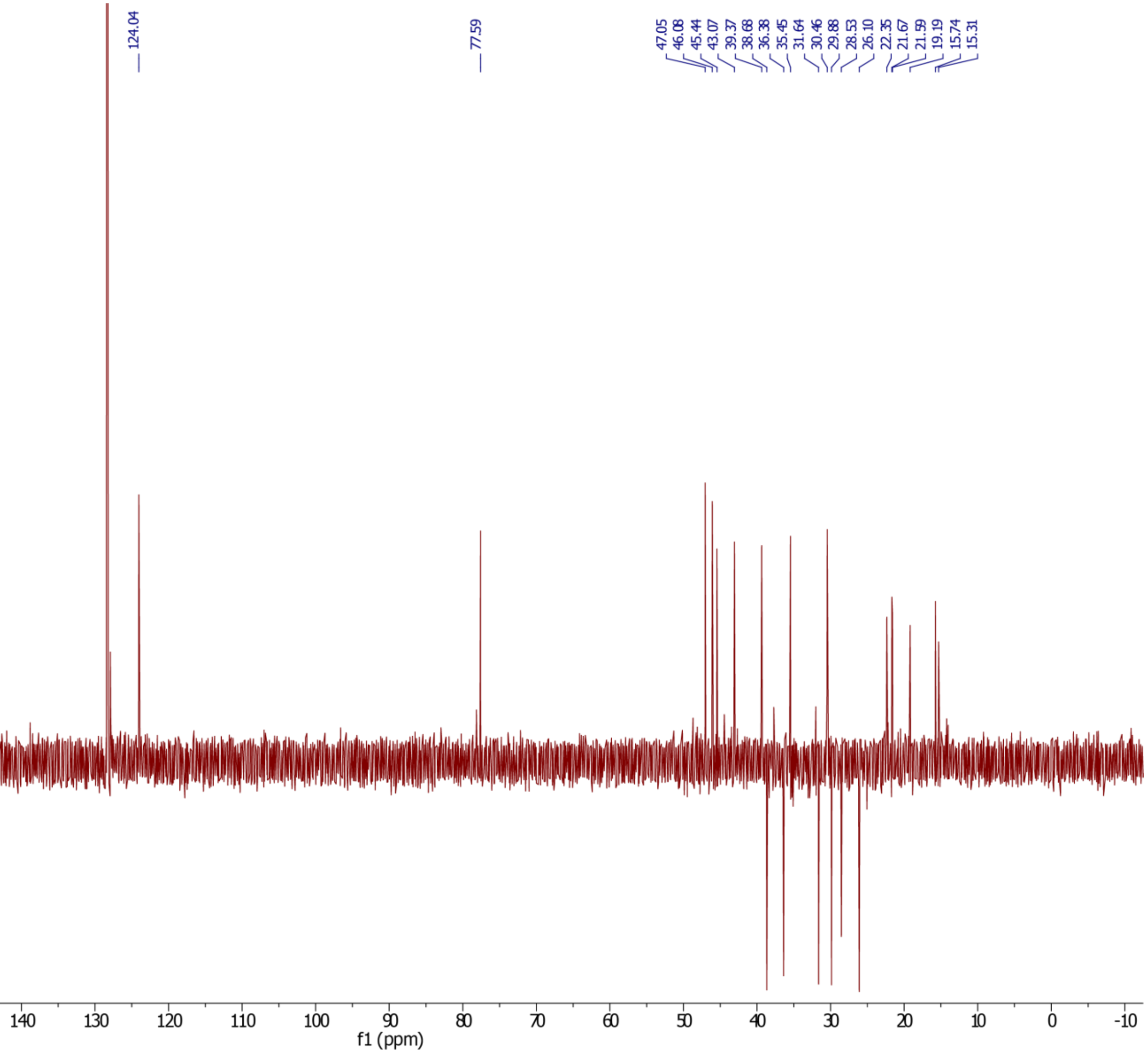

Figure S131. DEPT spectrum of **13** (176 MHz, C_6D_6).

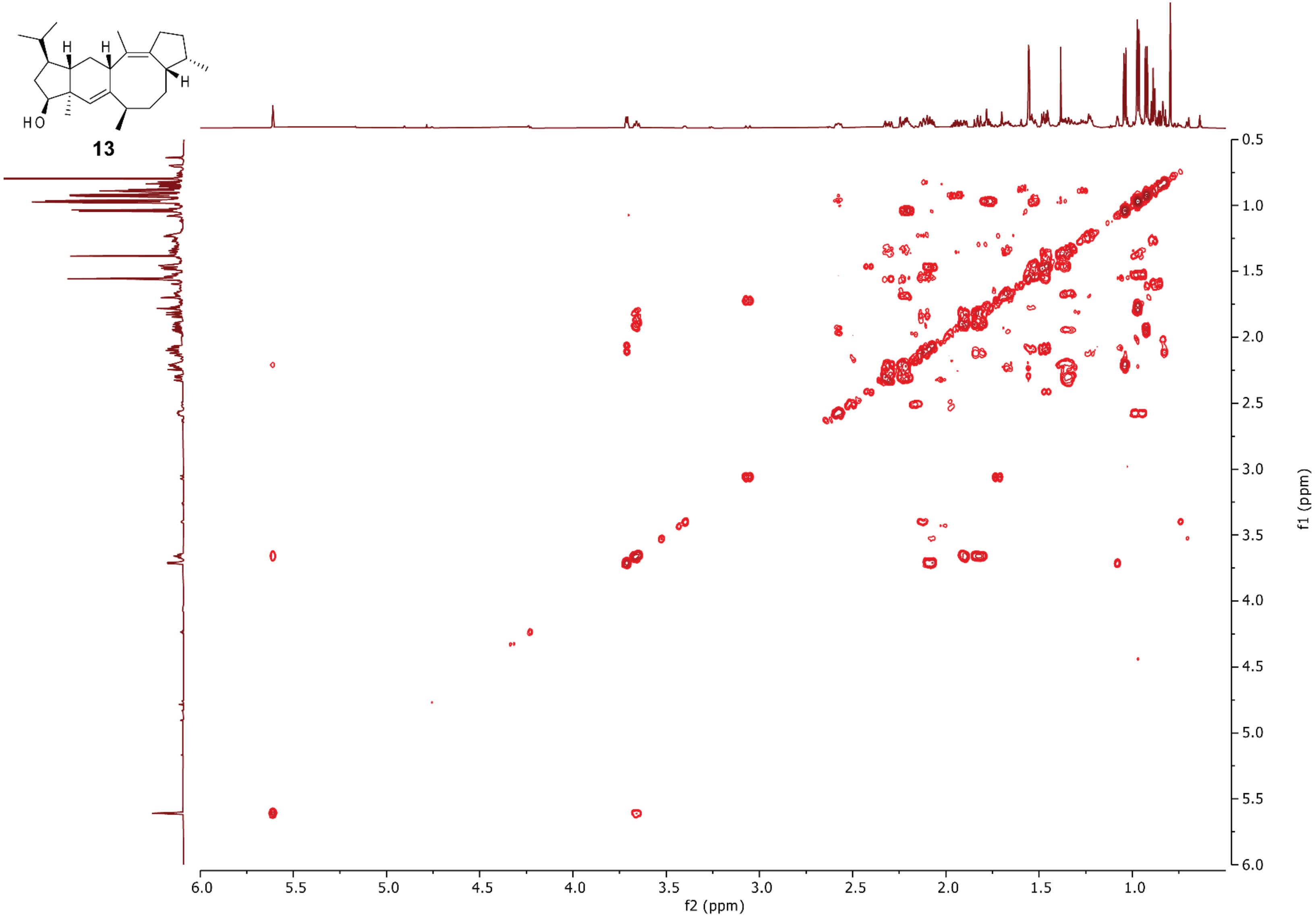

Figure S132. ^{1}H,^{1}H-COSY spectrum of **13** (700 MHz, C_6D_6).

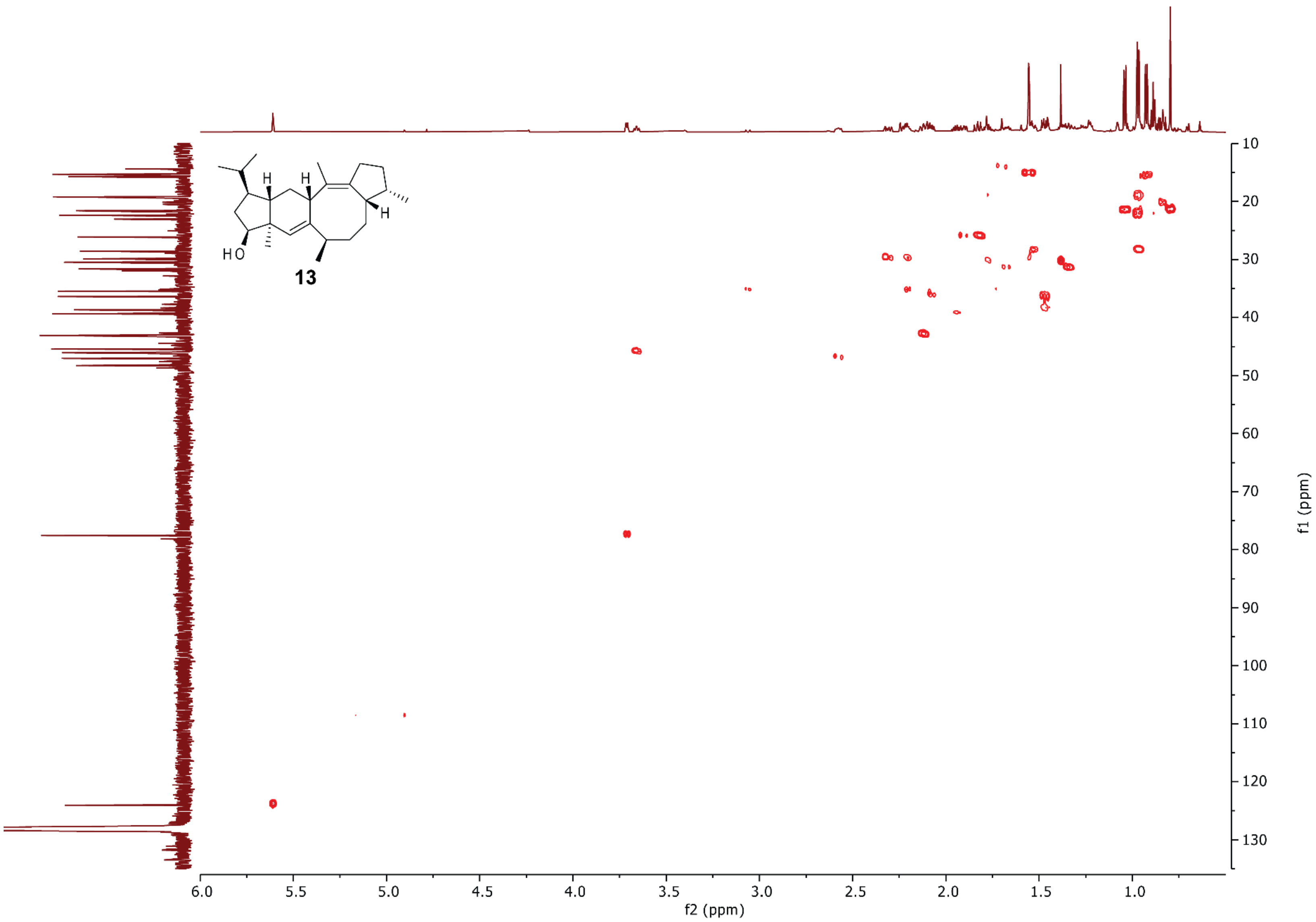

Figure S133. HMQC spectrum of **13** (C_6D_6).

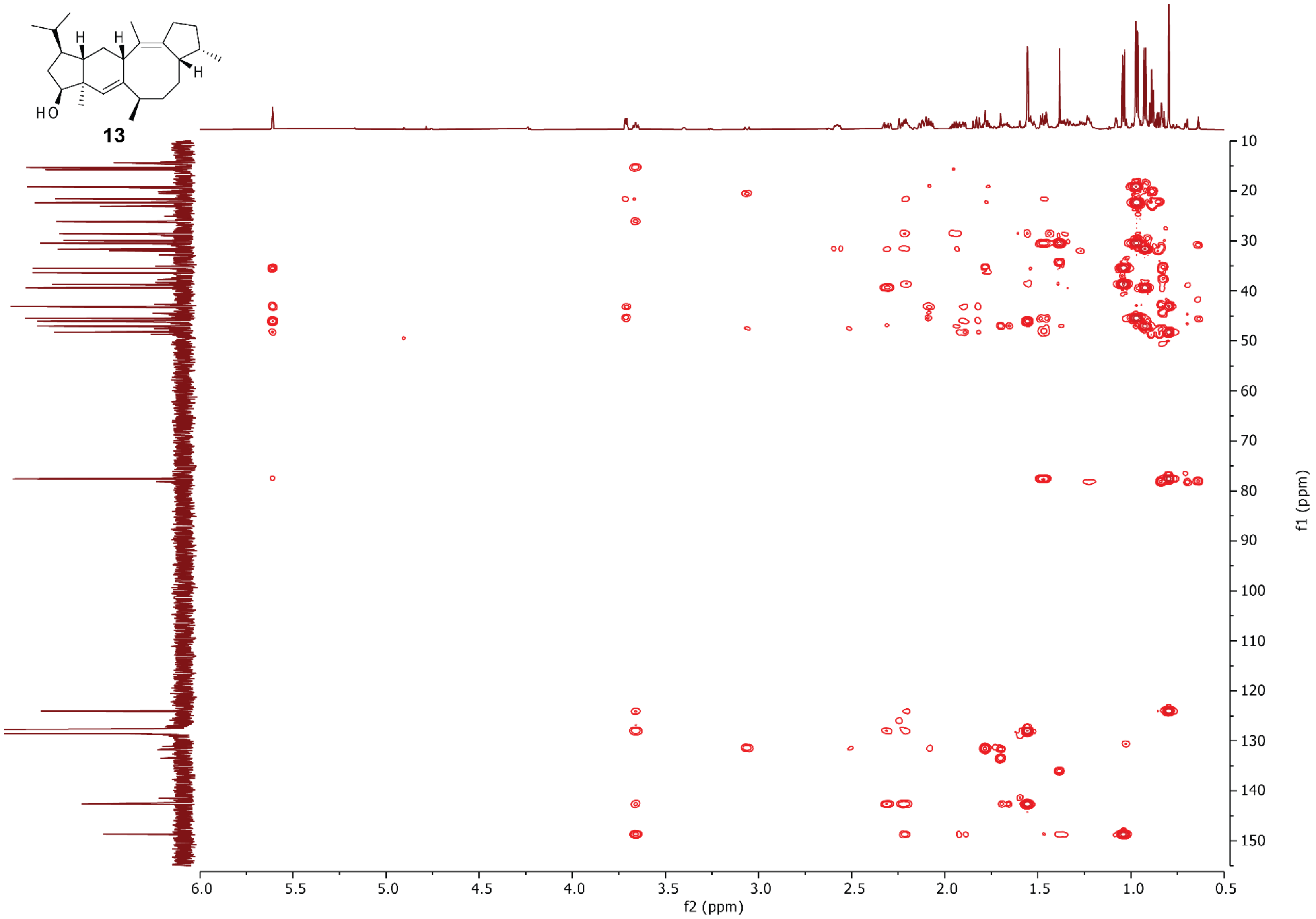

Figure S134. HMBC spectrum of **13** (C_6D_6).

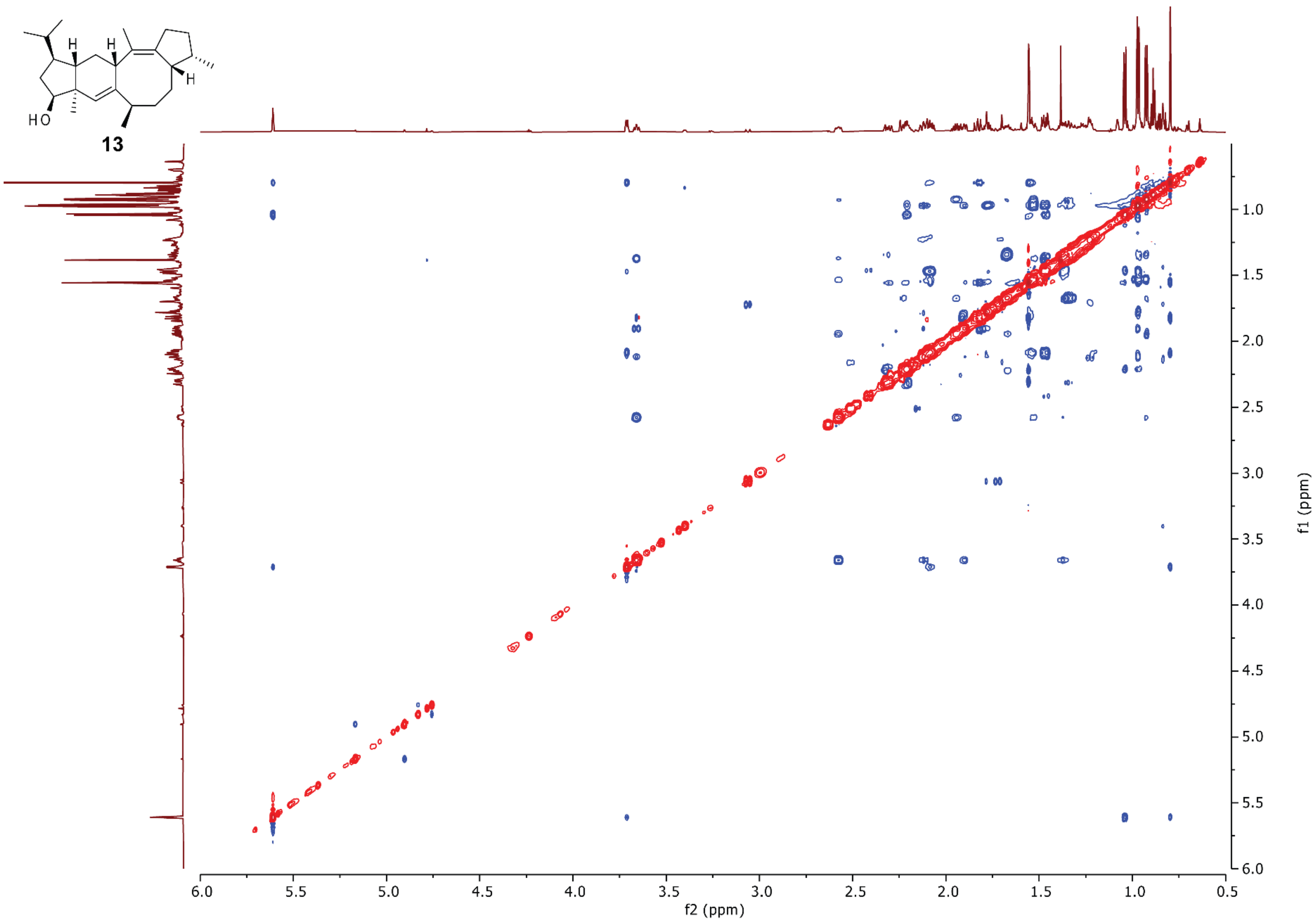

13

Figure S135. NOESY spectrum of **13** (700 MHz, C_6D_6).

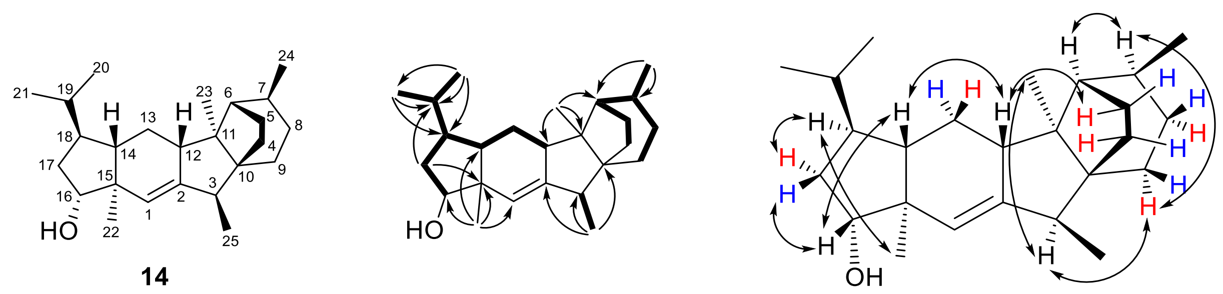

Figure S136. Structure elucidation of **14**. Bold: ^{1}H,^{1}H-COSY correlations, single-headed arrows: key HMBC correlations, and double headed arrows: NOESY correlations.

Table S13. NMR data of 16-hydroxycalidoustene (**14**) in C_6D_6 recorded at 298 K.

C[a]		δ_C[b]	δ_H[a,b]
1	CH	126.9	5.79 (dd, $^4J_{H,H}$ = 0.7, 0.7, 1H)
2	C_q	148.4	–
3	CH	42.4	2.04 (m, 1H)
4	CH_2	30.6	1.08 (m, 1H, H)<br>1.62 (m, 1H, H)
5	CH_2	20.6	1.40 (m, 1H, H)<br>1.54 (m, 1H, H)
6	CH	48.0	1.67 (m, 1H)
7	CH	29.9	1.89 (m, 1H)
8	CH_2	27.5	0.96 (m, 1H, H_β)<br>1.38 (m, 1H, H_α)
9	CH_2	30.9	1.26 (m, 1H, H_β)<br>1.31 (m, 1H, H_α)
10	C_q	51.9	–
11	C_q	52.6	–
12	CH	50.5	2.48 (dddd, $^3J_{H,H}$ = 11.2, 5.5, $^4J_{H,H}$ = 2.6, 2.6, 1H)
13	CH_2	22.1	1.31 (m, 1H, H_α)<br>1.57 (m, 1H, H_β)
14	CH	47.1	1.56 (m, 1H)
15	C_q	47.7	–
16	CH	77.4	3.72 (t, $J_{H,H}$ = 8.3, 1H)
17	CH_2	34.6	1.45 (m, 1H, H_α)<br>1.75 (m, 1H, H_β)
18	CH	44.0	1.68 (m, 1H)
19	CH	29.6	1.64 (m, 1H)
20	CH_3	22.3	0.90 (d, $J_{H,H}$ = 6.7, 3H)
21	CH_3	18.2	0.80 (d, $J_{H,H}$ = 6.7, 3H)
22	CH_3	17.5	0.94 (s, 3H)
23	CH_2	13.3	0.78 (s, 3H)
24	CH_3	20.6	0.82 (d, $J_{H,H}$ = 6.6, 3H)
25	CH_3	14.4	1.05 (d, $J_{H,H}$ = 7.1, 3H)

[a] Carbon numbering and colour code for hydrogens as in Figure S135, [b] chemical shifts δ in ppm, multiplicity: s = singlet, d = doublet, t = triplet, m = multiplet, coupling constants J are given in Hertz.

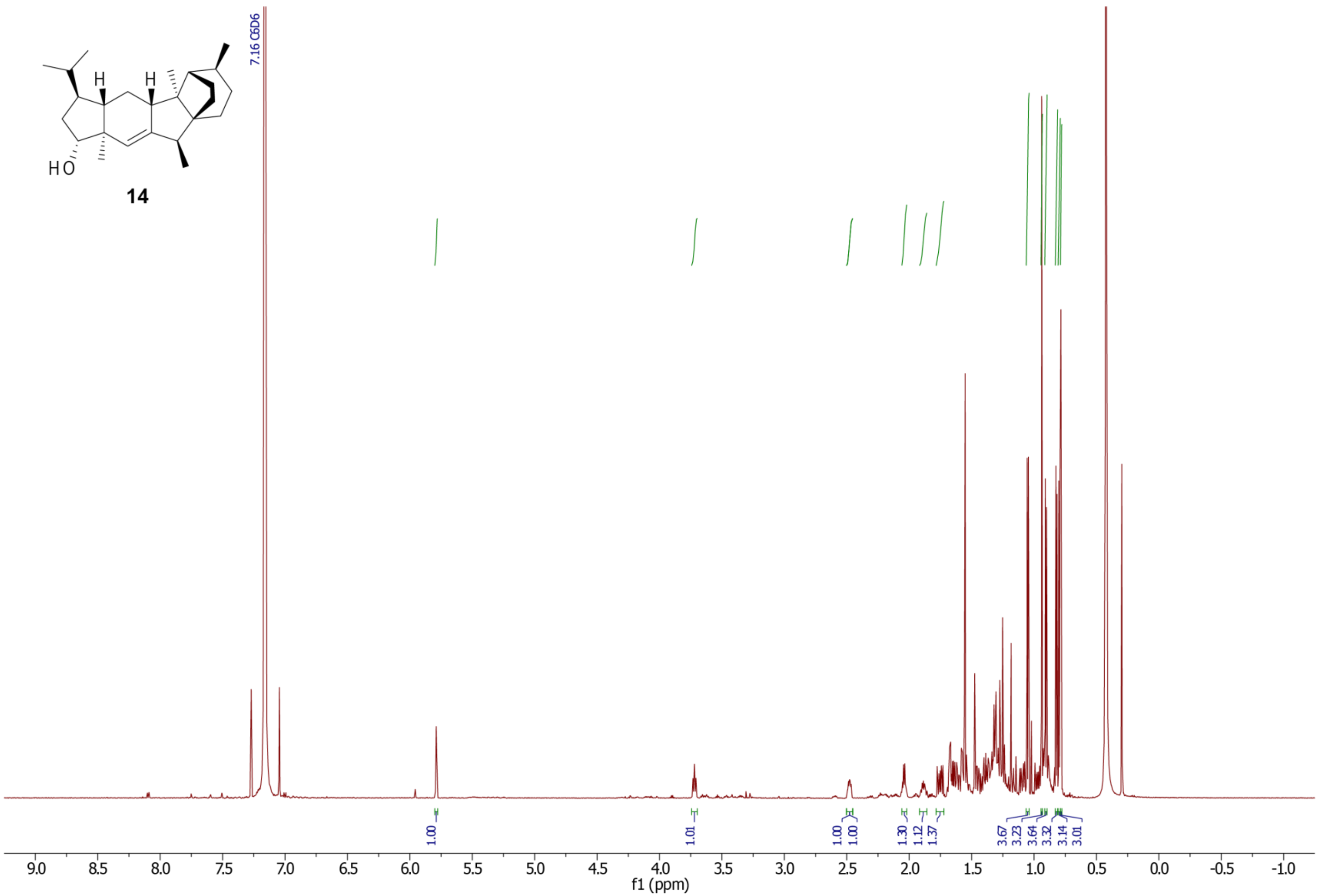

Figure S137. ^{1}H-NMR spectrum of **14** (700 MHz, C_6D_6).

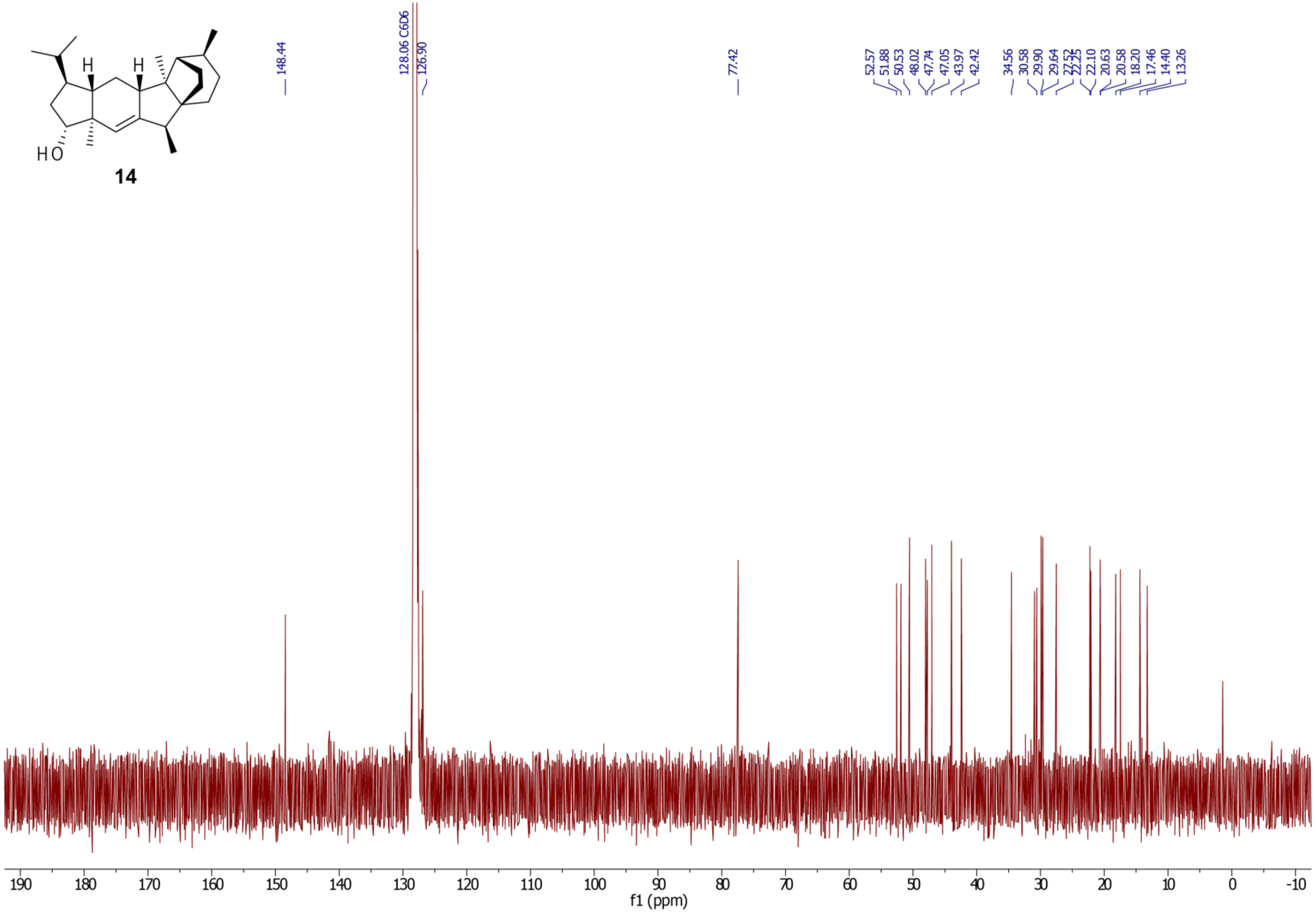

Figure S138. ^{13}C-NMR spectrum of **14** (176 MHz, C_6D_6).

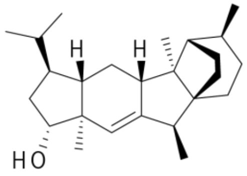

14

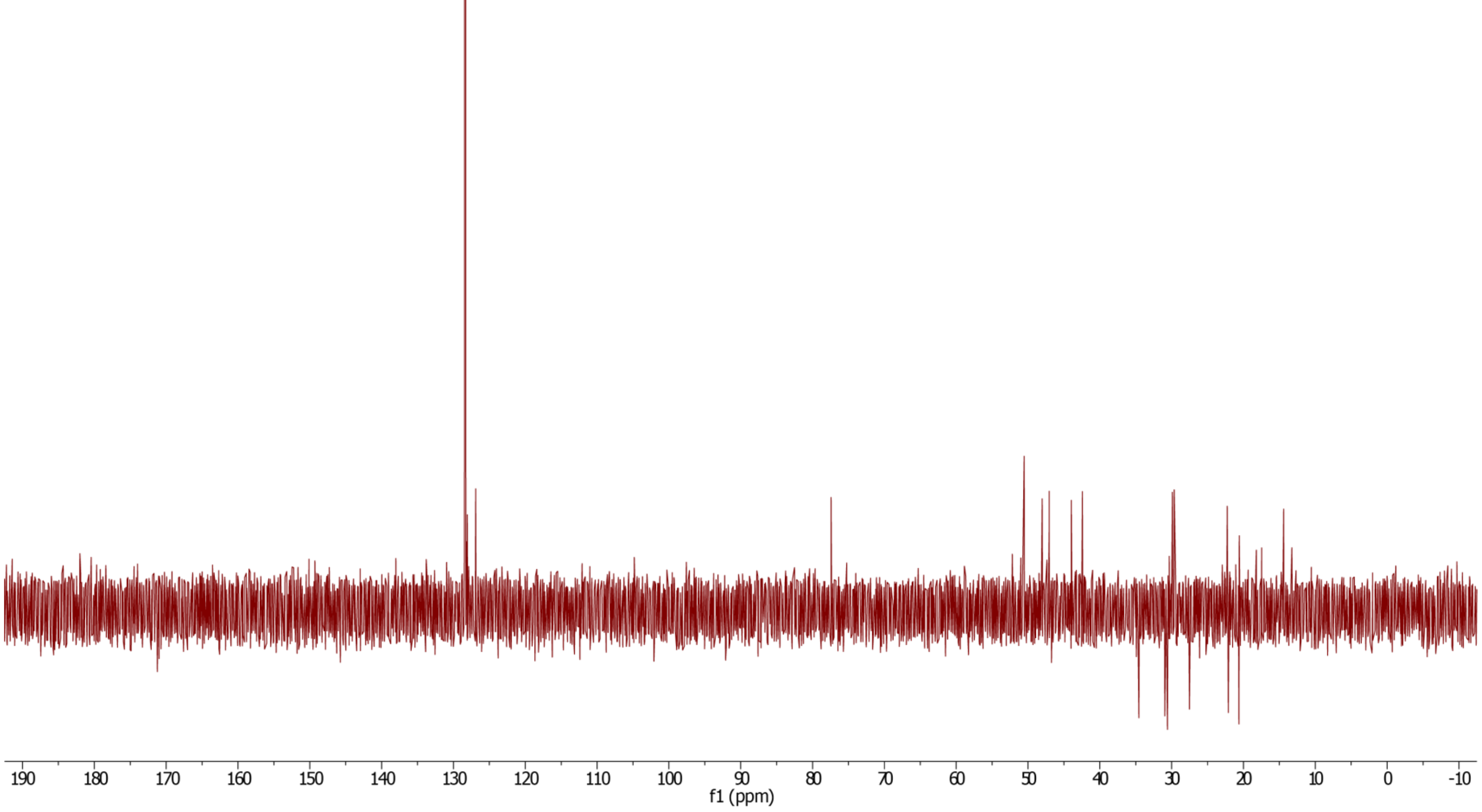

Figure S139. DEPT spectrum of **14** (176 MHz, C_6D_6).

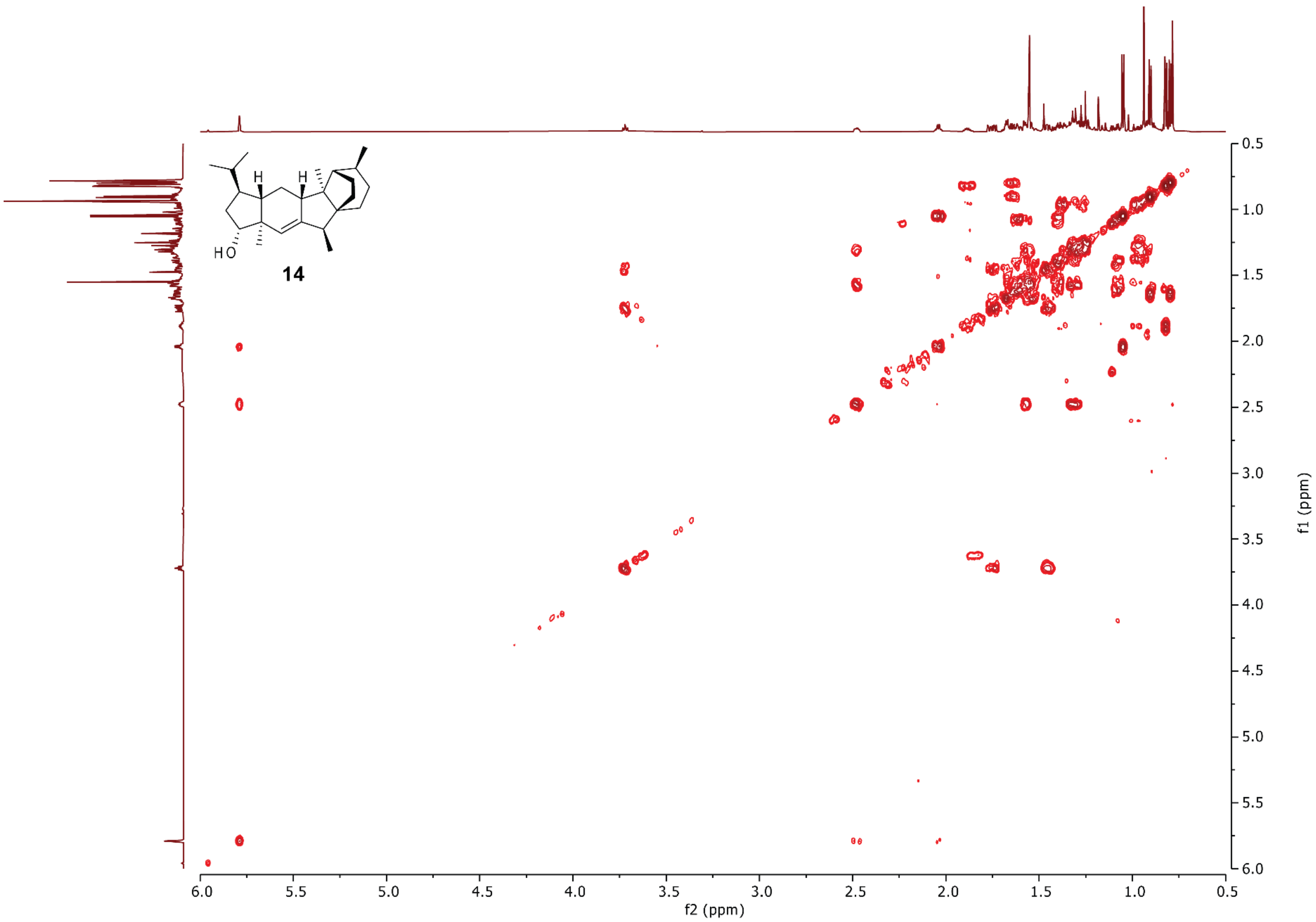

Figure S140. $^{1}H,^{1}H$-COSY spectrum of **14** (700 MHz, C_6D_6).

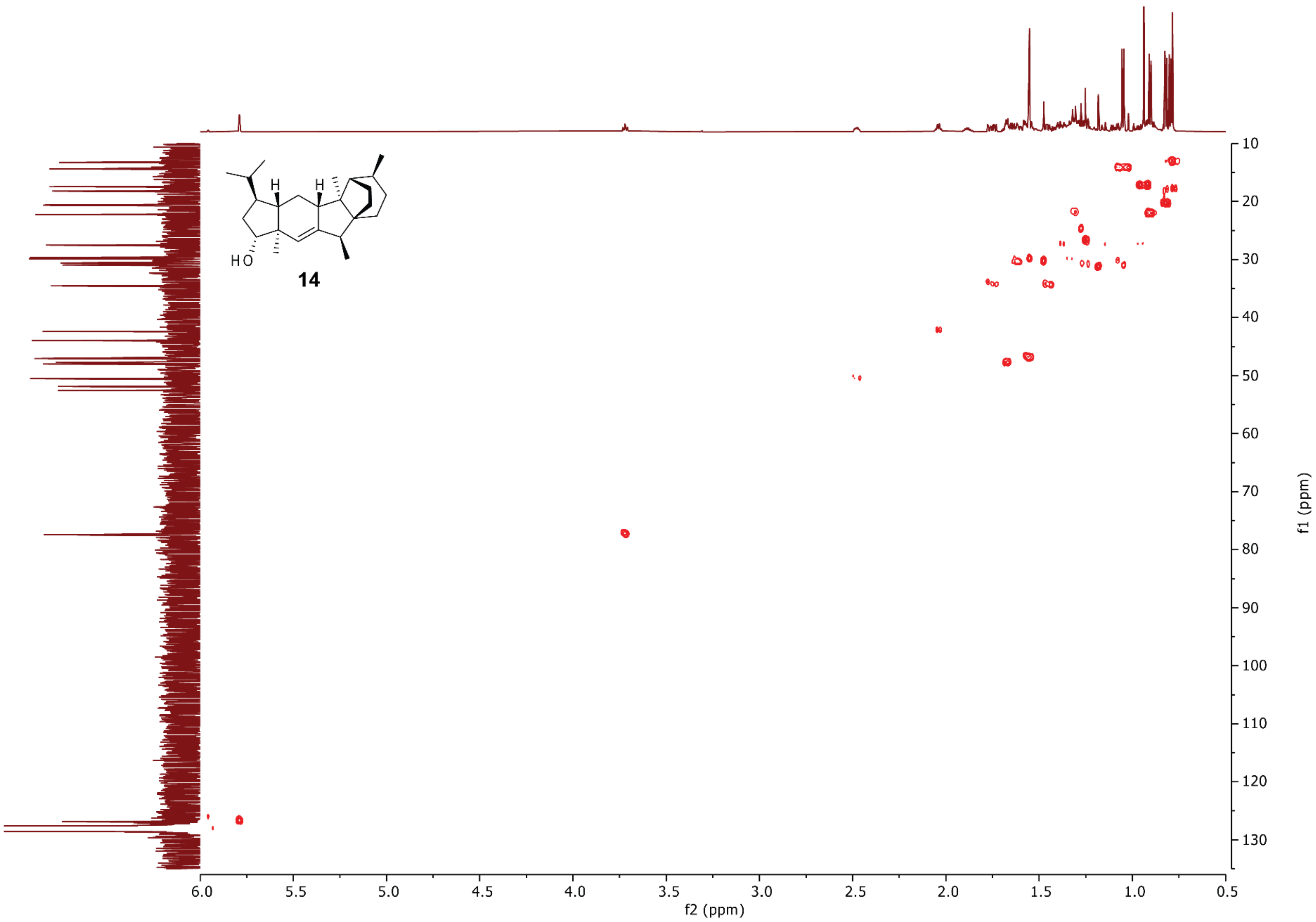

Figure S141. HMQC spectrum of **14** (C_6D_6).

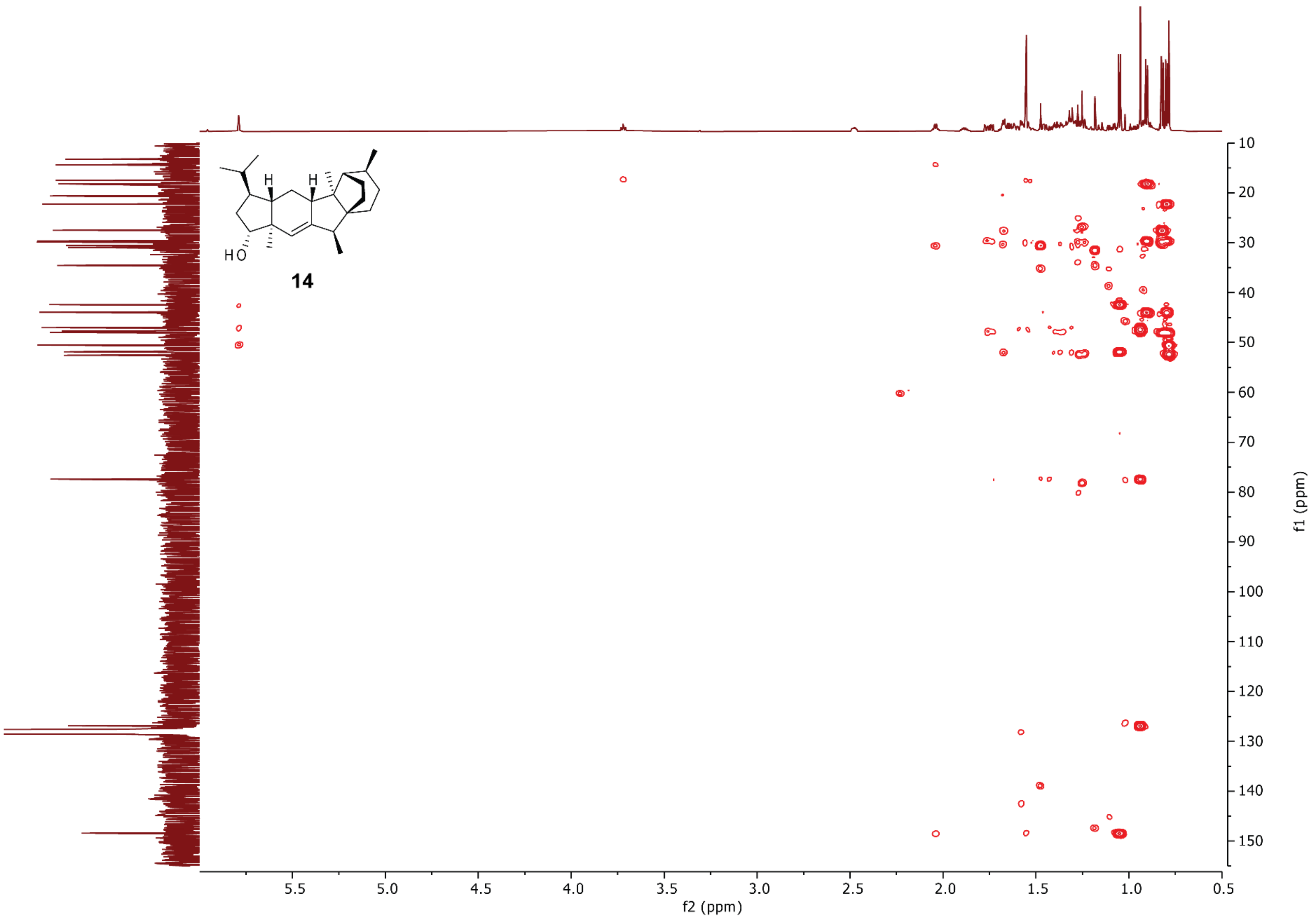

Figure S142. HMBC spectrum of **14** (C_6D_6).

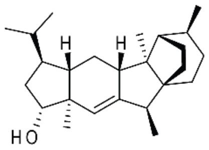

14

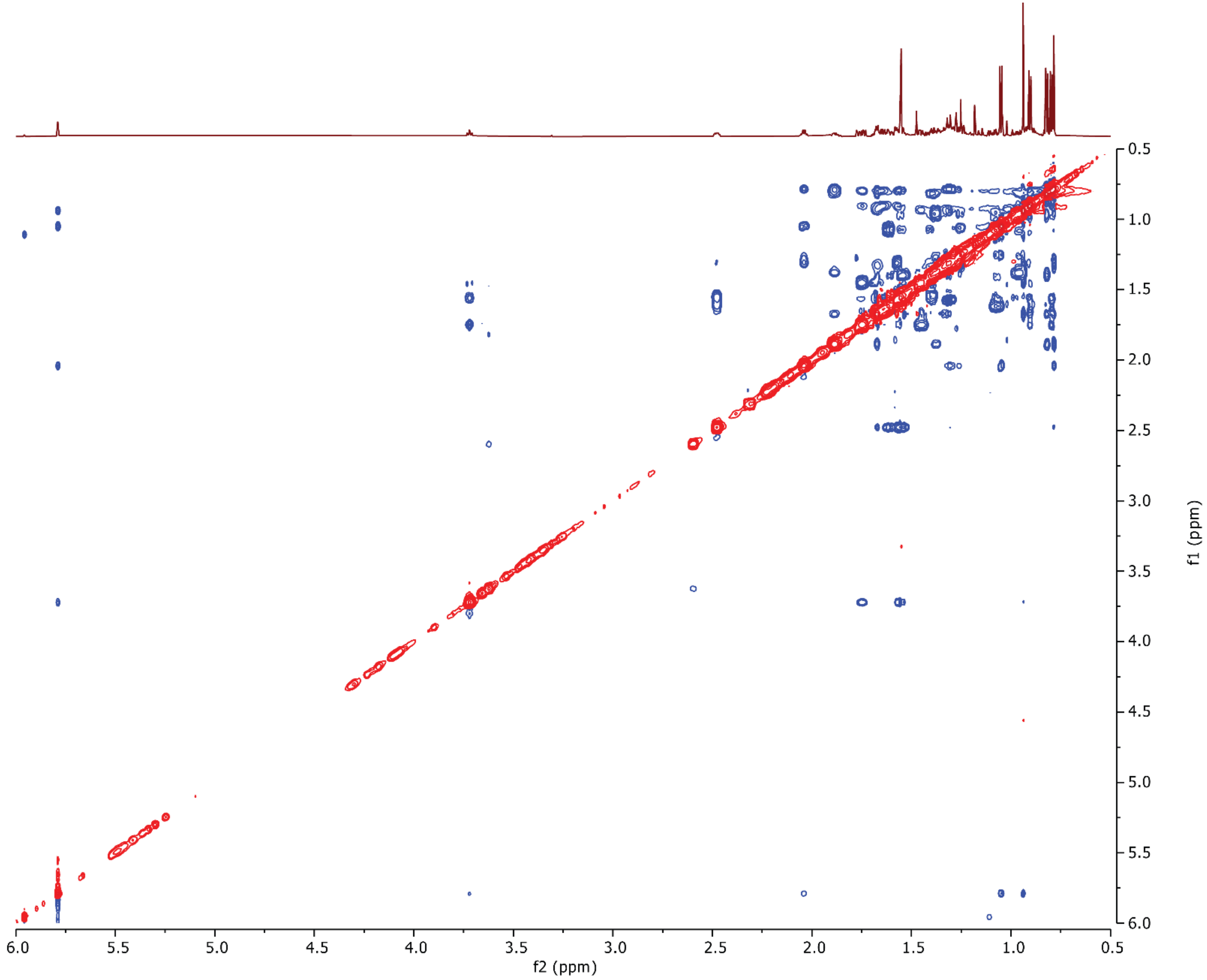

Figure S143. NOESY spectrum of **14** (700 MHz, C_6D_6).

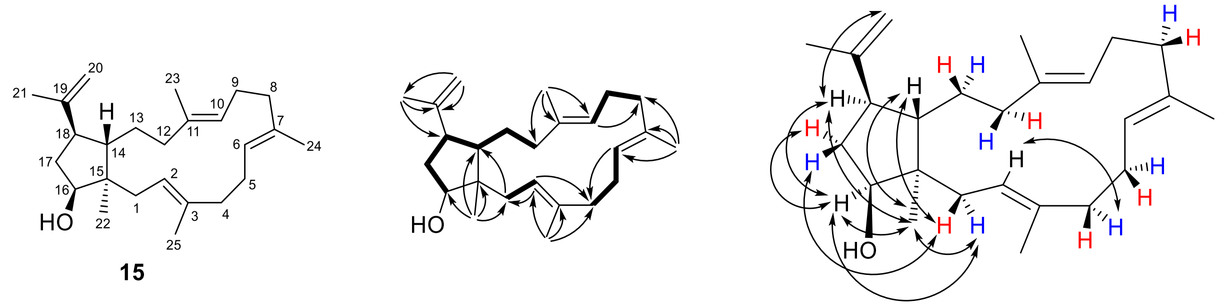

15

Figure S144. Structure elucidation of **15**. Bold: $^{1}H,^{1}H$-COSY correlations, single-headed arrows: key HMBC correlations, and double headed arrows: NOESY correlations.

Table S14. NMR data of 16-hydroxycalidoustatetraene (**15**) in C_6D_6 recorded at 298 K.

C[a]		δ_C[b]	δ_H[a,b]
1	CH_2	35.7	2.05 (m, 1H, H_α)<br>2.40 (dd, $^2J_{H,H}$ = 15.4, $^3J_{H,H}$ = 8.3, 1H, H_β)
2	CH	122.6	5.44 (m, 1H)
3	C_q	135.4	–
4	CH_2	39.5	2.09 (m, 2H)
5	CH_2	25.3	2.12 (m, 1H, H_α)<br>2.20 (m, 1H, H_β)
6	CH	125.5	5.15 (m, 1H)
7	C_q	133.8	–
8	CH_2	39.6	2.09 (m, 2H)
9	CH_2	24.9	2.12 (m, 1H)<br>2.20 (m, 1H)
10	CH	124.4	5.19 (m, 1H)
11	C_q	134.8	–
12	CH_2	37.6	2.05 (m, 1H, H_α)<br>2.14 (m, 1H, H_β)
13	CH_2	29.4	1.34 (m, 1H, H_α)<br>1.65 (m, 1H, H_β)
14	C_q	43.7	–
15	C_q	47.3	–
16	CH	82.6	3.53 (t, $^3J_{H,H}$ = 5.8, 1H)
17	CH_2	39.5	1.47 (ddd, $^2J_{H,H}$ = 13.3, $^3J_{H,H}$ = 8.6, 5.9, 1H, H_α)<br>2.01 (ddd, $^2J_{H,H}$ = 13.3, $^3J_{H,H}$ = 8.8, 5.9, 1H, H_β)
18	CH	51.9	2.19 (s, 1H)
19	C_q	148.9	–
20	CH_2	111.0	4.78 (dq, $^2J_{H,H}$ = 2.7, $^4J_{H,H}$ = 1.3, 1H)<br>4.89 (m, 1H)
21	CH_3	19.2	1.77 (dd, $^4J_{H,H}$ = 1.2, 0.7, 3H)
22	CH_3	21.4	0.85 (s, 3H)
23	CH_3	16.7	1.58 (s, 3H)
24	CH_3	16.0	1.54 (s, 3H)
25	CH_3	16.3	1.57 (s, 3H)

[a] Carbon numbering and colour code for hydrogens as in Figure S143, [b] chemical shifts δ in ppm, multiplicity: s = singlet, d = doublet, t = triplet, q = quartet, m = multiplet, coupling constants J are given in Hertz.

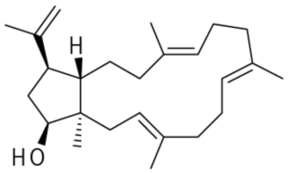

15

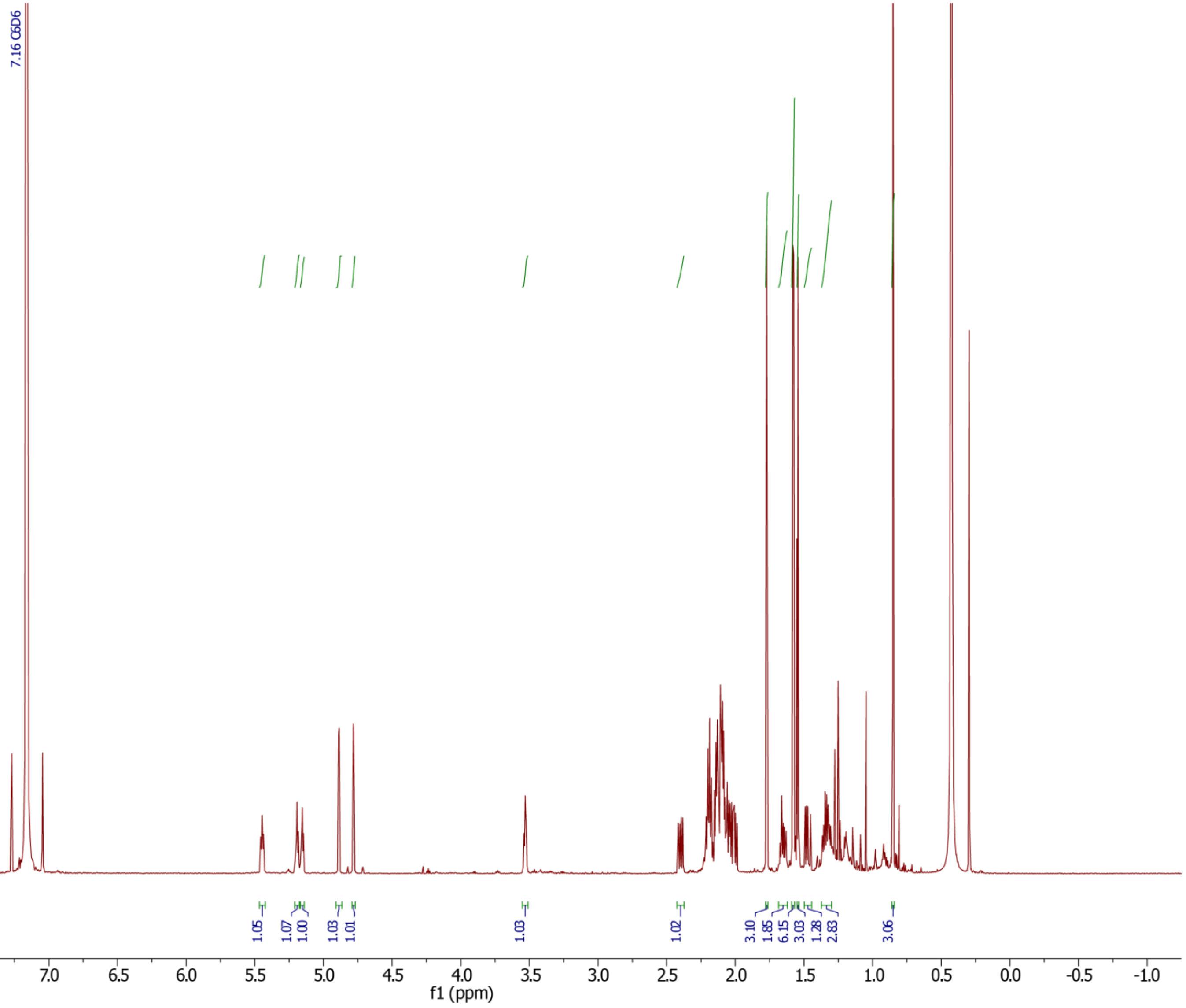

Figure S145. ^{1}H-NMR spectrum of **15** (700 MHz, C_6D_6).

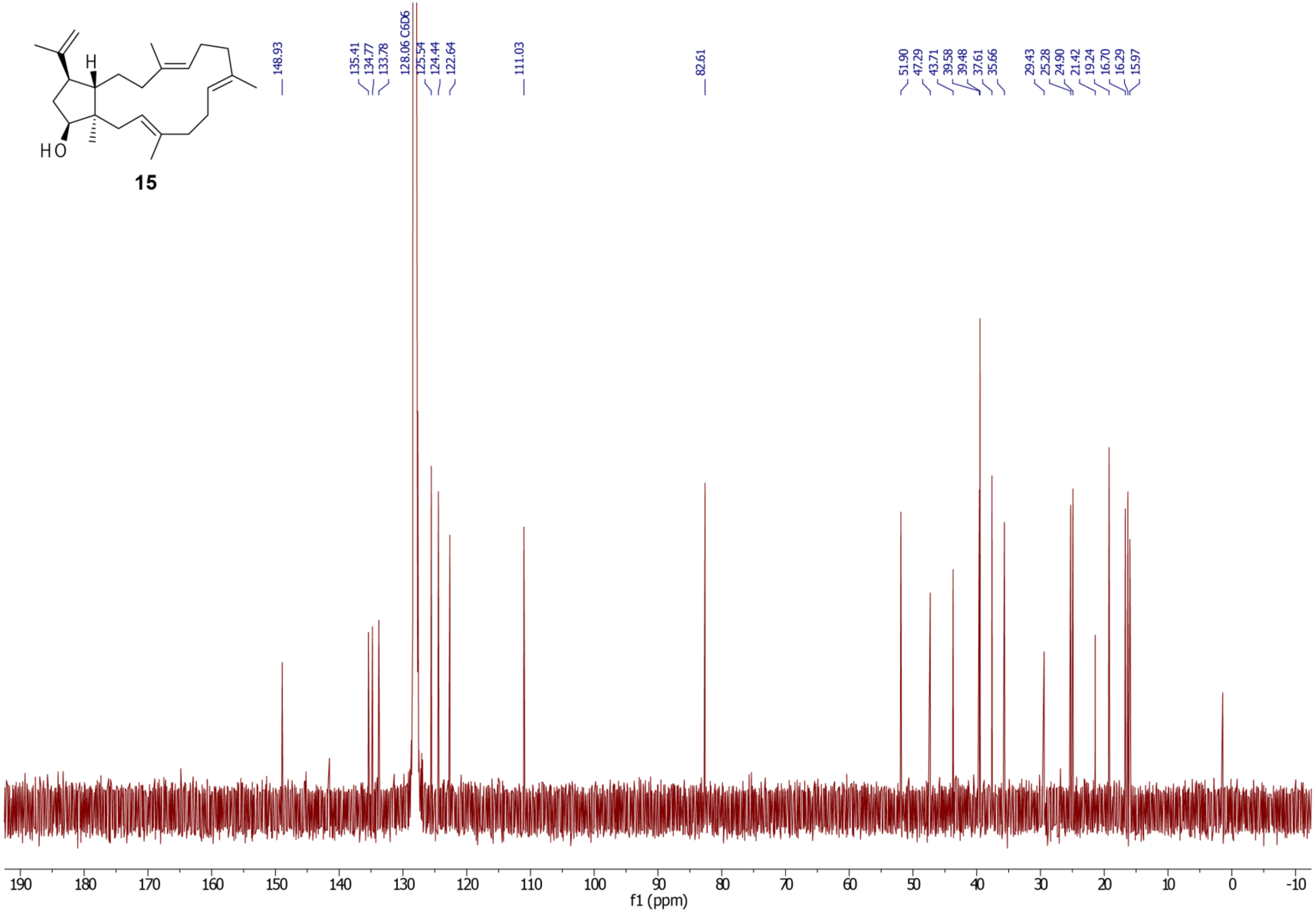

Figure S146. ^{13}C-NMR spectrum of **15** (176 MHz, C_6D_6).

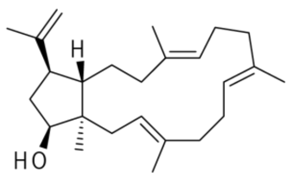

15

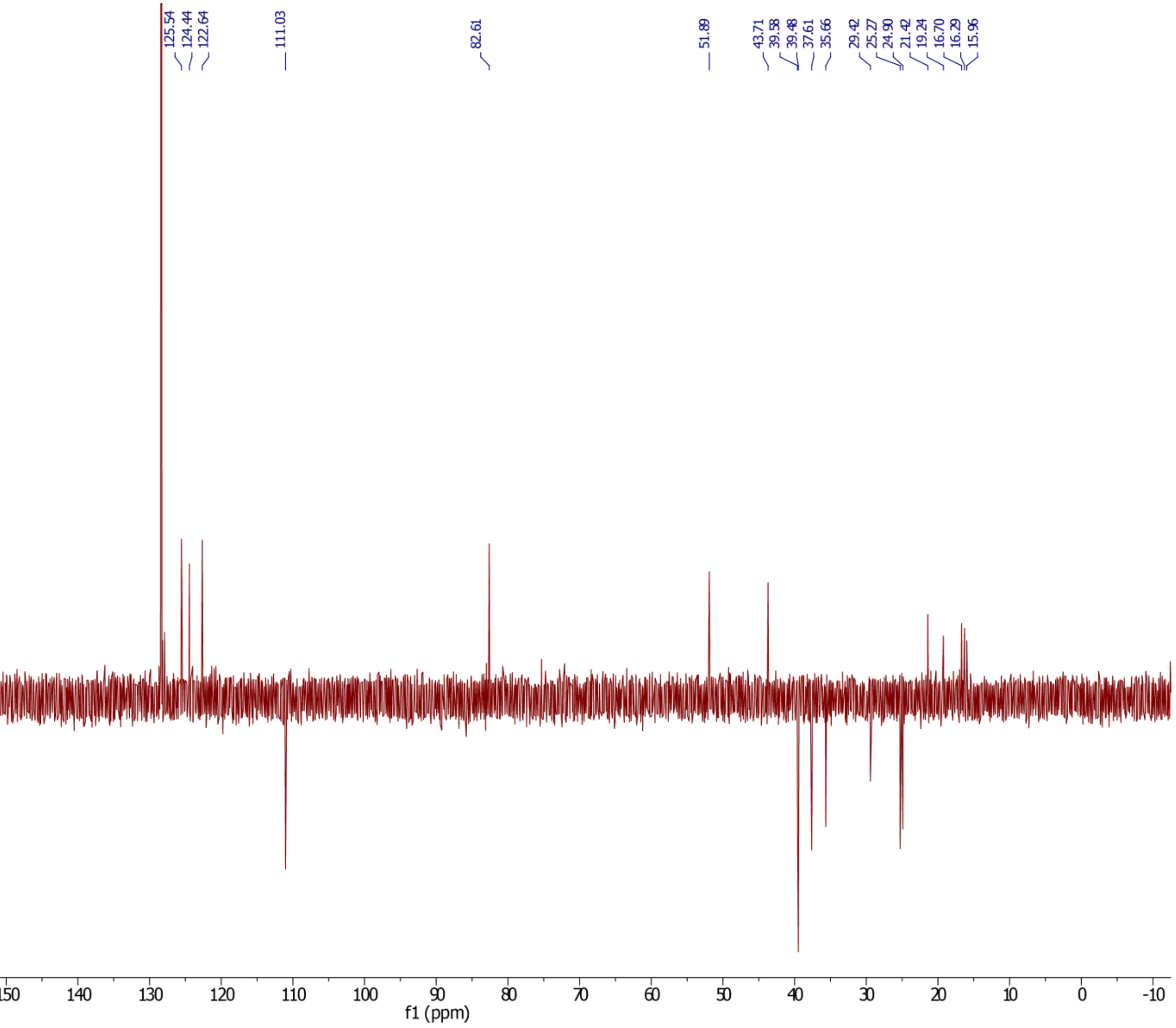

Figure S147. DEPT spectrum of **15** (176 MHz, C_6D_6).

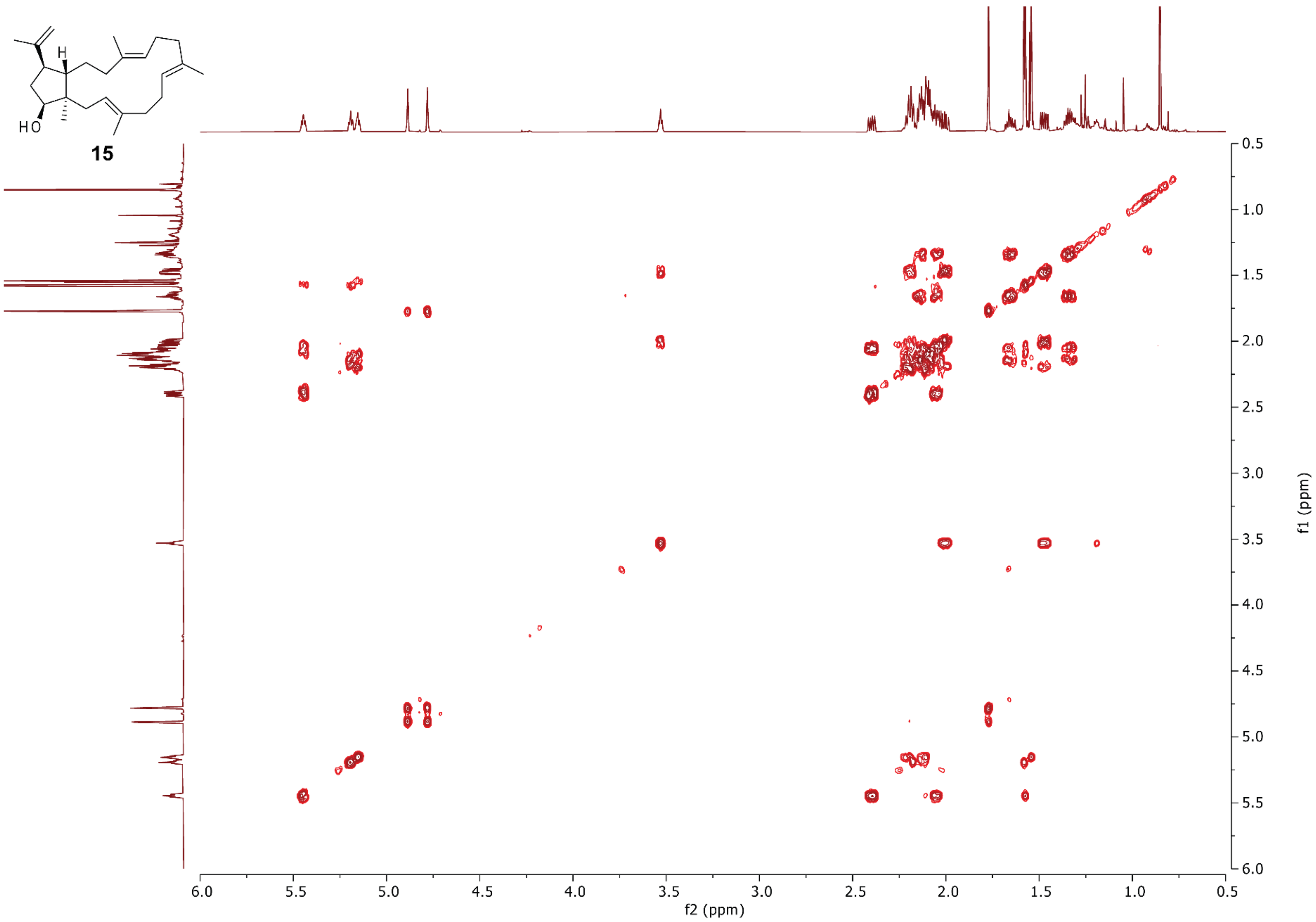

Figure S148. $^{1}H,^{1}H$-COSY spectrum of **15** (700 MHz, C_6D_6).

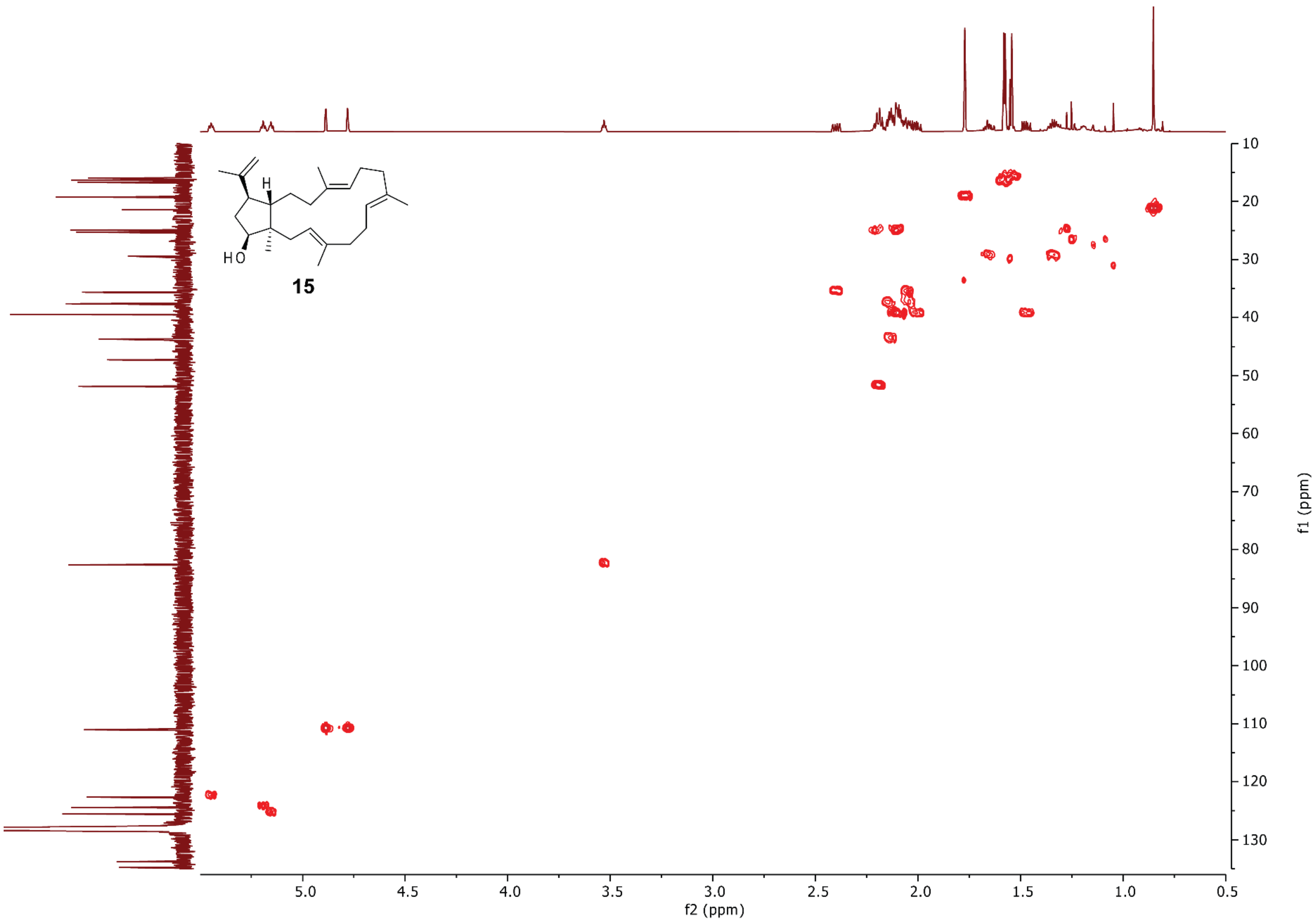

Figure S149. HMQC spectrum of **15** (C_6D_6).

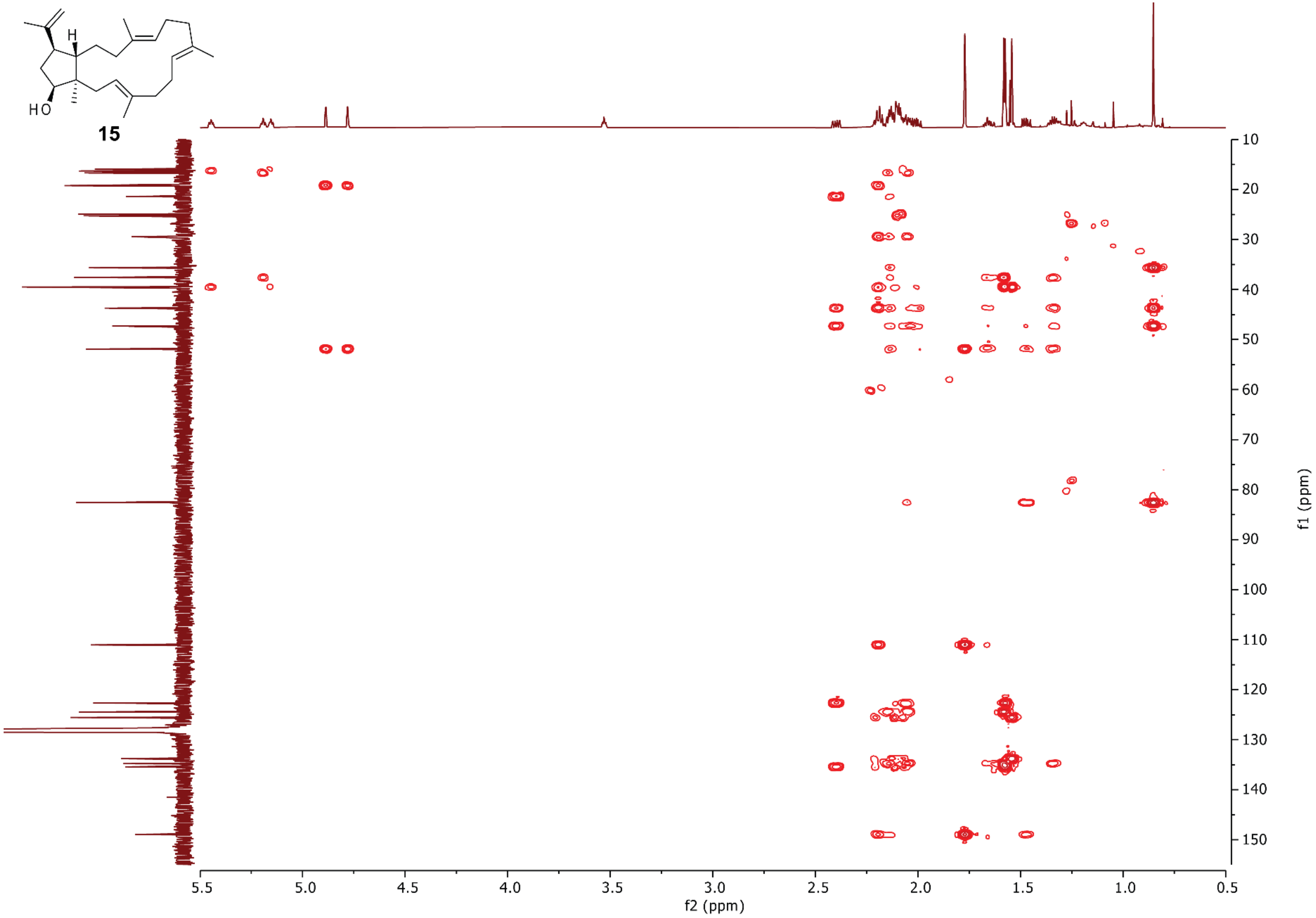

Figure S150. HMBC spectrum of **15** (C_6D_6).

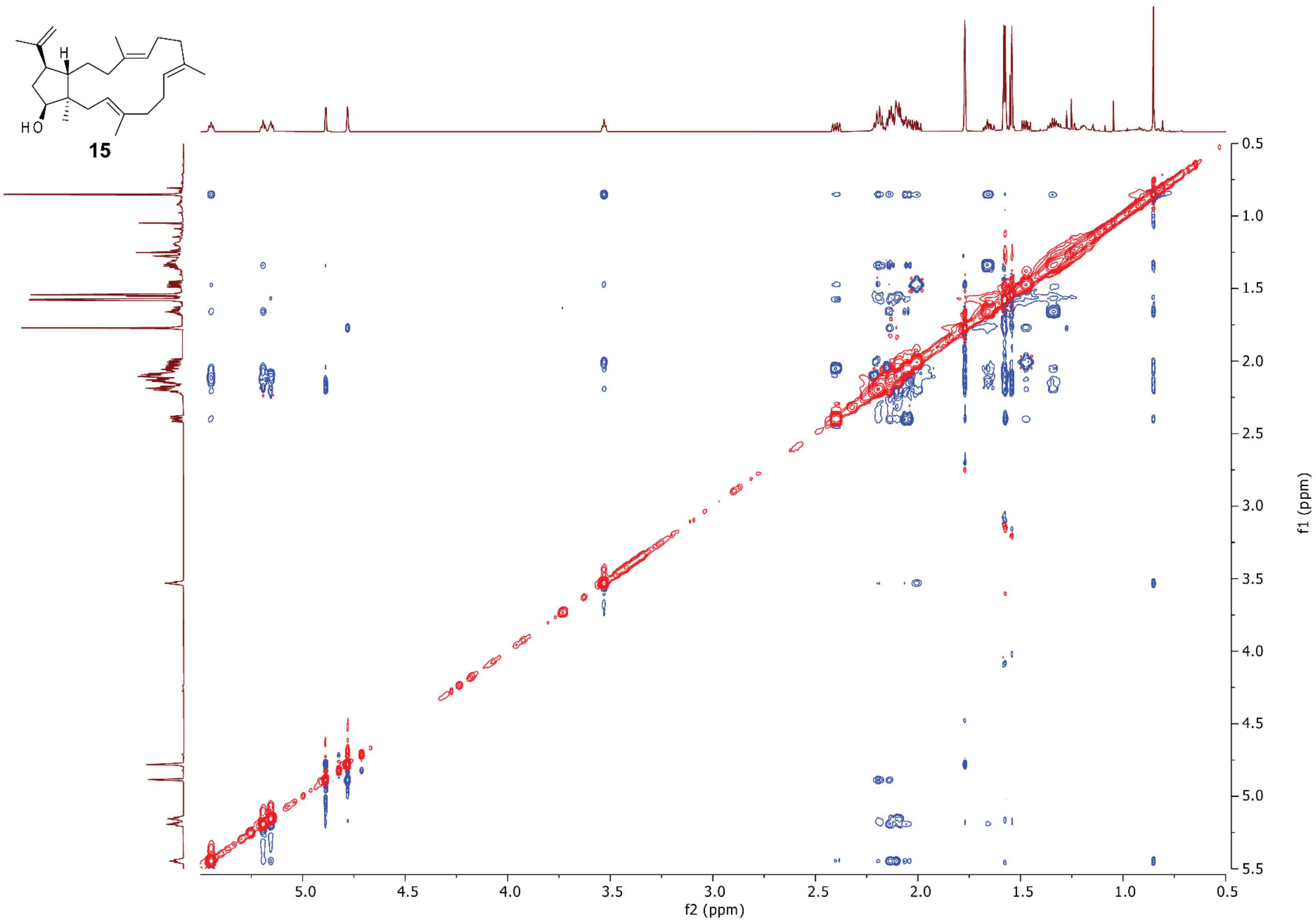

Figure S151. NOESY spectrum of **15** (700 MHz, C_6D_6).

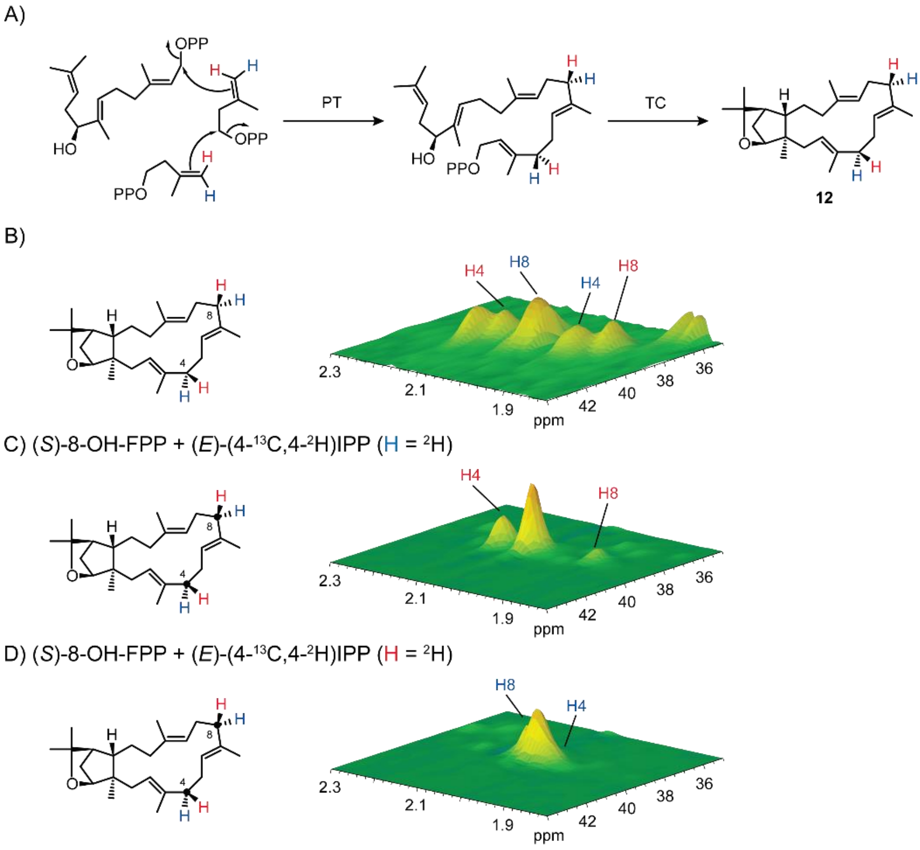

Figure S152. The absolute configuration of **12**. A) Enzymatic reaction of (*S*)-8-OH-FPP and (*E*)- or (*Z*)-(4-^{13}C,4-^{2}H)IPP with AcAS. Partial HSQC spectra showing the regions for C4, C8 of B) unlabelled **12**, C) labelled **12** obtained from (*S*)-8-OH-FPP and (*E*)-(4-^{13}C,4-^{2}H)IPP (blue H = ^{2}H), and D) labelled **12** obtained from (*S*)-8-OH-FPP and (*Z*)-(4-^{13}C,4-^{2}H)IPP (red H = ^{2}H). Colour code of hydrogens corresponds to the same colour code used in Table S11.

A)

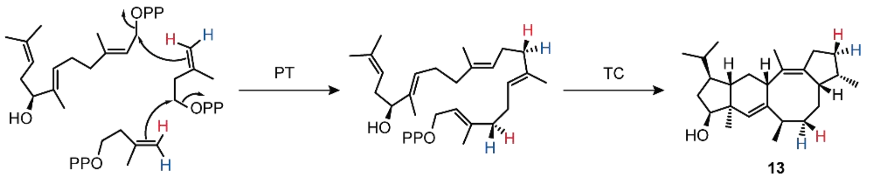

B)

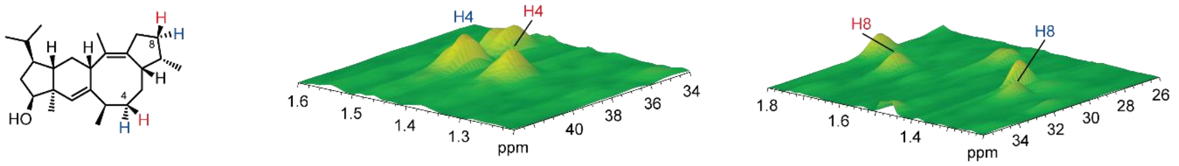

C) (*S*)-8-OH-FPP + (*E*)-(4-^{13}C,4-^{2}H)IPP (H = ^{2}H)

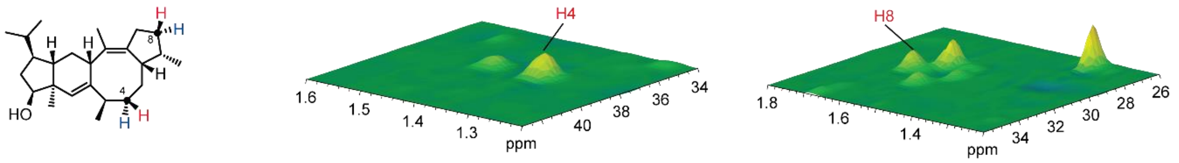

D) (*S*)-8-OH-FPP + (*E*)-(4-^{13}C,4-^{2}H)IPP (H = ^{2}H)

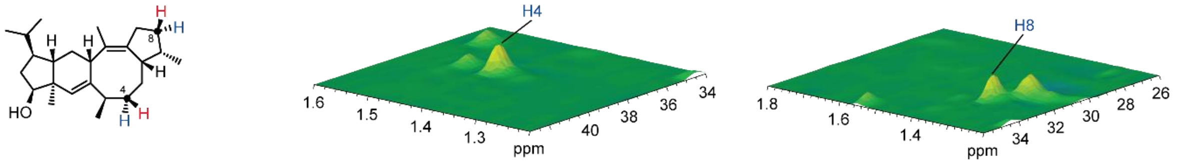

Figure S153. The absolute configuration of **13**. A) Enzymatic reaction of (*S*)-8-OH-FPP and (*E*)- or (*Z*)-(4-^{13}C,4-^{2}H)IPP with AcAS. Partial HSQC spectra showing the regions for C4, C8 of B) unlabelled **13**, C) labelled **13** obtained from (*S*)-8-OH-FPP and (*E*)-(4-^{13}C,4-^{2}H)IPP (blue H = ^{2}H), and D) labelled **13** obtained from (*S*)-8-OH-FPP and (*Z*)-(4-^{13}C,4-^{2}H)IPP (red H = ^{2}H). Colour code of hydrogens corresponds to the same colour code used in Table S12.

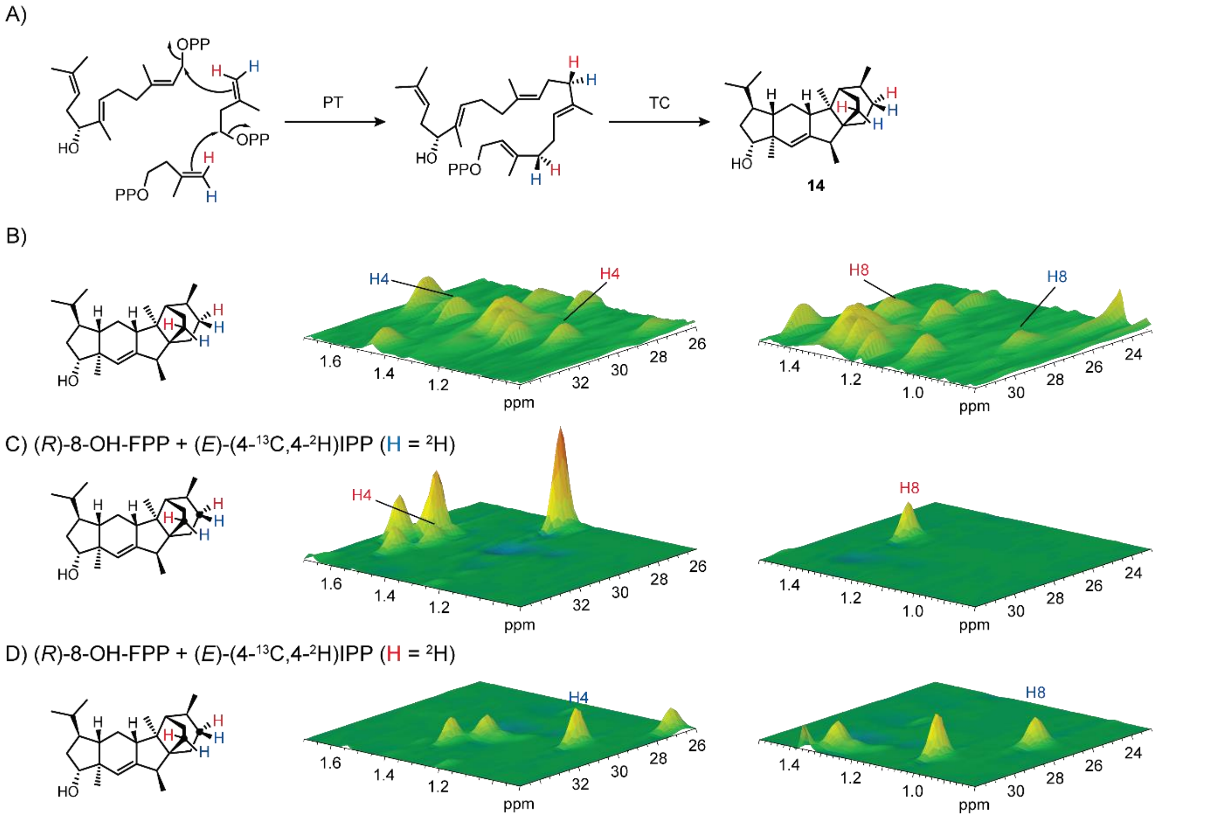

Figure S154. The absolute configuration of **14**. A) Enzymatic reaction of (*R*)-8-OH-FPP and (*E*)- or (*Z*)-(4-^{13}C,4-^{2}H)IPP with AcAS. Partial HSQC spectra showing the regions for C4, C8 of B) unlabelled **14**, C) labelled **14** obtained from (*R*)-8-OH-FPP and (*E*)-(4-^{13}C,4-^{2}H)IPP (blue H = ^{2}H), and D) labelled **14** obtained from (*R*)-8-OH-FPP and (*Z*)-(4-^{13}C,4-^{2}H)IPP (red H = ^{2}H). Colour code of hydrogens corresponds to the same colour code used in Table S13.

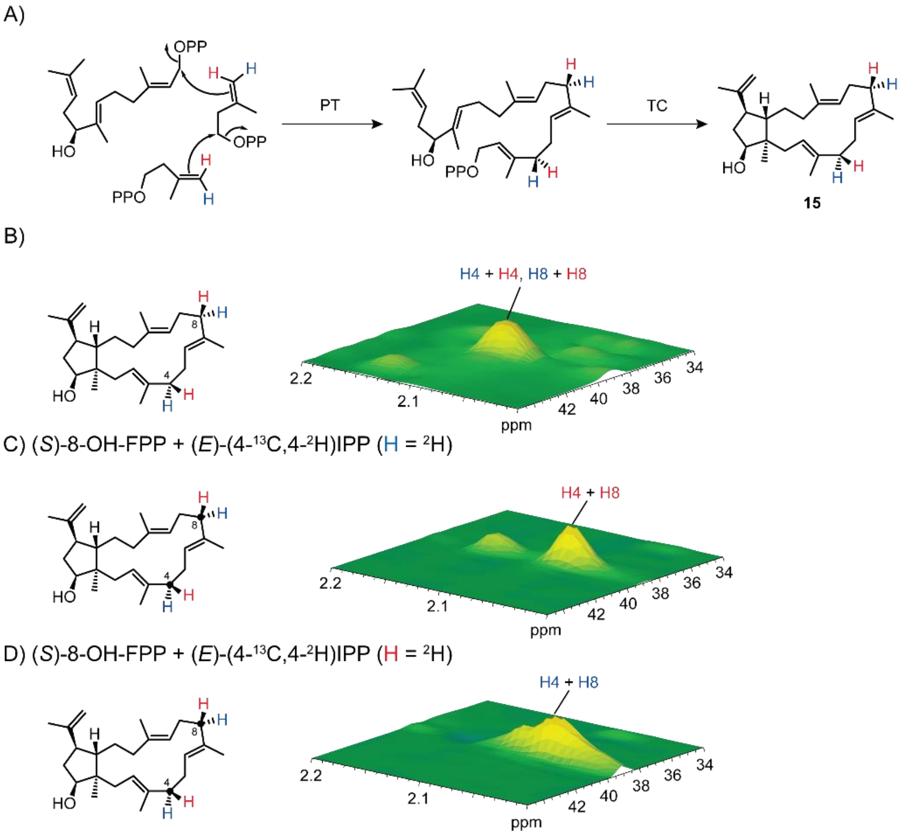

Figure S155. The absolute configuration of **15**. A) Enzymatic reaction of (*S*)-8-OH-FPP and (*E*)- or (*Z*)-(4-^{13}C,4-^{2}H)IPP with AcAS. Partial HSQC spectra showing the regions for C4, C8 of B) unlabelled **15**, C) labelled **15** obtained from (*S*)-8-OH-FPP and (*E*)-(4-^{13}C,4-^{2}H)IPP (blue H = ^{2}H), and D) labelled **15** obtained from (*S*)-8-OH-FPP and (*Z*)-(4-^{13}C,4-^{2}H)IPP (red H = ^{2}H). Colour code of hydrogens corresponds to the same colour code used in Table S14.

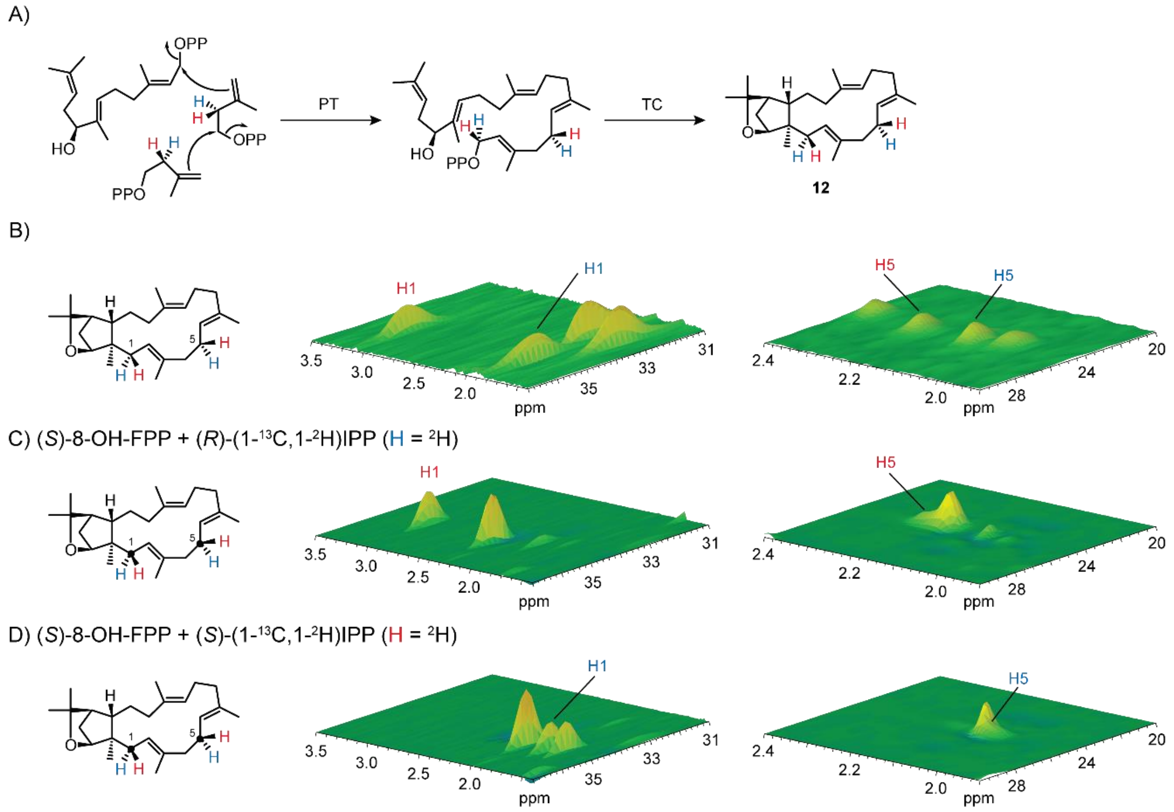

Figure S156. The absolute configuration of **12**. A) Enzymatic reactions of (*S*)-8-OH-FPP and (*R*)- or (*S*)-(1-^{13}C,1-^{2}H)IPP with AcAS. Partial HSQC spectra showing the regions for C1, and C5 of B) unlabelled **12**, C) labelled **12** obtained from (*S*)-8-OH-FPP and (*R*)-(1-^{13}C,1-^{2}H)IPP (blue H = ^{2}H), and D) labelled **12** obtained from (*S*)-8-OH-FPP and (*S*)-(1-^{13}C,1-^{2}H)IPP (red H = ^{2}H). Colour code of hydrogens corresponds to the same colour code used in Table S11.

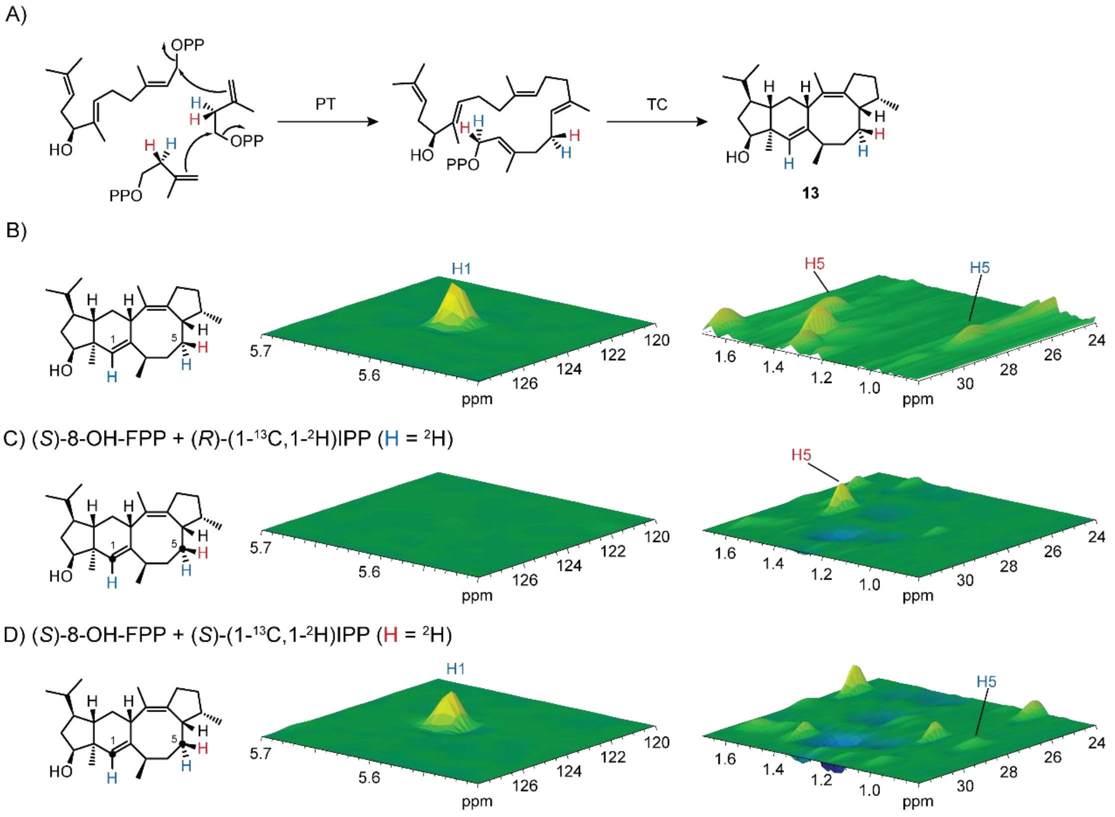

Figure S157. The absolute configuration of **13**. A) Enzymatic reactions of (*S*)-8-OH-FPP and (*R*)- or (*S*)-(1-^{13}C,1-^{2}H)IPP with AcAS. Partial HSQC spectra showing the regions for C1, and C5 of B) unlabelled **13**, C) labelled **13** obtained from (*S*)-8-OH-FPP and (*R*)-(1-^{13}C,1-^{2}H)IPP (blue H = ^{2}H), and D) labelled **13** obtained from (*S*)-8-OH-FPP and (*S*)-(1-^{13}C,1-^{2}H)IPP (red H = ^{2}H). Colour code of hydrogens corresponds to the same colour code used in Table S12.

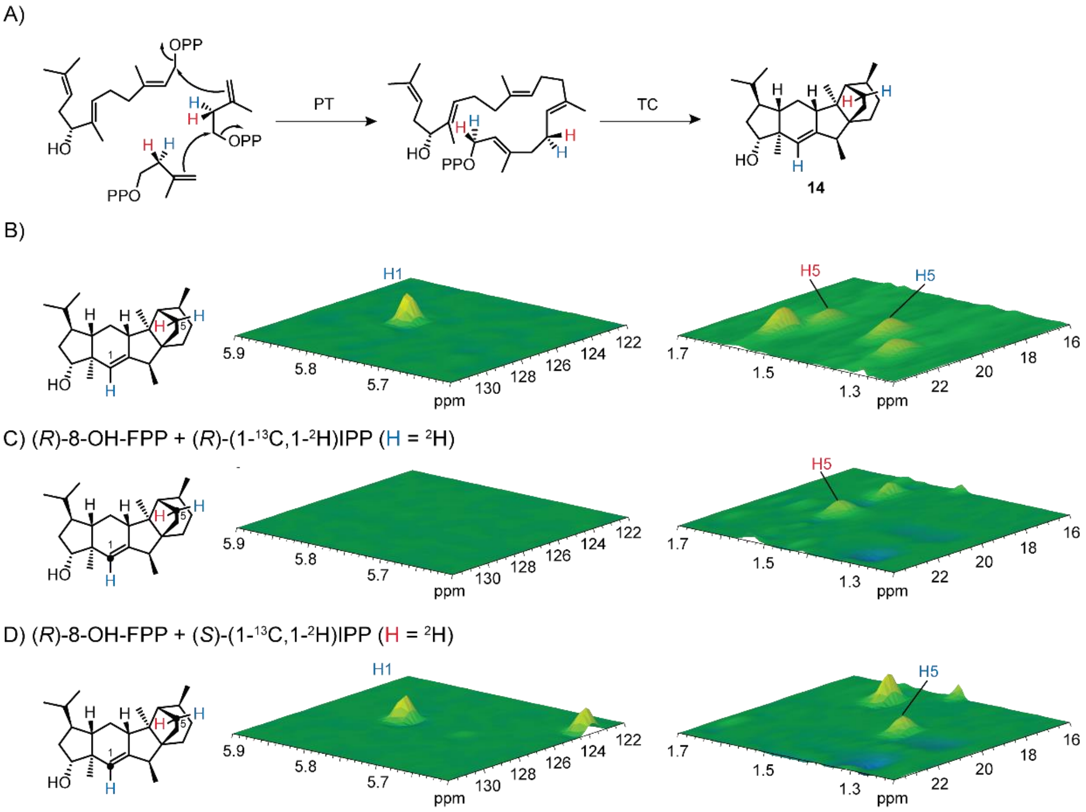

Figure S158. The absolute configuration of **14**. A) Enzymatic reactions of (*R*)-8-OH-FPP and (*R*)- or (*S*)-(1-^{13}C,1-^{2}H)IPP with AcAS. Partial HSQC spectra showing the regions for C1, and C5 of B) unlabelled **14**, C) labelled **14** obtained from (*R*)-8-OH-FPP and (*R*)-(1-^{13}C,1-^{2}H)IPP (blue H = ^{2}H), and D) labelled **14** obtained from (*R*)-8-OH-FPP and (*S*)-(1-^{13}C,1-^{2}H)IPP (red H = ^{2}H). Colour code of hydrogens corresponds to the same colour code used in Table S13.

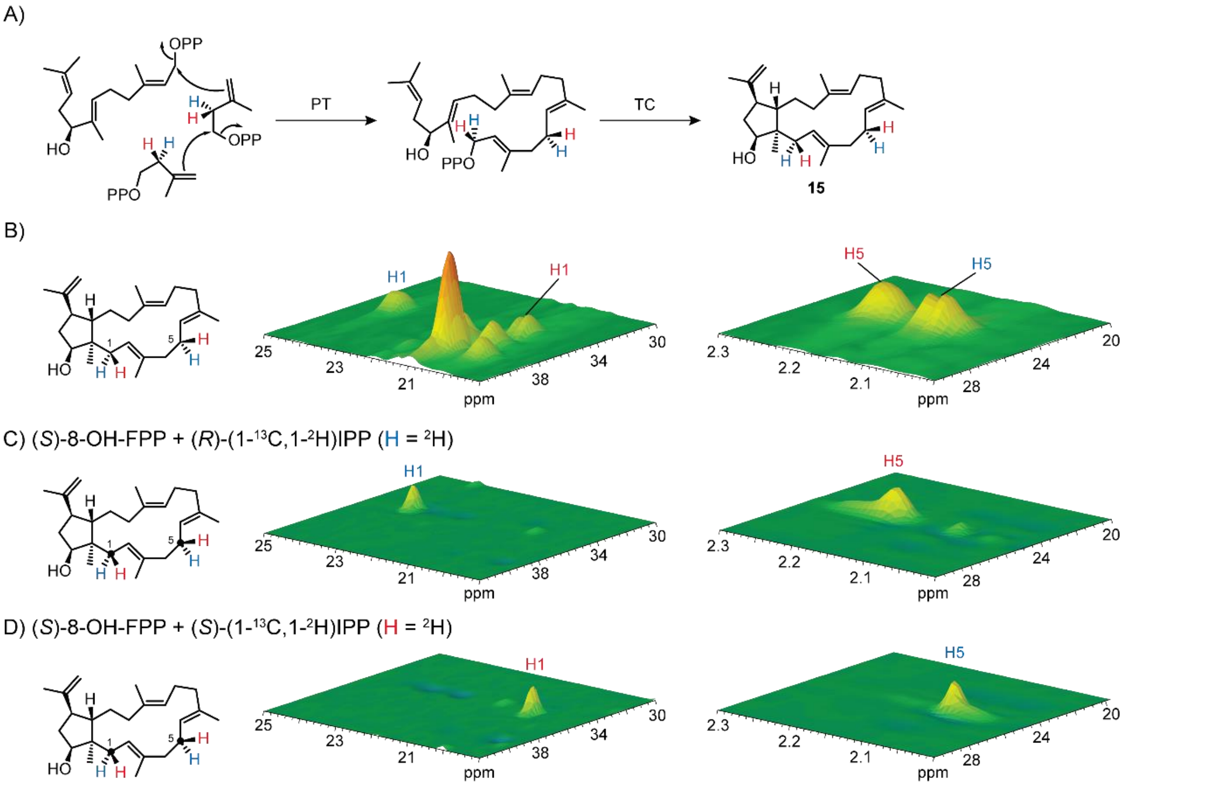

Figure S159. The absolute configuration of **15**. A) Enzymatic reactions of (*S*)-8-OH-FPP and (*R*)- or (*S*)-(1-^{13}C,1-^{2}H)IPP with AcAS. Partial HSQC spectra showing the regions for C1, and C5 of B) unlabelled **15**, C) labelled **15** obtained from (*S*)-8-OH-FPP and (*R*)-(1-^{13}C,1-^{2}H)IPP (blue H = ^{2}H), and D) labelled **15** obtained from (*S*)-8-OH-FPP and (*S*)-(1-^{13}C,1-^{2}H)IPP (red H = ^{2}H). Colour code of hydrogens corresponds to the same colour code used in Table S14.

References

[1] Z. Quan, J. S. Dickschat, *Org. Lett.* **2020**, *22*, 7552.
[2] G. R. Fulmer, A. J. M. Miller, N. H. Sherden, H. E. Gottlieb, A. Nudelman, B. M. Stoltz, J. E. Bercaw, K. I. Goldberg, *Organometallics* **2010**, *29*, 2176.
[3] Y. Ye, A. Minani, A. Mandi, C. Liu, T. Taniguchi, T. Kuzuyama, K. Monde, K. Gomi, H. Oikawa, *J. Am. Chem. Soc.* **2015**, *137*, 11846.
[4] K. Narita, H. Sato, A. Minami, K. Kudo, L. Gao, C. Liu, T. Ozaki, M. Kodama, X. Lei, T. Taniguchi, K. Monde, M. Yamazaki, M. Uchiyama, H. Oikawa, *Org. Lett.* **2017**, *19*, 6696.
[5] M. Okada, Y. Matsuda, T. Mitsuhashi, S. Hoshino, T. Mori, K. Nakagawa, Z. Quan, B. Qin, H. Zhang, F. Hayashi, H. Kawaide, I. Abe, *J. Am. Chem. Soc.* **2016**, *138*, 10011.
[6] T. Mitsuhashi, J. Rinkel, M. Okada, I. Abe, J. S. Dickschat, *Chem. Eur. J.* **2017**, *23*, 10053.
[7] R. Chiba, A. Minami, K. Gomi, H. Oikawa, *Org. Lett.* **2013**, *15*, 594.
[8] Z. Quan, J. S. Dickschat, *Org. Biomol. Chem.* **2020**, *18*, 6072.
[9] Y. Matsuda, T. Mitsuhashi, Z. Quan, I. Abe, *Org. Lett.* **2015**, *17*, 4644.
[10] Y. Matsuda, T. Mitsuhashi, S. Lee, M. Hoshino, T. Mori, M. Okada, H. Zhang, F. Hayashi, M. Fujita, I. Abe, *Angew. Chem. Int. Ed.* **2016**, *55*, 5785.
[11] J. Huang, J. Lv, Q. Wang, J. Zou, Y. Lu, Q. Wang, D. Chen, X. Yao, H. Gao, D. Hu, *Org. Biomol. Chem.* **2019**, *17*, 248.
[12] J. Guo, Y. Cai, F. Cheng, C. Yang, W. Zhang, W. Yu, J. Yan, Z. Deng, K. Hong, *Org. Lett.* **2021**, *23*, 1525.
[13] C. M. Starks, K. Back, J. Chappell, J. P. Noel, *Science* **1997**, *277*, 1815.
[14] E. Y. Shishova, L. Di Costanzo, D. E. Cane, D. W. Christianson, *Biochemistry* **2007**, *46*, 1941.
[15] P. Baer, P. Rabe, K. Fisher, C. A. Citron, T. A. Klapschinski, M. Groll, J. S. Dickschat, *Angew. Chem. Int. Ed.* **2014**, *53*, 7652.
[16] J. S. Dickschat, *Nat. Prod. Rep.* **2016**, *33*, 87–110.
[17] J. Sambrook, D. W. Russell, *Cold Spring Harb Protoc.* **2006**, *1*, pdb-prot4455.
[18] K. Murai, L. Lauterbach, K. Teramoto, Z. Quan, L. Barra, T. Yamamoto, K. Nonaka, K. Shiomi, M. Nishiyama, T. Kuzuyama, J. S. Dickschat, *Angew. Chem. Int. Ed.* **2019**, *58*, 15046.
[19] K. Gomi, Y. Iimura, S. Hara, *Agric. Biol. Chem.* **1987**, *51*, 2549.
[20] M. M. Bradford, *Anal. Biochem.* **1976**, *72*, 248.
[21] X. Robert, P. Gouet, *Nucleic Acids Res.* **2014**, *42*, W320.
[22] R. D. Giets, R. H. Schiestl, *Nat. Protoc.* **2007**, *2*, 31.
[23] J. Rinkel, L. Lauterbach, P. Rabe, J. S. Dickschat, *Angew. Chem. Int. Ed.* **2018**, *57*, 3238.
[24] L. Lauterbach, J. Rinkel, J. S. Dickschat, *Angew. Chem. Int. Ed.* **2018**, *57*, 8280.
[25] J. Rinkel, J. S. Dickschat, *Org. Lett.* **2019**, *21*, 2426.
[26] P. Rabe, J. Rinkel, E. Dolja, T. Schmitz, B. Nubbemeyer, T. H. Luu, J. S. Dickschat, *Angew. Chem. Int. Ed.* **2017**, *56*, 2776.
[27] J. Rinkel, L. Lauterbach, J. S. Dickschat, *Angew. Chem. Int. Ed.* **2019**, *58*, 452.
[28] P. Rabe, L. Barra, J. Rinkel, R. Riclea, C. A. Citron, T. A. Klapschinski, A. Janusko, J. S. Dickschat, *Angew. Chem. Int. Ed.* **2015**, *54*, 13448.
[29] A. Hou, J. S. Dickschat, *Angew. Chem. Int. Ed.* **2020**, *59*, 19961.
[30] J. Donath, W. Boland, *Phytochemistry.* **1995**, *39*, 785.
[31] S. Grimme, S. Ehrlich, L. Goerigk, *J. Comp. Chem.* **2011**, *32*, 1456.
[32] M. J. Frisch, et al. in *Gaussian 16*, *Revision B.01*, Gaussian Inc., Wallingford CT, **2016**.
[33] a) Y. J. Hong, D. J. Tantillo, *J. Org. Chem.* **2018**, *83*, 3780; b) C. Adamo, V. Barone, *J. Chem. Phys.* **1998**, *108*, 664; c) S. P. T. Matsuda, W. K. Wilson, Q. Xiong, *Org. Biomol. Chem.* **2006**, *4*, 530; d) L. Lauterbach, B. Goldfuss, J. S. Dickschat, *Angew. Chem. Int. Ed.* **2020**, *59*, 11943.

[34] a) S. Grimme, *Chem. Eur. J.* **2012**, *18*, 9955; b) G. Luchini, J. V. Alegre-Requena, Y. Guan, I. Funes-Ardoiz, R. S. Paton in *GoodVibes v3.0.1*, **2019**.
[35] H. He, G. Bian, C. J. Herbst-Gervasoni, T. Mori, S. A. Shinsky, A. Hou, X. Mu, M. Huang, S. Cheng, Z. Deng, D. W. Christianson, I. Abe, T. Liu, *Nat. Commun.* **2020**, *11*, 3958.
[36] R. Higuchi, B. Krummel, R. Saiki, *Nucleic Acids Res.* **1988**, *16*, 7351.
